# Discovery and Optimization of Ergosterol Peroxide Derivatives as Novel Glutaminase 1 Inhibitors for the Treatment of Triple-Negative Breast Cancer

**DOI:** 10.3390/molecules29184375

**Published:** 2024-09-14

**Authors:** Ran Luo, Haoyi Zhao, Siqi Deng, Jiale Wu, Haijun Wang, Xiaoshan Guo, Cuicui Han, Wenkang Ren, Yinglong Han, Jianwen Zhou, Yu Lin, Ming Bu

**Affiliations:** 1 College of Pharmacy, Qiqihar Medical University, Qiqihar 161006, China; luoran1224@163.com (R.L.); zhaohaoyi1989@126.com (H.Z.); 17735745701@163.com (S.D.); wanghaijun@qmu.edu.cn (H.W.); xiaoshan990519@163.com (X.G.); hancuicui-1234@163.com (C.H.); 15039417930@163.com (W.R.); 15776267895@163.com (Y.H.); 2 College of Pharmacy, Hainan University, Haikou 570228, China; wwwwjl55@163.com; 3 Research Institute of Medicine & Pharmacy, Qiqihar Medical University, Qiqihar 161006, China; zhoujianwen123@qmu.edu.cn

**Keywords:** ergosterol peroxide derivatives, GLS1 inhibitors, antitumor, TNBC

## Abstract

In this study, novel ergosterol peroxide (EP) derivatives were synthesized and evaluated to assess their antiproliferative activity against four human cancer cell lines (A549, HepG2, MCF-7, and MDA-MB-231). Compound **3g** exhibited the most potent antiproliferative activity, with an IC_50_ value of 3.20 µM against MDA-MB-231. This value was 5.4-fold higher than that of the parental EP. Bioassay optimization further identified **3g** as a novel glutaminase 1 (GLS1) inhibitor (IC_50_ = 3.77 µM). In MDA-MB-231 cells, **3g** reduced the cellular glutamate levels by blocking the glutamine hydrolysis pathway, which triggered reactive oxygen species production and induced caspase-dependent apoptosis. Molecular docking indicated that **3g** interacts with the reaction site of the variable binding pocket by forming multiple interactions with GLS1. In a mouse model of breast cancer, **3g** showed remarkable therapeutic effects at a dose of 50 mg/kg, with no apparent toxicity. Based on these results, **3g** could be further evaluated as a novel GLS1 inhibitor for triple-negative breast cancer (TNBC) therapy.

## 1. Introduction

Glutamine was the most abundant non-essential amino acid in human serum and provided carbon and nitrogen sources for cancer cells in the process of tumor occurrence and development. It was used for cancer cell biosynthesis, providing energy for cancer cells and promoting tumor growth [1,2,3,4,5]. Glutamine was hydrolyzed by glutaminase (GLS) to produce glutamic acid, which was then catalyzed by glutamine dehydrogenase to produce α-ketoglutaric acid. Glutamine then entered the tricarboxylic acid (TCA) cycle to provide nutrients for tumor cells [6,7,8,9]. The glutamine hydrolysis pathway could also adjust the reactive oxygen species (ROS) levels to regulate the antioxidant defense functions in cells, thereby protecting cells from oxidative stress [10,11,12]. Several human cancer cell lines showed sensitivity to glutamine starvation, including ovarian carcinoma, pancreatic cancer, breast cancer, glioblastoma multiforme, acute myeloid leukemia, and small-cell lung cancer. Blocking glutamine hydrolysis was, therefore, a viable cancer treatment strategy. Glutamine was hydrolyzed by glutaminase 1 (GLS1), which was a key metabolic enzyme in glutamine hydrolysis. Indeed, inhibiting GLS1 activity could effectively suppress the occurrence of tumors [13,14,15,16,17]. Therefore, the development of novel GLS1 inhibitors became a research focus in recent years.

**Figure 1:**
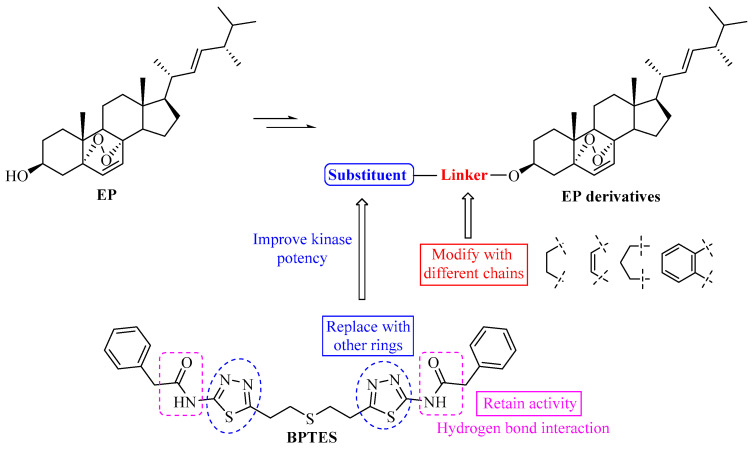
Design of GLS1 inhibitors based on the EP and BPTES binding groups.

Natural products had good medicinal value and became one of the main choices for the development of new anticancer drugs [18]. Ergosterol peroxide (EP, **1**) (Figure 1), as a natural steroid compound extracted from the traditional Chinese medicine *Ganoderma lucidum*, received extensive attention in recent years [19]. It had a variety of biological effects, including anti-inflammatory, antitumor, antioxidant and immunosuppressive activities [20,21,22]. Among these activities, the antitumor activity of EP was the most significant, and it had obvious inhibitory effects on breast cancer, lung cancer, colorectal cancer, ovarian cancer, gastric cancer, hepatocellular carcinoma and prostate cancer [23,24,25,26,27,28,29]. In recent years, our group explored the mechanisms and derivatization of EP. Many highly active EP derivatives were obtained in our studies with derivatization on the C-3 position of EP [30,31,32]. In addition, in our previous studies, it was found that EP offers moderate GLS1-inhibitory activity (IC_50_ = 33.67 μM). Therefore, with reasonable structural modifications, EP, as the lead compound, can be used by us to develop new and efficient GLS1 inhibitors.

BPTES was the first reported small-molecule inhibitor of GLS1 with a symmetrical structure consisting of thiadiazole, amide, and phenyl [33,34,35]. A large number of derivatives that significantly inhibited GLS1 had been synthesized in structural derivatization studies based on the skeleton structure of BPTES [36,37,38]. A structure–activity relationship analysis showed that the amide group was an important pharmacophore for binding and that the thiadiazole group at its end could be replaced with a variety of substituents, without affecting the GLS1-inhibitory activity of the compound [39]. The benzene ring also featured a large space for modification [40]. Based on the idea of molecular hybridization, important groups or substituents from the structure of BPTES and its derivatives were introduced into EP by us to improve the antitumor and enzyme-inhibitory activities of EP derivatives. At the same time, it was also considered by us that the introduction of large sterically hindered groups into the C-3 hydroxyl group of EP might have decreased its biological activity. Therefore, different linkers with varying lengths and structures were designed by us to connect EP and BPTES substituents and the influence of the chemical space on the derivatives’ biological activities was examined. The structural design strategy for the new EP derivative is shown in Figure 1.

In the present study, diverse substituents derived from BPTES were introduced to the 3-hydroxyl group of EP through different linkers. Consequently, a novel compound was identified, which demonstrated enhanced GLS1-inhibitory activity and held potential as a candidate for cancer therapy.

## 2. Results and Discussion

### 2.1. Chemistry

**Scheme 1:**
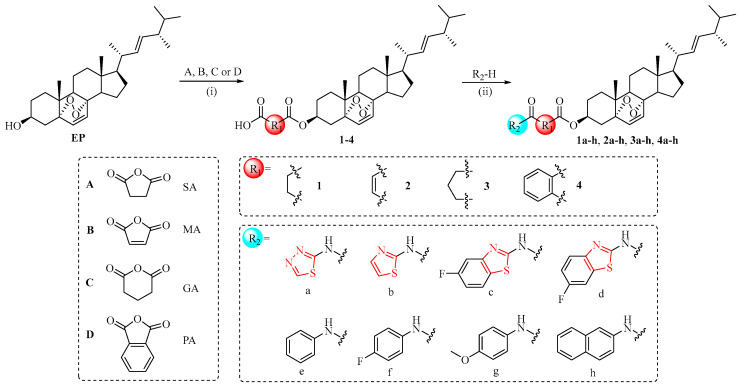
Synthesis of EP derivatives (**1a**–**h**, **2a**–**h**, **3a**–**h**, and **4a**–**h**). Reagents and conditions: (i) Et_3_N, CH_2_Cl_2_, SA (A), MA (B), GA (C), or PA (D), reflux, 24 h, 76–85%; and (ii) R_2_-H, HOBT·H_2_O, EDCI·HCl, pyridine, DMF, room temperature, 12–48 h, 77–88%.

As shown in Scheme 1 and detailed in the Experimental Section, the synthetic pathways employed for the preparation of the key intermediates **1**–**4** and the target compounds **1a**–**h**, **2a**–**h**, **3a**–**h**, and **4a**–**h** were presented. Initially, in dichloromethane (CH_2_Cl_2_) under reflux conditions, the 3-hydroxyl group of EP was subjected to acylation with succinic anhydride (SA), maleic anhydride (MA), glutaric anhydride (GA), or phthalic anhydride (PA), thereby yielding the intermediates, namely **1**–**4**. Subsequently, the intermediates were treated with appropriate amino compounds (R_2_-H) in the presence of the catalysts 1-hydroxybenzotriazole monohydrate (HOBT·H_2_O), 1-ethyl-3-(3-dimethylaminopropyl) carbodiimide hydrochloride (EDCI·HCl), and pyridine (Py) in dimethylformamide (DMF) at room temperature to obtain the target compounds **1a**–**h**, **2a**–**h**, **3a**–**h**, and **4a**–**h**.

### 2.2. In Vitro Antiproliferative Activity of EP Derivatives

The in vitro antiproliferative activity of the novel EP derivatives (**1a**–**h**, **2a**–**h**, **3a**–**h**, and **4a**–**h**) was evaluated on four different human cancer cell lines (A549, HepG2, MCF-7, and MDA-MB-231) using an MTT assay. The experimental results are summarized in Table 1. Compared with EP, most of the compounds exhibited better cytotoxic activities against almost all the cell lines. Compound **3g** exhibited the most potent inhibitory activity, with an IC_50_ value of 3.20 µM against the MDA-MB-231 cell line, and it had 5.4-fold higher activity than EP (IC_50_ = 17.26 µM).

The current data suggest that when the hydroxyl at the C-3 atom was substituted with an ester linkage, most EP derivatives offered better inhibitory activity than EP against all four tumor cell lines. The preliminary structure–activity relationship analysis was as follows. Compounds **1a**, **1b**, **2a**, **2b**, **3a**, **3b**, **4a**, and **4b** containing either a thiadiazole ring or a thiazole ring exhibited improved activity, while the introduction of benzothiazole presented relatively weak inhibition activity (**1c**, **1d**, **2c**, **2d**, **3c**, **3d**, **4c**, and **4d**). Meanwhile, compounds with high lipophilic group content, such as compounds **1h**, **2h**, **3h**, and **4h**, showed relatively moderate cytotoxicity.

Furthermore, the replacement of the heterocyclic ring with a phenyl ring could contribute to the enhancement of the inhibitory activity (compounds **1e**, **2e**, **3e**, and **4e**). Consequently, the phenyl ring of the substituents was retained, and EP derivatives possessing a phenyl ring were given priority. The subsequent introduction of an electron-donating methoxy group into compounds **1g**, **2g**, **3g**, and **4g** led to an improvement in their inhibitory activity.

For the purpose of further validating our design strategy aimed at enhancing the inhibitory activities, four linkers were chosen. Compounds **3e**, **3g**, and **3h** exhibited the highest inhibitory activity when the chain length of the linker region included five carbon atoms (such as glutaric acid). However, introducing a large sterically hindered linker (such as phthalic acid) into the 3-hydroxyl of EP decreased the antiproliferative activity (i.e., compounds **4f**, **4g**, and **4h**). These results implied that the flexibility of the linker, which determined the spatial orientation of the substituent portion, was crucial for the inhibitory activities. Additionally, these linkers comprised two carbonyl groups that were connected to the substituents through an amide linkage, and this might have offered a binding group for protein–ligand interactions.

Overall, structural modification of EP generated the most potent derivative **3g**, in which a 3-hydroxyl group was modified with the lipophilic substituent 4-methoxyphenyl via the linker glutaric acid. Based on these data, we further investigated **3g** as a potential anticancer agent. Triple-negative breast cancer (TNBC) was the most aggressive molecular subtype of breast tumors [41,42,43], with high levels of GLS1 and increased glutamine uptake, providing cells with intermediates that met the TCA cycle [44,45]. Based on the potent inhibitory effects observed in MDA-MB-231 cells, we evaluated the inhibitory effects of GLS1 in order to further characterize compound **3g**.

### 2.3. Compound ***3g*** Inhibited the GLS1 Activity of MDA-MB-231 Cells

**Figure 2:**
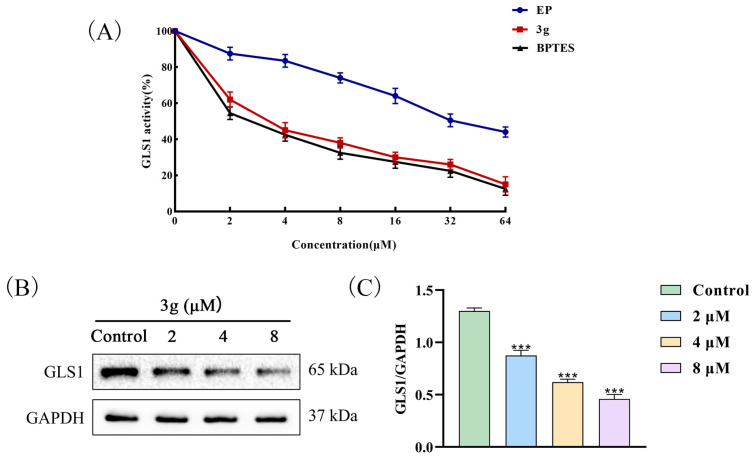
The inhibitory effect of compound **3g** on GLS1 activity in MDA-MB-231 cells. (**A**) A GLS1 inhibitor screening kit was utilized to detect the GLS1 levels of EP, **3g**, and BPTES. (**B**) After treating MDA-MB-231 cells with different concentrations of **3g** for 48 h, the expression of the GLS1 protein was detected by Western blot. (**C**) Quantitative analysis. The data are expressed as the mean ± SD (*n* = 3), *** *p* < 0.001, compared with the control group.

The GLS1 inhibitory activity of compound **3g** in MDA-MB-231 cells was evaluated using a GLS1 inhibitor screening kit. The parent structure EP and BPTES was used as a reference, and the results are depicted in Figure 2A. Interestingly, both **3g** and EP significantly suppressed the activity of GLS1. Notably, **3g** inhibited GLS1 significantly more than EP (IC_50_ = 33.67 μM) in MDA-MB-231 cells (IC_50_ = 3.77 μM), and the GLS1 inhibitory effect of **3g** was similar to that of BPTES (IC_50_ = 3.23 μM). Western blot analysis also yielded the same results (Figure 2B,C). Therefore, **3g** was worthy of further study to determine its bioactivity and pharmacological mechanisms as a new antitumor drug candidate.

### 2.4. Compound ***3g*** Inhibited the Colony Formation of MDA-MB-231 Cells

**Figure 3:**
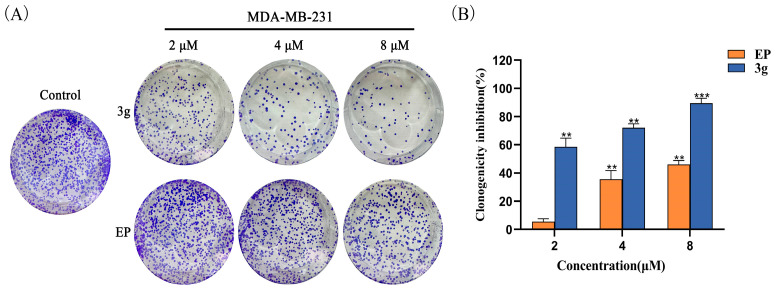
Compound **3g** inhibited the proliferation of MDA-MB-231 cells. (**A**) Compound **3g** inhibited the colony formation of MDA-MB-231 cells. (**B**) Clonogenic suppression expressed as a percentage relative to the vehicle-treated controls. Data represent the mean ± SD (*n* = 3), ** *p* < 0.01, *** *p* < 0.001, compared with the control group.

To explore the inhibitory effect of **3g** on the proliferation of MDA-MB-231 cells, we treated MDA-MB-231 cells with different concentrations of **3g** and EP. The results illustrated in Figure 3 showed that **3g** had a significant inhibitory effect on the proliferation of MDA-MB-231 cells, with inhibitory abilities greater than those of EP. Compared with the blank control group, the proliferation inhibition rate of **3g** against MDA-MB-231 cells at 2 μM reached 56.27%.

### 2.5. Compound ***3g*** Induced Apoptosis in MDA-MB-231 Cells

**Figure 4:**
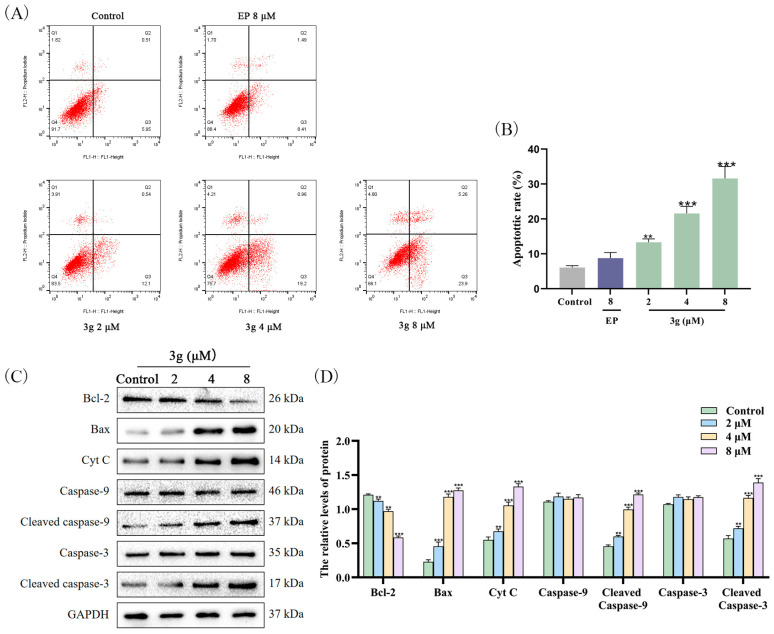
Compound **3g** induced apoptosis of MDA-MB-231 cells. (**A**) MDA-MB-231 cells were treated with different concentrations of **3g** for 48 h; then, the cells were fixed and stained with Annexin V-FITC/PI and analyzed via flow cytometry. Annexin V-FITC and PI data are expressed as percentages (%) for each quadrant. (**B**) The apoptosis rate was quantitatively detected. (**C**) Western blot analysis. MDA-MB-MB-231 cells were treated with different concentrations of **3g** for 48 h, and the protein expressions of Bcl-2, Bax, Cyt C, caspase-9, cleaved caspase-9, caspase-3, and cleaved caspase-3 were detected via Western blot. (**D**) Quantitative analysis. Data represent the means ± SD (*n* = 3), ** *p* < 0.01, *** *p* < 0.001, compared with the control group.

To determine whether the decrease in MDA-MB-231 cell viability observed after **3g** treatment was related to apoptosis, we treated MDA-MB-231 cells with **3g** and assessed the changes in apoptosis. Annexin V-FITC/PI double staining showed that apoptosis of MDA-MB-231 cells increased significantly after **3g** treatment (Figure 4A,B). Additionally, Western blot analysis revealed that the expression of B-cell lymphoma-2-associated X (Bax), cytochrome c (Cyt C), cleaved caspase-9, and cleaved caspase-3 was significantly upregulated and that **3g** decreased the expression of B-cell lymphoma-2 (Bcl-2) (Figure 4C,D). These results suggest that **3g** may induce apoptosis in MDA-MB-231 cells with overexpressed GLS1 by regulating the mitochondrial apoptotic pathway.

### 2.6. Compound ***3g*** Inhibited Glutamate Production in MDA-MB-231 Cells

**Figure 5:**
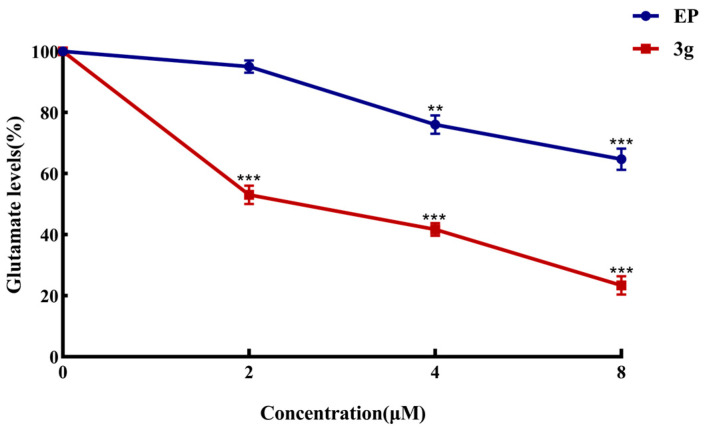
The effect of compound **3g** and EP on the glutamate levels in MDA-MB-231 cells. MDA-MB-231 cells were treated with different concentrations of **3g** (2, 4, and 8 μM) and EP for 48 h. The changes in the glutamate levels of MDA-MB-231 cells were detected by using a glutamate kit. Data represent the means ± SD (*n* = 3), ** *p* < 0.01, *** *p* < 0.001, compared with the control group.

The content of glutamate in MDA-MB-231 cells was detected using Glutamate Assay Kit. This experiment was conducted in accordance with the manufacturer’s instructions. During the experiment, compound **3g** reduced the production of glutamate in MDA-MB-231 cells in a dose-dependent manner (Figure 5). Compared with EP, **3g** exhibited a more effective inhibitory effect on glutamate in MDA-MB-231 cells. These results suggest that **3g** can effectively inhibit GLS1, thereby blocking the glutamine hydrolysis pathway and inducing cell death.

### 2.7. Compound ***3g*** Induced ROS Generation in MDA-MB-231 Cells

**Figure 6:**
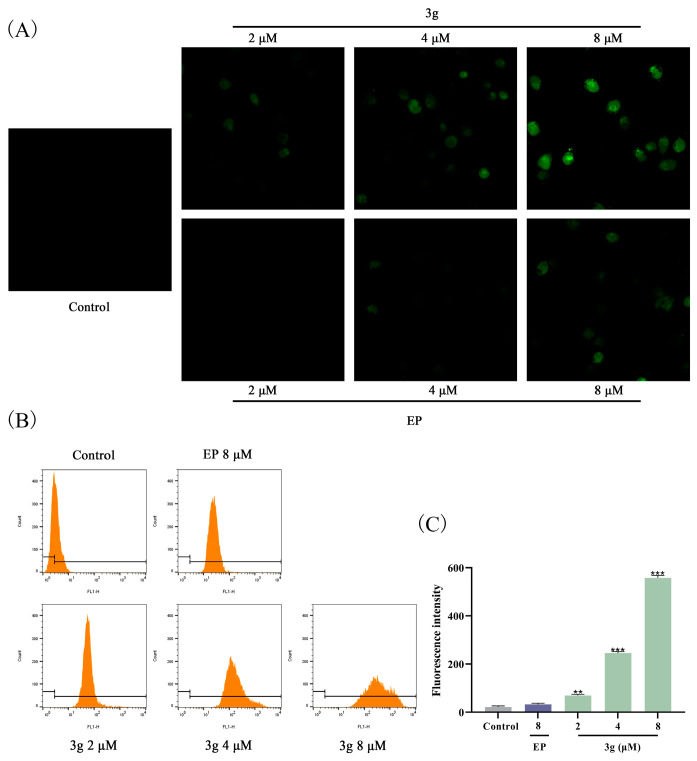
Compound **3g** induced an increase in the ROS levels in MDA-MB-231 cells. MDA-MB-231 cells were subjected to treatment with **3g** and EP at different concentrations for 48 h. (**A**) Fluorescence microscopy image of intracellular ROS production in MDA-MB-231 cells stained with DCFH-DA (green). (**B**) Quantification of ROS levels by flow cytometry. (**C**) Quantitative analysis. Data represent the means ± SD (*n* = 3), ** *p* < 0.01, *** *p* < 0.001, compared with the control group.

Excessive ROS in tumor cells can induce tumor cell apoptosis. Nevertheless, the ROS levels in tumor cells were often influenced by glutamine. Thus, the regulation of glutamine metabolism was crucial for ROS homeostasis. In this study, laser confocal microscopy and flow cytometry were employed to detect the ROS level. As shown in Figure 6, **3g** significantly elevated the intracellular ROS level in MDA-MB-231 cells in a dose-dependent manner, with stronger effects than those of EP. These results indicate that **3g**, as a GLS1 inhibitor, can effectively inhibit glutamine hydrolysis, thereby leading to an increase in ROS.

### 2.8. Compound ***3g*** Docked with GLS1 Molecule

**Figure 7:**
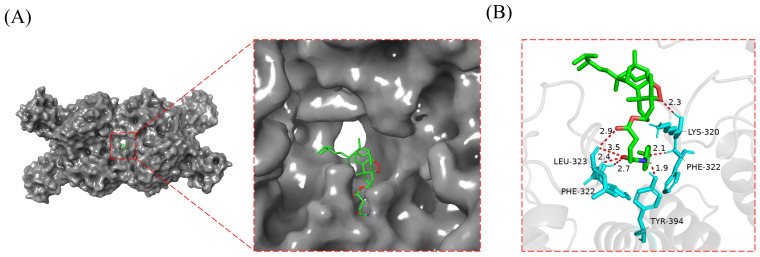
The eutectic structure of compound **3g** with GLS1 (PDB ID: 3UO9). (**A**) Modeled and enlarged close-up of the surface mosaic of the GLS1 tetramer and **3g** binding. (**B**) Close-up of the **3g** interactions in the GLS1 allosteric binding pocket. Here, **3g** is rendered as a rod and colored according to the atom type. Green denotes carbon, blue denotes nitrogen, and red denotes oxygen. The key residual atoms in GLS1 that interacted with the compound are denoted in cyan. The red dashed lines indicate hydrogen bonds, and the numbers are the hydrogen bond lengths.

GLS1 usually exists as a dimer or tetramer, where the tetramer is catalytically active and the dimer is inactive. The tetramer is structurally composed of four molecules with an asymmetric unit characterized by two sets of interfaces. In order to investigate the binding mode of compound **3g** with GLS1, molecular docking studies were carried out based on the crystal structure of GLS1 (PDB: 3UO9), and it was found that **3g** interacts with different reaction sites in the allosteric pocket of GLS1 (Figure 7). The amino acid residues TRY-394, PHE-322, and LEU-323 form hydrogen bonding connections with the amide and ester groups in compound **3g**. This hydrogen bonding interaction drives the linker and substituent deep into the narrow cavity of the GLS1 tetramer, which in turn orients 4-methoxyaniline to the other reactive site to form a hydrogen bonding interaction with PHE-322. At the same time, the peroxy bridge in EP also forms a hydrogen bonding interaction with LYS-320. All these interactions help to stabilize the conformation and assembly of the ligand in the new site of the GLS1 allosteric pocket. Notably, the binding energy of the protein–ligand complex reached –10.79 kcal/mol, which was a good indication of the good inhibitory activity of compound **3g** toward GLS1.

### 2.9. Compound ***3g*** Inhibited Tumor Growth In Vivo

**Figure 8:**
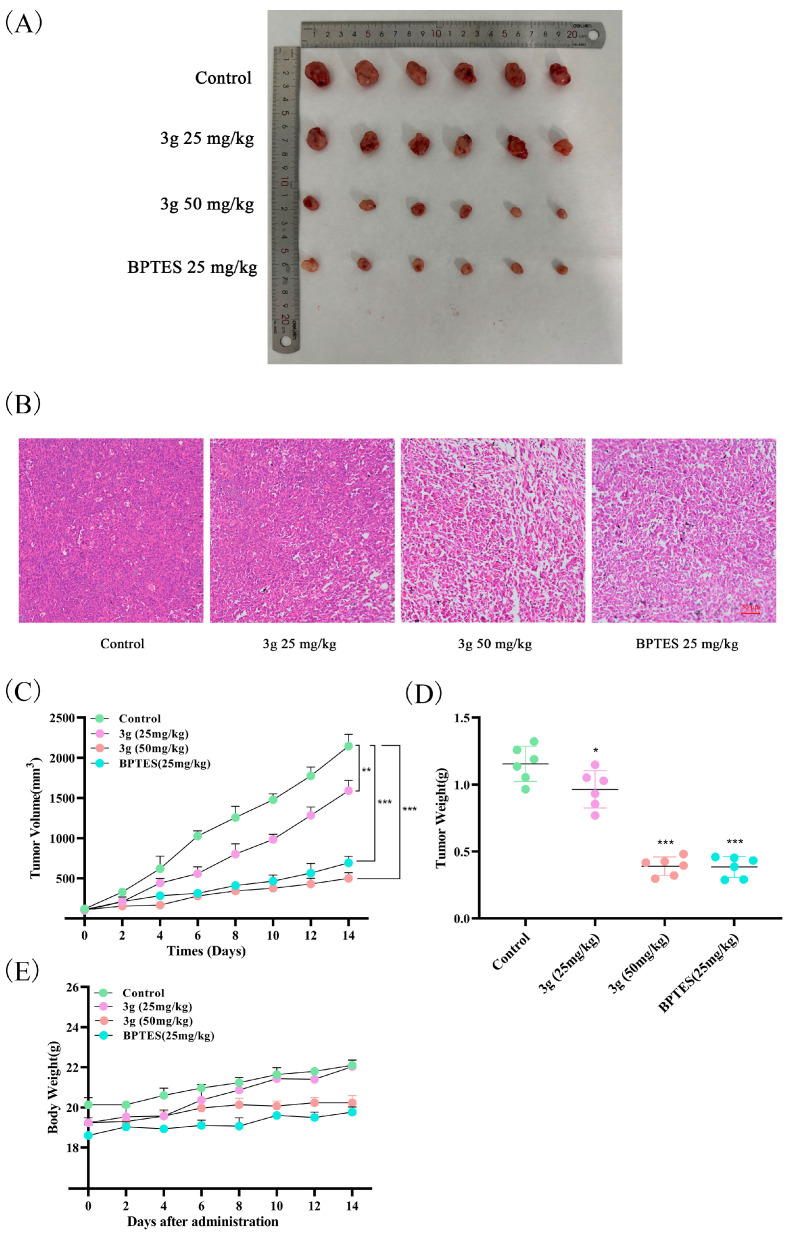
Compound **3g** inhibited the growth of 4T1 cells in vivo. (**A**) Tumor images of 4T1 tumor-bearing mice treated with **3g** or BPTES; and (**B**) tumor HE staining. Scale = 50 μm. (**C**) Changes in the tumor volume; (**D**) tumor weight; and (**E**) body weight of 4T1 tumor-bearing mice. Data represent the mean ± SD (*n* = 6), * *p* < 0.05, ** *p* < 0.01, *** *p* < 0.001, compared with the control group. Scale = 50 μm.

**Figure 9:**
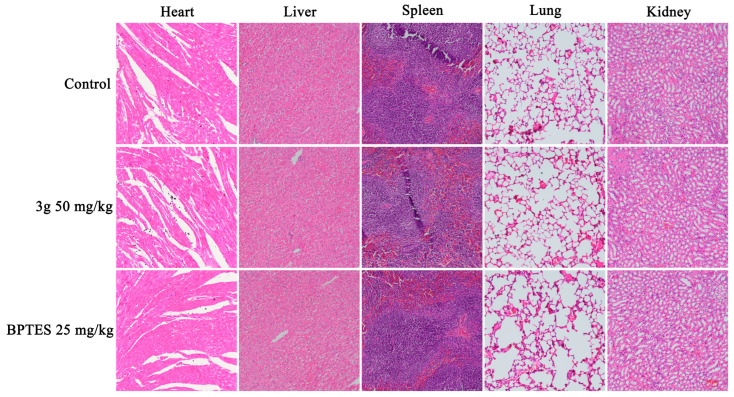
The effect of compound **3g** on organ damage in model mice. The hearts, livers, spleens, lungs, and kidneys of the mice were harvested and sectioned for HE staining. Scale bars = 50 μm.

To more deeply investigate the antitumor activity of compound **3g** in vivo, 4T1 cells were injected subcutaneously into BALB/c mice by us to successfully establish a mouse-transplanted tumor model. The mice were treated with **3g** (25 and 50 mg/kg/2 days) and BPTES (25 mg/kg/2 days) until they were euthanized on the 14th day. Then, the mice’s organs were collected for histopathological analysis. The tumor growth in the **3g** treatment group was effectively inhibited compared with that in the blank control group, as shown in Figure 8A,B. After **3g** treatment, both the tumor volume and the tumor weight were significantly reduced (Figure 8C,D). Additionally, compound **3g** had no significant effect on the body weights of the mice throughout the experimental treatment period, with no obvious signs of adverse reactions (Figure 8E). To explore the impact of **3g** on organ damage, we used the HE staining method to observe the changes in the heart, liver, spleen, lungs, and kidneys. In the tumor-bearing mouse group treated with **3g**, no histopathological changes were detected in these organs, clearly indicating the absence of significant toxicity under the current treatment method (Figure 9). These results fully demonstrate that **3g** can effectively inhibit the proliferation of tumors in model mice without obvious toxicity to other organs.

## 3. Materials and Methods

### 3.1. Chemistry

All the reagents were procured from Hyclone Inc (Logan, UT, USA) and Aladdin (Shanghai, China), and they were used without prior purification. The separation and purification of all the reactions were conducted using silica gel column chromatography with a particle size of 300–400 mesh, and the progress was monitored by TLC plates coated with silica gel GF254. The 1H and 13C nuclear magnetic resonance (NMR) spectra were recorded on an Avance DRX400 spectrometer from Bruker (Beijing, China), operating at 600 MHz and 150 MHz, respectively. The reported chemical shift values are presented in terms of the chemical shift (δ) in parts per million (ppm). Mass spectrometry was performed on an Esquire 6000 mass spectrometer from Skyray Instrument Co., Ltd. (Jiangsu, China).

#### 3.1.1. General Procedure to Prepare Intermediates **1**–**4**

EP (500 mg, 1.1 mmol) was dissolved in anhydrous CH_2_Cl_2_ (10 mL), followed by the sequential addition of Et_3_N (0.6 mmol) and a mixture of SA, MA, GA, and PA (A, B, C, D, 3.5 mmol, 3 eq.). The reaction mixture was refluxed under nitrogen at room temperature for 24 h. Upon completion of the reaction, as monitored by thin-layer chromatography (TLC), the mixture was diluted with CH_2_Cl_2_ (50 mL), extracted with water, and washed with saturated sodium chloride solution. The organic layer was dried with anhydrous sodium sulfate, and the solvent was removed under reduced pressure. The resulting product was purified by silica gel column chromatography using a solvent system of n-hexane and ethyl acetate (50:1, *v*/*v*), yielding the desired pure compounds **1**–**4** (see Supplementary Materials).

Ergosterol peroxide-3-(4-oxobut-2-enoic acid) (**1**)

White solid (85%). ^1^H NMR (600 MHz, CDCl_3_) *δ* 6.54 (1H, d, *J* = 8.5 Hz, H-7), 6.45 (1H, d, *J* = 12.8 Hz, C=C), 6.33 (1H, d, *J* = 12.8 Hz, C=C), 6.24 (1H, d, *J* = 8.5 Hz, H-6), 5.24–5.20 (1H, m, H-22), 5.15 (2H, dt, *J* = 15.2, 7.7 Hz, H-23,H-3), 2.22 (1H, dd, *J* = 14.4, 6.1 Hz), 2.13–2.02 (4H, m), 1.98–1.95 (1H, m), 1.85 (1H, d, *J* = 6.4 Hz), 1.76 (1H, d, *J* = 9.8 Hz), 1.68 (1H, d, *J* = 14.9 Hz), 1.61–1.55 (2H, m), 1.53 (2H, d, *J* = 6.8 Hz), 1.47 (1H, q, *J* = 6.6 Hz), 1.38 (1H, d, *J* = 8.6 Hz), 1.24 (4H, dd, *J* = 11.0, 7.8 Hz), 1.00 (3H, d, *J* = 6.6 Hz, H-18), 0.91 (6H, d, *J* = 7.6 Hz, H-28, H-21), 0.82 (9H, t, *J* = 5.0 Hz, H-26, H-27, H-19); ^13^C NMR (150 MHz, CDCl_3_) *δ* 167.2, 164.2, 137.0, 135.2, 134.7, 132.4, 131.2, 129.5, 81.6, 79.6, 73.2, 56.2, 51.6, 51.0, 44.6, 42.8, 39.7, 39.3, 36.9, 34.2, 33.1, 32.8, 28.6, 25.9, 23.4, 20.9, 20.6, 20.0, 19.7, 18.1, 17.6, 12.9.

Ergosterol peroxide-3-(4-oxobutanoic acid) (**2**)

White solid (81%). ^1^H NMR (600 MHz, CDCl_3_) *δ* 6.51 (d, *J* = 8.5 Hz, 1H, H-7), 6.23 (d, *J* = 8.5 Hz, 1H, H-6), 5.22 (dd, *J* = 15.2, 7.7 Hz, 1H), 5.14 (dd, *J* = 15.2, 8.5 Hz, 1H), 5.01 (td, *J* = 11.5, 5.6 Hz, 1H), 2.68–2.65 (m, 2H, H-2′), 2.58 (t, *J* = 6.3 Hz, 2H, H-3′), 2.14–2.11 (m, 1H), 2.06–1.97 (m, 4H), 1.96–1.94 (m, 1H), 1.86–1.83 (m, 1H), 1.78–1.73 (m, 1H), 1.71–1.68 (m, 1H), 1.57 (d, *J* = 11.1 Hz, 2H), 1.51 (d, *J* = 9.6 Hz, 2H), 1.48–1.45 (m, 1H), 1.42 (q, *J* = 6.1, 5.3 Hz, 1H), 1.37 (d, *J* = 9.9 Hz, 1H), 1.26–1.22 (m, 4H), 1.00 (d, *J* = 6.6 Hz, 3H, H-18), 0.92–0.89 (m, 6H, H-28, H-21), 0.82 (dd, *J* = 6.9, 2.9 Hz, 9H, H-26, H-27, H-19); ^13^C NMR (150 MHz, CDCl_3_) *δ* 178.07, 171.20, 135.22, 135.08, 132.32, 130.91, 81.76, 79.42, 70.05, 56.17, 51.61, 51.00, 44.56, 42.78, 39.75, 39.30, 36.95, 34.24, 33.07, 29.72, 29.14, 28.98, 28.65, 26.17, 23.37, 20.89, 20.62, 19.96, 19.65, 18.08, 17.59, 12.89.

Ergosterol peroxide-3-(5-oxopentanoic acid) (**3**)

White solid (82%). ^1^H NMR (600 MHz, CDCl_3_) *δ* 6.51 (d, *J* = 8.5 Hz, 1H, H-7), 6.23 (d, *J* = 8.5 Hz, 1H, H-6), 5.22 (dd, *J* = 15.2, 7.6 Hz, 1H), 5.14 (dd, *J* = 15.3, 8.4 Hz, 1H), 5.00 (dt, *J* = 11.7, 6.1 Hz, 1H), 2.42 (d, *J* = 7.3 Hz, 2H, H-4′), 2.36 (t, *J* = 7.3 Hz, 2H, H-3′), 2.14–2.11 (m, 1H), 2.01 (t, *J* = 12.8 Hz, 4H), 1.95 (d, *J* = 7.2 Hz, 3H, H-2′), 1.85 (d, *J* = 6.8 Hz, 1H), 1.76–1.73 (m, 1H), 1.70 (d, *J* = 13.6 Hz, 1H), 1.57 (d, *J* = 11.5 Hz, 2H), 1.51 (d, *J* = 11.9 Hz, 2H), 1.47–1.45 (m, 1H), 1.39 (d, *J* = 5.2 Hz, 1H), 1.36 (s, 1H), 1.25 (s, 4H), 1.00 (d, *J* = 6.6 Hz, 3H), 0.91–0.89 (m, 6H), 0.84–0.81 (m, 9H); ^13^C NMR (150 MHz, CDCl_3_) *δ* 177.82, 171.92, 135.22, 135.08, 132.33, 130.94, 81.76, 79.42, 69.63, 56.18, 51.61, 51.01, 44.57, 42.78, 39.75, 36.96, 34.27, 33.44, 33.17, 33.07, 29.72, 28.65, 26.30, 23.38, 20.89, 20.63, 19.96, 19.65, 18.09, 17.58, 12.89.

Ergosterol peroxide-3-(2-carbonyl-benzoic acid) (**4**)

White solid (76%). ^1^H NMR (600 MHz, CDCl_3_) *δ* 7.80 (s, 1H), 7.64 (s, 1H), 7.48 (s, 2H), 6.44 (s, 1H), 6.17 (s, 1H), 5.22 (d, *J* = 7.7 Hz, 1H), 5.15 (dd, *J* = 15.1, 8.2 Hz, 2H), 2.23 (s, 1H), 2.02 (d, *J* = 9.2 Hz, 3H), 1.94 (d, *J* = 11.0 Hz, 2H), 1.85 (d, *J* = 6.7 Hz, 1H), 1.75 (d, *J* = 10.0 Hz, 2H), 1.58 (s, 2H), 1.49–1.45 (m, 3H), 1.38 (s, 1H), 1.34 (d, *J* = 2.9 Hz, 1H), 1.25 (s, 4H), 1.00 (d, *J* = 6.5 Hz, 3H, C-18), 0.92 (d, *J* = 6.8 Hz, 3H, C-28), 0.84–0.80 (m, 12H, C-21, C-26, C-27, C-19); ^13^C NMR (150 MHz, CDCl_3_) *δ* 167.08, 166.72, 134.21, 132.74, 131.29, 129.91, 129.59, 128.96, 128.76, 128.73, 127.83, 127.33, 80.89, 78.32, 64.56, 55.16, 50.61, 49.99, 43.52, 41.78, 38.73, 38.31, 35.90, 33.26, 32.04, 30.91, 27.63, 26.21, 22.31, 19.88, 19.63, 18.94, 18.64, 16.97, 16.60, 11.85.

#### 3.1.2. General Procedure to Prepare Compounds **1a**–**h**, **2a**–**h**, **3a**–**h**, and **4a**–**h**

Intermediates **1**, **2**, **3**, and **4** (100 mg, 0.2 mmol) were dissolved in 10 mL of DMF, and EDCI (0.2 mmol, 0.5 eq.), HOBt (0.1 mmol), and pyridine (py, 0.1 mmol) were added sequentially. The mixture was stirred at room temperature for 30 min, followed by the addition of an amine substituent (0.3 mmol, 1.2 eq.). The reaction was continued under nitrogen protection at room temperature with stirring for 12 to 48 h. After the completion of the reaction was confirmed by thin-layer chromatography (TLC), the mixture was extracted with water and ethyl acetate, and the aqueous layer was dewatered using saturated sodium chloride. The organic layer was dried with anhydrous sodium sulfate and the solvent was concentrated under reduced pressure. The target compounds **1a–h**, **2a–h**, **3a–h**, and **4a–h** were obtained through purification by silica gel column chromatography using a dichloromethane-methanol solvent system at a ratio of 100:1 (*v*/*v*).

Ergosterol peroxide-3-(4-((1,3,4-thiadiazol-2-yl)amino)-4-oxobut-2-enoate) (**1a**)

White solid (86%). ^1^H NMR (600 MHz, CDCl_3_) *δ* 8.78 (s, 1H, S-CH), 6.63 (d, *J* = 12.4 Hz, 1H, H-3′), 6.43 (d, *J* = 8.5 Hz, 1H, H-7), 6.36 (d, *J* = 12.4 Hz, 1H, H-2′), 6.12 (d, *J* = 8.5 Hz, 1H, H-6), 5.15–5.13 (m, 1H), 5.09–5.04 (m, 2H), 2.11–2.08 (m, 1H), 1.95 (d, *J* = 13.8 Hz, 4H), 1.89–1.87 (m, 1H), 1.78 (d, *J* = 6.7 Hz, 1H), 1.68 (d, *J* = 7.4 Hz, 1H), 1.64–1.61 (m, 1H), 1.51 (s, 2H), 1.45–1.42 (m, 2H), 1.40 (d, *J* = 6.5 Hz, 1H), 1.34–1.32 (m, 1H), 1.29 (s, 1H), 1.18 (s, 4H), 0.93 (d, *J* = 6.5 Hz, 3H, H-18), 0.84 (d, *J* = 6.8 Hz, 3H, H-28), 0.78 (s, 3H, H-21), 0.76–0.74 (m, 9H, H-26, H-27, H-19); ^13^C NMR (150 MHz, CDCl_3_) *δ* 163.88, 161.15, 158.59, 146.81, 134.16, 133.85, 131.30, 131.00, 130.15, 130.02, 80.60, 78.39, 70.52, 55.13, 50.52, 49.90, 43.52, 41.75, 38.71, 38.24, 35.90, 33.13, 32.04, 31.81, 27.61, 24.88, 22.33, 19.86, 19.59, 18.94, 18.62, 16.99, 16.56, 11.86; HRMS (ESI) *m*/*z*: calcd for C_34_H_47_N_3_O_5_S [M + Na]^+^: 632.3236, found: 632.3125.

Ergosterol peroxide-3-(4-(thiazol-2-ylamino)-4-oxobut-2-enoate) (**1b**)

White solid (81%). ^1^H NMR (600 MHz, CDCl_3_) *δ* 7.48 (s, 1H, N-CH), 7.01 (d, *J* = 3.5 Hz, 1H, S-CH), 6.55 (d, *J* = 12.8 Hz, 1H, H-3′), 6.50 (d, *J* = 8.5 Hz, 1H, H-7), 6.30 (d, *J* = 12.8 Hz, 1H, H-2′), 6.17 (d, *J* = 8.5 Hz, 1H, H-6), 5.22 (dd, *J* = 15.2, 7.6 Hz, 1H), 5.14 (dd, *J* = 15.1, 8.4 Hz, 2H), 2.13 (dd, *J* = 13.6, 5.3 Hz, 1H), 2.00 (d, *J* = 9.1 Hz, 4H), 1.94 (s, 1H), 1.86–1.83 (m, 1H), 1.74 (d, *J* = 9.1 Hz, 1H), 1.69 (d, *J* = 10.5 Hz, 1H), 1.59–1.55 (m, 2H), 1.49 (t, *J* = 5.3 Hz, 2H), 1.45 (d, *J* = 6.5 Hz, 1H), 1.40–1.37 (m, 1H), 1.35 (d, *J* = 7.3 Hz, 1H), 1.25 (s, 4H), 1.00 (d, *J* = 6.6 Hz, 3H, H-18), 0.91 (d, *J* = 6.8 Hz, 3H, H-28), 0.84–0.80 (m, 12H, H-21, H-26, H-27, H-19); ^13^C NMR (150 MHz, CDCl_3_) *δ* 164.90, 161.69, 158.40, 137.60, 135.73, 135.20, 134.82, 132.34, 131.07, 128.20, 113.94, 81.58, 79.43, 71.83, 56.17, 51.54, 50.91, 44.55, 42.79, 39.75, 39.26, 36.92, 34.11, 33.07, 32.80, 28.64, 25.90, 23.36, 20.89, 20.62, 19.97, 19.65, 17.97, 17.59, 12.88; HRMS (ESI) *m/z:* calcd for C_35_H_48_N_2_O_5_S [M + Na]^+^: 631.3284, found: 631.3183.

Ergosterol peroxide-3-(4-((5-fluorobenzo[d]thiazol-2-yl)amino)-4-oxobut-2-enoate) (**1c**)

Yellow solid (79%). ^1^H NMR (600 MHz, CDCl_3_) *δ* 7.73 (dd, *J* = 8.7, 5.1 Hz, 1H, H-Ar), 7.53 (d, *J* = 9.5 Hz, 1H, H-Ar), 7.08 (t, *J* = 7.6 Hz, 1H, H-Ar), 6.52 (d, *J* = 13.1 Hz, 2H, H-3′, H-7), 6.36 (d, *J* = 13.2 Hz, 1H, H-2′), 6.21 (d, *J* = 8.4 Hz, 1H, H-6), 5.22 (dd, *J* = 15.1, 7.8 Hz, 2H), 5.17–5.13 (m, 1H), 2.21 (dd, *J* = 13.6, 5.0 Hz, 1H), 2.10–2.01 (m, 4H), 1.96 (d, *J* = 10.0 Hz, 1H), 1.85 (d, *J* = 6.8 Hz, 1H), 1.75 (s, 1H), 1.62 (s, 1H), 1.61–1.56 (m, 2H), 1.51 (d, *J* = 11.9 Hz, 2H), 1.48–1.45 (m, 1H), 1.41–1.38 (m, 1H), 1.35 (d, *J* = 8.4 Hz, 1H), 1.25 (s, 4H), 1.00 (d, *J* = 6.6 Hz, 3H, H-18), 0.91 (d, *J* = 6.7 Hz, 3H, H-28), 0.88 (s, 3H, H-21), 0.82 (d, *J* = 10.1 Hz, 9H, H-26, H-27, H-19); ^13^C NMR (150 MHz, CDCl_3_) *δ* 165.54, 162.84, 161.70, 159.63, 136.46, 135.19, 134.73, 132.36, 131.17, 128.81, 127.62, 122.09, 112.63, 107.80, 107.64, 81.60, 79.47, 72.48, 56.18, 51.54, 50.92, 44.57, 42.80, 39.75, 39.27, 36.95, 34.16, 33.08, 32.86, 28.65, 26.02, 23.38, 20.90, 20.63, 19.98, 19.65, 18.01, 17.60, 12.90; HRMS (ESI) *m*/*z*: calcd for C_39_H_49_FN_2_O_5_S [M + Na]^+^: 699.3346, found: 699.3239.

Ergosterol peroxide-3-(4-((6-fluorobenzo[d]thiazol-2-yl)amino)-4-oxobut-2-enoate) (**1d**)

Yellow solid (77%). ^1^H NMR (600 MHz, CDCl_3_) *δ* 7.79 (dd, *J* = 8.9, 4.7 Hz, 1H, H-Ar), 7.50 (dd, *J* = 8.0, 2.6 Hz, 1H, H-Ar), 7.17 (td, *J* = 8.9, 2.6 Hz, 1H, H-Ar), 6.54–6.50 (m, 2H, H-3′, H-7), 6.35 (d, *J* = 13.2 Hz, 1H, H-2′), 6.20 (d, *J* = 8.5 Hz, 1H, H-6), 5.22 (dd, *J* = 15.2, 7.6 Hz, 2H), 5.15 (dd, *J* = 15.3, 8.4 Hz, 1H), 2.23–2.19 (m, 1H), 2.09–2.01 (m, 4H), 1.96 (d, *J* = 10.5 Hz, 1H), 1.85 (d, *J* = 6.7 Hz, 1H), 1.74 (d, *J* = 3.7 Hz, 1H), 1.64 (d, *J* = 8.9 Hz, 1H), 1.60–1.55 (m, 2H), 1.50 (d, *J* = 11.4 Hz, 2H), 1.48–1.45 (m, 1H), 1.40–1.38 (m, 1H), 1.36 (d, *J* = 10.4 Hz, 1H), 1.25 (s, 4H), 1.00 (d, *J* = 6.7 Hz, 3H, H-18), 0.91 (d, *J* = 6.8 Hz, 3H, H-28), 0.88 (s, 3H, H-21), 0.84–0.81 (m, 9H, H-26, H-27, H-19); ^13^C NMR (150 MHz, CDCl_3_) *δ* 165.57, 161.70, 158.91, 157.18, 145.20, 136.61, 135.19, 134.73, 133.27, 132.36, 131.16, 128.70, 122.25, 114.63, 107.66, 81.61, 79.47, 72.45, 56.18, 51.54, 50.92, 44.57, 42.79, 39.75, 39.27, 36.95, 34.16, 33.07, 32.86, 28.65, 26.01, 23.38, 20.90, 20.63, 19.98, 19.65, 18.01, 17.60, 12.89; HRMS (ESI) *m*/*z*: calcd for C_39_H_49_FN_2_O_5_S [M + Na]^+^: 699.3346, found: 699.3235.

Ergosterol peroxide-3-(4-oxo-4-(phenylamino)but-2-enoate) (**1e**)

White solid (77%). ^1^H NMR (600 MHz, CDCl_3_) *δ* 10.92 (s, 1H, H-N), 7.66 (d, *J* = 7.7 Hz, 2H, H-Ar), 7.34 (d, *J* = 7.7 Hz, 2H, H-Ar), 7.12 (d, *J* = 7.2 Hz, 1H, H-Ar), 6.52 (d, *J* = 8.5 Hz, 1H, H-7), 6.42 (d, *J* = 13.2 Hz, 1H, H-3′), 6.21 (d, *J* = 8.3 Hz, 1H, H-6), 6.15 (d, *J* = 13.3 Hz, 1H, H-2′), 5.22 (dd, *J* = 15.2, 7.6 Hz, 1H), 5.17–5.11 (m, 2H), 2.22 (dd, *J* = 13.6, 5.3 Hz, 1H), 2.06–2.00 (m, 4H), 1.97–1.95 (m, 1H), 1.85 (d, *J* = 6.6 Hz, 1H), 1.74 (d, *J* = 3.7 Hz, 1H), 1.64 (d, *J* = 3.9 Hz, 1H), 1.60–1.56 (m, 2H), 1.53–1.49 (m, 2H), 1.48–1.45 (m, 1H), 1.41–1.38 (m, 1H), 1.36 (d, *J* = 10.6 Hz, 1H), 1.25 (s, 4H), 1.00 (d, *J* = 6.6 Hz, 3H, H-18), 0.91 (d, *J* = 6.8 Hz, 3H, H-28), 0.88 (s, 3H, H-21), 0.84–0.81 (m, 9H, H-26, H-27, H-19); ^13^C NMR (150 MHz, CDCl_3_) *δ* 165.96, 161.68, 140.41, 137.91, 135.18, 134.83, 132.36, 131.09, 129.02, 125.45, 124.64, 124.56, 120.14, 120.02, 81.72, 79.53, 71.68, 56.20, 51.59, 51.03, 44.58, 42.79, 39.74, 39.28, 36.96, 34.27, 33.07, 32.91, 28.65, 26.06, 23.39, 20.89, 20.61, 19.98, 19.66, 18.03, 17.60, 12.90; HRMS (ESI) *m*/*z*: calcd for C_38_H_51_NO_5_ [M + Na]^+^: 624.3767, found: 624.3665.

Ergosterol peroxide-3-(4-((4-fluorophenyl)amino)-4-oxobut-2-enoate) (**1f**)

White solid (80%). ^1^H NMR (600 MHz, CDCl_3_) *δ* 11.10 (s, 1H, H-N), 7.63 (dd, *J* = 8.9, 4.8 Hz, 2H, H-Ar), 7.02 (t, *J* = 8.6 Hz, 2H, H-Ar), 6.53 (d, *J* = 8.5 Hz, 1H, H-7), 6.41 (d, *J* = 13.5 Hz, 1H, H-3′), 6.22 (d, *J* = 8.5 Hz, 1H, H-6), 6.16 (d, *J* = 13.4 Hz, 1H, H-2′), 5.22 (dd, *J* = 15.3, 7.7 Hz, 1H), 5.17–5.11 (m, 2H), 2.23 (dd, *J* = 13.6, 7.1 Hz, 1H), 2.08–1.99 (m, 4H), 1.95 (s, 1H), 1.85 (d, *J* = 6.6 Hz, 1H), 1.77–1.75 (m, 1H), 1.66 (d, *J* = 13.9 Hz, 1H), 1.61–1.55 (m, 2H), 1.52 (t, *J* = 5.4 Hz, 2H), 1.49–1.45 (m, 1H), 1.41–1.38 (m, 1H), 1.36 (d, *J* = 12.7 Hz, 1H), 1.25 (d, *J* = 9.7 Hz, 4H), 1.00 (d, *J* = 6.6 Hz, 3H, H-18), 0.92–0.89 (m, 6H, H-28, H-21), 0.84–0.81 (m, 9H, H-26, H-27, H-19); ^13^C NMR (150 MHz, CDCl_3_) *δ* 166.11, 161.47, 160.30, 158.69, 140.57, 135.17, 134.77, 134.00, 132.37, 131.15, 125.53, 121.68, 121.63, 115.72, 115.57, 81.72, 79.56, 71.81, 56.20, 51.59, 51.03, 44.59, 42.79, 39.73, 39.27, 36.97, 34.27, 33.07, 32.91, 28.65, 26.07, 23.39, 20.89, 20.61, 19.97, 19.65, 18.04, 17.59, 12.89; HRMS (ESI) *m*/*z*: calcd for C_38_H_50_FNO_5_ [M + Na]^+^: 642.3673, found: 642.3572.

Ergosterol peroxide-3-(4-((4-methoxyphenyl)amino)-4-oxobut-2-enoate) (**1g**)

White solid (85%). ^1^H NMR (600 MHz, CDCl_3_) *δ* 10.90 (s, 1H, H-N), 7.58 (d, *J* = 9.0 Hz, 2H, H-Ar), 6.87 (d, *J* = 8.9 Hz, 2H, H-Ar), 6.52 (d, *J* = 8.5 Hz, 1H, H-7), 6.42 (d, *J* = 13.4 Hz, 1H, H-3′), 6.22 (d, *J* = 8.5 Hz, 1H, H-6), 6.14 (d, *J* = 13.4 Hz, 1H, H-2′), 5.24–5.20 (m, 1H), 5.16–5.11 (m, 2H), 3.80 (s, 3H, H-OCH_3_), 2.22 (d, *J* = 9.4 Hz, 1H), 2.07–2.01 (m, 4H), 1.95 (s, 1H), 1.85 (d, *J* = 6.5 Hz, 1H), 1.75 (d, *J* = 6.7 Hz, 1H), 1.65 (s, 1H), 1.58 (d, *J* = 10.9 Hz, 2H), 1.52 (t, *J* = 5.3 Hz, 2H), 1.48–1.45 (m, 1H), 1.42 (d, *J* = 6.9 Hz, 1H), 1.36 (d, *J* = 10.5 Hz, 1H), 1.25 (s, 4H), 1.00 (d, *J* = 6.6 Hz, 3H, H-18), 0.90 (d, *J* = 5.3 Hz, 6H, H-28, H-21), 0.84–0.81 (m, 9H, H-26, H-27, H-19); ^13^C NMR (150 MHz, CDCl_3_) *δ* 166.06, 161.30, 156.54, 140.78, 135.18, 134.82, 132.37, 131.11, 125.12, 121.56, 114.17, 81.73, 79.54, 71.64, 56.20, 55.49, 51.59, 51.02, 44.59, 42.78, 39.74, 39.28, 36.97, 34.27, 33.07, 32.92, 28.65, 26.07, 23.39, 20.88, 20.61, 19.97, 19.65, 18.04, 17.59, 12.90; HRMS (ESI) *m*/*z*: calcd for C_39_H_53_NO_6_ [M + Na]^+^: 654.3873, found: 654.3770.

Ergosterol peroxide-3-(4-(naphthalen-2-ylamino)-4-oxobut-2-enoate) (**1h**)

White solid (87%). ^1^H NMR (600 MHz, CDCl_3_) *δ* 11.33 (s, 1H, H-N), 8.40 (s, 1H, H-Ar), 7.84–7.77 (m, 3H, H-Ar), 7.58 (dd, *J* = 8.8, 2.2 Hz, 1H, H-Ar), 7.46 (t, *J* = 7.7 Hz, 1H, H-Ar), 7.40 (t, *J* = 7.5 Hz, 1H, H-Ar), 6.52 (d, *J* = 8.5 Hz, 1H, H-7), 6.47 (d, *J* = 13.4 Hz, 1H, H-3′), 6.22–6.18 (m, 2H, H-6, H-2′), 5.24–5.20 (m, 1H), 5.16 (td, *J* = 15.3, 14.4, 6.9 Hz, 2H), 2.27–2.24 (m, 1H), 2.08–2.00 (m, 4H), 1.96 (d, *J* = 9.5 Hz, 1H), 1.85 (d, *J* = 6.7 Hz, 1H), 1.77–1.74 (m, 1H), 1.68 (d, *J* = 8.7 Hz, 1H), 1.59 (t, *J* = 9.6 Hz, 2H), 1.54–1.50 (m, 2H), 1.48–1.45 (m, 1H), 1.41 (dd, *J* = 11.4, 5.3 Hz, 1H), 1.36 (d, *J* = 10.5 Hz, 1H), 1.25 (s, 4H), 1.00 (d, *J* = 6.6 Hz, 3H, H-18), 0.91 (d, *J* = 6.9 Hz, 3H, H-28), 0.89 (s, 3H, H-21), 0.84–0.81 (m, 9H, H-26, H-27, H-19); ^13^C NMR (150 MHz, CDCl_3_) *δ* 166.17, 161.69, 140.93, 135.39, 135.18, 134.80, 133.93, 132.37, 131.13, 130.86, 128.78, 127.89, 127.57, 126.42, 125.42, 125.06, 120.01, 116.84, 81.74, 79.55, 71.83, 56.20, 51.60, 51.05, 44.59, 42.79, 39.74, 39.28, 36.97, 34.30, 33.08, 32.93, 28.66, 26.10, 23.40, 20.89, 20.61, 19.98, 19.66, 18.03, 17.59, 12.91; HRMS (ESI) *m*/*z*: calcd for C_42_H_53_NO_5_ [M + Na]^+^: 674.3924, found: 674.3822.

Ergosterol peroxide-3-(4-((1,3,4-thiadiazol-2-yl)amino)-4-oxobutanoate) (**2a**)

White solid (83%). ^1^H NMR (600 MHz, CDCl_3_) *δ* 13.38 (s, 1H, H-N), 8.80 (s, 1H, S-CH), 6.49 (d, *J* = 8.5 Hz, 1H, H-7), 6.18 (d, *J* = 8.5 Hz, 1H, H-6), 5.21 (dd, *J* = 15.2, 7.7 Hz, 1H), 5.16–5.12 (m, 1H), 5.00 (dt, *J* = 11.6, 6.1 Hz, 1H), 3.07 (q, *J* = 6.3 Hz, 2H, H-3′), 2.79 (t, *J* = 6.5 Hz, 2H, H-2′), 2.09 (dd, *J* = 12.9, 6.1 Hz, 1H), 2.03–1.95 (m, 4H), 1.94 (s, 1H), 1.85 (d, *J* = 6.7 Hz, 1H), 1.76–1.72 (m, 1H), 1.68–1.65 (m, 1H), 1.57–1.53 (m, 2H), 1.48 (d, *J* = 7.3 Hz, 2H), 1.45 (d, *J* = 6.5 Hz, 1H), 1.40 (t, *J* = 6.1 Hz, 1H), 1.37–1.33 (m, 1H), 1.27–1.18 (m, 4H), 0.99 (d, *J* = 6.5 Hz, 3H, H-18), 0.90 (d, *J* = 6.8 Hz, 3H, H-28), 0.85–0.80 (m, 12H, H-21, H-26, H-27, H-19); ^13^C NMR (150 MHz, CDCl_3_) *δ* 171.18, 170.69, 160.45, 147.42, 135.22, 135.03, 132.31, 130.93, 81.70, 79.39, 70.07, 56.17, 51.59, 50.97, 44.55, 42.78, 39.74, 39.29, 36.92, 34.21, 33.07, 30.87, 29.16, 28.64, 26.17, 23.35, 20.90, 20.62, 19.96, 19.65, 18.01, 17.58, 12.88; HRMS (ESI) *m*/*z*: calcd for C_34_H_49_N_3_O_5_S [M + Na]^+^: 634.3393, found: 634.3295.

Ergosterol peroxide-3-(4-oxo-4-(thiazol-2-ylamino)butanoate) (**2b**)

White solid (80%). ^1^H NMR (600 MHz, CDCl_3_) *δ* 7.50 (d, *J* = 3.6 Hz, 1H, N-CH), 6.99 (d, *J* = 3.6 Hz, 1H, S-CH), 6.50 (d, *J* = 8.5 Hz, 1H, H-7), 6.20 (d, *J* = 8.5 Hz, 1H, H-6), 5.22 (dd, *J* = 15.3, 7.6 Hz, 1H), 5.14 (dd, *J* = 15.3, 8.4 Hz, 1H), 5.02 (tt, *J* = 11.2, 5.1 Hz, 1H), 2.88 (q, *J* = 6.6, 6.2 Hz, 2H, H-3′), 2.80–2.74 (m, 2H, H-2′), 2.11 (dd, *J* = 14.6, 4.4 Hz, 1H), 2.04–1.95 (m, 4H), 1.94 (s, 1H), 1.85 (q, *J* = 6.8 Hz, 1H), 1.77–1.71 (m, 1H), 1.70–1.66 (m, 1H), 1.58–1.54 (m, 2H), 1.49 (d, *J* = 9.4 Hz, 2H), 1.47–1.43 (m, 1H), 1.42–1.37 (m, 1H), 1.36–1.31 (m, 1H), 1.26–1.19 (m, 4H), 0.99 (d, *J* = 6.5 Hz, 3H, H-18), 0.91 (d, *J* = 6.8 Hz, 3H, H-28), 0.86 (s, 3H, H-21), 0.84–0.80 (m, 9H, H-26, H-27, H-19); ^13^C NMR (150 MHz, CDCl_3_) *δ* 171.36, 169.73, 159.99, 136.25, 135.22, 135.04, 132.32, 130.93, 113.54, 81.72, 79.40, 70.16, 56.17, 51.59, 50.99, 44.56, 42.78, 39.75, 39.30, 36.94, 34.23, 33.07, 30.83, 29.72, 29.14, 28.65, 26.20, 23.37, 20.90, 20.62, 19.97, 19.65, 18.03, 17.59, 12.88; HRMS (ESI) *m*/*z*: calcd for C_35_H_50_N_2_O_5_S [M + H]^+^: 633.3440, found: 633.3346.

Ergosterol peroxide-3-(4-((5-fluorobenzo[d]thiazol-2-yl)amino)-4-oxobutanoate) (**2c**)

Yellow solid (85%). ^1^H NMR (600 MHz, CDCl_3_) *δ* 7.73 (dd, *J* = 8.7, 5.1 Hz, 1H, H-Ar), 7.47 (d, *J* = 9.6 Hz, 1H, H-Ar), 7.07 (td, *J* = 8.8, 2.5 Hz, 1H, H-Ar), 6.50 (d, *J* = 8.5 Hz, 1H, H-7), 6.20 (d, *J* = 8.5 Hz, 1H, H-6), 5.22 (dd, *J* = 15.2, 7.7 Hz, 1H), 5.14 (dd, *J* = 15.3, 8.5 Hz, 1H), 5.03 (td, *J* = 11.4, 5.6 Hz, 1H), 2.82–2.76 (m, 4H, H-3′, H-2′), 2.16–2.12 (m, 1H), 2.00 (q, *J* = 13.0, 12.5 Hz, 4H), 1.94 (s, 1H), 1.85 (d, *J* = 6.8 Hz, 1H), 1.76–1.72 (m, 1H), 1.70–1.67 (m, 1H), 1.58–1.55 (m, 2H), 1.51–1.48 (m, 2H), 1.47–1.45 (m, 1H), 1.41–1.38 (m, 1H), 1.34 (d, *J* = 11.6 Hz, 1H), 1.26–1.21 (m, 4H), 0.99 (d, *J* = 6.6 Hz, 3H, H-18), 0.91 (d, *J* = 6.7 Hz, 3H, H-28), 0.87 (s, 3H, H-21), 0.84–0.80 (m, 9H, H-26, H-27, H-19); ^13^C NMR (150 MHz, CDCl_3_) *δ* 171.64, 170.30, 162.85, 161.25, 160.45, 135.21, 135.00, 132.33, 130.95, 127.38, 122.23, 112.34, 107.22, 81.74, 79.43, 70.53, 56.17, 51.58, 50.97, 44.56, 42.78, 39.75, 39.28, 36.94, 34.23, 33.07, 31.20, 29.72, 29.15, 28.65, 26.19, 23.37, 20.89, 20.62, 19.96, 19.65, 18.04, 17.58, 12.88; HRMS (ESI) *m*/*z*: calcd for C_39_H_51_FN_2_O_5_S [M + Na]^+^: 701.3503, found: 701.3399.

Ergosterol peroxide-3-(4-((6-fluorobenzo[d]thiazol-2-yl)amino)-4-oxobutanoate) (**2d**)

Yellow solid (78%). ^1^H NMR (600 MHz, CDCl_3_) *δ* 7.65 (dd, *J* = 8.9, 4.6 Hz, 1H, H-Ar), 7.42 (dd, *J* = 8.0, 2.6 Hz, 1H, H-Ar), 7.09 (td, *J* = 8.8, 2.6 Hz, 1H, H-Ar), 6.43 (d, *J* = 8.5 Hz, 1H, H-7), 6.13 (d, *J* = 8.5 Hz, 1H, H-6), 5.15 (dd, *J* = 15.2, 7.7 Hz, 1H), 5.07 (dd, *J* = 15.3, 8.4 Hz, 1H), 4.96 (dt, *J* = 11.6, 6.1 Hz, 1H), 2.74–2.68 (m, 4H, H-3′, H-2′), 2.06 (dd, *J* = 13.9, 5.2 Hz, 1H), 1.95 (d, *J* = 12.8 Hz, 3H), 1.88 (d, *J* = 11.2 Hz, 2H), 1.79–1.76 (m, 1H), 1.67 (d, *J* = 10.7 Hz, 1H), 1.62 (d, *J* = 14.0 Hz, 1H), 1.51–1.49 (m, 2H), 1.42 (t, *J* = 6.6 Hz, 2H), 1.40–1.38 (m, 1H), 1.34–1.31 (m, 1H), 1.28 (d, *J* = 11.4 Hz, 1H), 1.19–1.13 (m, 4H), 0.92 (d, *J* = 6.6 Hz, 3H, H-18), 0.84 (d, *J* = 6.8 Hz, 3H, H-28), 0.80 (s, 3H, H-21), 0.77–0.73 (m, 9H, H-26, H-27, H-19); ^13^C NMR (150 MHz, CDCl_3_) *δ* 170.63, 169.16, 159.43, 157.81, 156.79, 143.56, 134.17, 133.95, 131.30, 129.94, 120.75, 113.73, 106.80, 80.69, 77.99, 69.49, 55.13, 50.97, 49.94, 43.52, 41.74, 38.71, 38.24, 35.90, 33.19, 32.03, 30.15, 28.12, 27.61, 25.17, 22.33, 19.84, 19.58, 18.93, 18.61, 17.00, 16.54, 11.85; HRMS (ESI) *m*/*z*: calcd for C_39_H_51_FN_2_O_5_S [M + Na]^+^: 701.3503, found: 701.3402.

Ergosterol peroxide-3-(4-oxo-4-(phenylamino)-4-oxobutanoate) (**2e**)

White solid (83%). ^1^H NMR (600 MHz, CDCl_3_) *δ* 7.78 (s, 1H, H-N), 7.49 (d, *J* = 7.8 Hz, 2H, H-Ar), 7.30 (t, *J* = 7.8 Hz, 2H, H-Ar), 7.08 (t, *J* = 7.4 Hz, 1H, H-Ar), 6.50 (d, *J* = 8.5 Hz, 1H, H-7), 6.20 (d, *J* = 8.5 Hz, 1H, H-6), 5.22 (dd, *J* = 15.2, 7.6 Hz, 1H), 5.14 (dd, *J* = 15.3, 8.4 Hz, 1H), 5.02 (dq, *J* = 11.3, 5.6, 5.1 Hz, 1H), 2.74–2.67 (m, 2H, H-3′), 2.64 (t, *J* = 7.0 Hz, 2H), H-2′, 2.13 (dd, *J* = 14.6, 4.3 Hz, 1H), 2.05–1.94 (m, 4H), 1.91 (s, 1H), 1.85 (q, *J* = 6.8 Hz, 1H), 1.77–1.72 (m, 1H), 1.69 (d, *J* = 13.7 Hz, 1H), 1.58 (t, *J* = 7.1 Hz, 2H), 1.52–1.48 (m, 2H), 1.48–1.43 (m, 1H), 1.41–1.37 (m, 1H), 1.37–1.31 (m, 1H), 1.26–1.19 (m, 4H), 1.00 (d, *J* = 6.6 Hz, 3H, H-18), 0.91 (d, *J* = 6.9 Hz, 3H, H-28), 0.87 (s, 3H, H-21), 0.84–0.80 (m, 9H, H-26, H-27, H-19); ^13^C NMR (150 MHz, CDCl_3_) *δ* 172.44, 169.93, 137.92, 135.21, 135.06, 132.33, 130.92, 128.99, 124.20, 119.81, 81.78, 79.45, 70.21, 56.18, 51.61, 51.02, 44.57, 42.78, 39.75, 39.29, 36.95, 34.27, 33.11, 33.07, 32.31, 29.90, 28.65, 26.23, 23.37, 20.89, 20.63, 19.97, 19.65, 18.06, 17.59, 12.89; HRMS (ESI) *m*/*z*: calcd for C_38_H_53_NO_5_ [M + Na]^+^: 626.3924, found: 626.3826.

Ergosterol peroxide-3-(4-((4-fluorophenyl)amino)-4-oxobutanoate) (**2f**)

White solid (81%). ^1^H NMR (600 MHz, CDCl_3_) *δ* 7.85 (s, 1H, H-N), 7.45 (dd, *J* = 8.9, 4.8 Hz, 2H, H-Ar), 6.98 (t, *J* = 8.6 Hz, 2H, H-Ar), 6.51 (d, *J* = 8.5 Hz, 1H, H-7), 6.21 (d, *J* = 8.5 Hz, 1H, H-6), 5.22 (dd, *J* = 15.2, 7.6 Hz, 1H), 5.14 (dd, *J* = 15.2, 8.4 Hz, 1H), 5.03 (tt, *J* = 11.2, 5.1 Hz, 1H), 2.71 (q, *J* = 5.8, 5.3 Hz, 2H, H-3′), 2.63 (t, *J* = 6.5 Hz, 2H, H-2′), 2.13 (dd, *J* = 13.7, 5.2 Hz, 1H), 2.04–1.95 (m, 4H), 1.92 (d, *J* = 12.6 Hz, 1H), 1.85 (q, *J* = 6.8 Hz, 1H), 1.78–1.73 (m, 1H), 1.70 (d, *J* = 13.6 Hz, 1H), 1.60–1.56 (m, 2H), 1.50 (t, *J* = 7.6 Hz, 2H), 1.48–1.43 (m, 1H), 1.42–1.38 (m, 1H), 1.37–1.31 (m, 1H), 1.29–1.15 (m, 4H), 1.00 (d, *J* = 6.5 Hz, 3H, H-18), 0.91 (d, *J* = 6.8 Hz, 3H, H-28), 0.88 (s, 3H, H-21), 0.84–0.81 (m, 9H, H-26, H-27, H-19); ^13^C NMR (150 MHz, CDCl_3_) *δ* 172.56, 169.91, 158.50, 135.19, 135.01, 133.95, 132.34, 130.97, 121.65, 121.60, 115.65, 115.50, 81.78, 79.48, 70.27, 56.18, 51.61, 51.03, 44.57, 42.78, 39.74, 39.29, 36.95, 34.27, 33.12, 33.07, 32.11, 29.85, 28.65, 26.23, 23.37, 20.89, 20.62, 19.97, 19.65, 18.06, 17.59, 12.88; HRMS (ESI) *m*/*z*: calcd for C_38_H_52_FNO_5_ [M + Na]^+^: 644.3830, found: 644.3722.

Ergosterol peroxide-3-(4-((4-methoxyphenyl)amino)-4-oxobutanoate) (**2g**)

White solid (87%). ^1^H NMR (600 MHz, CDCl_3_) *δ* 7.68 (s, 1H, H-N), 7.39 (d, *J* = 9.0 Hz, 2H, H-Ar), 6.83 (d, *J* = 8.9 Hz, 2H, H-Ar), 6.50 (d, *J* = 8.5 Hz, 1H, H-7), 6.21 (d, *J* = 8.4 Hz, 1H, H-6), 5.22 (dd, *J* = 15.2, 7.7 Hz, 1H), 5.14 (dd, *J* = 15.3, 8.4 Hz, 1H), 5.02 (tt, *J* = 11.2, 5.1 Hz, 1H), 3.78 (s, 3H, H-OCH_3_), 2.73–2.67 (m, 2H, H-3′), 2.62 (d, *J* = 6.6 Hz, 2H, H-2′), 2.15–2.11 (m, 1H), 2.04–1.94 (m, 4H), 1.91 (d, *J* = 3.2 Hz, 1H), 1.85 (q, *J* = 6.8 Hz, 1H), 1.77–1.72 (m, 1H), 1.71–1.67 (m, 1H), 1.57 (t, *J* = 9.2 Hz, 2H), 1.51–1.48 (m, 2H), 1.46 (dd, *J* = 13.2, 6.6 Hz, 1H), 1.41–1.37 (m, 1H), 1.37–1.31 (m, 1H), 1.29–1.14 (m, 4H), 1.00 (d, *J* = 6.6 Hz, 3H, H-18), 0.91 (d, *J* = 6.8 Hz, 3H, H-28), 0.88 (s, 3H, H-21), 0.84–0.80 (m, 9H, H-26, H-27, H-19); ^13^C NMR (150 MHz, CDCl_3_) *δ* 172.44, 169.73, 156.34, 135.21, 135.07, 132.33, 131.06, 130.91, 121.71, 114.12, 81.79, 79.44, 70.14, 56.18, 55.49, 51.62, 51.03, 44.57, 42.78, 39.75, 39.30, 36.95, 34.27, 33.12, 33.07, 32.07, 29.94, 28.65, 26.23, 23.37, 20.89, 20.63, 19.97, 19.65, 18.06, 17.59, 12.88; HRMS (ESI) *m*/*z*: calcd for C_39_H_55_NO_6_ [M + Na]^+^: 656.4029, found: 656.3922.

Ergosterol peroxide-3-(4-(naphthalen-2-ylamino)-4-oxobutanoate) (**2h**)

White solid (84%). ^1^H NMR (600 MHz, CDCl_3_) *δ* 8.19 (s, 1H, H-N), 7.96 (s, 1H, H-Ar), 7.76 (t, *J* = 7.1 Hz, 3H, H-Ar), 7.43 (t, *J* = 7.7 Hz, 2H, H-Ar), 7.38 (t, *J* = 7.4 Hz, 1H, H-Ar), 6.49 (d, *J* = 8.5 Hz, 1H, H-7), 6.19 (d, *J* = 8.5 Hz, 1H, H-6), 5.22 (dd, *J* = 15.3, 7.6 Hz, 1H), 5.14 (dd, *J* = 15.3, 8.4 Hz, 1H), 5.04 (dq, *J* = 11.3, 5.7, 5.2 Hz, 1H), 2.75 (q, *J* = 6.1, 5.4 Hz, 2H, H-3′), 2.70 (d, *J* = 7.0 Hz, 2H, H-2′), 2.15 (dd, *J* = 13.7, 3.4 Hz, 1H), 1.99 (ddd, *J* = 22.2, 16.8, 11.7 Hz, 4H), 1.93 (s, 1H), 1.85 (q, *J* = 6.8 Hz, 1H), 1.74 (dt, *J* = 13.4, 3.9 Hz, 1H), 1.70–1.67 (m, 1H), 1.57 (dd, *J* = 13.2, 10.2 Hz, 2H), 1.51–1.48 (m, 2H), 1.45 (dd, *J* = 13.2, 6.6 Hz, 1H), 1.41–1.37 (m, 1H), 1.36–1.32 (m, 1H), 1.27–1.17 (m, 4H), 0.99 (d, *J* = 6.6 Hz, 3H, H-18), 0.91 (d, *J* = 6.8 Hz, 3H, H-28), 0.87 (s, 3H, H-21), 0.84–0.80 (m, 9H, H-26, H-27, H-19); ^13^C NMR (150 MHz, CDCl_3_) *δ* 172.53, 170.13, 135.36, 135.21, 135.04, 133.86, 132.33, 130.93, 130.61, 128.75, 127.70, 127.54, 126.44, 124.92, 119.86, 116.51, 81.80, 79.46, 70.30, 56.17, 51.61, 51.02, 44.57, 42.78, 39.75, 39.29, 36.95, 34.27, 33.12, 33.07, 32.41, 29.93, 28.65, 26.24, 23.37, 20.89, 20.63, 19.97, 19.65, 18.05, 17.59, 12.88; HRMS (ESI) *m*/*z*: calcd for C_42_H_55_NO_5_ [M + Na]^+^: 676.4080, found: 676.3977.

Ergosterol peroxide-3-(5-((1,3,4-thiadiazol-2-yl)amino)-5-oxopentanoate) (**3a**)

White solid (78%). ^1^H NMR (600 MHz, CDCl_3_) *δ* 13.39 (s, 1H, H-N), 8.81 (s, 1H. S-CH), 6.50 (d, *J* = 8.5 Hz, 1H, H-7), 6.20 (d, *J* = 8.5 Hz, 1H, H-6), 5.22 (dd, *J* = 15.2, 7.6 Hz, 1H), 5.14 (dd, *J* = 15.3, 8.4 Hz, 1H), 5.00 (dt, *J* = 11.7, 6.1 Hz, 1H), 2.84 (t, *J* = 6.8 Hz, 2H, H-4′), 2.44 (t, *J* = 7.4 Hz, 2H, H-3′), 2.13 (q, *J* = 7.2 Hz, 3H, H-2′), 2.00 (t, *J* = 12.8 Hz, 4H), 1.94 (s, 1H), 1.85 (d, *J* = 6.7 Hz, 1H), 1.75 (d, *J* = 9.1 Hz, 1H), 1.68 (d, *J* = 13.6 Hz, 1H), 1.58–1.54 (m, 2H), 1.50 (d, *J* = 8.6 Hz, 2H), 1.47–1.45 (m, 1H), 1.42–1.39 (m, 1H), 1.35 (d, *J* = 9.5 Hz, 1H), 1.26–1.21 (m, 4H), 1.00 (d, *J* = 6.6 Hz, 3H, H-18), 0.91 (d, *J* = 6.8 Hz, 3H, H-28), 0.88 (s, 3H, H-21), 0.84–0.80 (m, 9H, H-26, H-27, H-19); ^13^C NMR (150 MHz, CDCl_3_) *δ* 171.87, 171.32, 160.48, 147.45, 135.21, 135.08, 132.32, 130.94, 81.77, 79.41, 69.60, 56.17, 51.60, 51.00, 44.57, 42.78, 39.75, 39.30, 36.95, 35.10, 34.28, 33.62, 33.15, 33.07, 29.72, 28.65, 26.28, 23.37, 20.89, 20.64, 19.97, 19.65, 18.09, 17.59, 12.88; HRMS (ESI) *m*/*z*: calcd for C_35_H_51_N_3_O_5_S [M + Na]^+^: 648.3549, found: 648.3449.

Ergosterol peroxide-3-(5-oxo-5-(thiazol-2-ylamino)pentanoate) (**3b**)

White solid (88%). ^1^H NMR (600 MHz, CDCl_3_) *δ* 7.47 (d, *J* = 3.7 Hz, 1H, H-N), 7.01 (d, *J* = 3.6 Hz, 1H, H-S), 6.50 (d, *J* = 8.5 Hz, 1H, H-7), 6.20 (d, *J* = 8.5 Hz, 1H, H-6), 5.22 (dd, *J* = 15.2, 7.7 Hz, 1H), 5.14 (dd, *J* = 15.3, 8.4 Hz, 1H), 5.00 (tt, *J* = 11.2, 5.1 Hz, 1H), 2.65 (t, *J* = 7.2 Hz, 2H, H-4′), 2.42 (t, *J* = 7.1 Hz, 2H, H-3′), 2.10 (q, *J* = 7.4 Hz, 3H, H-2′), 2.03–1.94 (m, 4H), 1.90 (d, *J* = 9.4 Hz, 1H), 1.85 (q, *J* = 6.7 Hz, 1H), 1.76 (td, *J* = 9.5, 4.5 Hz, 1H), 1.69–1.66 (m, 1H), 1.57 (dd, *J* = 19.0, 8.0 Hz, 2H), 1.50 (t, *J* = 7.6 Hz, 2H), 1.47–1.44 (m, 1H), 1.40–1.37 (m, 1H), 1.35 (d, *J* = 10.4 Hz, 1H), 1.27 (s, 4H), 1.00 (d, *J* = 6.6 Hz, 3H, H-18), 0.88 (d, *J* = 3.8 Hz, 9H, H-28, H-21, H-26), 0.83–0.81 (m, 6H, H-27, H-19); ^13^C NMR (150 MHz, CDCl_3_) *δ* 172.01, 170.49, 160.03, 136.22, 135.21, 135.05, 132.32, 130.93, 113.60, 81.74, 79.41, 69.65, 56.17, 51.61, 51.02, 44.56, 42.78, 39.75, 39.30, 36.94, 34.82, 34.28, 33.41, 33.17, 33.07, 29.72, 28.65, 26.30, 23.37, 20.88, 20.62, 19.96, 19.65, 18.06, 17.58, 12.88; HRMS (ESI) *m*/*z*: calcd for C_36_H_52_N_2_O_5_S [M + Na]^+^: 647.3597, found: 647.3501.

Ergosterol peroxide-3-(5-((5-fluorobenzo[d]thiazol-2-yl)amino)-5-oxopentanoate) (**3c**)

Yellow solid (86%). ^1^H NMR (600 MHz, CDCl_3_) *δ* 7.74 (dd, *J* = 8.8, 5.1 Hz, 1H, H-Ar), 7.44 (d, *J* = 7.1 Hz, 1H, H-Ar), 7.08 (td, *J* = 8.8, 2.4 Hz, 1H, H-Ar), 6.50 (d, *J* = 8.5 Hz, 1H, H-7), 6.21 (d, *J* = 8.5 Hz, 1H, H-6), 5.22 (dd, *J* = 15.2, 7.6 Hz, 1H), 5.14 (dd, *J* = 15.3, 8.4 Hz, 1H), 5.00 (dt, *J* = 11.6, 6.2 Hz, 1H), 2.59 (t, *J* = 7.3 Hz, 2H, H-4′), 2.40 (t, *J* = 7.1 Hz, 2H, H-3′), 2.12 (d, *J* = 8.6 Hz, 1H), 2.06 (t, *J* = 7.2 Hz, 2H, H-2′), 2.00 (t, *J* = 13.0 Hz, 4H), 1.93 (d, *J* = 9.5 Hz, 1H), 1.87–1.83 (m, 1H), 1.75 (d, *J* = 9.1 Hz, 1H), 1.69 (d, *J* = 13.5 Hz, 1H), 1.59–1.55 (m, 2H), 1.51 (d, *J* = 6.7 Hz, 2H), 1.48–1.45 (m, 1H), 1.39 (d, *J* = 5.3 Hz, 1H), 1.35 (d, *J* = 11.9 Hz, 1H), 1.25 (s, 4H), 1.00 (d, *J* = 6.6 Hz, 3H, H-18), 0.89 (d, *J* = 11.5 Hz, 6H, H-28, H-21), 0.84–0.81 (m, 9H, H-26, H-27, H-19); ^13^C NMR (150 MHz, CDCl_3_) *δ* 172.00, 170.90, 160.58, 135.21, 135.04, 132.33, 130.96, 122.33, 122.27, 112.57, 112.41, 107.21, 107.05, 81.75, 79.44, 69.85, 56.17, 51.59, 50.99, 44.57, 42.78, 39.75, 39.29, 36.95, 35.22, 34.27, 33.23, 33.15, 33.07, 29.72, 28.65, 26.28, 23.38, 20.89, 20.63, 19.97, 19.65, 18.08, 17.59, 12.89; HRMS (ESI) *m*/*z*: calcd for C_40_H_53_FN_2_O_5_S [M + Na]^+^: 715.3659, found: 715.3562.

Ergosterol peroxide-3-(5-((6-fluorobenzo[d]thiazol-2-yl)amino)-5-oxopentanoate) (**3d**)

Yellow solid (82%). ^1^H NMR (600 MHz, CDCl_3_) *δ* 7.69 (dd, *J* = 8.9, 4.6 Hz, 1H, H-Ar), 7.51 (dd, *J* = 8.0, 2.6 Hz, 1H, H-Ar), 7.18 (td, *J* = 8.9, 2.6 Hz, 1H, H-Ar), 6.51 (d, *J* = 8.5 Hz, 1H, H-7), 6.22 (d, *J* = 8.5 Hz, 1H, H-6), 5.22 (dd, *J* = 15.2, 7.6 Hz, 1H), 5.14 (dd, *J* = 15.3, 8.4 Hz, 1H), 5.00 (dt, *J* = 11.7, 6.1 Hz, 1H), 2.59 (t, *J* = 7.3 Hz, 2H, H-4′), 2.39 (t, *J* = 7.1 Hz, 2H, H-3′), 2.14–2.10 (m, 1H), 2.06 (t, *J* = 7.2 Hz, 2H, H-2′), 2.02–1.95 (m, 4H), 1.92 (d, *J* = 9.1 Hz, 1H), 1.85 (q, *J* = 6.7 Hz, 1H), 1.75 (d, *J* = 9.1 Hz, 1H), 1.69 (d, *J* = 13.6 Hz, 1H), 1.59–1.55 (m, 2H), 1.51 (d, *J* = 7.4 Hz, 2H), 1.48–1.45 (m, 1H), 1.41–1.38 (m, 1H), 1.35 (d, *J* = 11.8 Hz, 1H), 1.25 (s, 4H), 1.00 (d, *J* = 6.6 Hz, 3H, H-18), 0.91–0.88 (m, 6H, H-28, H-21), 0.84–0.81 (m, 9H, H-26, H-27, H-19); ^13^C NMR (150 MHz, CDCl_3_) *δ* 172.00, 170.87, 158.89, 158.23, 144.00, 135.21, 135.03, 132.78, 132.33, 130.97, 121.44, 114.91, 107.99, 81.75, 79.43, 69.85, 56.18, 51.60, 51.00, 44.57, 42.78, 39.75, 39.29, 36.95, 35.19, 34.27, 33.22, 33.17, 33.07, 29.72, 28.65, 26.30, 23.38, 20.89, 20.63, 19.97, 19.65, 18.08, 17.59, 12.89; HRMS (ESI) *m*/*z*: calcd for C_40_H_53_FN_2_O_5_S [M + Na]^+^: 715.3659, found: 715.3560.

Ergosterol peroxide-3-(5-oxo-5-(phenylamino)pentanoate) (**3e**)

White solid (81%). ^1^H NMR (600 MHz, CDCl_3_) *δ* 7.56–7.50 (m, 3H, H-N, H-Ar), 7.31 (t, *J* = 7.7 Hz, 2H, H-Ar), 7.09 (t, *J* = 7.4 Hz, 1H, H-Ar), 6.51 (d, *J* = 8.5 Hz, 1H, H-7), 6.22 (d, *J* = 8.5 Hz, 1H, H-6), 5.22 (dd, *J* = 15.2, 7.6 Hz, 1H), 5.14 (dd, *J* = 15.3, 8.4 Hz, 1H), 5.02 (dt, *J* = 11.8, 6.2 Hz, 1H), 2.41 (dt, *J* = 14.1, 7.2 Hz, 4H, H-4′, H-3′), 2.14–2.11 (m, 1H), 2.02 (dd, *J* = 13.5, 5.7 Hz, 6H, H-2′), 1.95 (s, 1H), 1.85 (d, *J* = 6.7 Hz, 1H), 1.75 (d, *J* = 9.1 Hz, 1H), 1.70 (d, *J* = 13.7 Hz, 1H), 1.57 (d, *J* = 17.8 Hz, 2H), 1.51 (d, *J* = 8.0 Hz, 2H), 1.48–1.45 (m, 1H), 1.42–1.38 (m, 1H), 1.35 (d, *J* = 11.8 Hz, 1H), 1.26–1.21 (m, 4H), 1.00 (d, *J* = 6.6 Hz, 3H, H-18), 0.92–0.89 (m, 6H, H-28, H-21), 0.84–0.81 (m, 9H, H-26, H-27, H-19); ^13^C NMR (150 MHz, CDCl_3_) *δ* 172.52, 170.62, 137.96, 135.21, 135.05, 132.34, 130.95, 129.00, 124.21, 119.77, 81.78, 79.46, 69.69, 56.18, 51.62, 51.03, 44.57, 42.78, 39.75, 39.30, 36.97, 36.43, 34.29, 33.39, 33.19, 33.07, 29.72, 28.65, 26.31, 23.38, 20.89, 20.63, 19.97, 19.65, 18.09, 17.59, 12.89; HRMS (ESI) *m*/*z*: calcd for C_39_H_55_NO_5_ [M + Na]^+^: 640.4080, found: 640.3985.

Ergosterol peroxide-3-(5-((4-fluorophenyl)amino)-5-oxopentanoate) (**3f**)

White solid (84%). ^1^H NMR (600 MHz, CDCl_3_) *δ* 7.65 (s, 1H, H-N), 7.48 (dd, *J* = 8.9, 4.8 Hz, 2H, H-Ar), 7.00 (t, *J* = 8.5 Hz, 2H, H-Ar), 6.51 (d, *J* = 8.5 Hz, 1H, H-7), 6.22 (d, *J* = 8.5 Hz, 1H, H-6), 5.22 (dd, *J* = 15.2, 7.6 Hz, 1H), 5.14 (dd, *J* = 15.3, 8.4 Hz, 1H), 5.01 (tt, *J* = 11.3, 5.1 Hz, 1H), 2.43–2.38 (m, 4H, H-4′, H-3′), 2.14–2.10 (m, 1H), 2.05–1.97 (m, 6H, H-2′), 1.95 (s, 1H), 1.85 (q, *J* = 6.8 Hz, 1H), 1.77–1.73 (m, 1H), 1.72–1.69 (m, 1H), 1.57 (d, *J* = 10.6 Hz, 2H), 1.51 (d, *J* = 9.3 Hz, 2H), 1.48–1.45 (m, 1H), 1.39 (d, *J* = 5.2 Hz, 1H), 1.35 (d, *J* = 12.9 Hz, 1H), 1.26–1.21 (m, 4H), 1.00 (d, *J* = 6.6 Hz, 3H, H-18), 0.92–0.89 (m, 6H, H-28, H-21), 0.83 (dd, *J* = 8.8, 5.6 Hz, 9H, H-26, H-27, H-19); ^13^C NMR (150 MHz, CDCl_3_) *δ* 172.59, 170.64, 160.10, 135.19, 135.02, 134.00, 132.35, 130.98, 121.64, 121.58, 115.66, 115.51, 81.79, 79.48, 69.74, 56.18, 51.61, 51.03, 44.57, 42.78, 39.74, 39.29, 36.97, 36.24, 34.29, 33.38, 33.18, 33.07, 29.72, 28.65, 26.31, 23.37, 20.89, 20.82, 19.97, 19.65, 18.08, 17.59, 12.88; HRMS (ESI) *m*/*z*: calcd for C_39_H_54_FNO_5_ [M + Na]^+^: 658.3986, found: 658.3886.

Ergosterol peroxide-3-(5-((4-(dimethylamino)phenyl)amino)-5-oxopentanoate) (**3g**)

White solid (78%). ^1^H NMR (600 MHz, CDCl_3_) *δ* 7.47 (s, 1H, H-N), 7.41 (d, *J* = 8.5 Hz, 2H, H-Ar), 6.84 (d, *J* = 8.8 Hz, 2H, H-Ar), 6.51 (d, *J* = 8.5 Hz, 1H, H-7), 6.22 (d, *J* = 8.5 Hz, 1H, H-6), 5.22 (dd, *J* = 15.2, 7.6 Hz, 1H), 5.14 (dd, *J* = 15.3, 8.4 Hz, 1H), 5.01 (dq, *J* = 11.8, 6.1, 5.7 Hz, 1H), 3.78 (s, 3H, H-OCH_3_), 2.39 (t, *J* = 7.1 Hz, 4H, H-4′, H-3′), 2.12 (dd, *J* = 14.3, 5.0 Hz, 1H), 2.05–1.97 (m, 6H, H-2′), 1.95 (s, 1H), 1.87–1.83 (m, 1H), 1.75 (d, *J* = 9.1 Hz, 1H), 1.70 (d, *J* = 13.8 Hz, 1H), 1.57 (d, *J* = 11.2 Hz, 2H), 1.51 (d, *J* = 9.2 Hz, 2H), 1.48–1.44 (m, 1H), 1.40 (t, *J* = 5.8 Hz, 1H), 1.35 (d, *J* = 12.9 Hz, 1H), 1.26–1.21 (m, 4H), 1.00 (d, *J* = 6.6 Hz, 3H, H-18), 0.92–0.88 (m, 6H, H-28, H-21), 0.84–0.81 (m, 9H, H-26, H-27, H-19); ^13^C NMR (150 MHz, CDCl_3_) *δ* 172.51, 170.43, 156.35, 135.21, 135.06, 132.33, 131.07, 130.94, 121.70, 114.12, 81.78, 79.45, 69.65, 56.18, 55.49, 51.61, 51.03, 44.57, 42.78, 39.75, 39.30, 36.96, 36.24, 34.29, 33.44, 33.19, 33.07, 29.72, 28.65, 26.31, 23.37, 20.92, 20.89, 19.97, 19.65, 18.08, 17.59, 12.88; HRMS (ESI) *m*/*z*: calcd for C_40_H_57_NO_6_ [M + Na]^+^: 670.4186, found: 670.4091.

Ergosterol peroxide-3-(5-(naphthalen-2-ylamino)-5-oxopentanoate) (**3h**)

White solid (82%). ^1^H NMR (600 MHz, CDCl_3_) *δ* 8.23 (s, 1H, H-N), 7.78–7.75 (m, 4H, H-Ar), 7.44 (t, *J* = 9.7 Hz, 2H, H-Ar), 7.39 (d, *J* = 7.6 Hz, 1H, H-Ar), 6.50 (d, *J* = 8.5 Hz, 1H, H-7), 6.21 (d, *J* = 8.5 Hz, 1H, H-6), 5.23–5.20 (m, 1H), 5.16–5.12 (m, 1H), 5.03 (dt, *J* = 11.7, 6.1 Hz, 1H), 2.47 (d, *J* = 7.3 Hz, 2H, H-4′), 2.43 (t, *J* = 7.0 Hz, 2H, H-3′), 2.13 (d, *J* = 8.6 Hz, 1H), 2.08–2.00 (m, 6H, H-2′), 1.94 (s, 1H), 1.85 (d, *J* = 6.8 Hz, 1H), 1.76–1.73 (m, 1H), 1.71 (s, 1H), 1.57 (d, *J* = 12.0 Hz, 2H), 1.50 (d, *J* = 6.4 Hz, 2H), 1.47–1.45 (m, 1H), 1.40 (dd, *J* = 11.6, 5.3 Hz, 1H), 1.35 (d, *J* = 12.1 Hz, 1H), 1.26–1.22 (m, 4H), 1.00 (d, *J* = 6.6 Hz, 3H, H-18), 0.91 (d, *J* = 6.8 Hz, 3H, H-28), 0.88 (s, 3H, H-21), 0.84–0.81 (m, 9H, H-26, H-27, H-19); ^13^C NMR (150 MHz, CDCl_3_) *δ* 172.57, 170.84, 135.41, 135.21, 135.05, 133.88, 132.34, 130.96, 130.61, 128.76, 127.70, 127.56, 126.48, 124.95, 119.84, 116.49, 81.79, 79.46, 69.73, 56.18, 51.61, 51.03, 44.57, 42.79, 39.75, 39.29, 36.97, 36.48, 34.30, 33.41, 33.20, 33.07, 29.72, 28.65, 26.33, 23.37, 20.89, 19.98, 19.66, 18.08, 17.59, 12.89; HRMS (ESI) *m*/*z*: calcd for C_43_H_57_NO_5_ [M + Na]^+^: 690.4237, found: 690.4139.

Ergosterol peroxide-3-(2-((1,3,4-thiadiazol-2-yl)carbamoyl)benzoate) (**4a**)

White solid (77%). ^1^H NMR (600 MHz, CDCl_3_) *δ* 8.77 (s, 1H, H-N), 8.01 (d, *J* = 7.6 Hz, 1H, S-CH), 7.64 (ddd, *J* = 29.0, 15.0, 7.4 Hz, 4H, H-Ar), 6.47 (d, *J* = 8.5 Hz, 1H, H-7), 6.10 (d, *J* = 8.5 Hz, 1H, H-6), 5.21 (d, *J* = 4.6 Hz, 1H), 5.16–5.12 (m, 2H), 2.12 (d, *J* = 8.3 Hz, 1H), 2.00–1.94 (m, 4H), 1.92 (s, 1H), 1.85 (s, 1H), 1.74 (d, *J* = 8.4 Hz, 1H), 1.69 (dd, *J* = 13.5, 3.8 Hz, 1H), 1.56 (s, 2H), 1.46 (s, 2H), 1.41–1.39 (m, 1H), 1.37 (s, 1H), 1.33 (s, 1H), 1.25 (s, 4H), 0.99 (s, 3H, H-18), 0.91 (d, *J* = 6.9 Hz, 3H, H-28), 0.83–0.80 (m, 9H, H-21, H-26, H-27), 0.72 (s, 3H, H-19); ^13^C NMR (150 MHz, CDCl_3_) *δ* 167.12, 165.13, 159.74, 147.49, 135.43, 135.22, 134.93, 132.32, 131.03, 130.94, 130.76, 130.70, 129.98, 128.07, 81.60, 79.39, 71.23, 56.19, 51.68, 51.07, 44.54, 42.78, 39.74, 39.27, 36.96, 34.11, 33.07, 32.81, 28.67, 25.91, 23.40, 20.89, 20.61, 19.96, 19.65, 18.19, 17.59, 12.87; HRMS (ESI) *m*/*z*: calcd for C_38_H_49_N_3_O_5_S [M + Na]^+^: 682.3393, found: 682.3285.

Ergosterol peroxide-3-(2-oxo-5-((thiazol-2-yl)carbamoyl)benzoate) (**4b**)

White solid (86%). ^1^H NMR (600 MHz, CDCl_3_) *δ* 8.13–8.11 (m, 1H, N-CH), 7.65 (td, *J* = 4.5, 2.0 Hz, 2H, H-Ar), 7.59–7.57 (m, 1H, S-CH), 6.77 (d, *J* = 3.7 Hz, 1H, H-Ar), 6.44 (d, *J* = 8.5 Hz, 1H, H-7), 6.09 (d, *J* = 3.6 Hz, 1H, H-Ar), 6.02 (d, *J* = 8.5 Hz, 1H, H-6), 5.21 (dd, *J* = 15.3, 7.7 Hz, 1H), 5.16–5.09 (m, 2H), 2.00 (dd, *J* = 13.2, 4.9 Hz, 2H), 1.92 (dt, *J* = 13.5, 3.4 Hz, 2H), 1.84 (q, *J* = 6.8 Hz, 2H), 1.78 (d, *J* = 12.0 Hz, 1H), 1.75–1.72 (m, 1H), 1.58 (s, 1H), 1.56–1.51 (m, 2H), 1.46–1.43 (m, 2H), 1.36 (d, *J* = 6.8 Hz, 1H), 1.33 (s, 1H), 1.25 (s, 1H), 1.24–1.09 (m, 4H), 0.99 (d, *J* = 6.6 Hz, 3H, H-18), 0.90 (d, *J* = 6.8 Hz, 3H, H-28), 0.83–0.78 (m, 9H, H-21, H-26, H-27), 0.61 (s, 3H, H-19); ^13^C NMR (150 MHz, CDCl_3_) *δ* 167.49, 164.50, 160.02, 136.59, 136.07, 135.21, 134.97, 132.53, 132.32, 130.86, 130.84, 130.36, 129.11, 128.11, 113.07, 81.54, 79.33, 71.06, 56.15, 51.54, 50.89, 44.53, 42.78, 39.74, 39.26, 36.84, 34.09, 33.07, 32.65, 28.63, 25.75, 23.32, 20.89, 20.61, 19.97, 19.65, 17.75, 17.59, 12.87; HRMS (ESI) *m*/*z*: calcd for C_39_H_50_N_2_O_5_S [M + Na]^+^: 681.3440, found: 681.3334.

Ergosterol peroxide-3-(2-((5-fluorobenzo[d]thiazol-2-yl)carbamoyl)benzoate) (**4c**)

Yellow solid (82%). ^1^H NMR (600 MHz, CDCl_3_) *δ* 7.91–7.89 (m, 1H, H-Ar), 7.70 (dd, *J* = 8.7, 5.1 Hz, 1H, H-Ar), 7.55–7.53 (m, 1H, H-Ar), 7.49–7.46 (m, 2H, H-Ar), 7.00 (t, *J* = 7.5 Hz, 1H, H-Ar), 6.50 (d, *J* = 9.4 Hz, 1H, H-Ar), 6.42 (d, *J* = 8.5 Hz, 1H, H-7), 5.99 (d, *J* = 8.5 Hz, 1H, H-6), 5.22–5.18 (m, 1H), 5.12 (dd, *J* = 15.3, 8.8 Hz, 2H), 2.13 (dd, *J* = 13.8, 5.1 Hz, 1H), 1.99–1.94 (m, 3H), 1.93–1.89 (m, 2H), 1.84 (d, *J* = 6.8 Hz, 1H), 1.72 (d, *J* = 9.5 Hz, 1H), 1.60 (d, *J* = 14.4 Hz, 1H), 1.51 (d, *J* = 7.1 Hz, 2H), 1.43 (d, *J* = 9.1 Hz, 2H), 1.34 (d, *J* = 8.7 Hz, 1H), 1.26 (s, 2H), 1.20–1.10 (m, 4H), 0.97 (d, *J* = 6.5 Hz, 3H, H-18), 0.90 (d, *J* = 6.8 Hz, 3H, H-28), 0.83–0.80 (m, 6H, H-21, H-26), 0.77 (s, 3H, H-27), 0.66 (s, 3H, H-19); ^13^C NMR (150 MHz, CDCl_3_) *δ* 167.87, 164.92, 161.64, 135.52, 135.21, 134.86, 132.48, 132.30, 130.88, 130.70, 129.55, 127.72, 126.72, 121.94, 121.88, 112.48, 112.32, 106.67, 106.50, 81.59, 79.33, 71.42, 56.14, 51.53, 50.89, 44.52, 42.77, 39.73, 39.23, 36.87, 34.12, 33.07, 32.73, 28.62, 25.96, 23.31, 20.87, 20.59, 19.96, 19.65, 17.73, 17.58, 12.86; HRMS (ESI) *m*/*z*: calcd for C_43_H_51_FN_2_O_5_S [M + H]^+^: 727.3503, found: 727.3580.

Ergosterol peroxide-3-(2-((6-fluorobenzo[d]thiazol-2-yl)carbamoyl)benzoate) (**4d**)

Yellow solid (80%). ^1^H NMR (600 MHz, CDCl_3_) *δ* 7.96–7.92 (m, 1H, N-H), 7.58–7.56 (m, 1H, H-Ar), 7.53–7.48 (m, 4H, H-Ar), 7.14 (dd, *J* = 8.9, 4.5 Hz, 1H, H-Ar), 7.00–6.98 (m, 1H, H-Ar), 6.43 (d, *J* = 8.5 Hz, 1H, H-7), 5.99 (d, *J* = 8.5 Hz, 1H, H-6), 5.22–5.19 (m, 1H), 5.17–5.10 (m, 2H), 2.15–2.12 (m, 1H), 1.98 (dd, *J* = 15.6, 7.8 Hz, 4H), 1.92 (s, 1H), 1.84 (d, *J* = 6.8 Hz, 1H), 1.73 (d, *J* = 8.8 Hz, 1H), 1.62 (d, *J* = 3.7 Hz, 1H), 1.59 (d, *J* = 12.7 Hz, 2H), 1.53 (d, *J* = 11.7 Hz, 2H), 1.47 (d, *J* = 6.3 Hz, 1H), 1.43 (s, 1H), 1.41 (s, 1H), 1.26 (s, 4H), 0.98 (d, *J* = 6.6 Hz, 3H, H-18), 0.90 (d, *J* = 6.9 Hz, 3H, H-28), 0.83–0.80 (m, 6H, H-21, H-26,), 0.77 (s, 3H, H-27), 0.64 (s, 3H, H-19); ^13^C NMR (150 MHz, CDCl_3_) *δ* 167.61, 165.00, 160.43, 158.80, 135.35, 135.20, 134.83, 132.53, 132.39, 132.32, 130.91, 130.76, 129.59, 127.80, 121.27, 114.76, 114.60, 107.67, 107.50, 81.61, 79.36, 71.46, 56.15, 51.54, 50.90, 44.53, 42.78, 39.73, 39.24, 36.87, 34.13, 33.07, 32.74, 28.62, 25.94, 23.32, 20.87, 20.59, 19.96, 19.65, 17.69, 17.58, 12.86; HRMS (ESI) *m*/*z*: calcd for C_43_H_51_FN_2_O_5_S [M + H]^+^: 727.3503, found: 727.3607.

Ergosterol peroxide-3-(2-oxo-5-((phenyl)carbamoyl)benzoate) (**4e**)

White solid (82%). ^1^H NMR (600 MHz, CDCl_3_) *δ* 7.97 (d, *J* = 7.9 Hz, 1H, H-N), 7.74 (s, 1H, H-Ar), 7.65 (d, *J* = 8.0 Hz, 2H, H-Ar), 7.58–7.50 (m, 3H, H-Ar), 7.35 (t, *J* = 7.9 Hz, 2H, H-Ar), 7.13 (t, *J* = 7.4 Hz, 1H, H-Ar), 6.42 (d, *J* = 8.5 Hz, 1H, H-7), 5.95 (d, *J* = 8.5 Hz, 1H, H-6), 5.21 (dd, *J* = 15.2, 7.6 Hz, 1H), 5.13 (dd, *J* = 15.5, 8.4 Hz, 2H), 2.00 (s, 1H), 1.99–1.91 (m, 4H), 1.85 (d, *J* = 6.2 Hz, 1H), 1.83–1.80 (m, 1H), 1.72 (d, *J* = 9.5 Hz, 1H), 1.59 (d, *J* = 14.0 Hz, 1H), 1.47–1.44 (m, 2H), 1.42 (d, *J* = 11.5 Hz, 2H), 1.36 (d, *J* = 7.6 Hz, 1H), 1.32 (d, *J* = 11.9 Hz, 1H), 1.25 (s, 1H), 1.17 (p, *J* = 12.9, 11.6 Hz, 4H), 0.98 (d, *J* = 6.6 Hz, 3H, H-18), 0.90 (d, *J* = 6.9 Hz, 3H, H-28), 0.82 (dd, *J* = 10.1, 6.8 Hz, 6H, H-21, H-26), 0.78 (s, 3H, H-27), 0.63 (s, 3H, H-19); ^13^C NMR (150 MHz, CDCl_3_) *δ* 167.65, 165.47, 138.42, 138.29, 135.21, 135.08, 132.32, 130.67, 129.80, 129.17, 128.76, 127.63, 124.36, 119.41, 81.70, 79.38, 71.17, 56.15, 51.50, 50.89, 44.52, 42.78, 39.72, 39.27, 36.86, 34.09, 33.07, 32.83, 28.62, 25.90, 23.29, 20.87, 20.61, 19.97, 19.65, 17.79, 17.59, 12.84; HRMS (ESI) *m*/*z*: calcd for C_42_H_53_NO_5_ [M + Na]^+^: 674.3924, found: 674.3822.

Ergosterol peroxide-3-(2-((4-fluorophenyl)carbamoyl)benzoate) (**4f**)

White solid (78%). ^1^H NMR (600 MHz, CDCl_3_) *δ* 7.94 (d, *J* = 7.8 Hz, 1H, N-H), 7.84 (s, 1H, H-Ar), 7.63 (dd, *J* = 8.8, 4.7 Hz, 2H, H-Ar), 7.58–7.54 (m, 2H, H-Ar), 7.50 (t, *J* = 6.7 Hz, 1H, H-Ar), 7.05 (t, *J* = 8.6 Hz, 2H, H-Ar), 6.45 (d, *J* = 8.5 Hz, 1H, H-7), 6.01 (d, *J* = 8.4 Hz, 1H, H-6), 5.21 (dd, *J* = 15.2, 7.6 Hz, 1H), 5.14 (dd, *J* = 15.3, 8.3 Hz, 2H), 2.05–2.02 (m, 1H), 1.99–1.92 (m, 4H), 1.86 (d, *J* = 6.5 Hz, 1H), 1.84 (d, *J* = 6.3 Hz, 1H), 1.73–1.70 (m, 1H), 1.62 (d, *J* = 10.3 Hz, 1H), 1.53–1.48 (m, 2H), 1.45 (d, *J* = 7.1 Hz, 2H), 1.38 (dd, *J* = 11.5, 5.0 Hz, 1H), 1.35 (s, 1H), 1.33 (d, *J* = 11.0 Hz, 1H), 1.25 (s, 4H), 0.99 (d, *J* = 6.5 Hz, 3H, H-18), 0.91 (d, *J* = 6.9 Hz, 3H, H-28), 0.82 (dd, *J* = 10.0, 6.7 Hz, 6H, H-21, H-26), 0.78 (s, 3H, H-27), 0.69 (s, 3H, H-19); ^13^C NMR (150 MHz, CDCl_3_) *δ* 167.52, 165.58, 160.18, 158.56, 138.06, 135.20, 134.95, 132.34, 132.29, 130.86, 130.58, 129.88, 128.82, 127.76, 121.25, 121.20, 115.84, 115.69, 81.72, 79.45, 71.22, 56.15, 51.50, 50.90, 44.54, 42.79, 39.71, 39.26, 36.89, 34.10, 33.07, 32.90, 28.62, 25.96, 23.31, 20.87, 20.60, 19.97, 19.65, 17.80, 17.59, 12.85; HRMS (ESI) *m*/*z*: calcd for C_42_H_52_FNO_5_ [M + Na]^+^: 692.3830, found: 692.3731.

Ergosterol peroxide-3-(2-((4-(dimethylamino)phenyl)carbamoyl)benzoate) (**4g**)

White solid (80%). ^1^H NMR (600 MHz, CDCl_3_) *δ* 7.92 (d, *J* = 7.7 Hz, 1H, H-N), 7.69 (s, 1H, H-Ar), 7.59–7.55 (m, 4H, H-Ar), 7.50–7.47 (m, 1H, H-Ar), 6.89 (d, *J* = 8.9 Hz, 2H, H-Ar), 6.44 (d, *J* = 8.5 Hz, 1H, H-7), 6.02 (d, *J* = 8.5 Hz, 1H, H-6), 5.21 (dd, *J* = 15.2, 7.7 Hz, 1H), 5.14 (dd, *J* = 15.7, 8.0 Hz, 2H), 3.81 (s, 3H, H-OCH_3_), 2.05 (dd, *J* = 13.8, 5.1 Hz, 1H), 1.97–1.88 (m, 4H), 1.86–1.83 (m, 1H), 1.73 (d, *J* = 9.4 Hz, 1H), 1.63–1.60 (m, 1H), 1.56 (d, *J* = 9.5 Hz, 1H), 1.50 (d, *J* = 7.7 Hz, 2H), 1.48–1.45 (m, 2H), 1.43 (s, 1H), 1.37 (d, *J* = 9.7 Hz, 1H), 1.33 (d, *J* = 9.9 Hz, 1H), 1.26–1.15 (m, 4H), 0.99 (d, *J* = 6.6 Hz, 3H, H-18), 0.91 (d, *J* = 6.8 Hz, 3H, H-28), 0.84–0.81 (m, 6H, H-21, H-26), 0.79 (s, 3H, H-27), 0.68 (s, 3H, H-19); ^13^C NMR (150 MHz, CDCl_3_) *δ* 167.34, 165.70, 156.42, 138.32, 135.21, 135.06, 132.32, 132.22, 131.65, 130.73, 130.50, 129.73, 127.72, 121.14, 114.29, 81.73, 79.39, 71.16, 56.15, 51.55, 50.94, 44.54, 42.78, 39.73, 39.27, 36.88, 34.15, 33.07, 32.86, 28.63, 25.93, 23.32, 20.88, 20.61, 19.97, 19.65, 17.79, 17.59, 12.86; HRMS (ESI) *m*/*z*: calcd for C_43_H_55_NO_6_ [M + Na]^+^: 704.4029, found: 704.3925.

Ergosterol peroxide-3-(2-(naphthalen-2-ylcarbamoyl)benzoate) (**4h**)

White solid (83%). ^1^H NMR (600 MHz, CDCl_3_) *δ* 8.43 (s, 1H, H-N), 7.99 (d, *J* = 7.8 Hz, 1H, H-Ar), 7.86 (s, 1H, H-Ar), 7.81 (d, *J* = 9.8 Hz, 3H, H-Ar), 7.61 (d, *J* = 4.4 Hz, 2H, H-Ar), 7.53–7.47 (m, 3H, H-Ar), 7.42 (d, *J* = 7.4 Hz, 1H, H-Ar), 6.34 (d, *J* = 8.5 Hz, 1H, H-7), 5.79 (d, *J* = 8.5 Hz, 1H, H-6), 5.22–5.18 (m, 1H), 5.15–5.10 (m, 2H), 2.02 (d, *J* = 8.4 Hz, 1H), 1.94–1.84 (m, 4H), 1.83 (d, *J* = 7.1 Hz, 1H), 1.70 (d, *J* = 9.3 Hz, 1H), 1.54 (d, *J* = 5.9 Hz, 1H), 1.52 (s, 1H), 1.46 (d, *J* = 6.6 Hz, 2H), 1.35 (d, *J* = 12.1 Hz, 2H), 1.32 (d, *J* = 4.8 Hz, 1H), 1.29 (s, 1H), 1.25 (s, 1H), 1.18–1.03 (m, 4H), 0.96 (d, *J* = 6.5 Hz, 3H, H-18), 0.90 (d, *J* = 6.8 Hz, 3H, H-28), 0.83–0.80 (m, 6H, H-21, H-26), 0.74 (s, 3H, H-27), 0.47 (s, 3H, H-19); ^13^C NMR (150 MHz, CDCl_3_) *δ* 167.74, 165.58, 138.22, 135.73, 135.20, 134.90, 133.95, 132.36, 132.30, 130.71, 130.61, 129.92, 128.99, 128.87, 127.75, 127.70, 127.58, 126.66, 125.11, 119.47, 116.25, 81.64, 79.33, 71.24, 56.12, 51.50, 50.87, 44.50, 42.78, 39.71, 39.22, 36.77, 34.09, 33.07, 32.82, 28.60, 25.91, 23.25, 20.86, 20.58, 19.96, 19.64, 17.58, 17.49, 12.83; HRMS (ESI) *m*/*z*: calcd for C_46_H_55_NO_5_ [M + Na]^+^: 724.4080, found: 724.3981.

### 3.2. GLS1 Enzyme Assay

MDA-MB-231 cells were cultured for 24 h and then incubated with different concentrations of **3g** or 0.1%DMSO for 48 h and washed with PBS 1–2 times. The cells were then collected, subjected to ultrasound in an ice bath for 5 min, and centrifuged at 4 °C and 1200 g for 15 min. Next, the GLS1 activity in human GLS1 was evaluated according to the manufacturer’s guidelines using a GLS1 screening kit (Sangon Biotech #D799306, Shanghai, China) in a 96-well plate (Corning # 351172, New York, NY, USA).

### 3.3. Cell Proliferation Assay

The MDA-MB-231 cell line was purchased from the Shanghai Cell Bank of the Chinese Academy of Sciences (Shanghai, China). Cells containing 10% fetal bovine serum (FBS; WISENT, Nanjing, China) were cultured in an L-15 culture medium (L-15, BOSTER, Wuhan, China) in an incubator at 37 °C. The cells were then inoculated into 96-well plates at a density of 5 × 10^3^/well, cultured in 100 μL L-15 medium for 24 h, and incubated with the compound to be tested for 48 h. Next, 20 μL of MTT (5 mg/mL) solution was added and incubated at 37 °C for 4 h away from light. A resorption culture medium was then supplemented with 150 μL DMSO per well. The absorbance was measured at 570 nm using an enzymometer (SAFIRE2, Tecan, Swiss Confederation, Männedorf, Switzerland). The cytotoxic activity was presented in terms of half the inhibition rate.

### 3.4. Colony Formation Assay

MDA-MB-231 cells (1000 cells/well) were seeded into a 6-well plate with 2 mL L–15 culture medium and incubated at 37 °C for 24 h. Following this process, different concentrations of **3g** or EP were added and incubated for an additional 24 h before the culture medium was replaced. The cells then continued to be cultured for two weeks, with the culture medium being changed twice a week. After two weeks, the cell colonies were washed with PBS, fixed using a 4% paraformaldehyde solution (Beyotime, Shanghai, China), and subsequently stained with a 0.05% crystal violet solution (Beyotime, Shanghai, China).

### 3.5. Apoptosis Assays

An Annexin V FITC/PI apoptosis detection kit (Keygen Company, Nanjing, China) was employed to identify apoptotic cells. Briefly, MDA-MB-231 cells were subjected to treatment with varying concentrations of **3g** or 0.1% DMSO and incubated at 37 °C for 48 h. Subsequently, the cells were harvested and resuspended in PBS at a concentration of 1 × 10^5^ cells/mL. Following centrifugation at 2000 rpm/min for 5 min, 500 μL binding buffer, 5 μL Annexin V FITC, and 5 μL propidium iodide (PI) were added. The mixture was then incubated at room temperature in the absence of light for 20 min. Finally, the samples were analyzed using FACSCalibur flow cytometry (Becton, Dickinson and Company, Franklin Lakes, USA).

### 3.6. Western Blot Analysis

MDA-MB-231 cells were subjected to treatment with different concentrations of **3g** or 0.1% DMSO for 48 h. Subsequently, the cells were gathered, and the total protein was extracted by adding protease inhibitors and phosphatase inhibitors along with 1 × RIPA lysis buffer (Beyotime Company). The quantification of the extracted protein was carried out using a BCA kit (ComWin Biotech Company, Beijin, China). The extracted total protein, with an amount of 20 μg/well, underwent SDS–polyacrylamide gel electrophoresis (SDS-PAGE, provided by BioRad Laboratories located in Hercules, CA, USA). The protein was then wet-transferred to a PVDF membrane. Next, GLS1, Bcl-2, Bax, Cyt C, caspase-9, cleaved caspase-9, caspase-3, cleaved caspase-3, and GAPDH were incubated at 4 °C overnight. Subsequently, the secondary isotype-specific antibody, which was labeled with horseradish peroxidase, was obtained from Cell Signaling Technology. The color was developed with an ECL luminescent solution (Meilun Biotech Company, Dalian, China) and detected using a gel imager (ChemiDoc MP, BioRad Laboratories, CA, USA).

### 3.7. Determination of Intracellular Glutamate Levels

MDA-MB-231 cells were cultured for 24 h, incubated with different concentrations of **3g** or 0.1% DMSO for 48 h, and washed with PBS 1–2 times. Then, the collected cells were lysed with an extraction buffer, crushed in an ultrasonic ice bath for 5 min at 8000 *g*, and centrifuged at room temperature for 10 min. Then, the cells were tested according to a CheKine^TM^ glutamate detection kit (KTB1440, Abbkine Scientific Company, Wuhan, China).

### 3.8. ROS Assay

MDA-MB-231 cells were inoculated in 24-well plates at a density of 8 × 10^3^ cells per well and incubated with different concentrations of 3 g or 0.1% DMSO for 48 h. The cells were washed with PBS 1–2 times. The cells were then incubated at 37 °C for 20 min with reference to a DCFH-DA fluorescent probe kit (Keygen Company, Nanjing, China) and washed with a serum-free medium 1–2 times. The ROS levels were observed via laser confocal microscopy (LSM710, Zeiss, Oberkochen, Germany) or detected with flow cytometry (FACS Calibur, Becton, Dickinson and Company, Franklin Lakes, NJ, USA).

### 3.9. Molecular Docking

The crystal structure of human GLS1 (PDB ID: 3UO9) was retrieved from the PDB protein database. The ligand molecules were then sketched using the Chemdraw 19.0 software (CambridgeSoft Corporation, Cambridge, MA, USA). Then, the MM2 field minimum energy was quantified, and the mol2 structure file was derived. Molecular docking was performed with the Autodock 1.5.7 software (Scripps Software), with the docking results visualized using the PyMOL 2.6.0 software (DeLano Sciences, South San Francisco, CA, USA).

### 3.10. In Vivo Model

All the animal testing procedures were carried out in accordance with the National Institutes of Health guidelines concerning the care and use of laboratory animals. The relevant animal testing content received authorization from the Experimental Animal Ethics Committee of Qiqihar Medical College (approval number: QMU-AECC-2022-43). Female BALB/c mice that were three weeks old were procured from the Laboratory Animal Science Department of Harbin Medical University, which was located in Harbin, China. Logarithmic 4T1 cells were collected and suspended in PBS, and each mouse was inoculated subcutaneously with 100 μL of PBS containing 1 × 10^6^ cells. When the tumor reached a size of about 100 mm^3^, the tumor-bearing mice were randomly divided into a blank control group, positive control group (BPTES, 25 mg/kg), and treatment group (**3g**, 25 mg/kg, or 50 mg/kg). An intraperitoneal injection was administered every two days, and the tumor and mouse weights were recorded. After 14 days, the mice were humanely euthanized. Subsequently, the tumors and internal organs from each group were collected. These were then fixed using 4% paraformaldehyde to prepare them for the subsequent experiments.

## 4. Conclusions

In this study, a series of potential EP-based GLS1 inhibitors was designed, synthesized, and evaluated. These EP derivatives exhibit high antiproliferative activities against MDA-MB-231 cells that overexpress GLS1. Compound **3g** offered higher GLS1-inhibitory effects than EP, with similar inhibitory activities compared to BPTES on GLS1. Compound **3g** reduced the glutamate levels by blocking the glutamine hydrolysis pathway and further triggered the production of ROS, thereby activating the mitochondrial apoptosis pathway and promoting cell apoptosis. In molecular docking, **3g** bound to GLS1, indicating its good binding capabilities and significantly lower binding energy than EP. In addition, **3g** effectively inhibited tumor growth in model mice without significant toxicity. Therefore, as a natural GLS1 inhibitor, **3g** has good application prospects in the treatment of breast cancer and is worthy of further development.

## Figures and Tables

**Table 1:** Antiproliferative activities of EP derivatives **1a–h**, **2a–h**, **3a–h**, and **4a–h** against four tumor cell lines.

Compound	IC_50_^a^ (μM)			
	A549	HepG2	MCF-7	MDA-MB-231
**1a**	12.45 ± 0.84	8.89 ± 0.41	8.44 ± 0.82	11.61 ± 1.54
**1b**	16.76 ± 1.33	10.97 ± 0.82	9.07 ± 0.52	17.66 ± 1.71
**1c**	12.88 ± 0.91	11.35 ± 0.76	15.35 ± 1.22	19.89 ± 1.08
**1d**	17.28 ± 1.12	13.31 ± 1.05	10.85 ± 1.32	12.73 ± 0.26
**1e**	10.01 ± 0.61	15.17 ± 1.03	11.61 ± 0.96	11.69 ± 0.57
**1f**	13.99 ± 0.87	8.99 ± 0.67	8.03 ± 0.68	9.24 ± 0.41
**1g**	15.96 ± 1.42	11.53 ± 0.87	9.05 ± 0.49	10.10 ± 0.85
**1h**	17.93 ± 1.21	12.09 ± 1.11	9.27 ± 0.38	9.05 ± 1.02
**2a**	18.40 ± 1.40	10.98 ± 0.93	7.66 ± 1.01	16.83 ± 0.95
**2b**	16.23 ± 1.17	9.83 ± 0.76	6.39 ± 0.28	17.10 ± 0.83
**2c**	14.08 ± 0.98	9.78 ± 0.81	13.45 ± 1.03	16.55 ± 0.72
**2d**	9.86 ± 1.64	9.51 ± 0.73	12.93 ± 0.78	14.79 ± 1.27
**2e**	17.13 ± 1.22	18.17 ± 0.71	7.40 ± 0.28	5.19 ± 0.69
**2f**	13.42 ± 0.71	15.76 ± 1.27	10.93 ± 0.84	8.11 ± 0.76
**2g**	10.28 ± 0.38	12.20 ± 1.15	5.38 ± 0.38	6.02 ± 0.71
**2h**	14.88 ± 1.10	12.63 ± 1.08	14.74 ± 1.34	14.00 ± 0.79
**3a**	9.73 ± 0.42	10.66 ± 0.82	5.16 ± 0.93	7.06 ± 0.35
**3b**	11.69 ± 0.82	13.06 ± 1.22	9.47 ± 0.75	6.92 ± 0.28
**3c**	11.38 ± 0.79	11.87 ± 1.07	11.04 ± 0.62	11.99 ± 0.72
**3d**	9.09 ± 0.64	14.67 ± 0.35	8.39 ± 0.34	10.79 ± 0.53
**3e**	10.52 ± 0.79	14.91 ± 1.13	11.55 ± 0.66	4.78 ± 0.41
**3f**	11.08 ± 0.93	9.79 ± 1.01	8.09 ± 0.71	7.19 ± 0.39
**3g**	13.52 ± 1.14	15.48 ± 0.63	4.57 ± 0.62	3.20 ± 0.29
**3h**	9.30 ± 0.66	13.84 ± 1.30	8.65 ± 0.73	4.98 ± 0.84
**4a**	7.07 ± 0.47	8.08 ± 0.64	8.20 ± 0.68	10.19 ± 0.62
**4b**	12.80 ± 1.08	10.95 ± 0.76	12.64 ± 0.94	15.77 ± 1.26
**4c**	11.69 ± 1.02	11.40 ± 0.98	10.71 ± 0.47	7.32 ± 0.58
**4d**	8.99 ± 0.81	15.32 ± 1.31	10.56 ± 0.52	11.31 ± 0.44
**4e**	12.58 ± 0.99	18.54 ± 1.18	15.40 ± 1.21	9.07 ± 0.93
**4f**	18.79 ± 0.64	9.02 ± 0.87	9.64 ± 0.51	13.39 ± 1.24
**4g**	17.62 ± 1.39	15.59 ± 0.84	9.49 ± 0.24	8.16 ± 0.80
**4h**	10.13 ± 1.16	14.29 ± 1.55	11.39 ± 0.42	14.57 ± 0.47
**EP**	14.95 ± 1.27	18.05 ± 1.71	19.42 ± 1.50	17.26 ± 1.05
**BPTES**	49.08 ± 11.60	60.23 ± 8.35	21.26 ± 6.89	9.10 ± 0.26
**Cisplatin**	11.64 ± 0.38	6.29 ± 0.30	4.23 ± 0.24	10.37 ± 0.34

^a^ IC_50_: the concentration of a compound that inhibits cell growth by 50%. All data in the table above are averages of three independent experiments ± SD.

## Data Availability

The data will be provided upon request.

## Supplementary Materials

The following supporting information can be downloaded at https://www.mdpi.com/article/10.3390/molecules29184375/s1.
