# Supplementary material for: Discovery and Optimization of Ergosterol Peroxide Derivatives as Novel Glutaminase 1 Inhibitors for the Treatment of Triple-Negative Breast Cancer
Source: Molecules. 2024 Sep 14;29(18):4375. doi: 10.3390/molecules29184375 (PMC11434480; doi:10.3390/molecules29184375)

Supplemental Material

Discovery and optimization of ergosterol peroxide derivatives as novel glutaminase 1 inhibitors for the treatment of triple-negative breast cancer

Ran Luo [a,1], Haoyi Zhao [a,1], Siqi Deng [a], Jiale Wu [b], Haijun Wang [a], Xiaoshan Guo [a], Cuicui Han [a], Wenkang Ren [a], Yinglong Han [a], Jianwen Zhou [c], Yu Lin [a,*] and Ming Bu [a,*]

[a] College of Pharmacy, Qiqihar Medical University, Qiqihar, 161006, Heilongjiang, PR China
[b] College of Pharmacy, Hainan University, Haikou, 570228, Hainan, PR China
[c] Research Institute of Medicine & Pharmacy, Qiqihar Medical University, Qiqihar, 161006, Heilongjiang, PR China

* Corresponding author.
E-mail address: buming@qmu.edu.cn (M. Bu); linyu7373@163.com (Y. Lin); Tel.: +86-0452-2663-881

[1] Both authors contributed equally to this work.

^{1}H NMR and ^{13}C NMR of **1**

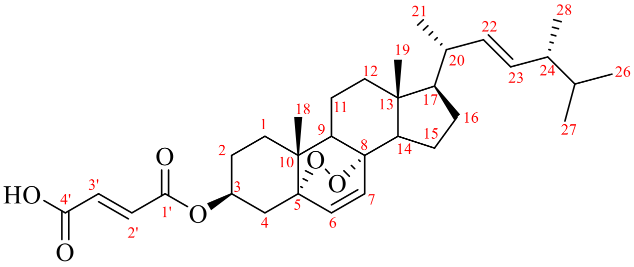

1

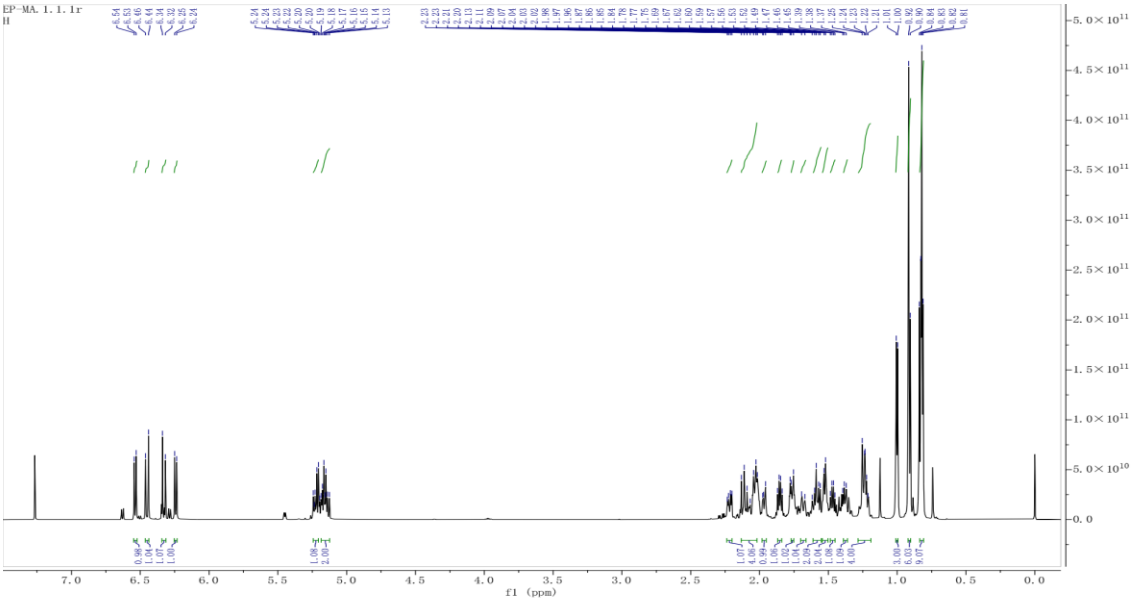

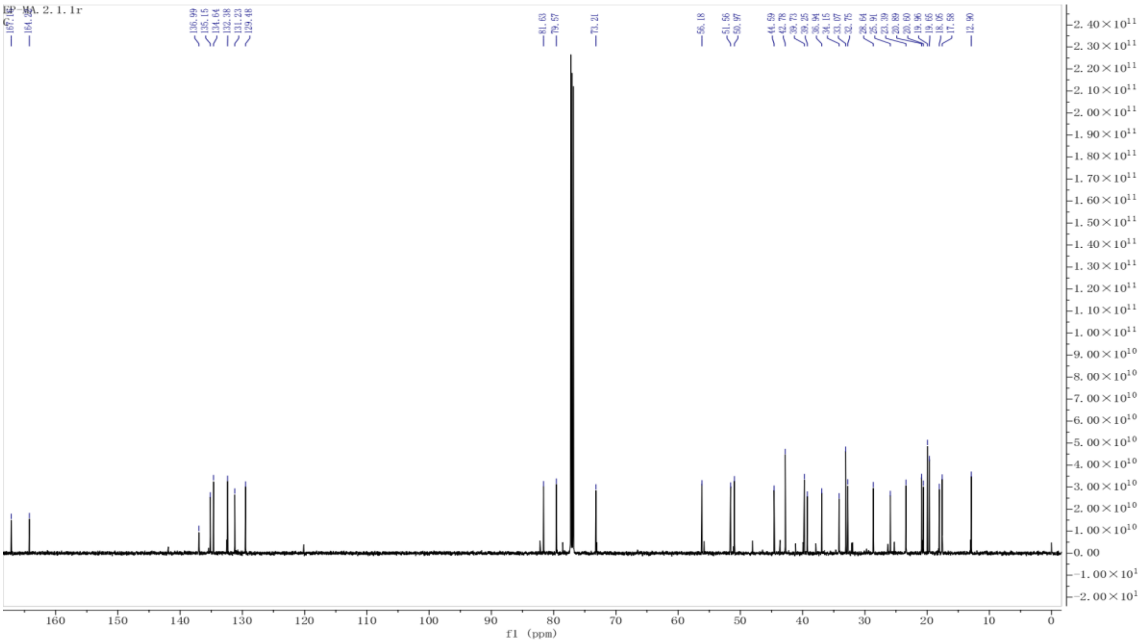

^{1}H NMR, ^{13}C NMR and HRMS spectra of **1a**

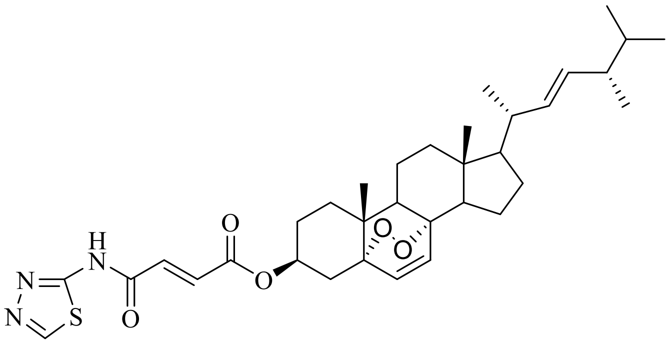

1a

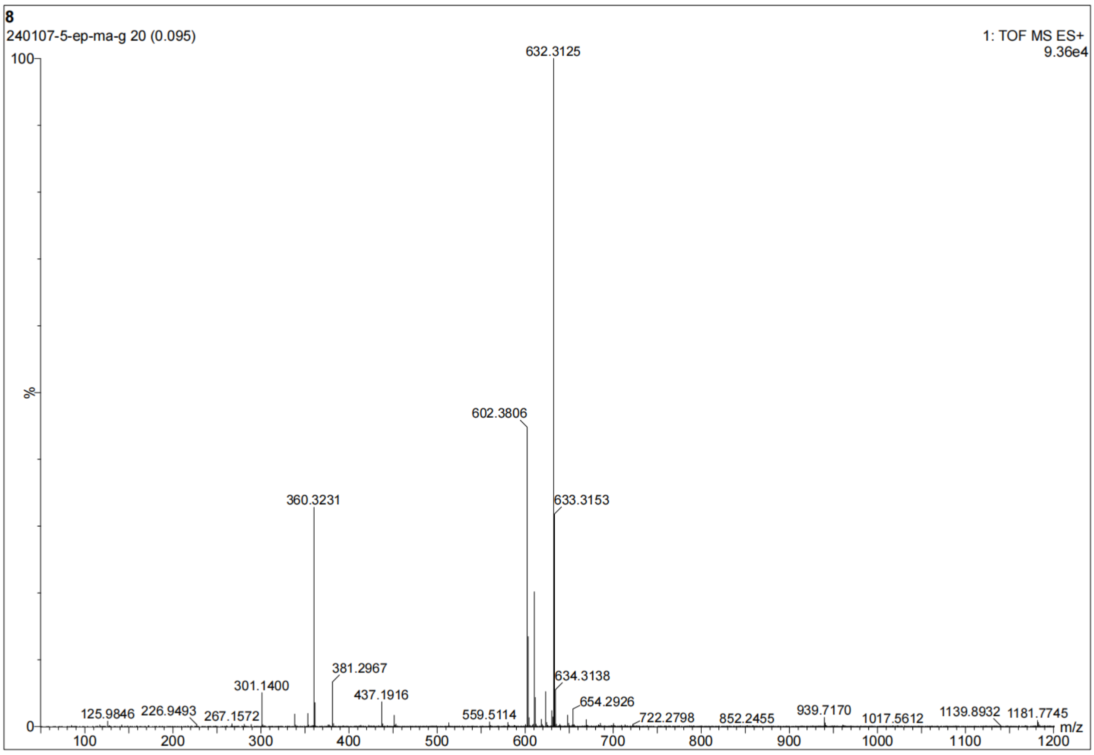

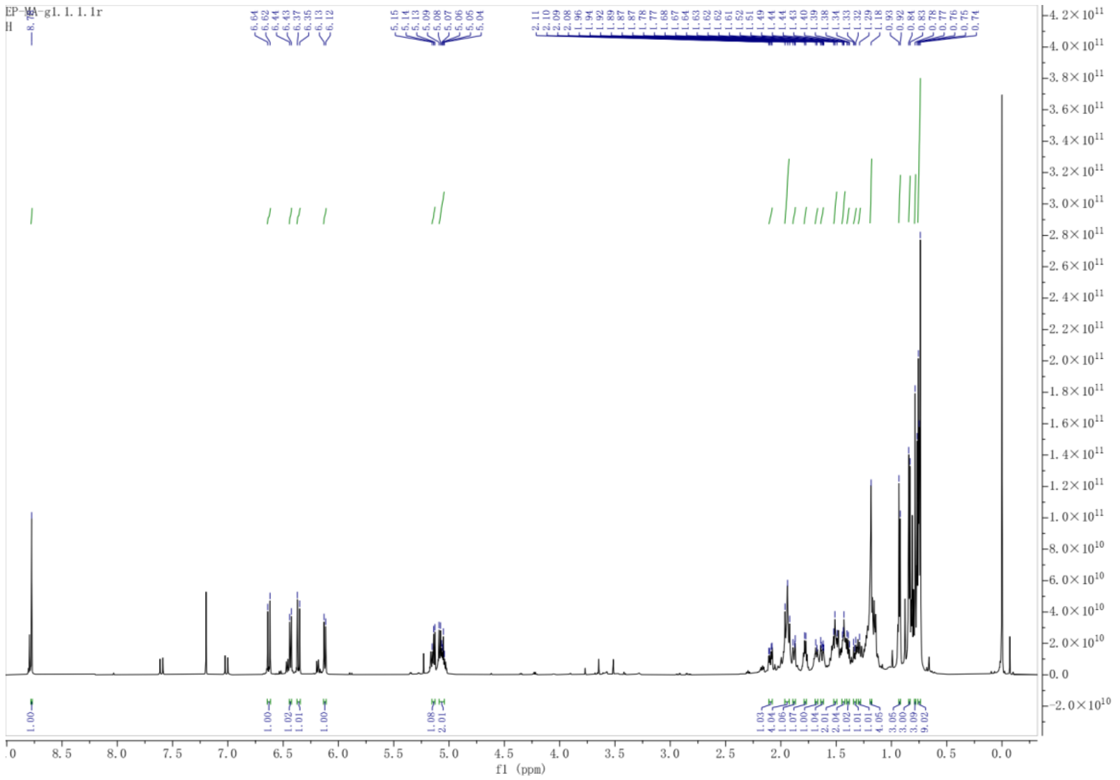
EP-MA-g1.1.1.1r
H
6.64 6.62 6.44 6.43 6.37 6.35 6.13 6.12
5.15 5.14 5.13 5.09 5.08 5.07 5.06 5.05 5.04
2.11 2.10 2.09 2.08 1.96 1.94 1.92 1.89 1.87 1.87 1.78 1.77 1.68 1.67 1.64 1.63 1.62 1.62 1.61 1.52 1.51 1.49 1.44 1.44 1.43 1.43 1.40 1.39 1.38 1.34 1.33 1.32 1.29 1.18 0.93 0.92 0.84 0.83 0.78 0.77 0.76 0.75 0.74
1.00 1.00 1.02 1.01 1.00 1.08 2.01 1.03 4.04 1.06 1.07 1.00 1.04 2.01 2.04 1.02 1.01 1.01 4.05 3.05 3.00 3.09 9.02
f1 (ppm)

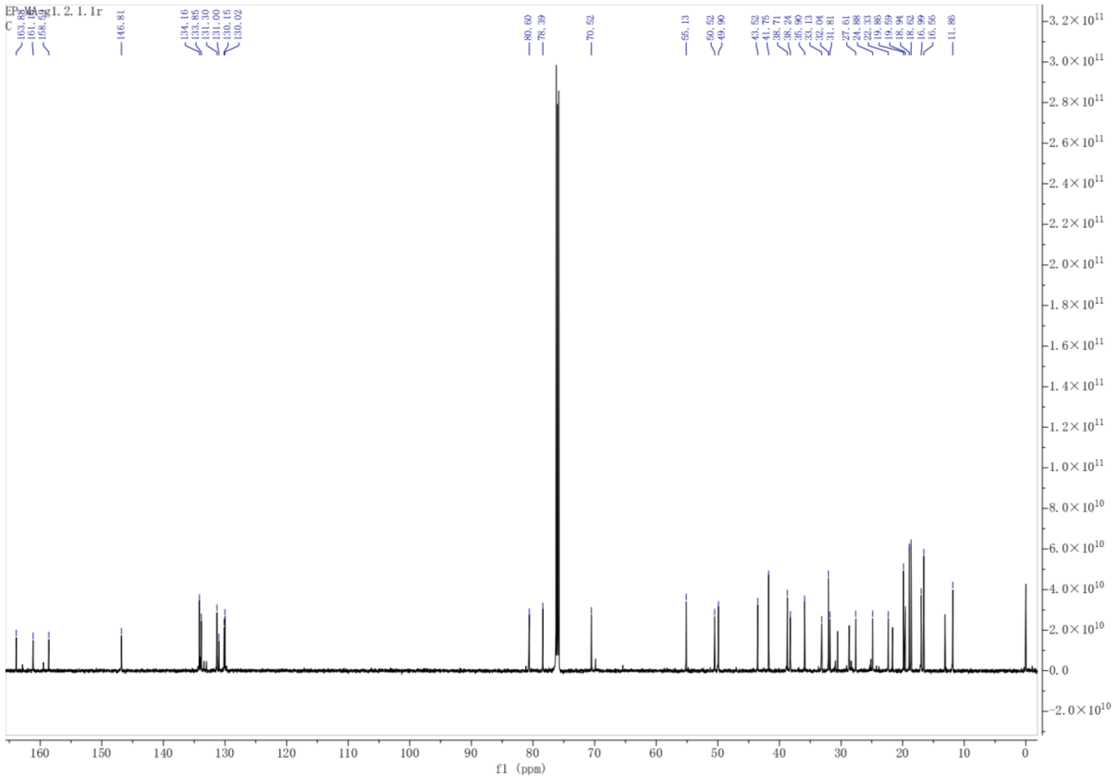
EP-MA-g1.2.1.1r
C
146.81
134.16 133.85 131.30 131.00 130.15 130.02
80.60 78.39 70.52 55.13 50.52 49.90
43.52 41.75 38.71 38.24 35.90 33.13 32.04 31.81 27.61 24.88 22.33 19.86 19.59 18.94 18.62 16.99 16.56 11.86
f1 (ppm)

^{1}H NMR, ^{13}C NMR and HRMS spectra of **1b**

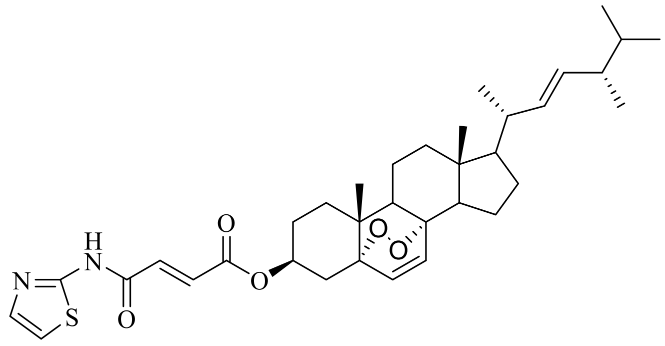

1b

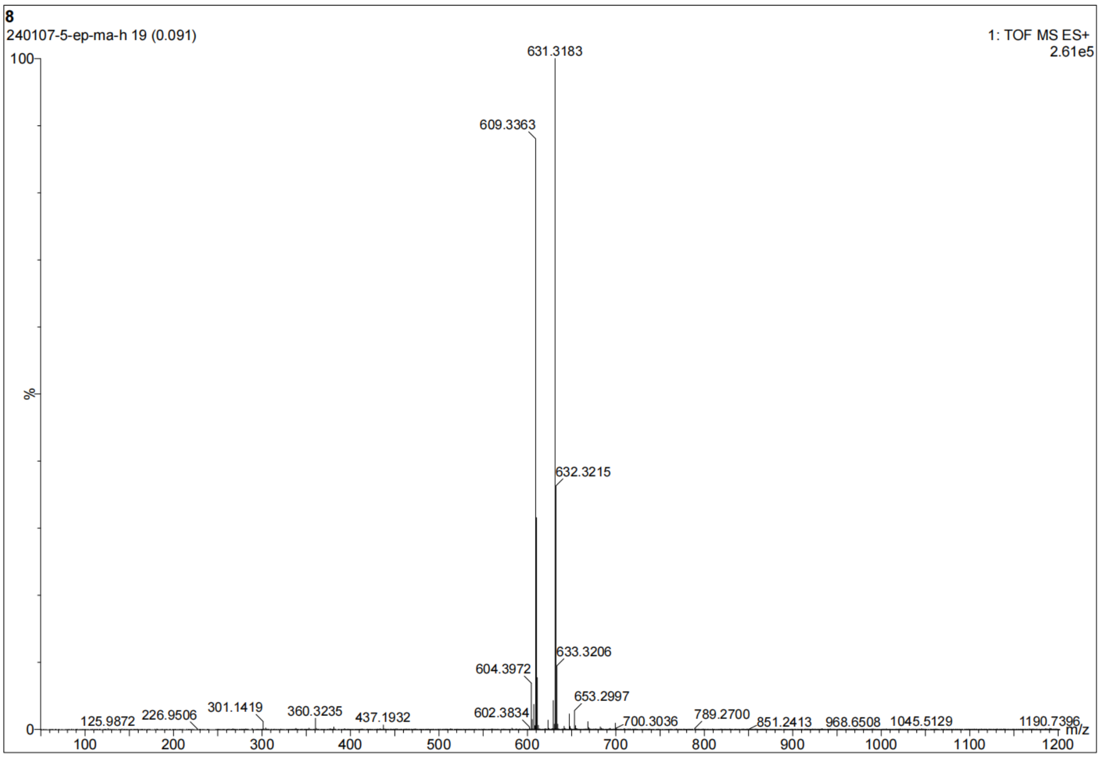

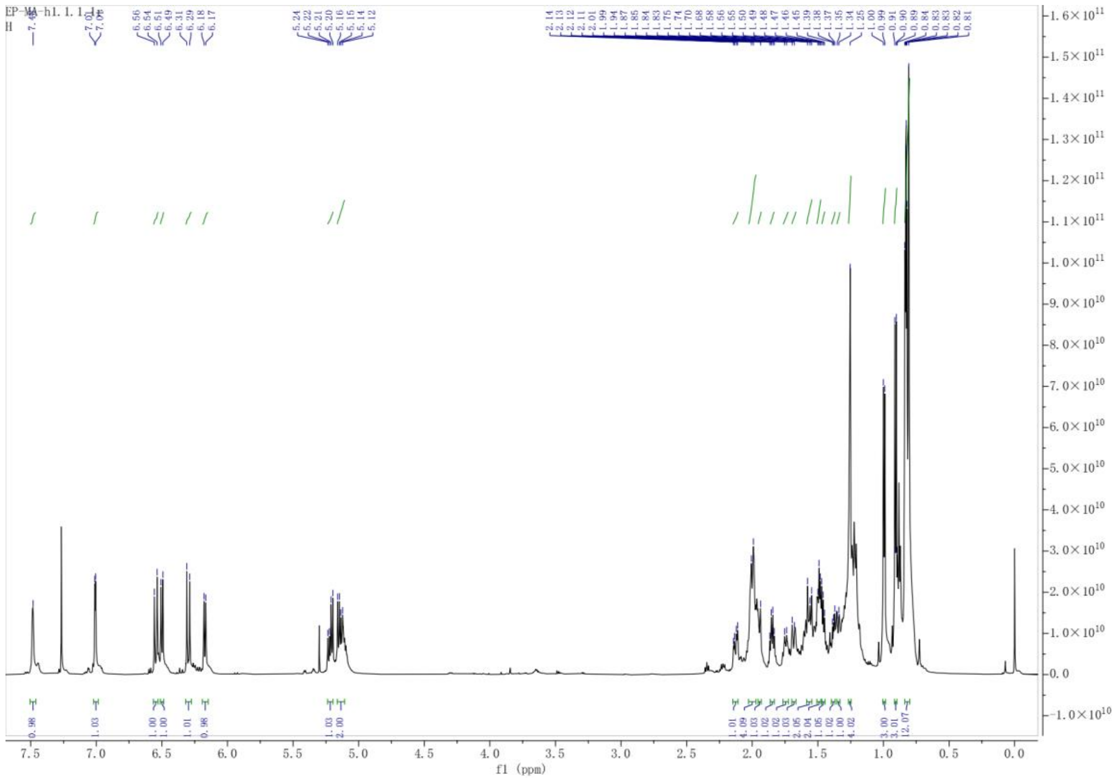

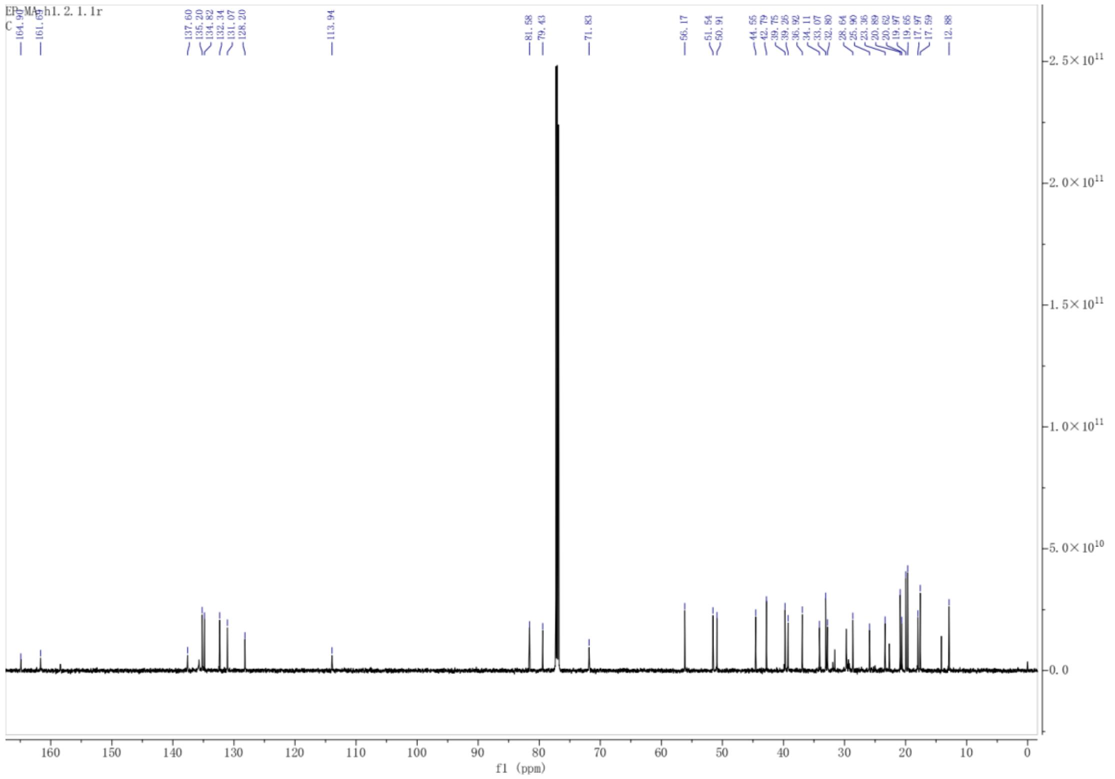

^{1}H NMR, ^{13}C NMR and HRMS spectra of **1c**

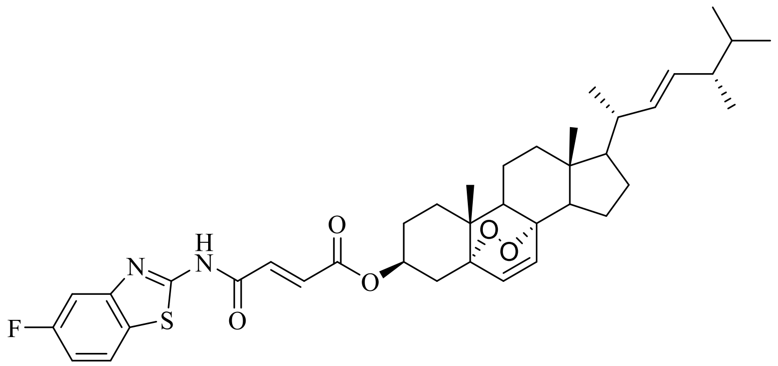

1c

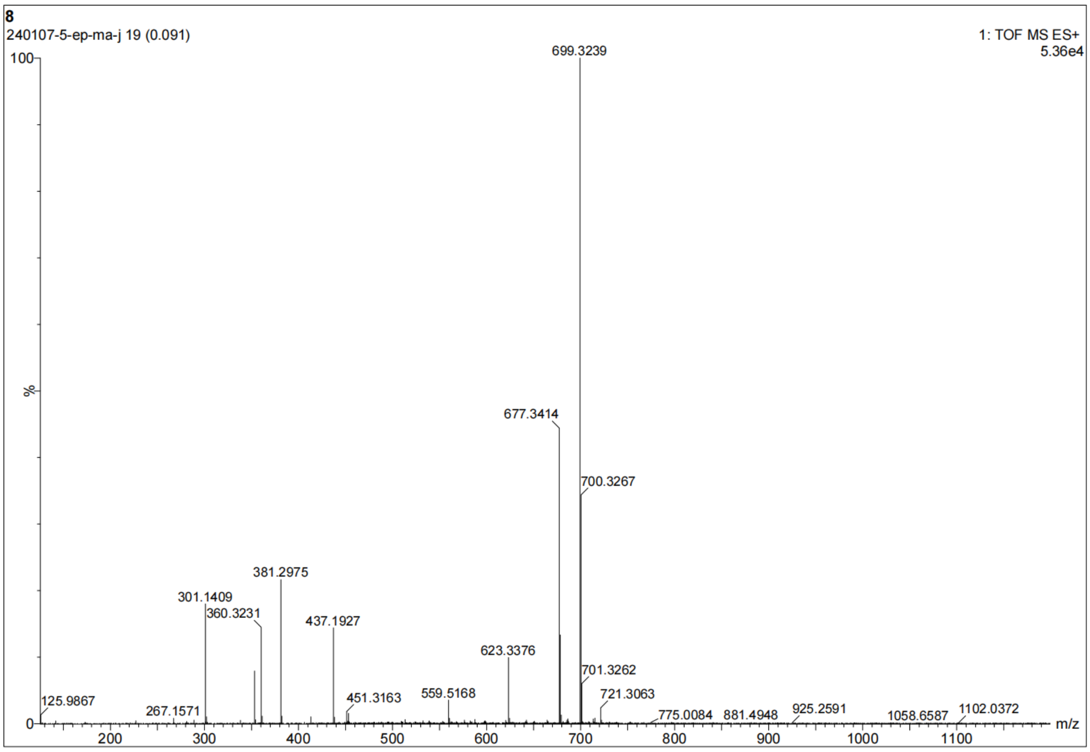

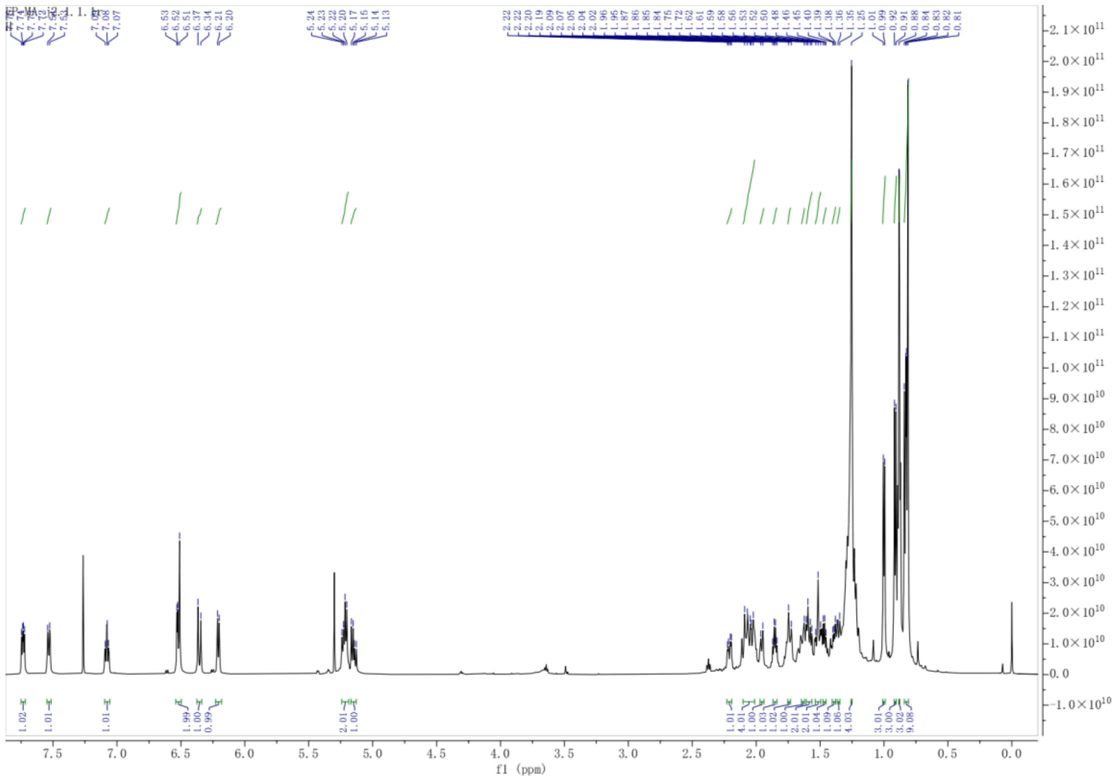
f1 (ppm)

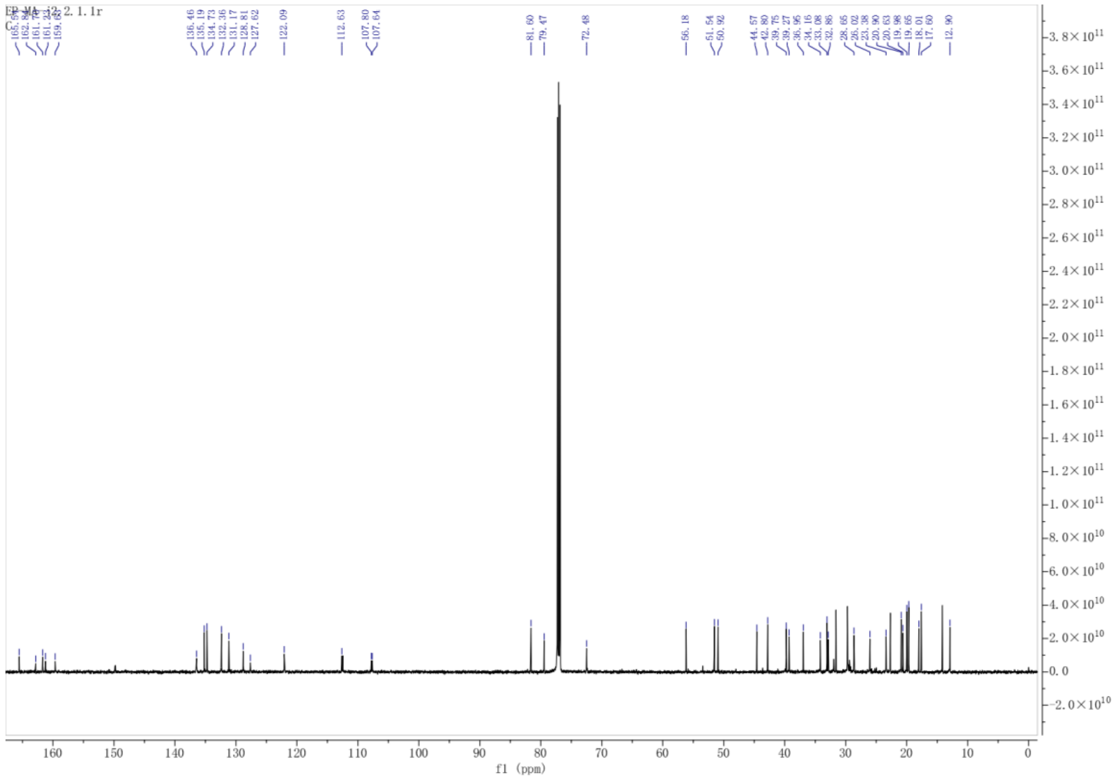
f1 (ppm)

^{1}H NMR, ^{13}C NMR and HRMS spectra of **1d**

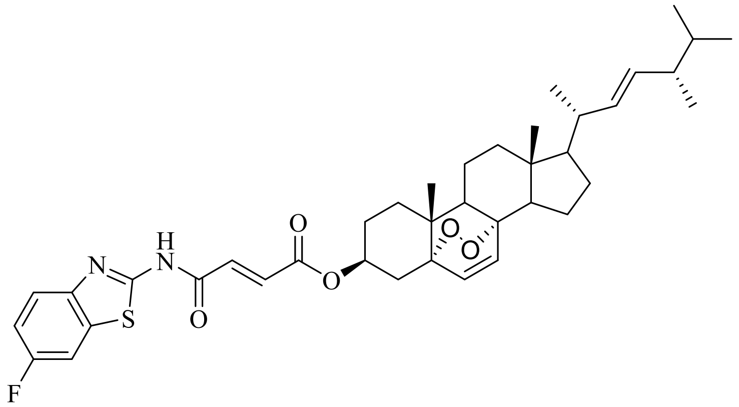

1d

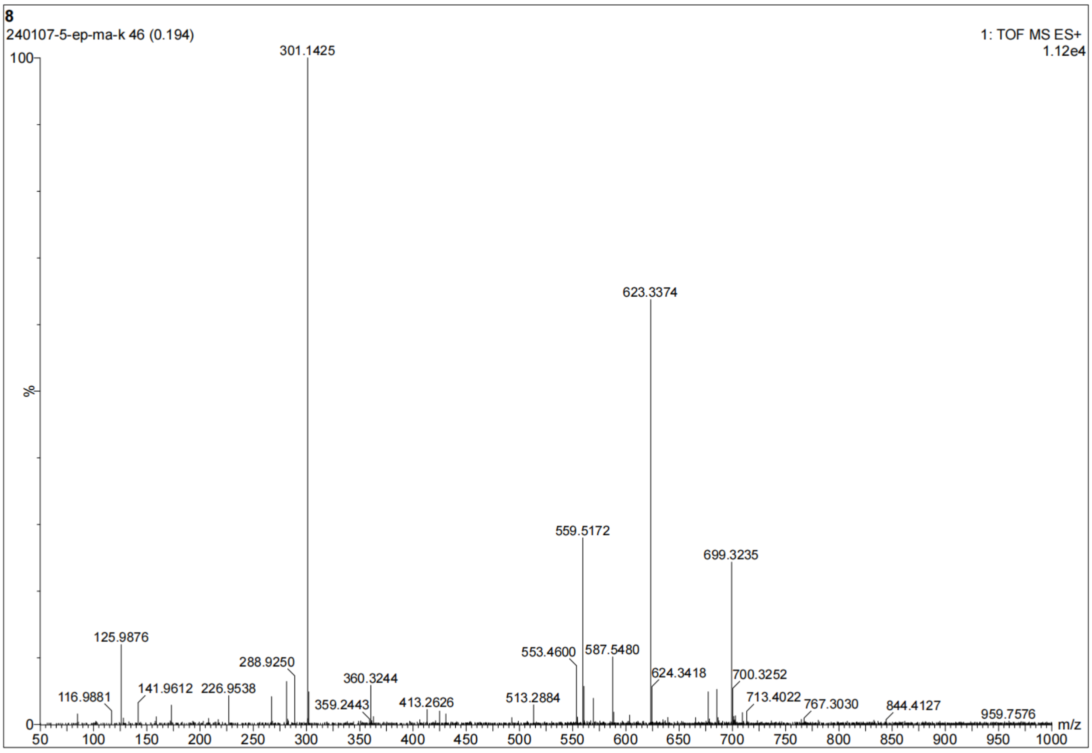

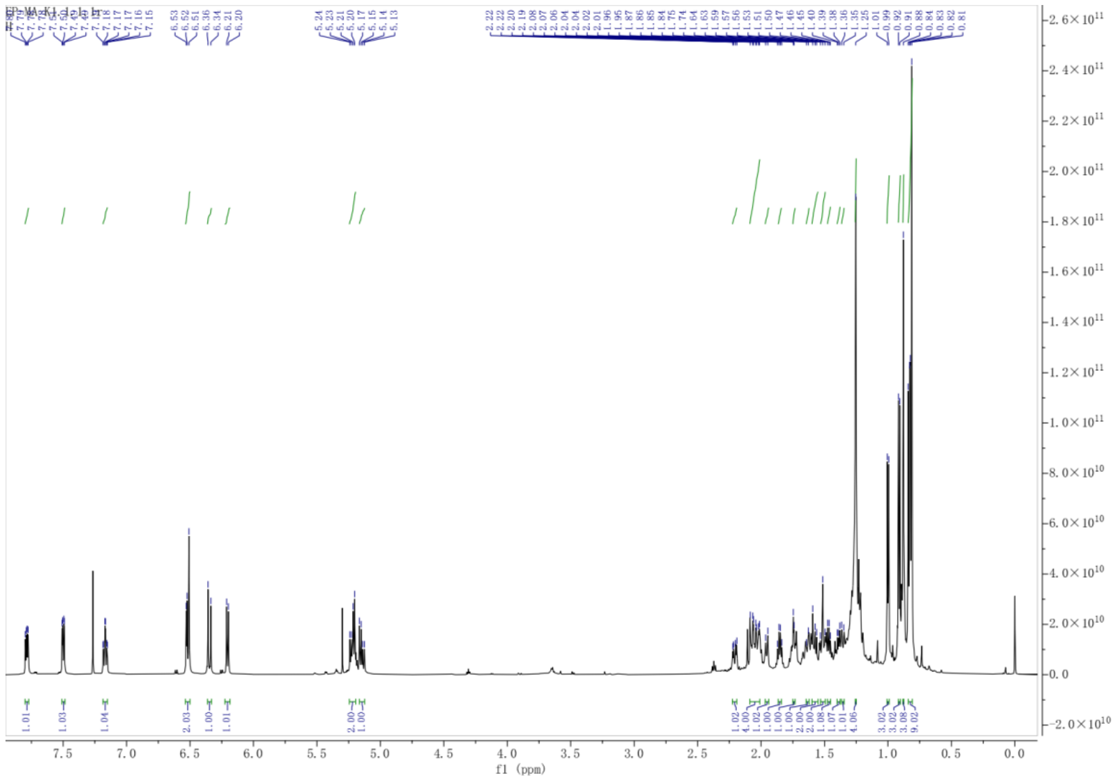

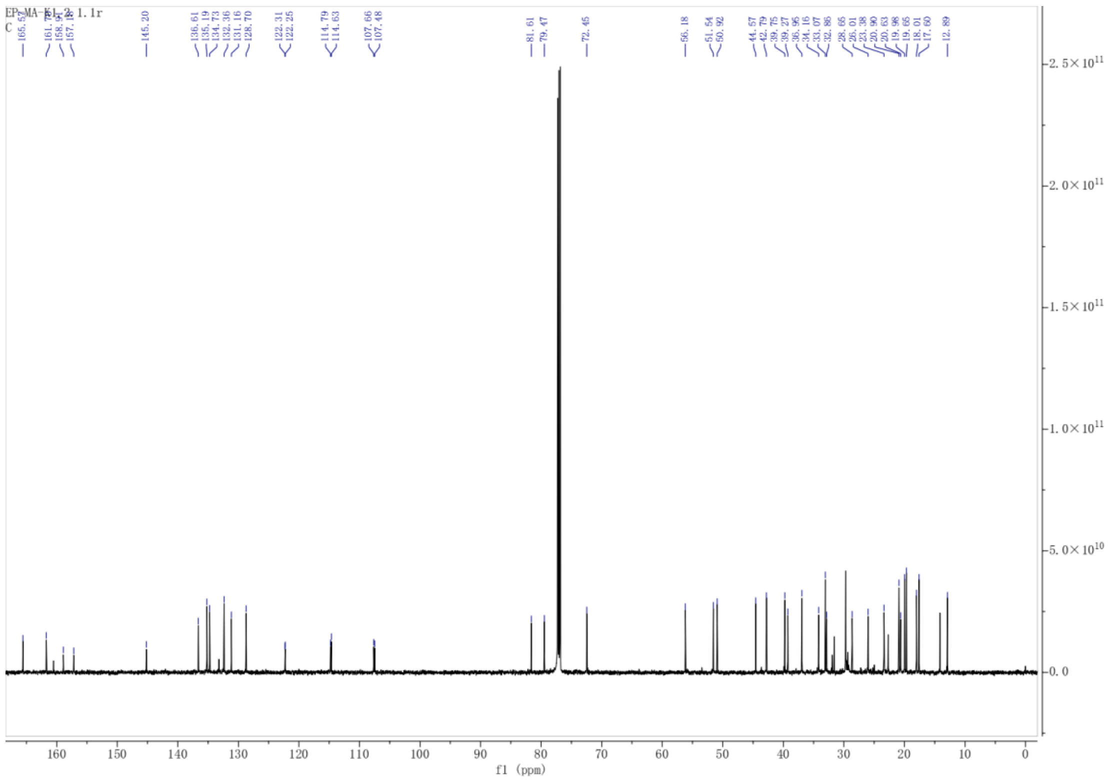

^{1}H NMR, ^{13}C NMR and HRMS spectra of **1e**

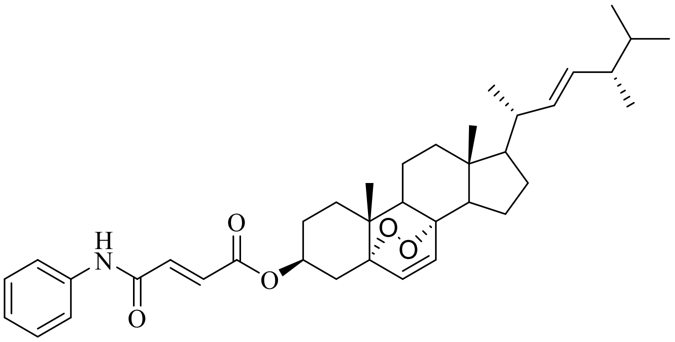

1e

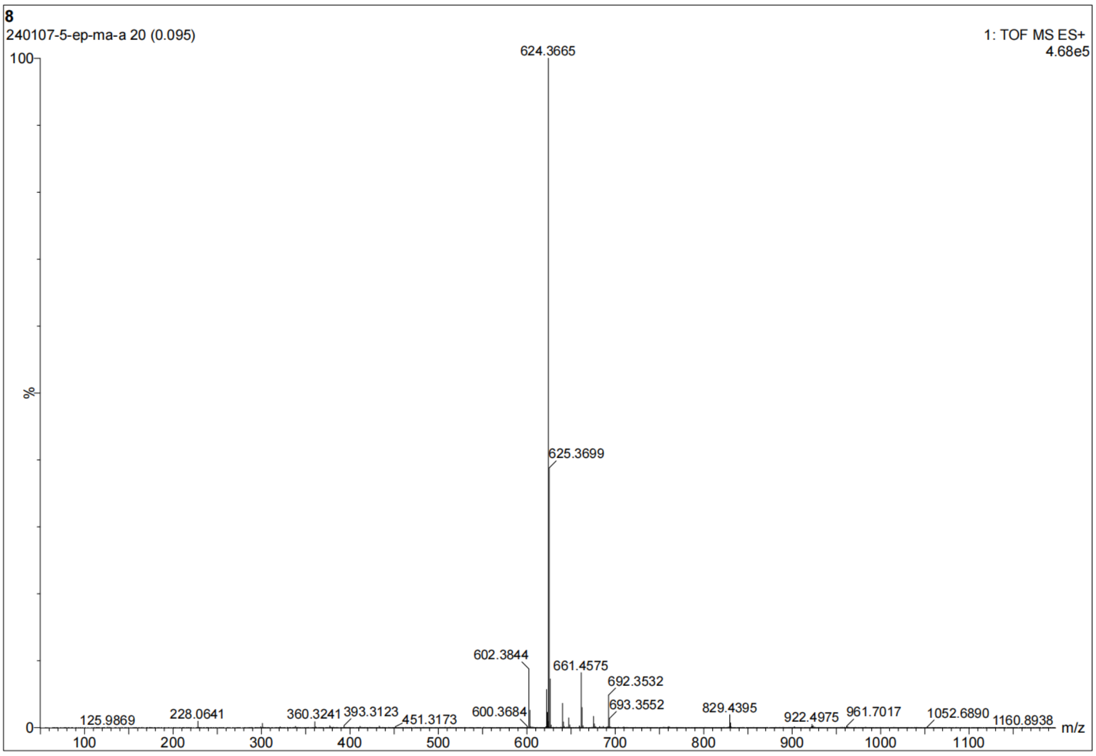

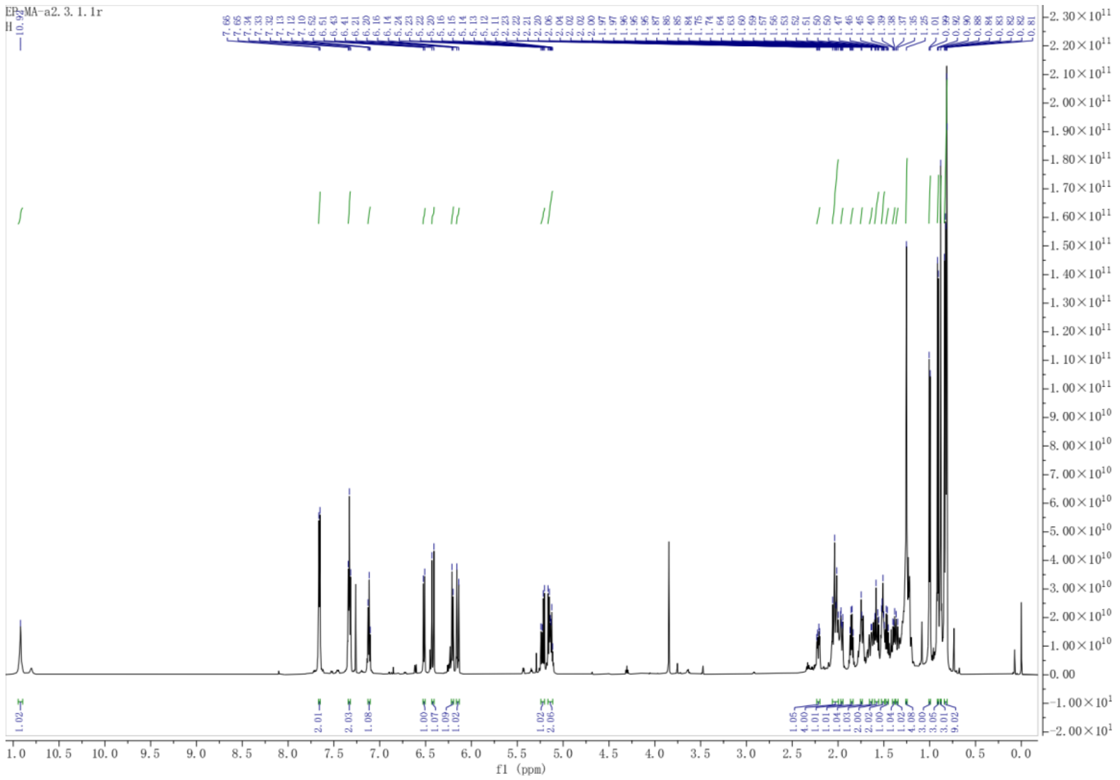
EP-MA-a2.3.1.1r
H
10.92
7.66 7.65 7.34 7.33 7.32 7.13 7.12 7.10 6.52 6.51 6.43 6.41 6.21 6.20 6.16 6.14 5.24 5.23 5.22 5.20 5.16 5.15 5.14 5.13 5.12 5.11 2.23 2.22 2.21 2.20 2.06 2.04 2.02 2.02 2.00 1.97 1.96 1.95 1.95 1.87 1.86 1.85 1.84 1.75 1.74 1.64 1.63 1.60 1.59 1.57 1.56 1.53 1.52 1.51 1.50 1.50 1.47 1.46 1.45 1.40 1.39 1.38 1.37 1.35 1.25 1.01 0.99 0.92 0.90 0.88 0.84 0.83 0.82 0.81
1.02 2.01 2.03 1.08 1.00 1.07 1.09 1.02 1.02 2.06 1.05 4.00 1.01 1.01 1.04 1.03 2.00 2.02 1.00 1.04 1.02 4.08 3.00 3.05 3.01 9.02
f1 (ppm)

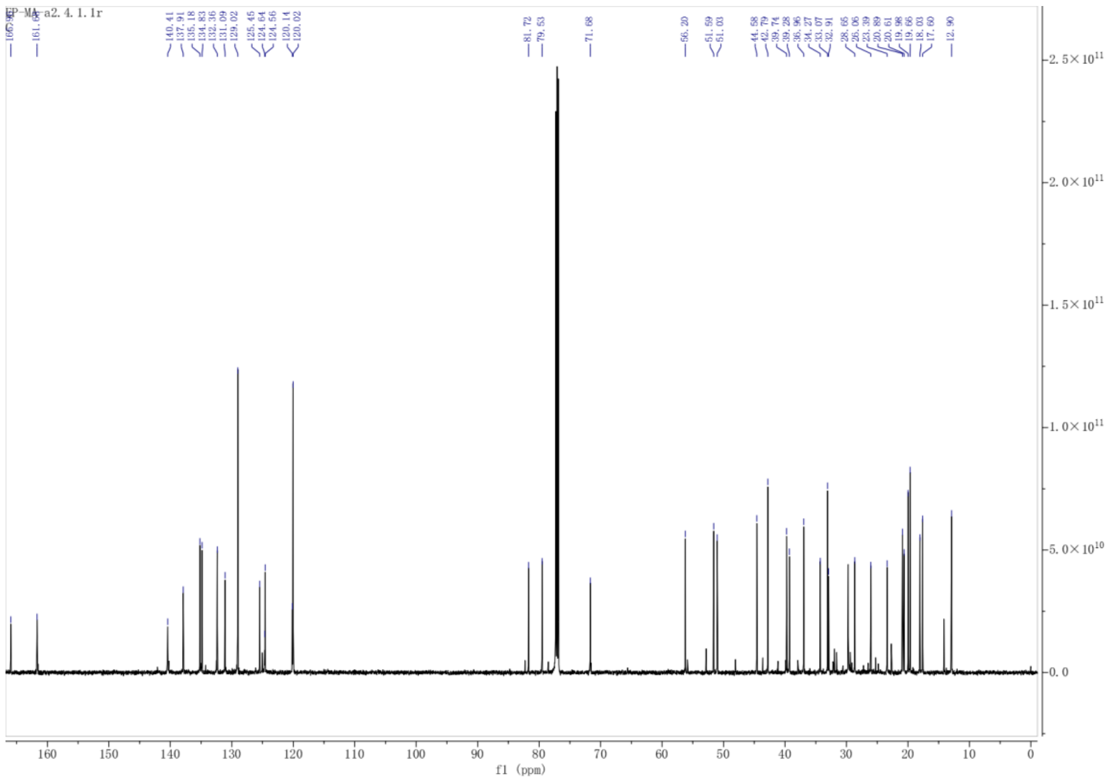
EP-MA-a2.4.1.1r
161.87
140.41 137.91 135.18 134.83 132.36 131.09 129.02 125.45 124.64 124.56 120.14 120.02
81.72 79.53
71.68
56.20
51.59 51.03
44.58 42.79 39.74 39.28 36.96 34.27 33.07 32.91 28.65 26.06 23.39 20.89 20.61 19.98 19.66 18.03 17.60 12.90
f1 (ppm)

^{1}H NMR, ^{13}C NMR and HRMS spectra of **1f**

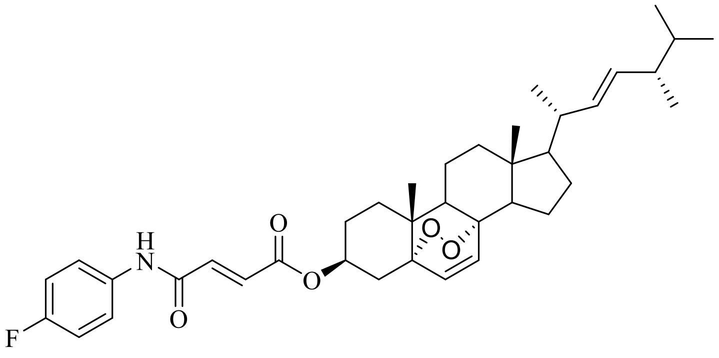

1f

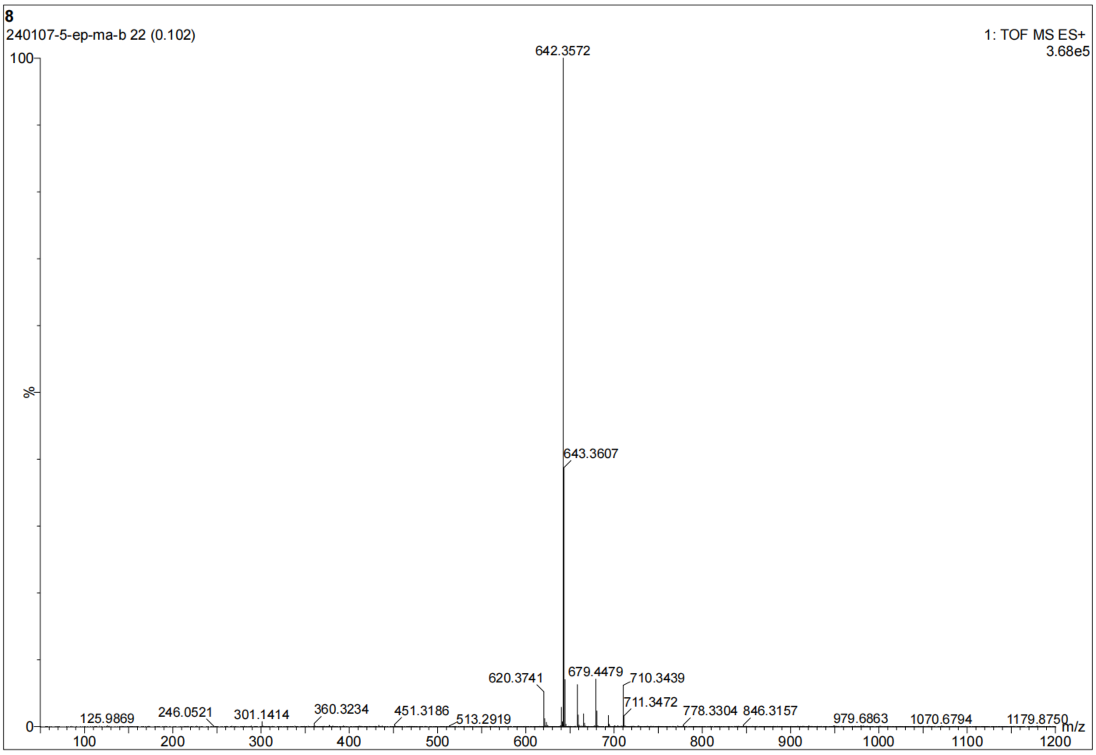

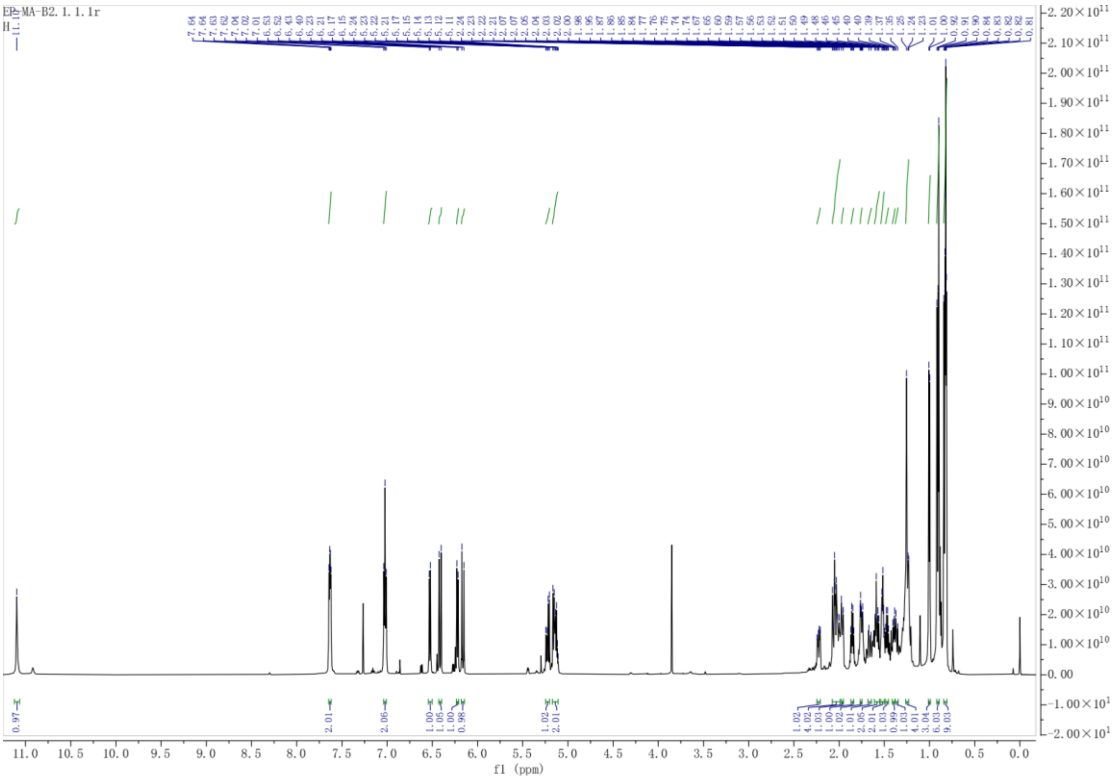
EP-MA-B2.1.1.1r
H
11.10
7.64 7.64 7.63 7.62 7.04 7.02 7.01 6.53 6.52 6.43 6.40 6.23 6.21 6.17 6.15 5.24 5.23 5.22 5.21 5.17 5.15 5.14 5.13 5.12 5.11 2.24 2.23 2.22 2.21 2.07 2.05 2.04 2.03 2.02 2.00 1.98 1.96 1.87 1.86 1.85 1.84 1.77 1.76 1.75 1.74 1.67 1.65 1.60 1.59 1.57 1.56 1.53 1.52 1.51 1.50 1.49 1.48 1.46 1.45 1.40 1.40 1.39 1.37 1.35 1.25 1.24 1.23 1.01 1.00 0.92 0.91 0.90 0.84 0.83 0.82 0.81
f1 (ppm)

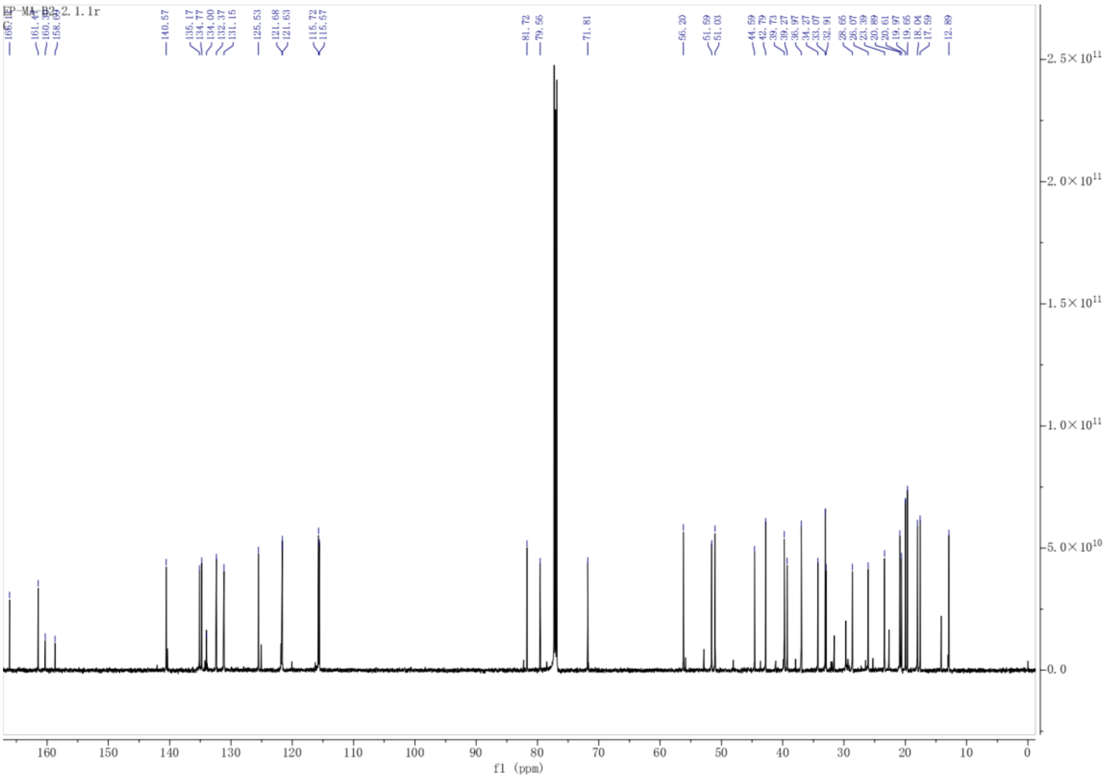
EP-MA-B2.2.1.1r
C
140.57
135.17 134.77 134.00 132.37 131.15
125.53 121.68 121.63 115.72 115.57
81.72 79.56 71.81
56.20 51.59 51.03
44.59 42.79 39.73 39.27 36.97 34.27 33.07 32.91 28.65 26.07 23.39 20.89 20.61 19.97 19.65 18.04 17.59 12.89
f1 (ppm)

^{1}H NMR, ^{13}C NMR and HRMS spectra of **1g**

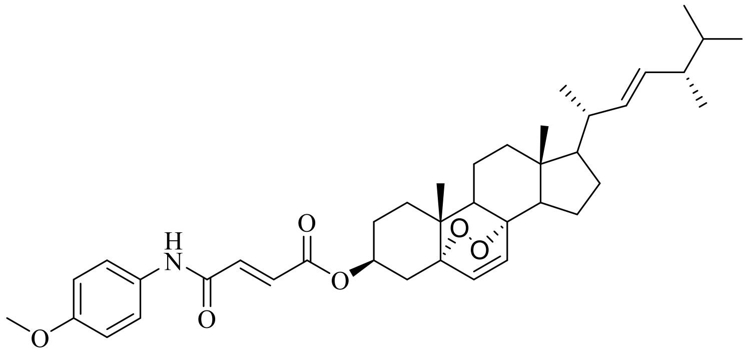

1g

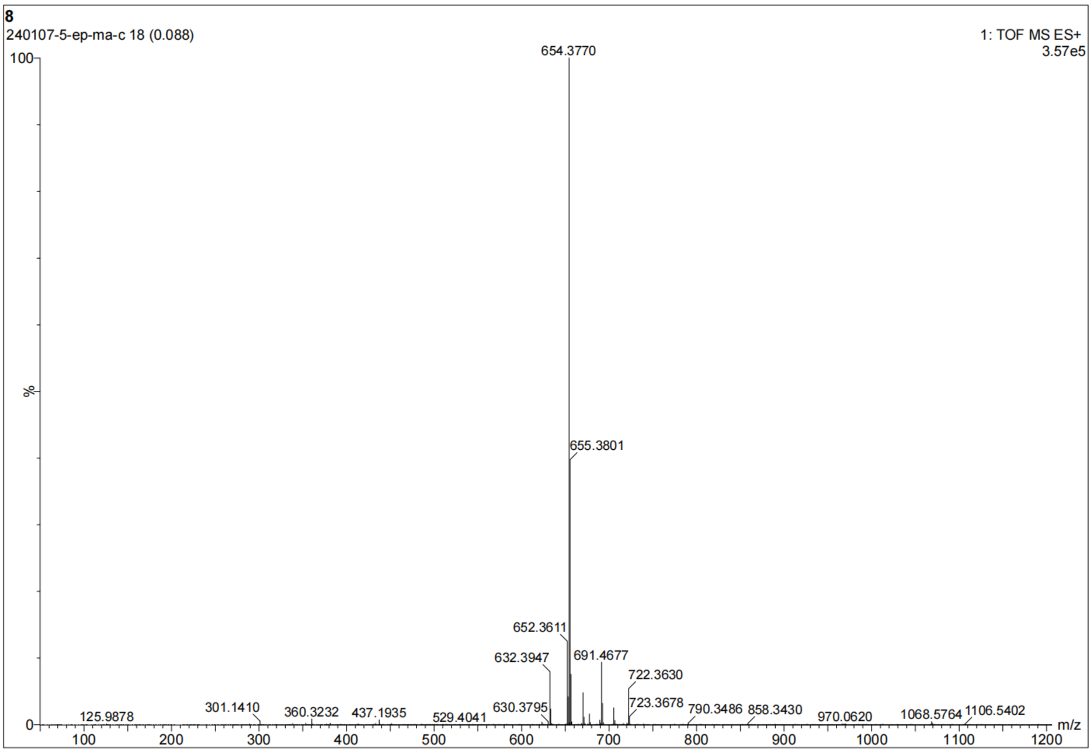

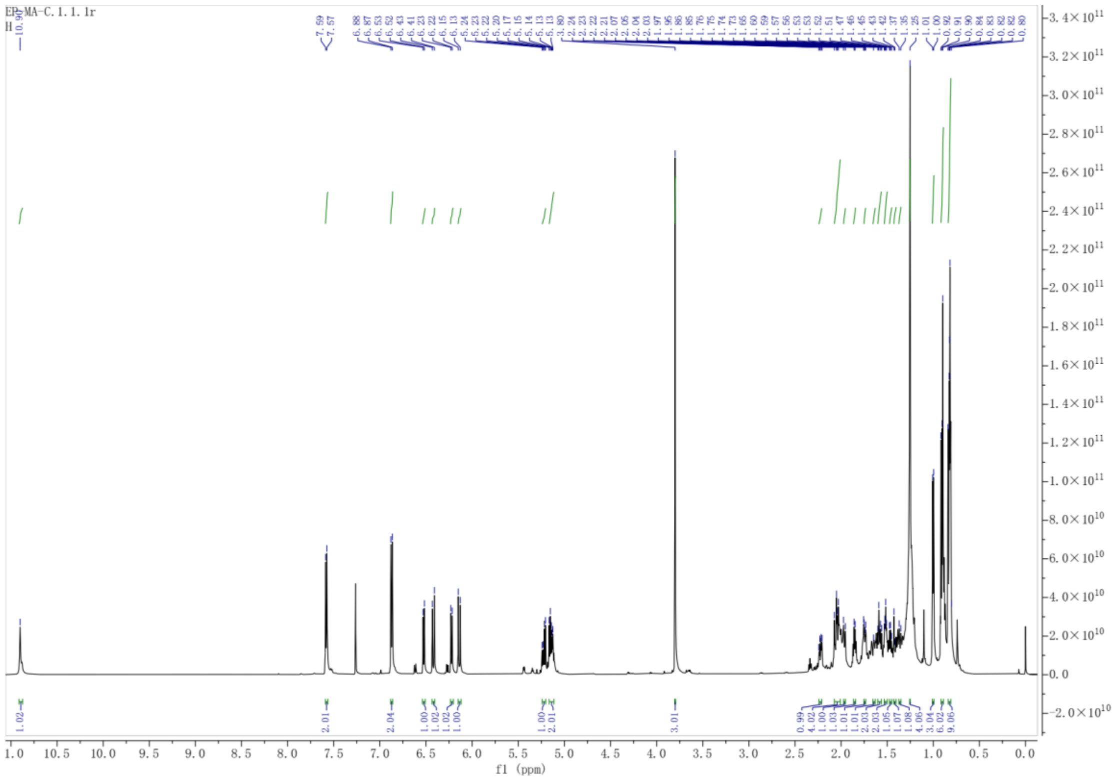

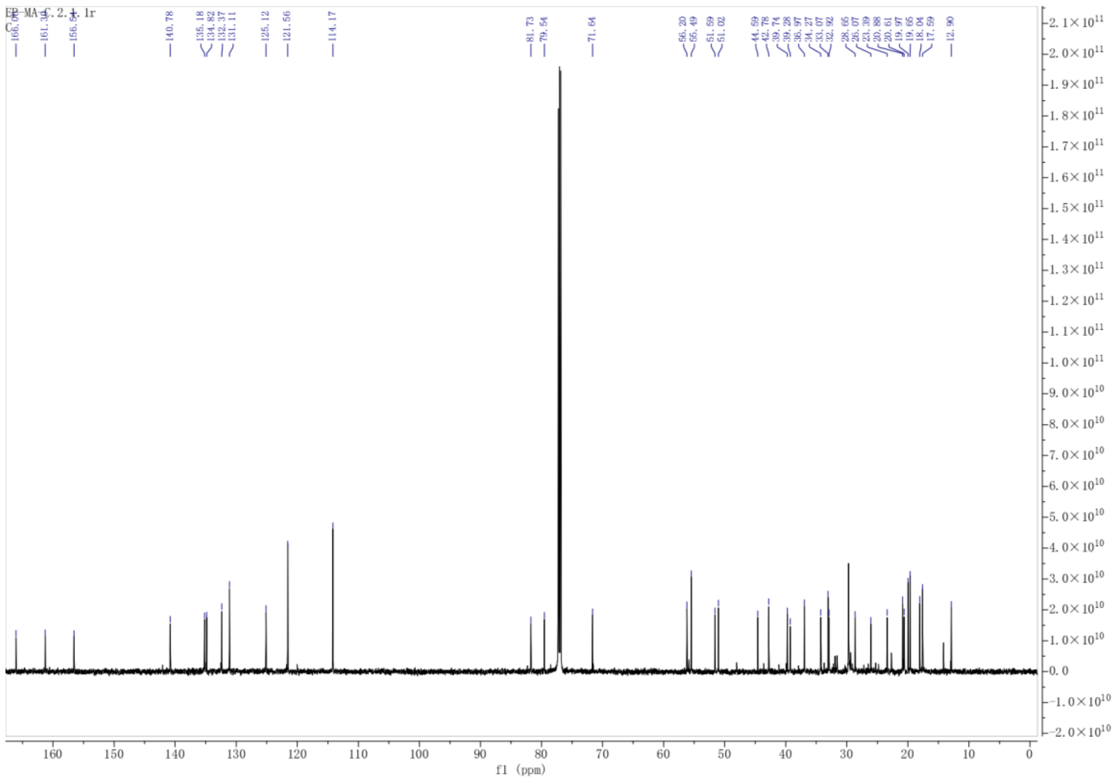

^{1}H NMR, ^{13}C NMR and HRMS spectra of **1h**

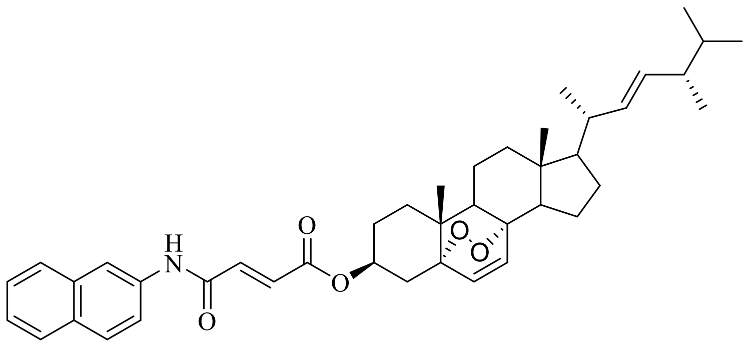

1h

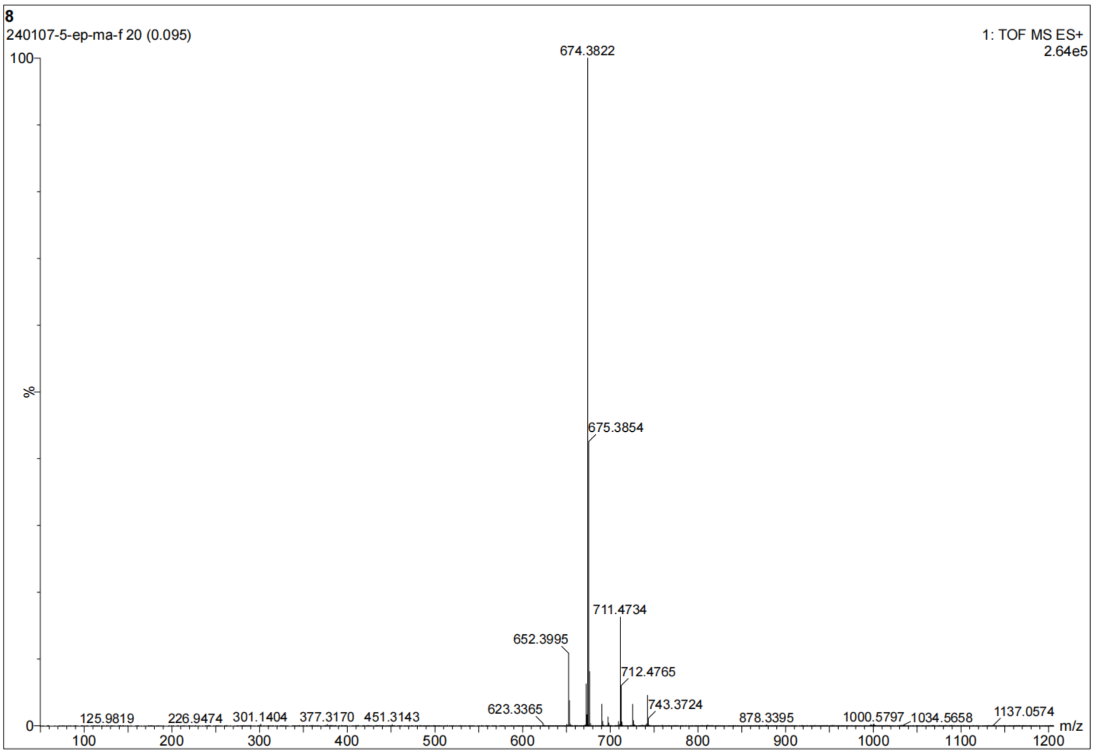

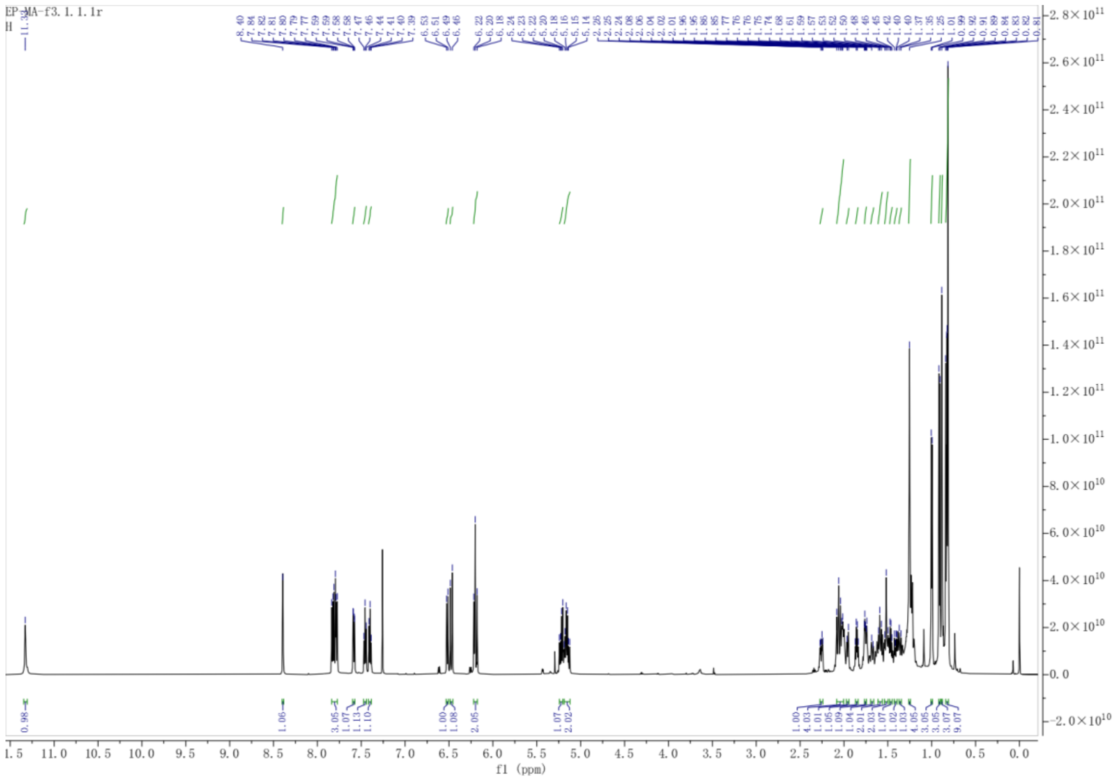
EP-MA-f3.1.1.1r
H
f1 (ppm)

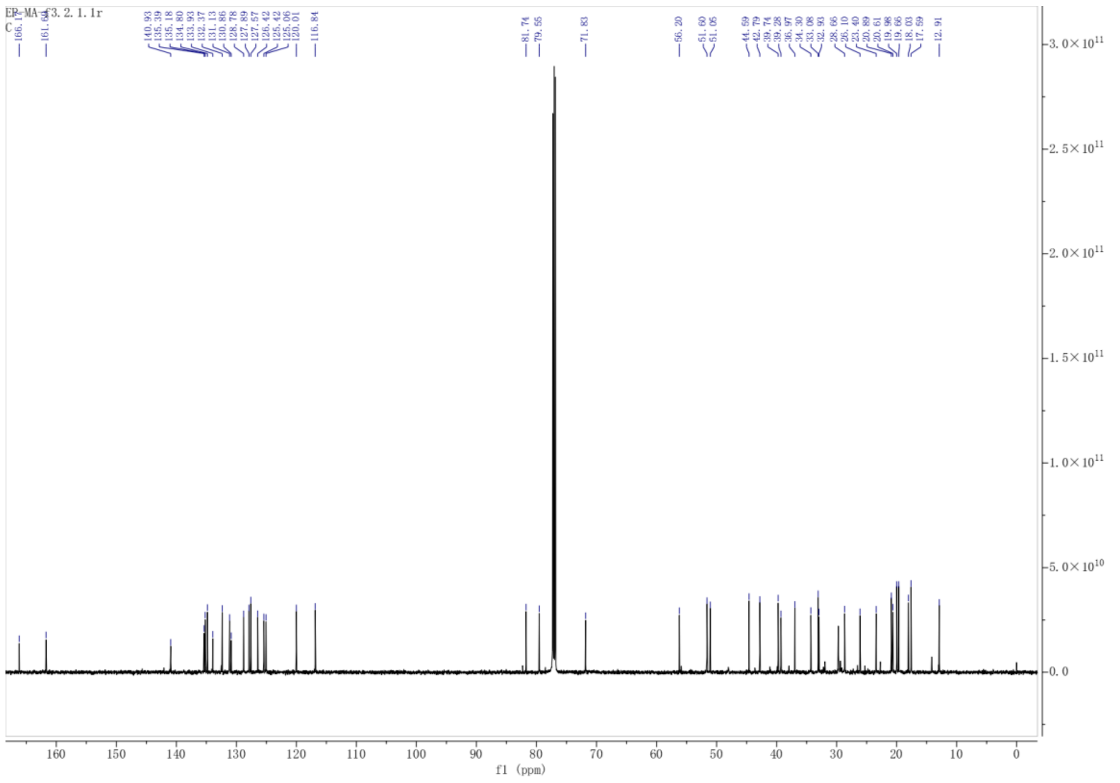
EP-MA-f3.2.1.1r
C
f1 (ppm)

^{1}H NMR and ^{13}C NMR of **2**

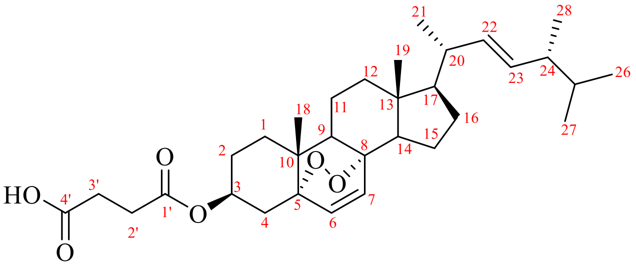

2

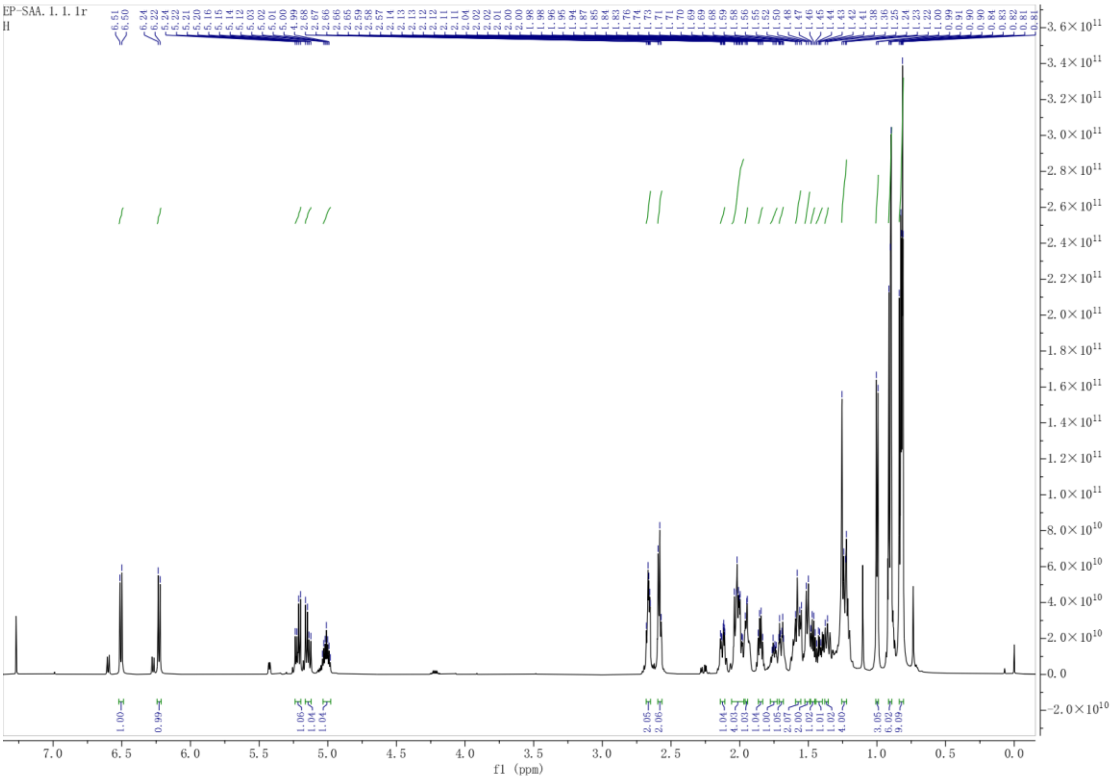

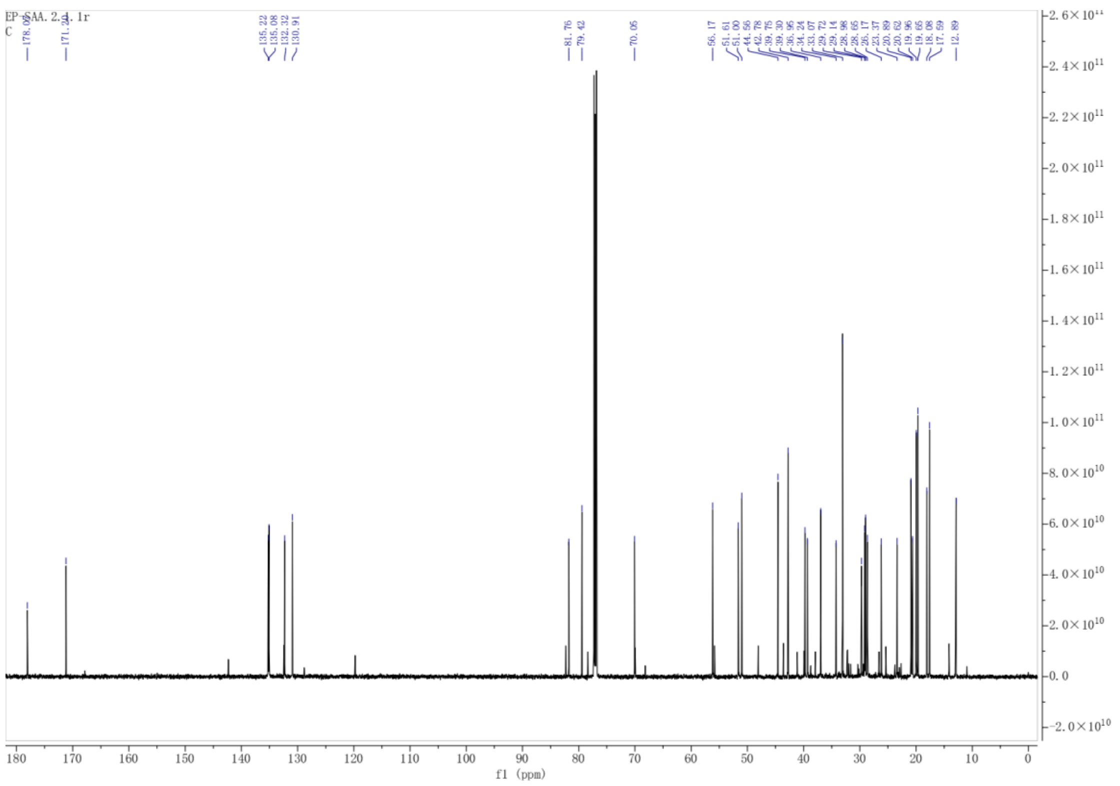
C
178.0
171.2
135.22
135.08
132.32
130.91
81.76
79.42
70.05
56.17
51.61
51.00
44.56
42.78
39.75
39.30
36.95
34.24
33.07
29.72
29.14
28.98
28.65
26.17
23.37
20.89
20.62
19.96
19.65
18.08
17.59
12.89
f1 (ppm)

^{1}H NMR, ^{13}C NMR and HRMS spectra of **2a**

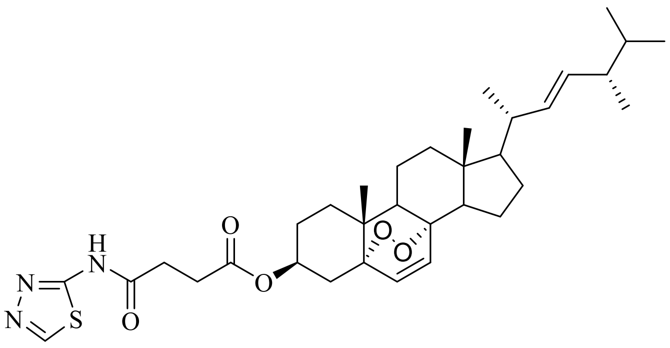

2a

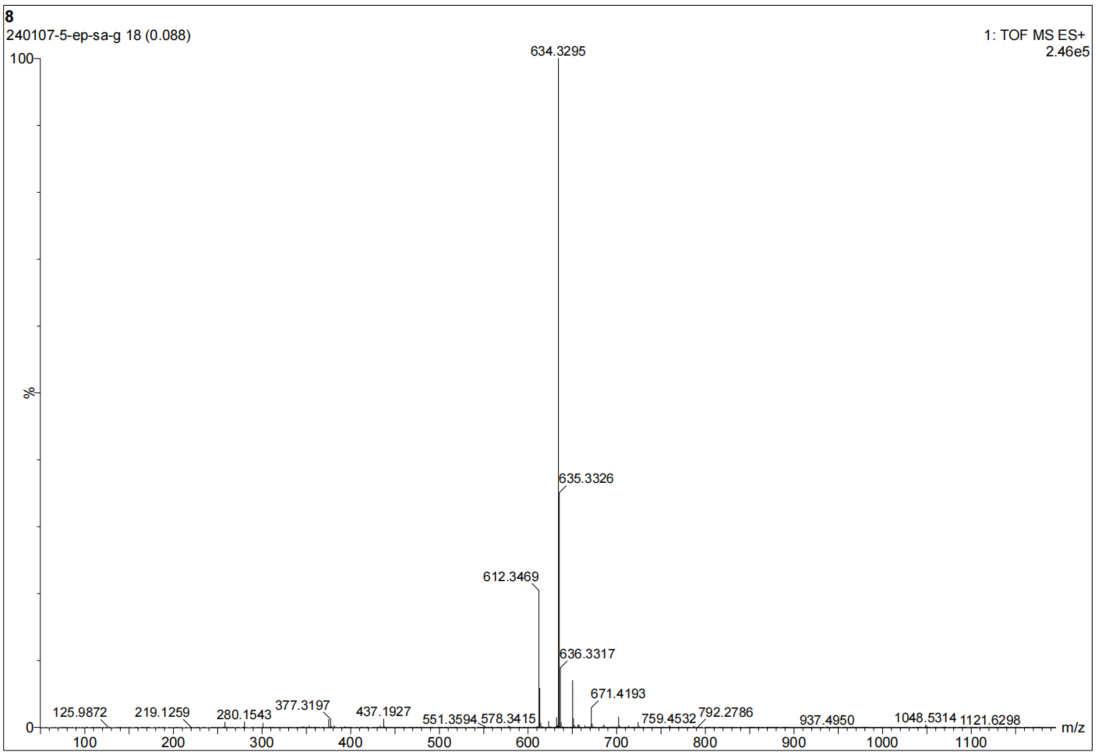

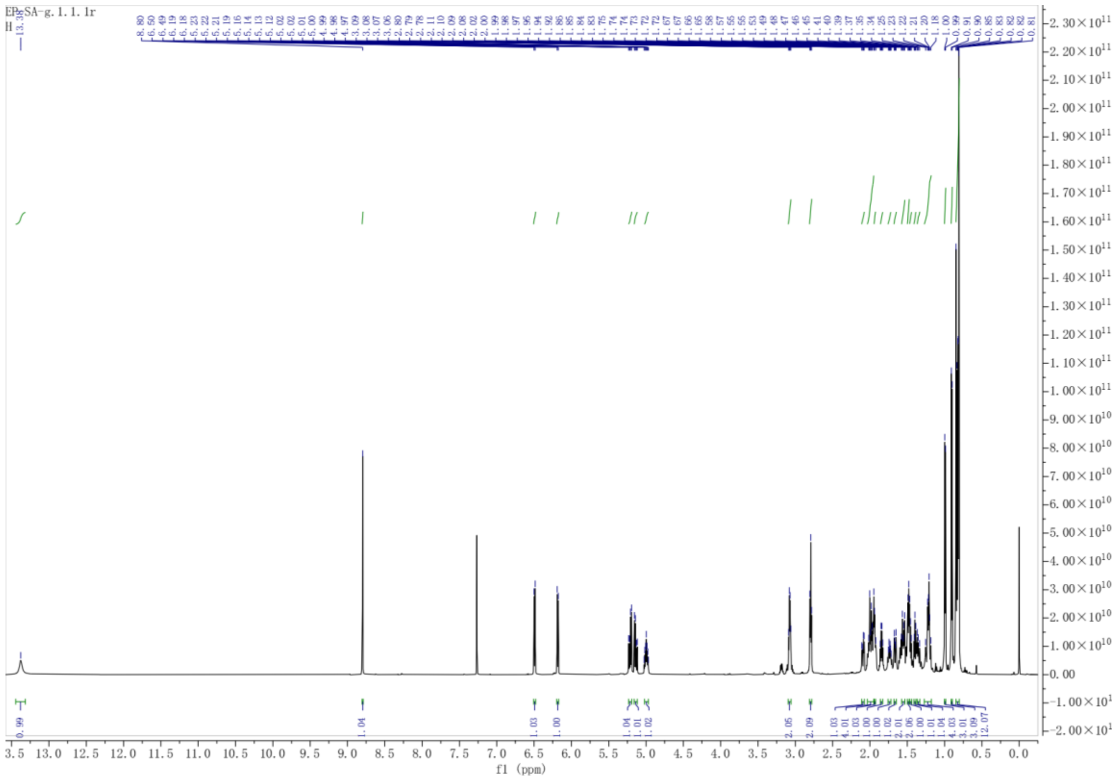
EP-SA-g.1.1.1r
H
f1 (ppm)

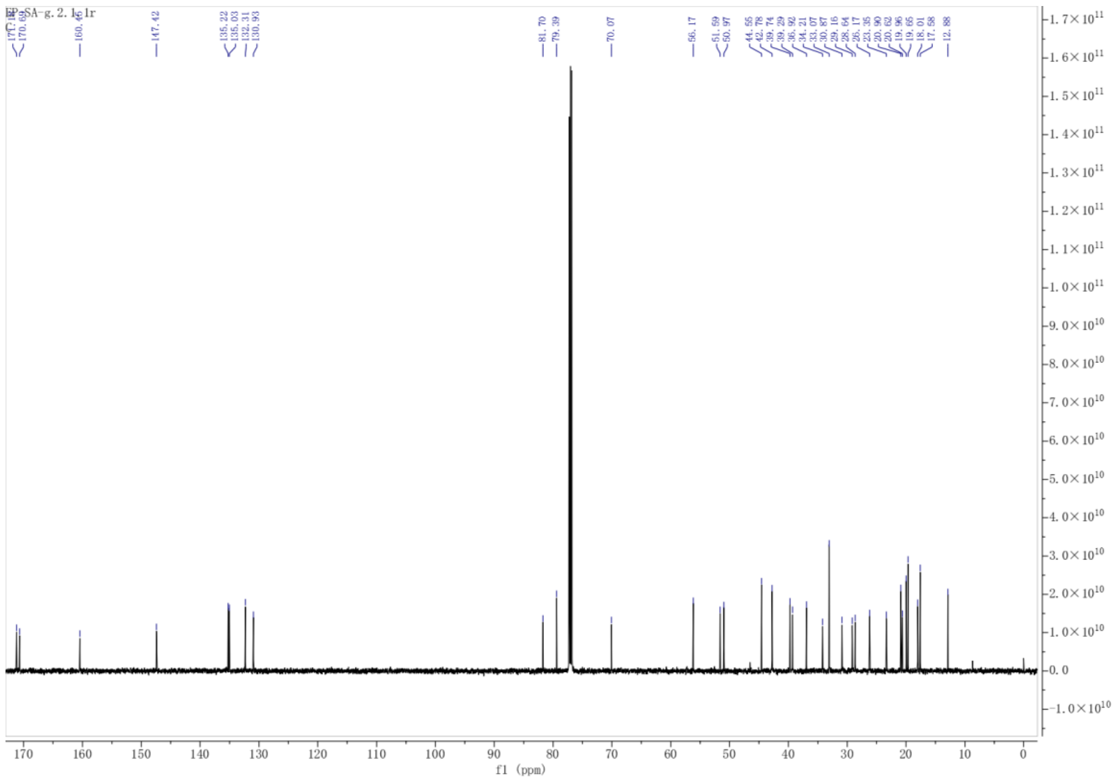
EP-SA-g.2.1.1r
C
f1 (ppm)

^{1}H NMR, ^{13}C NMR and HRMS spectra of **2b**

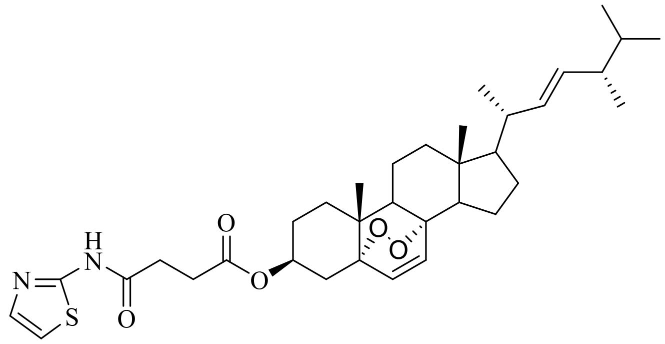

2b

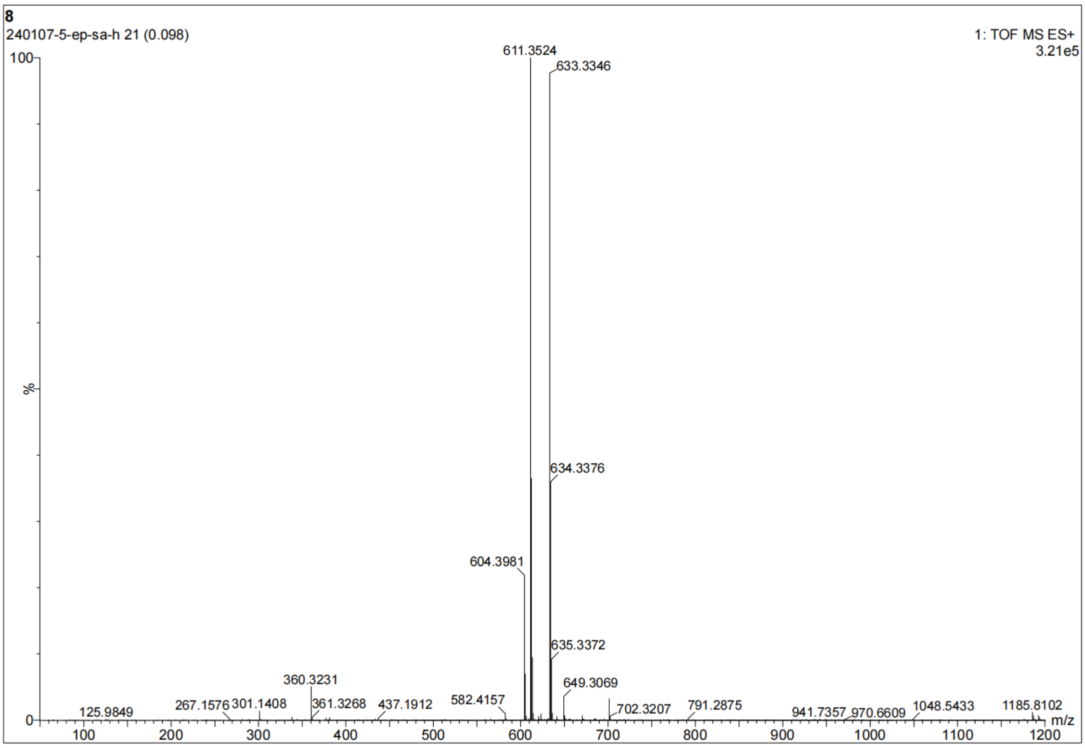

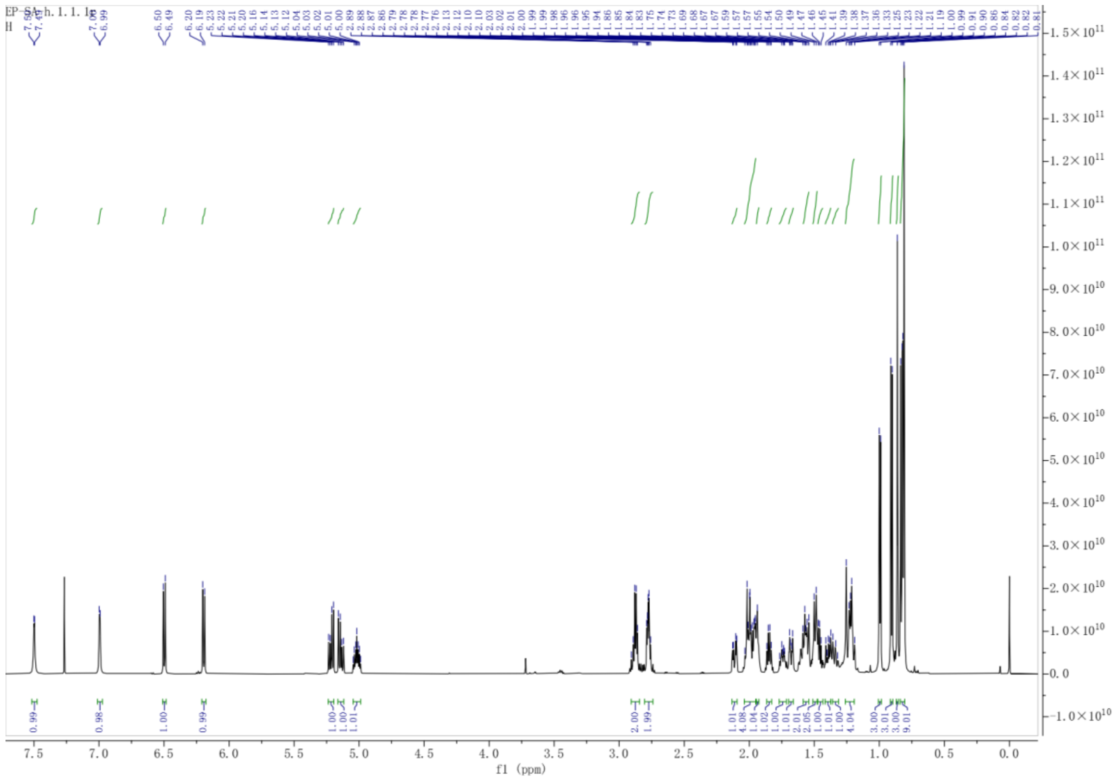

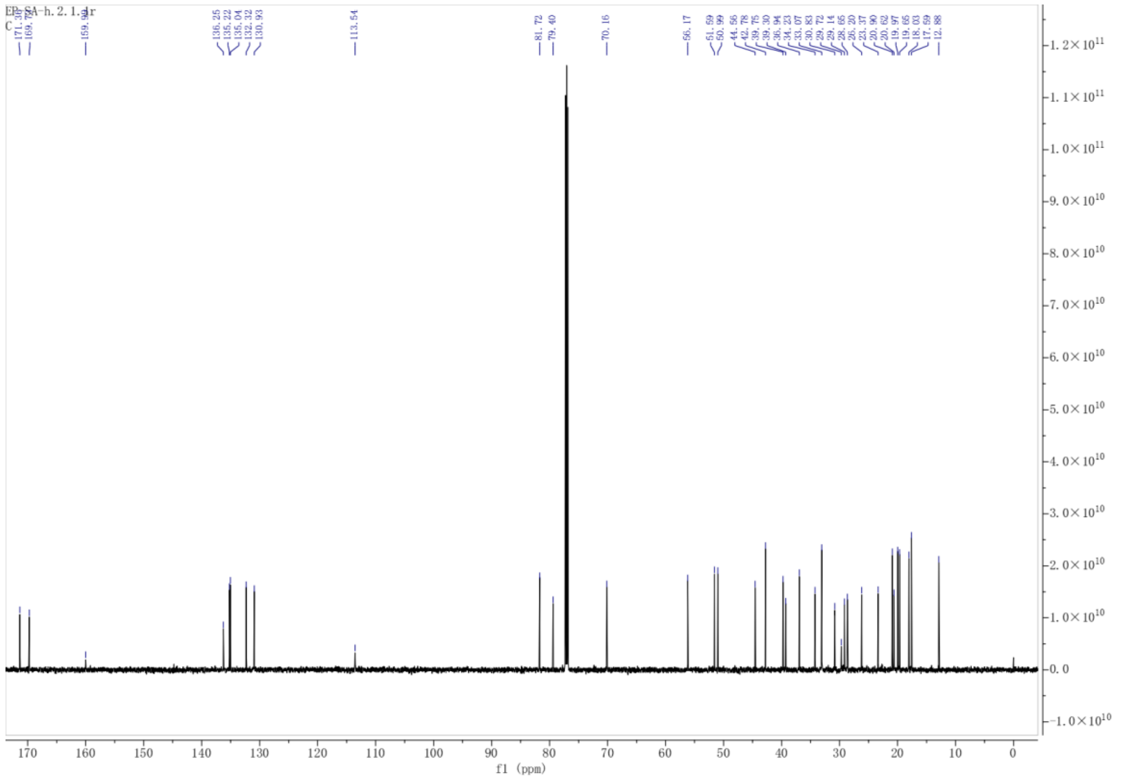

^{1}H NMR, ^{13}C NMR and HRMS spectra of **2c**

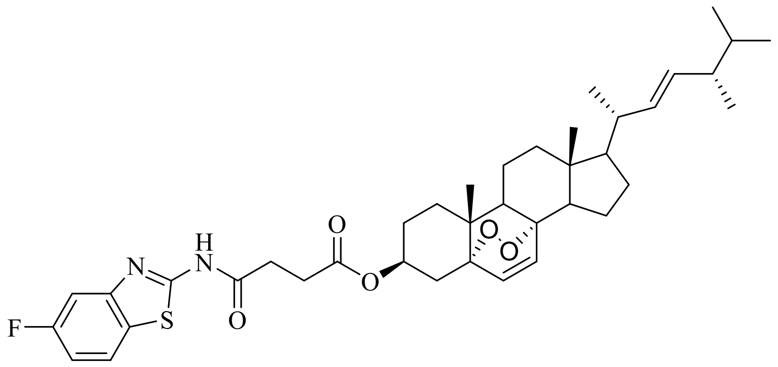

2c

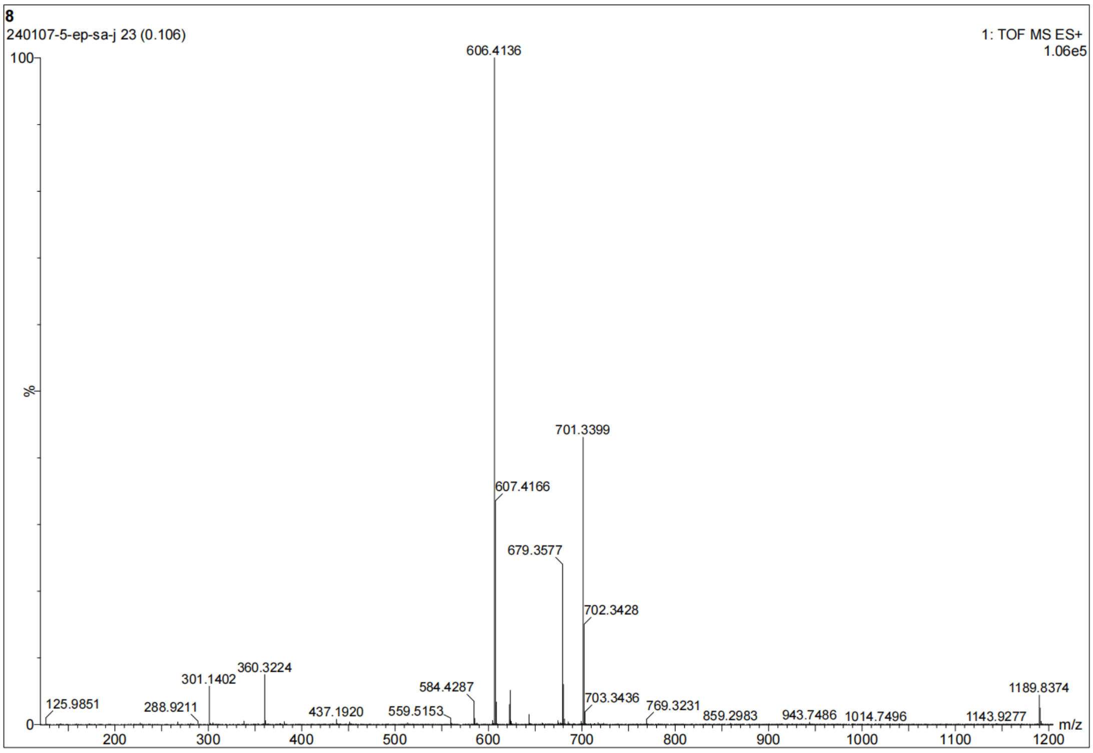

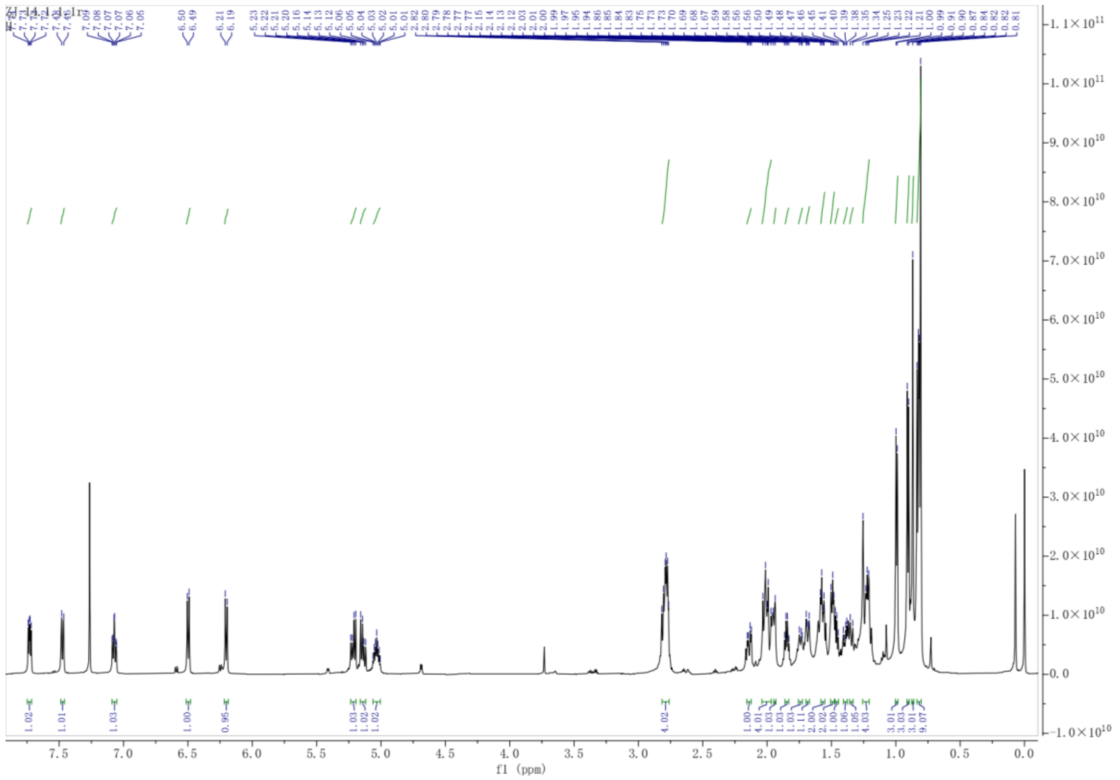

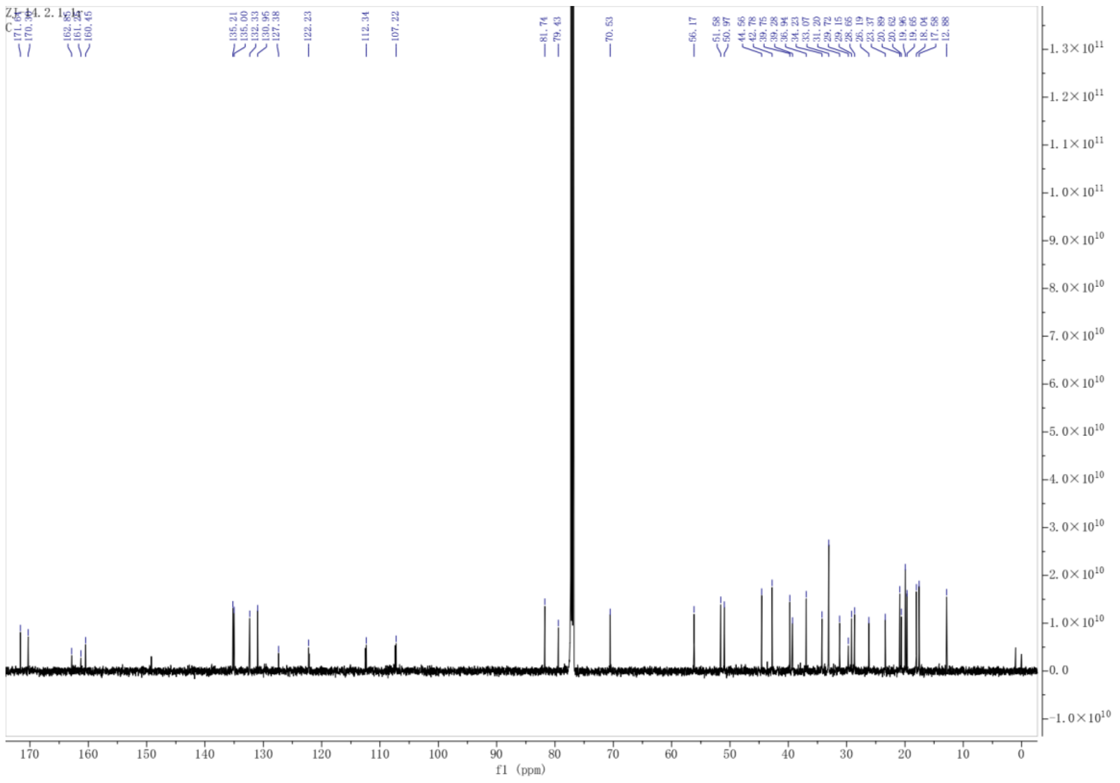

^{1}H NMR, ^{13}C NMR and HRMS spectra of **2d**

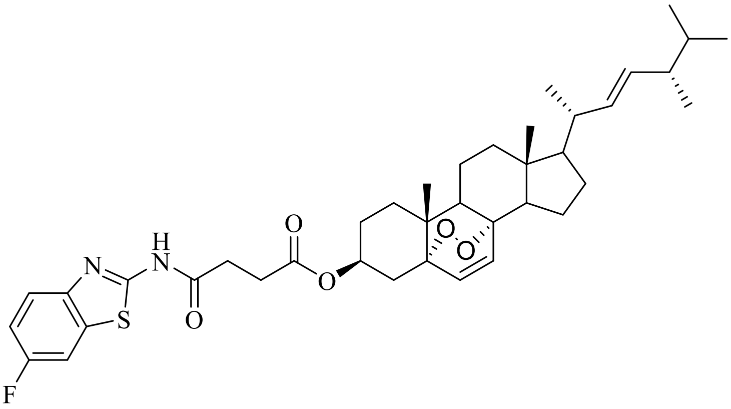

2d

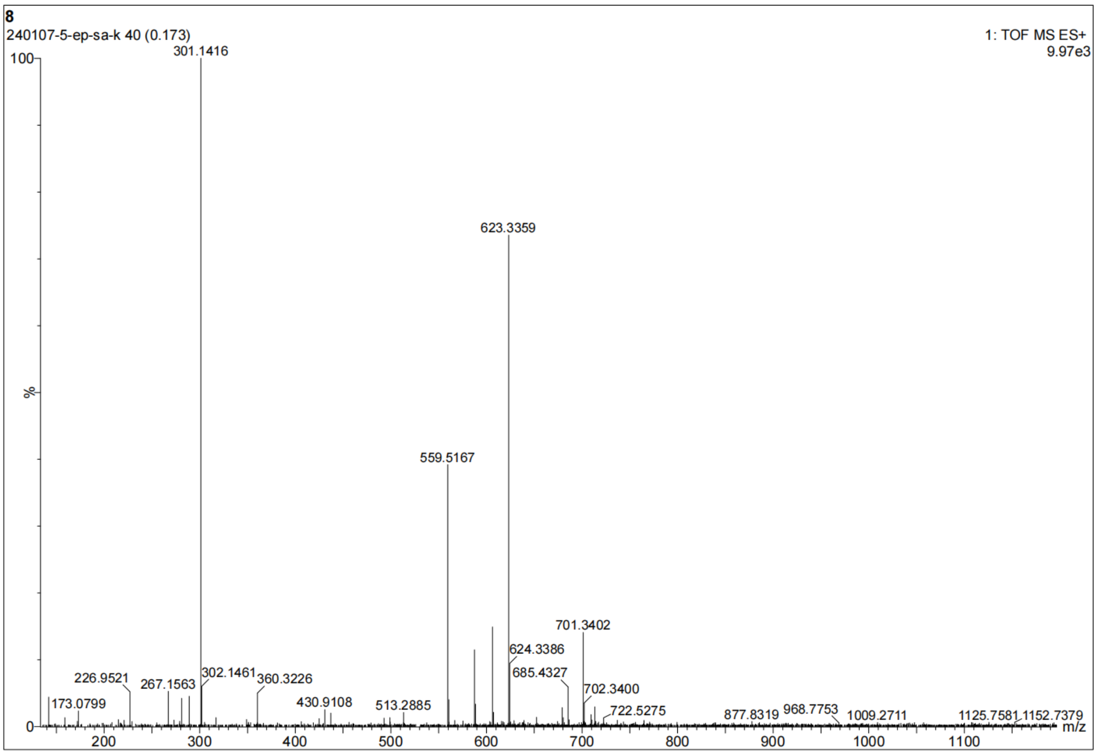

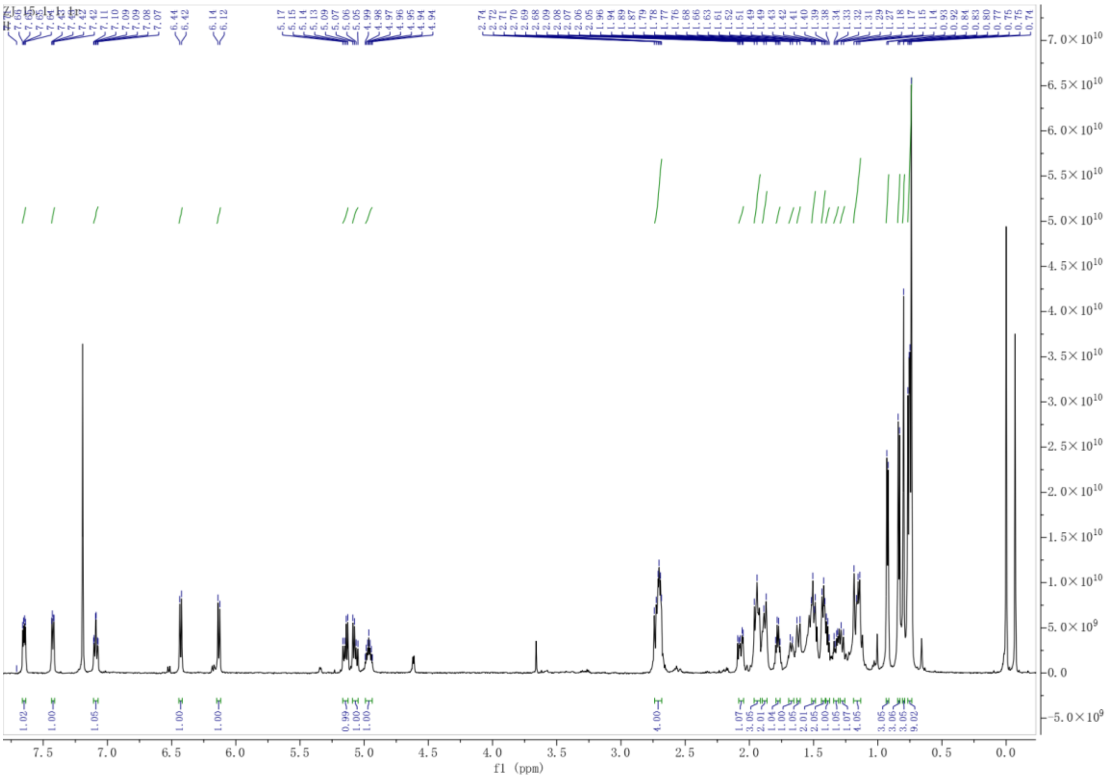
f1 (ppm)

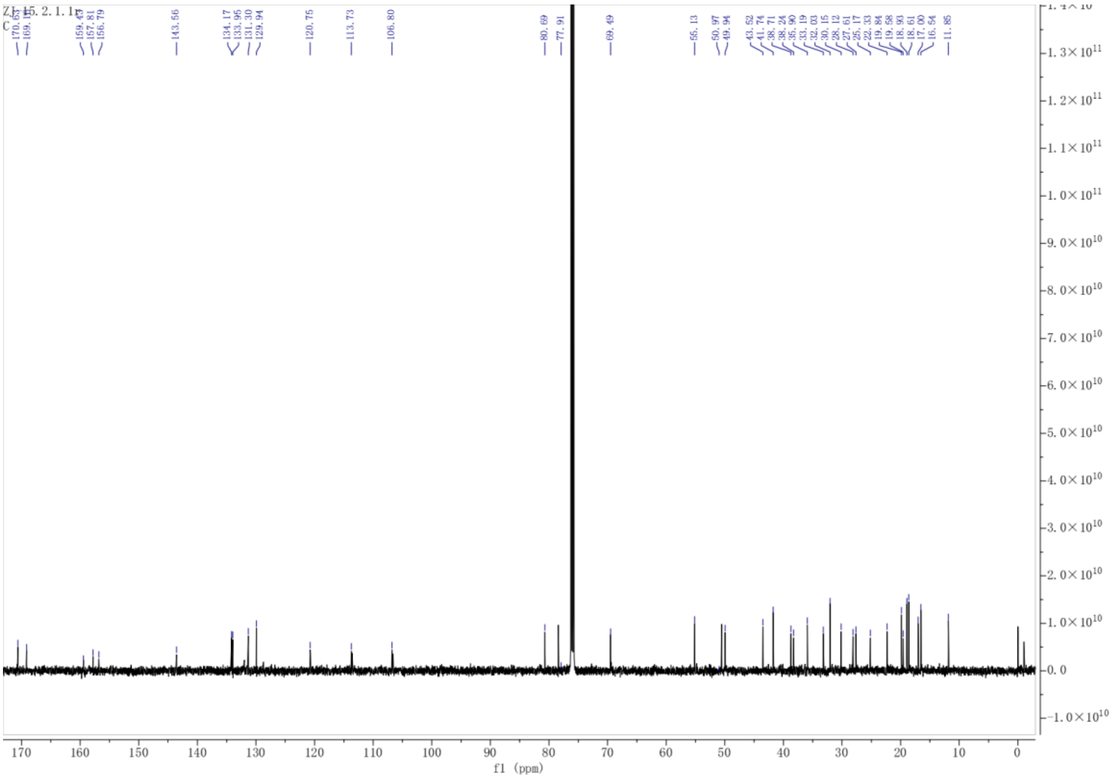
f1 (ppm)

^{1}H NMR, ^{13}C NMR and HRMS spectra of **2e**

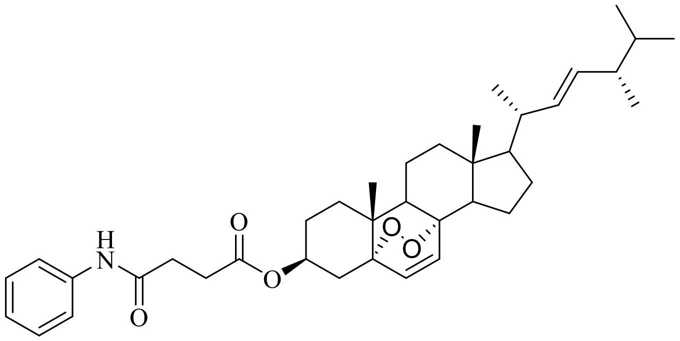

2e

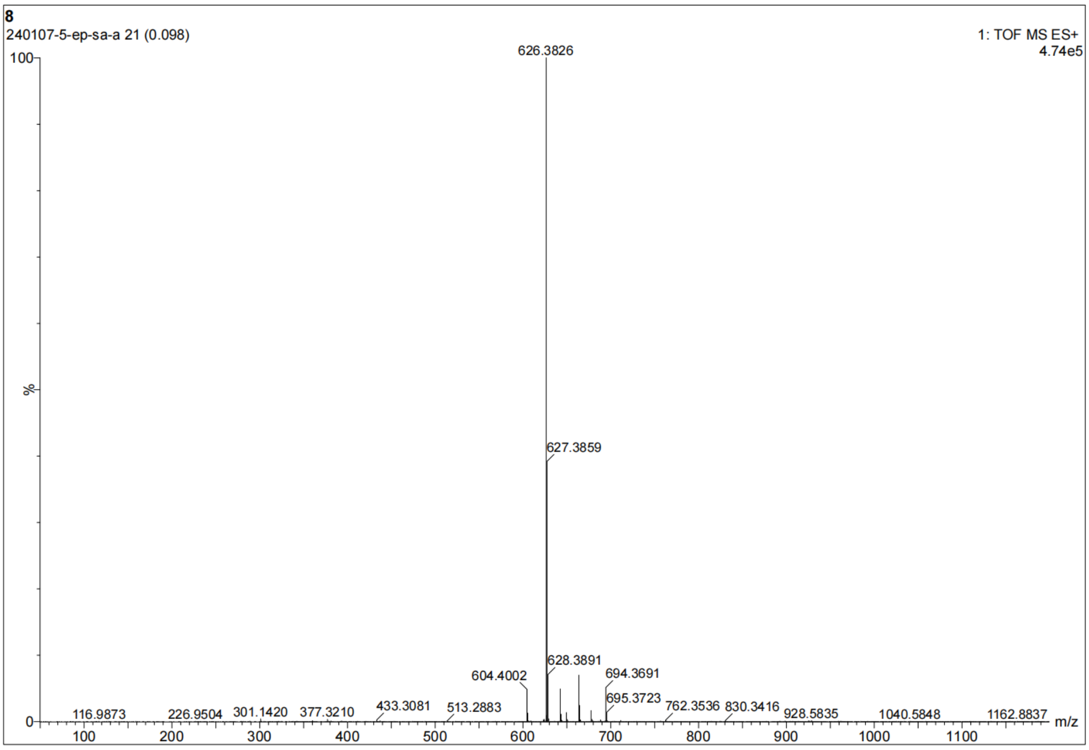

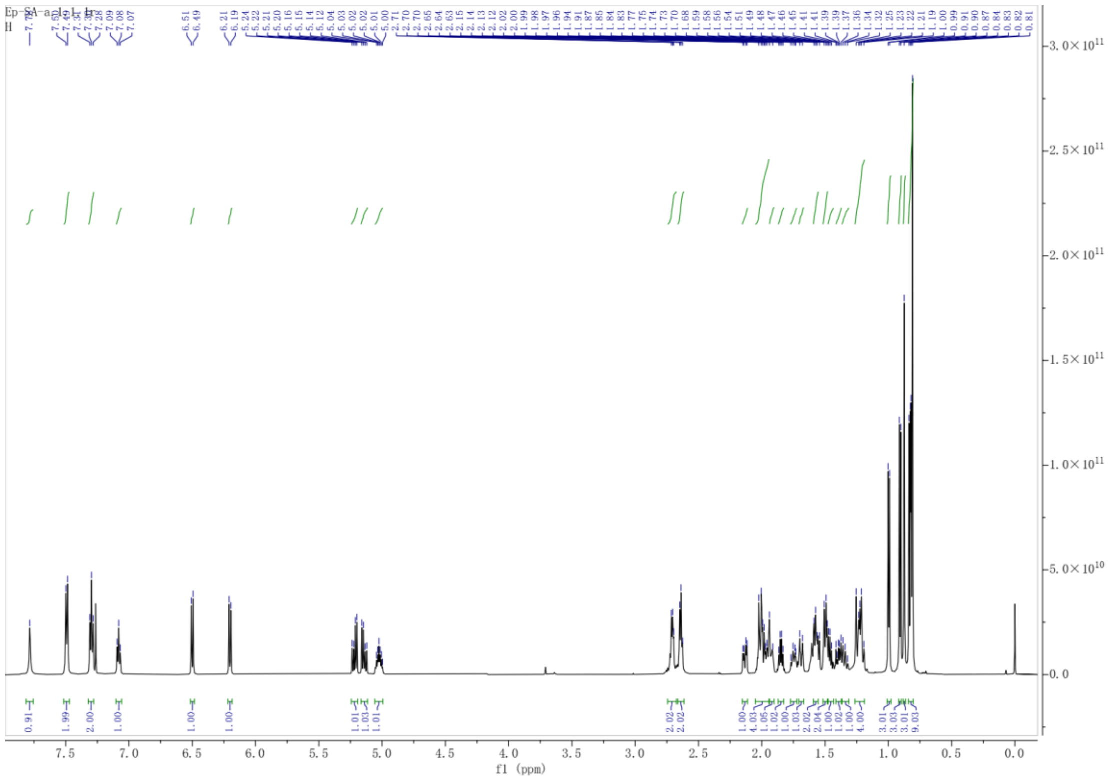

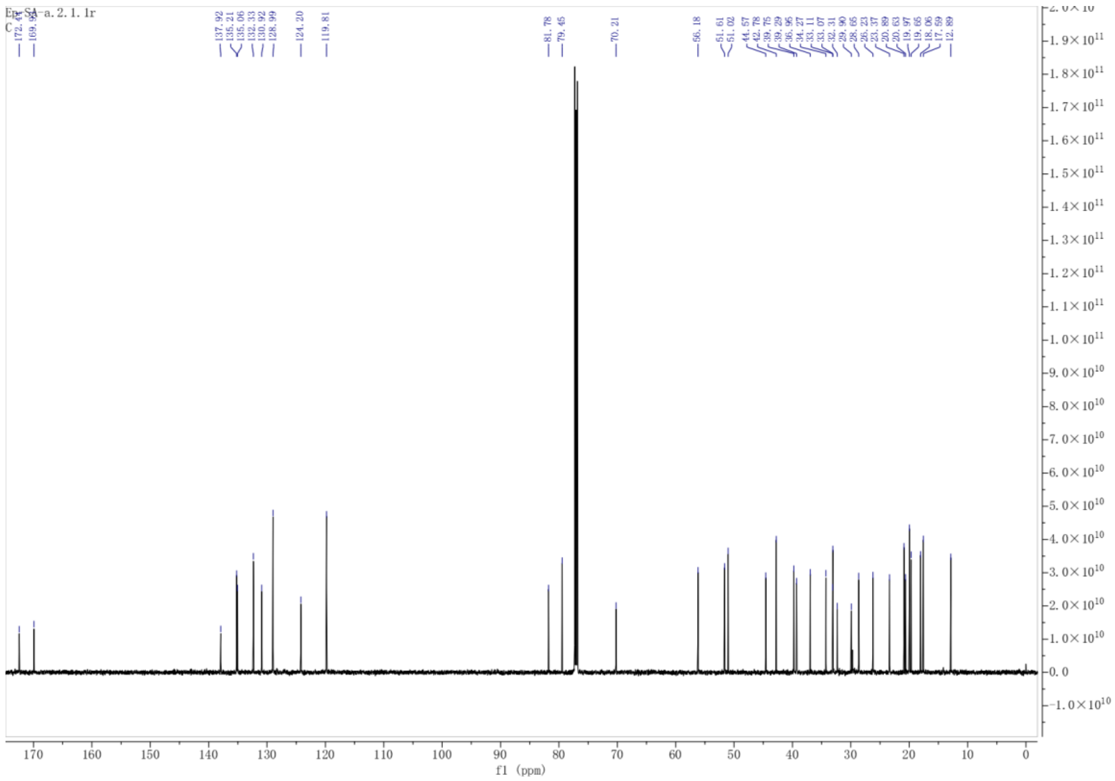

^{1}H NMR, ^{13}C NMR and HRMS spectra of **2f**

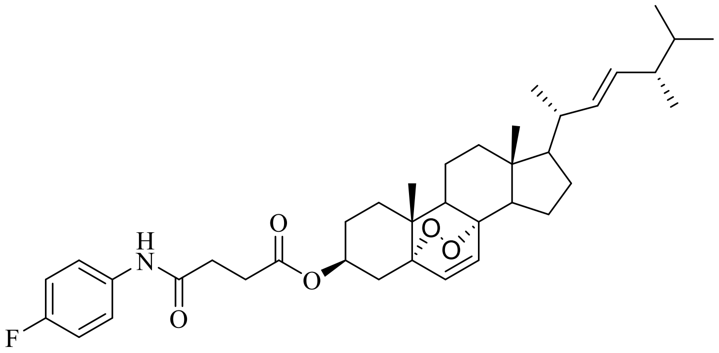

2f

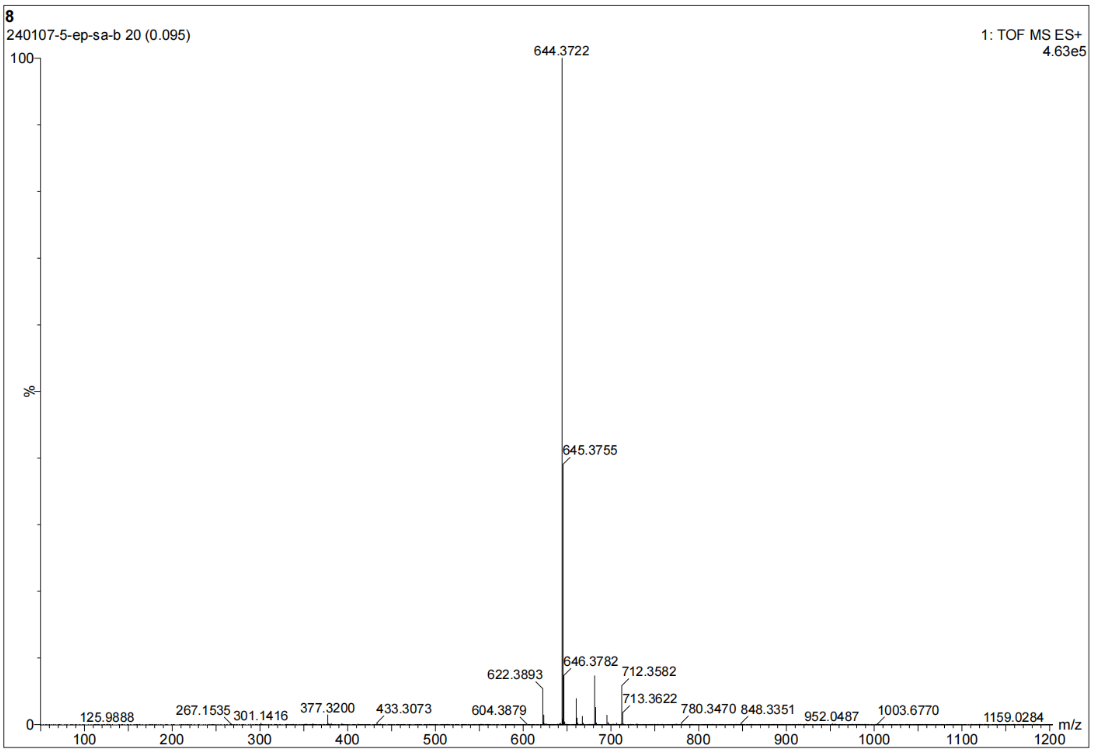

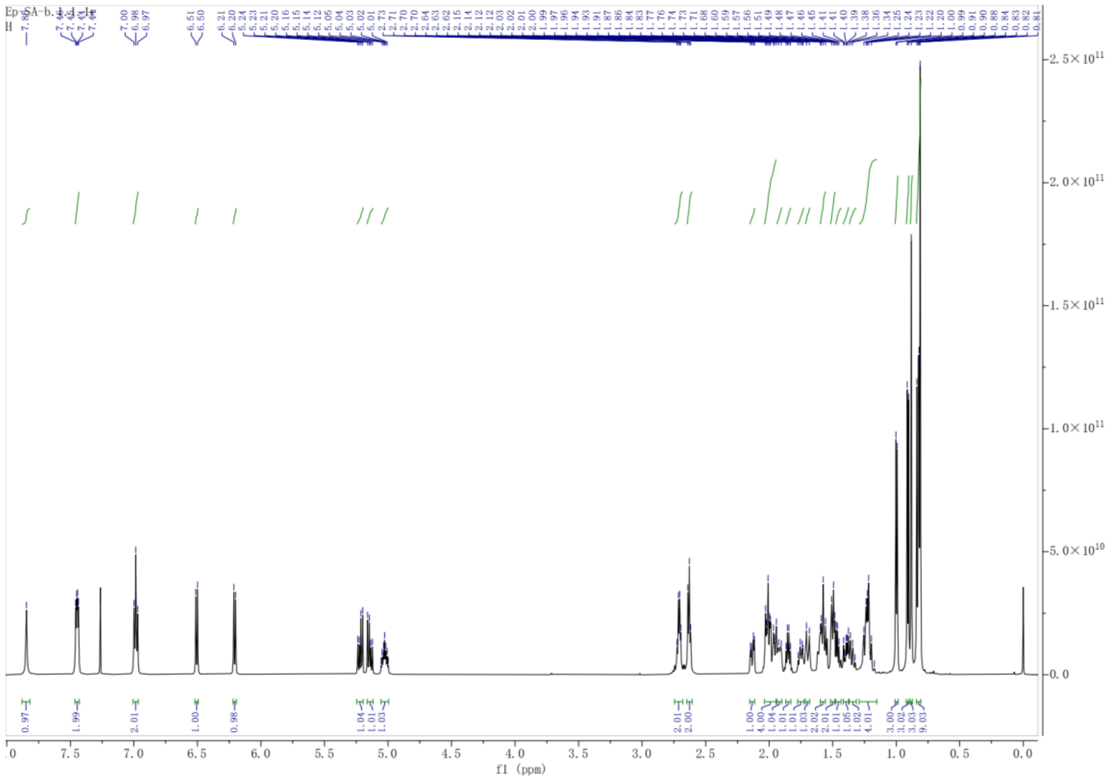

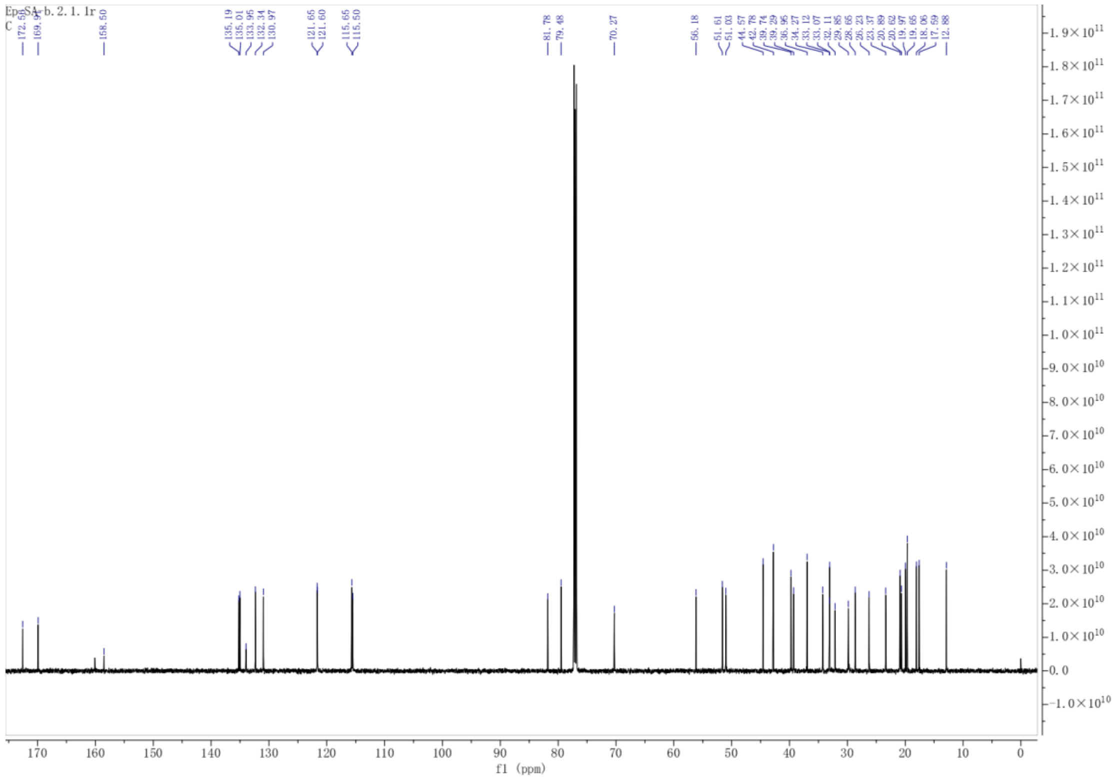

^{1}H NMR, ^{13}C NMR and HRMS spectra of **2g**

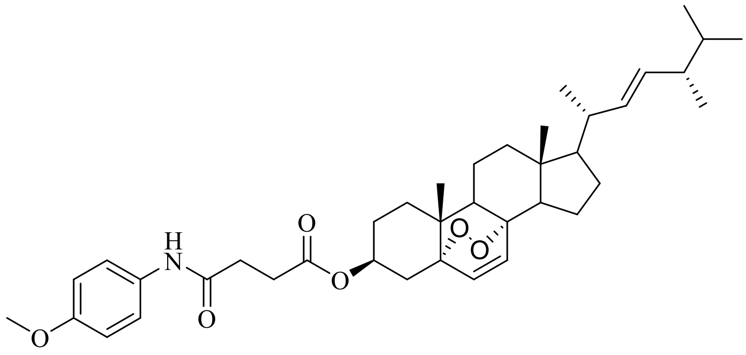

2g

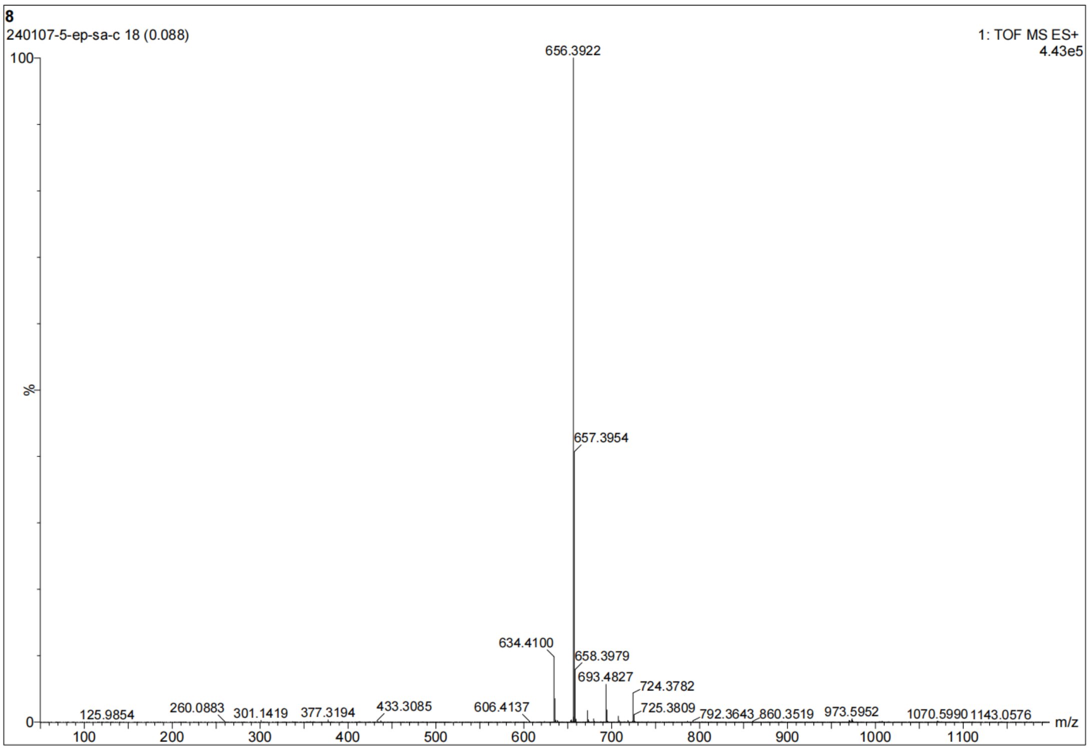

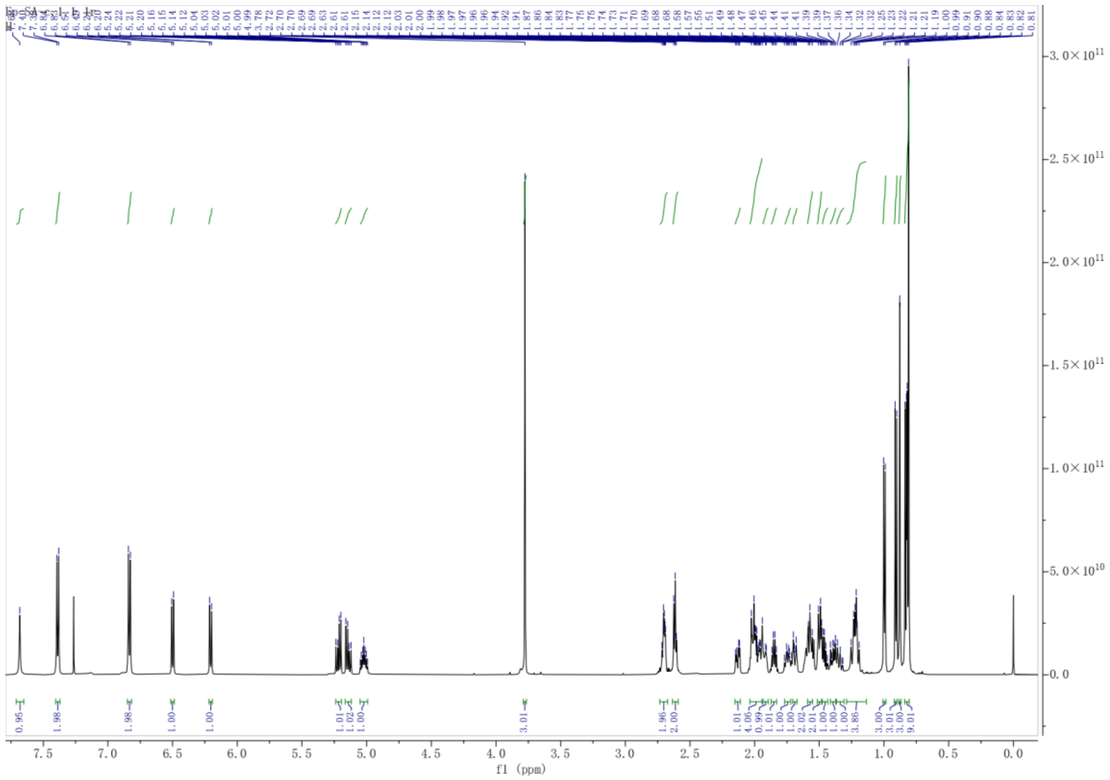

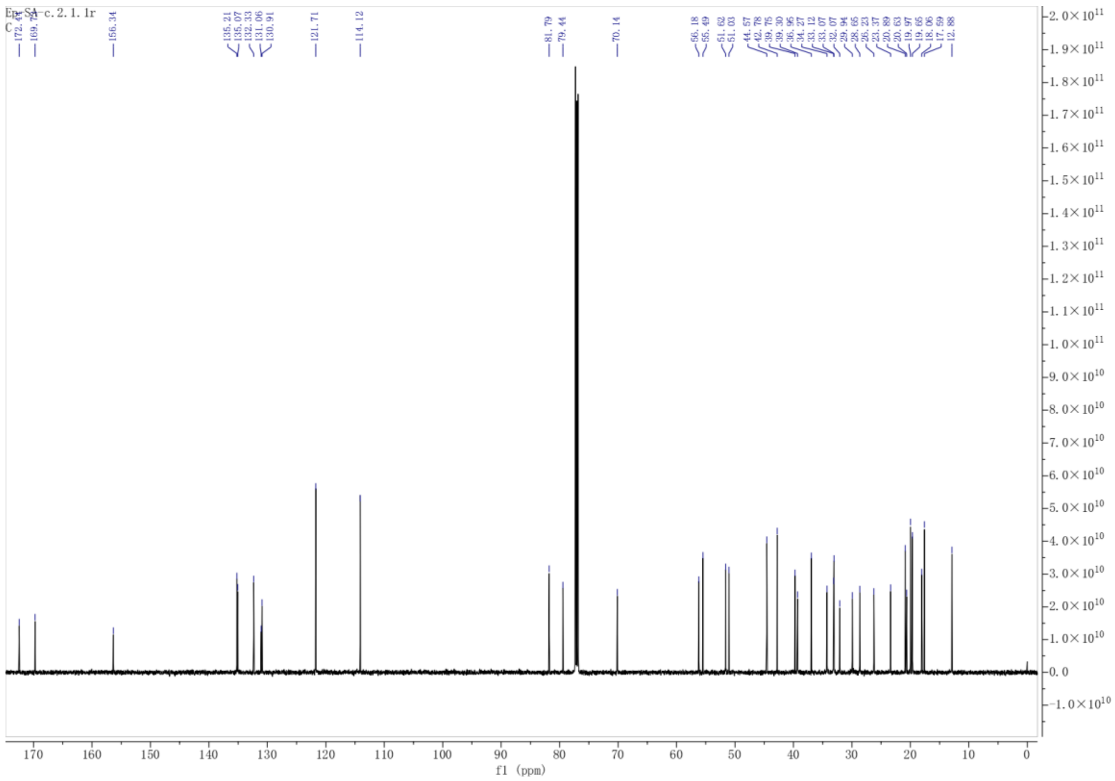

^{1}H NMR, ^{13}C NMR and HRMS spectra of **2h**

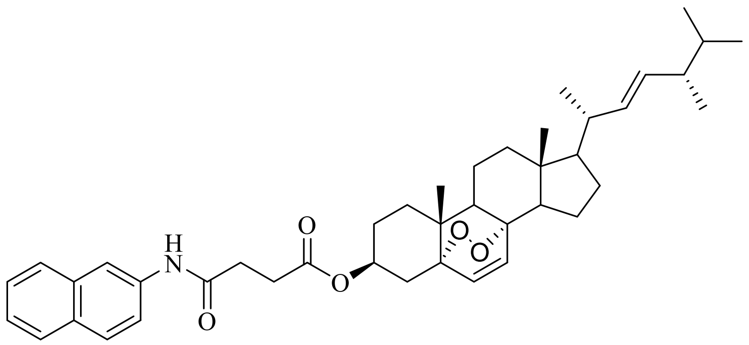

2h

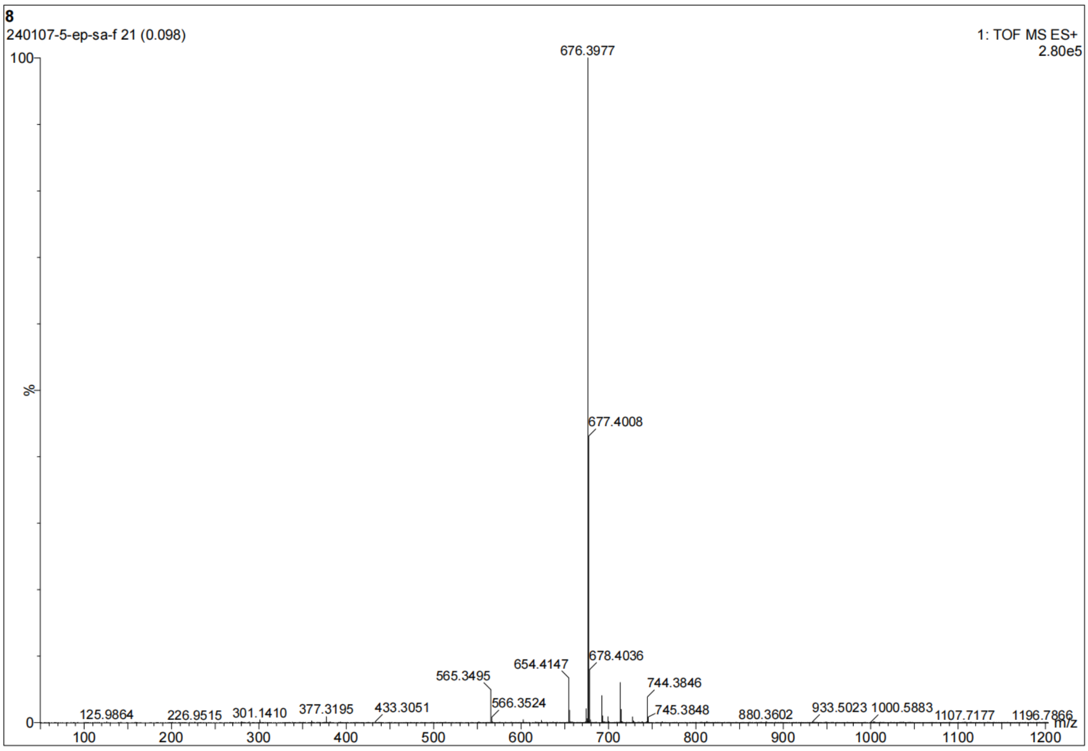

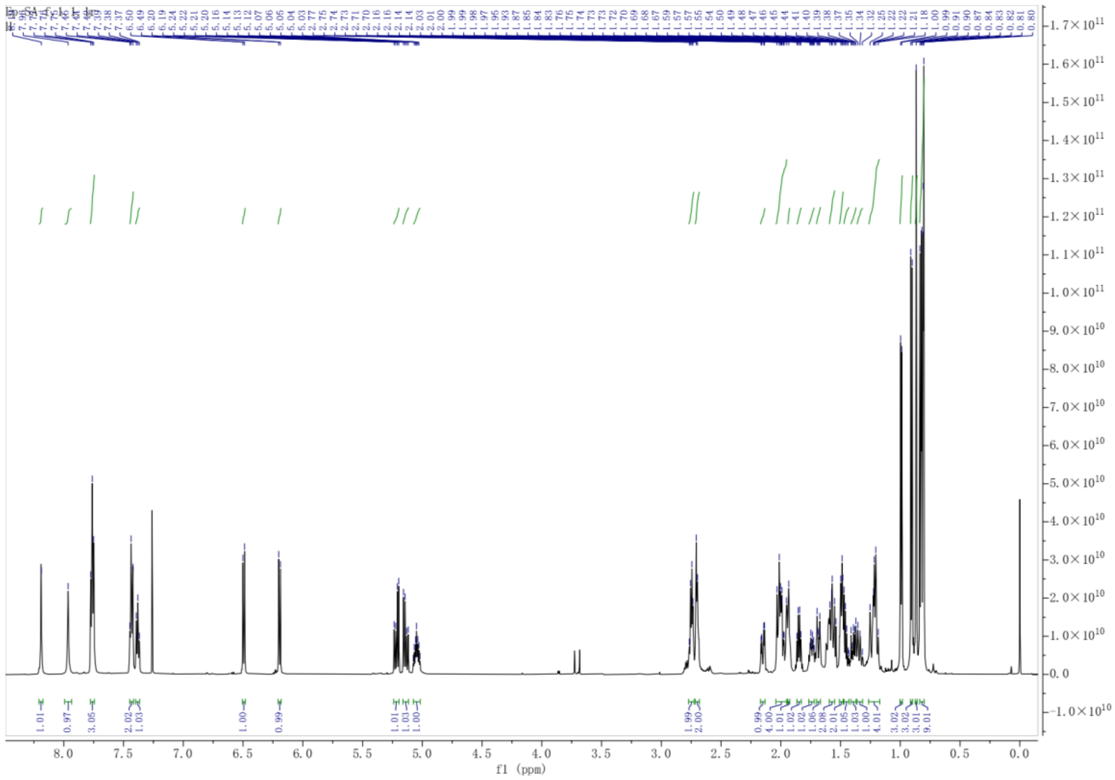

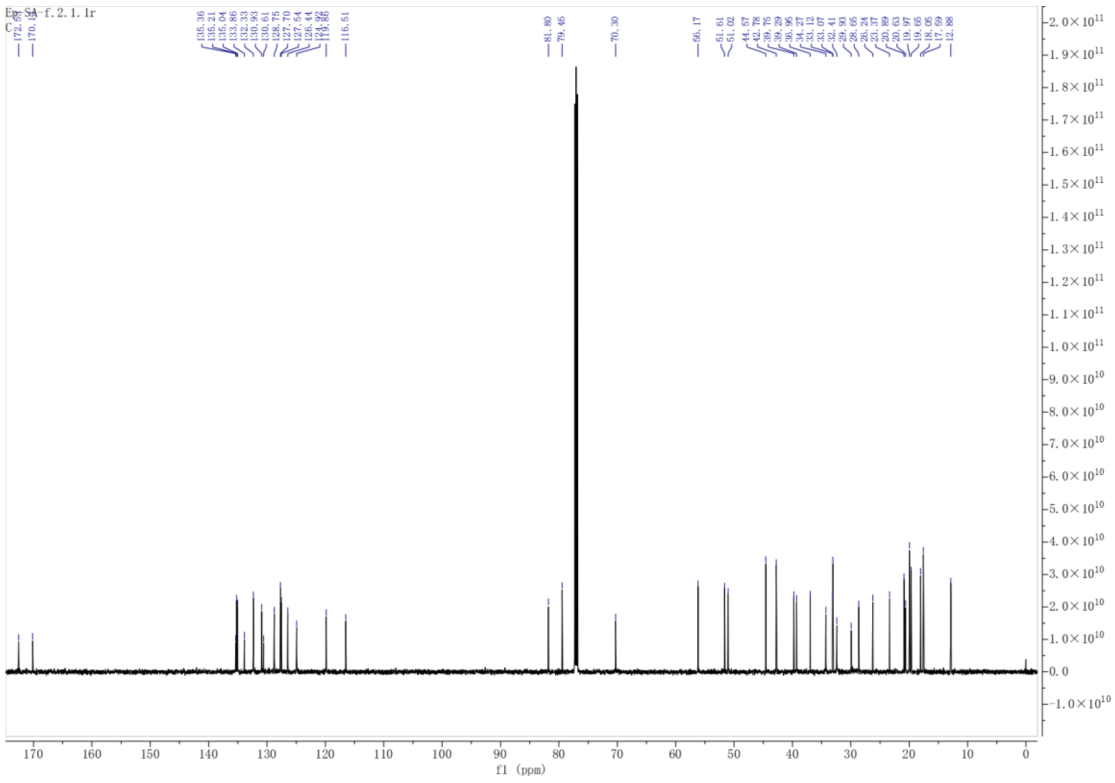

^{1}H NMR and ^{13}C NMR of **3**

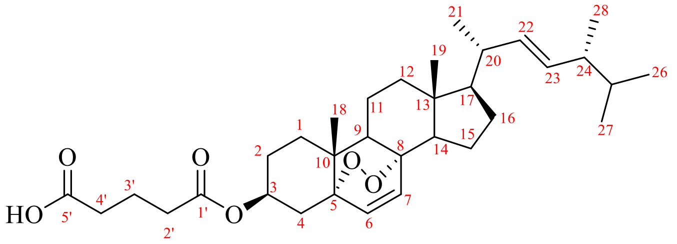

3

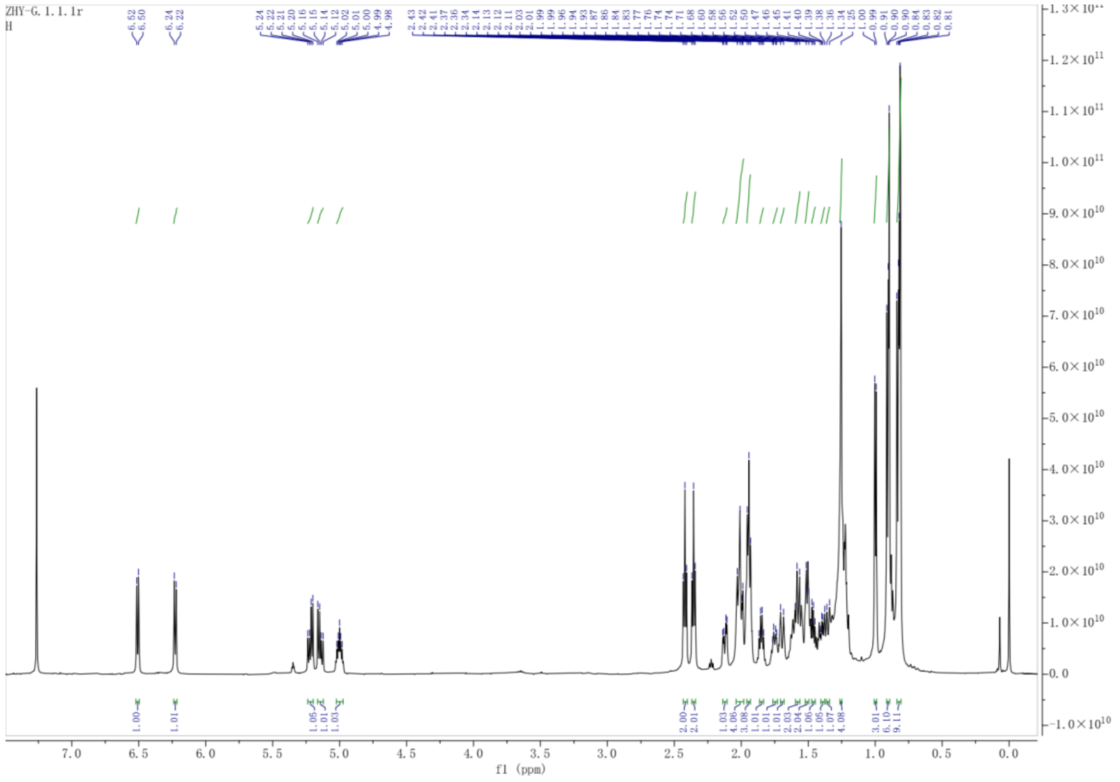

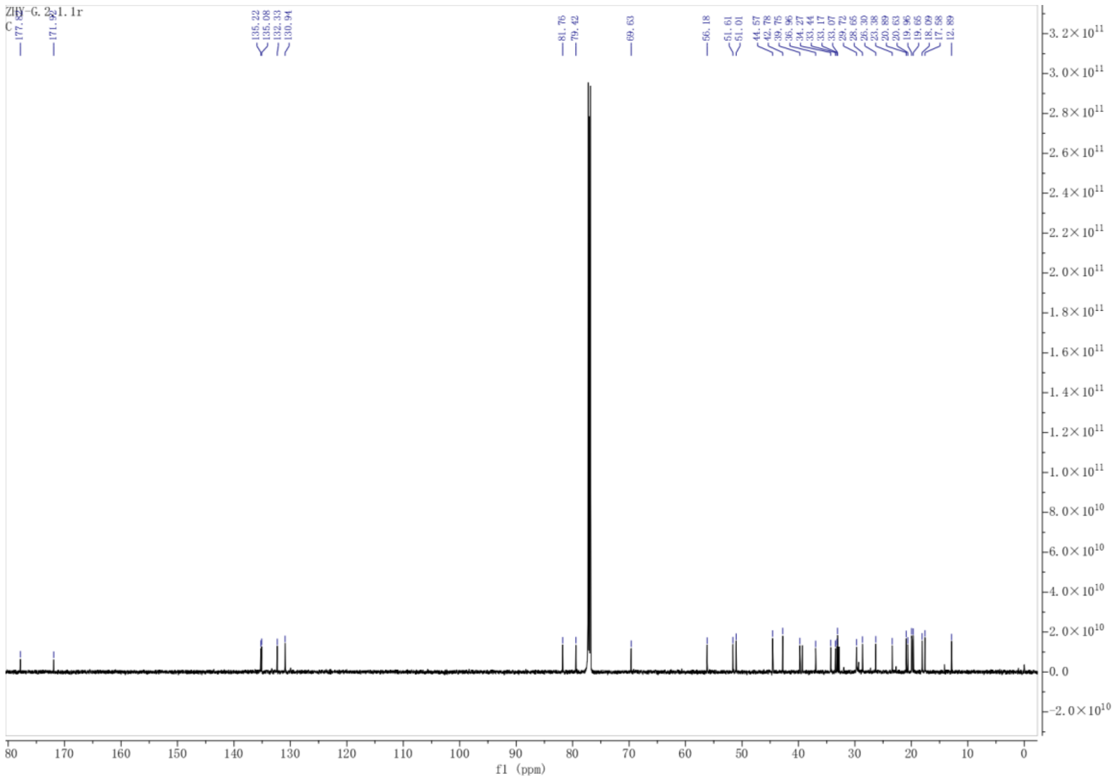
ZHY-G.2-1.1r
C
135.22
135.08
132.33
130.94
81.76
79.42
69.63
56.18
51.61
51.01
44.57
42.78
39.75
36.96
34.27
33.44
33.17
33.07
29.72
28.65
26.30
23.38
20.89
20.63
19.96
19.65
18.09
17.58
12.89
f1 (ppm)

^{1}H NMR, ^{13}C NMR and HRMS spectra of **3a**

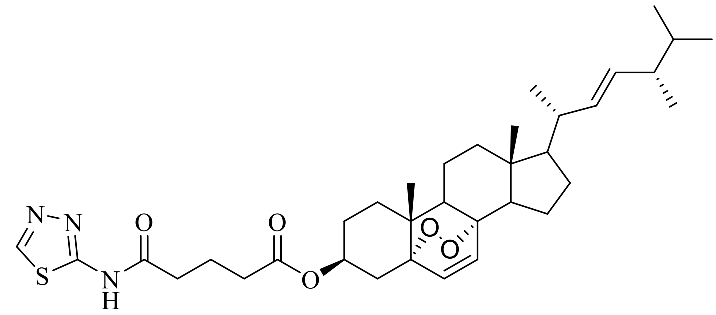

3a

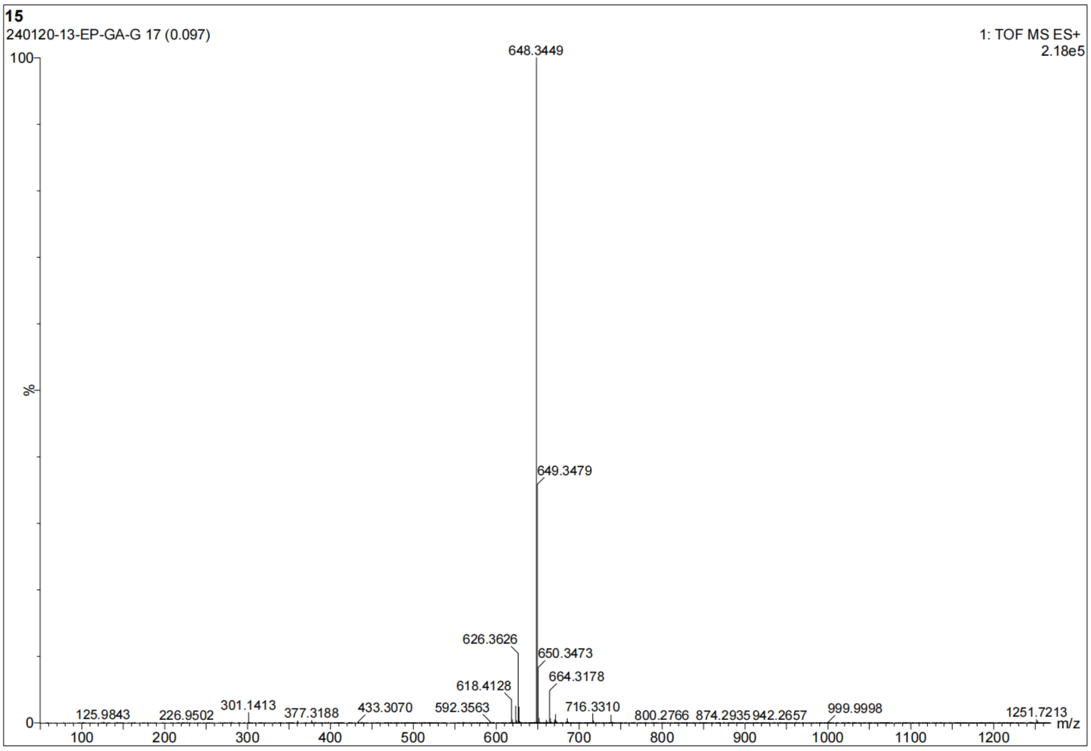

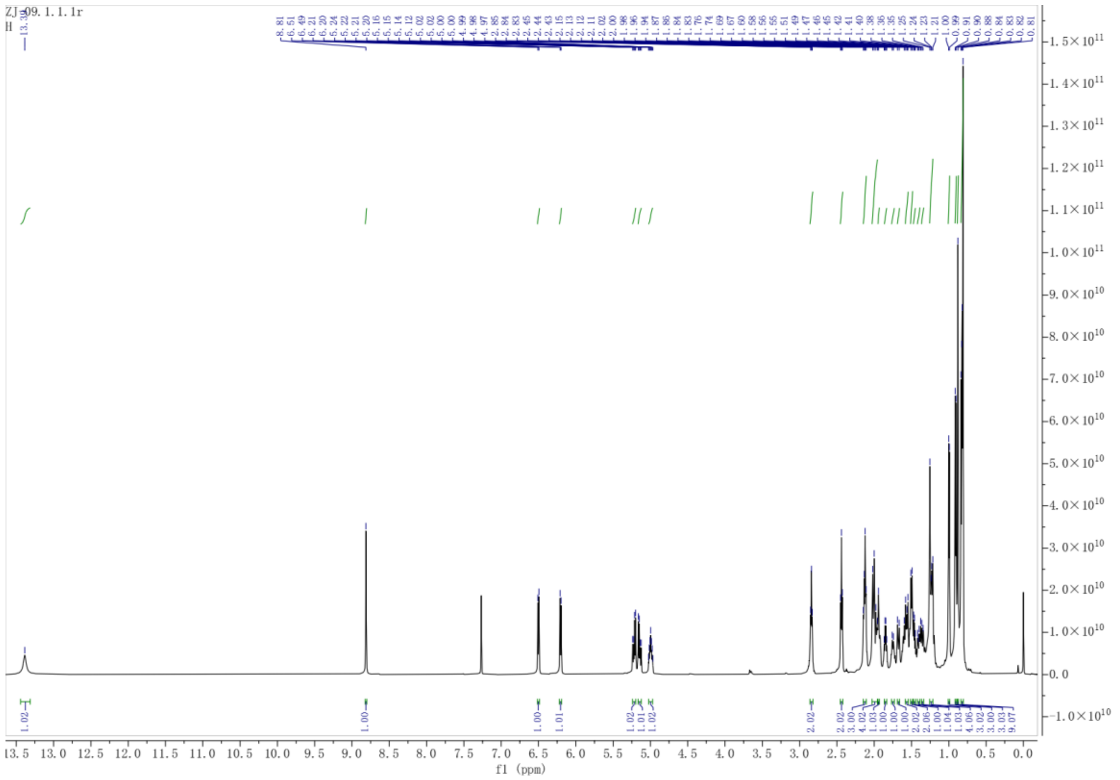
ZJ-09.1.1.1r
f1 (ppm)

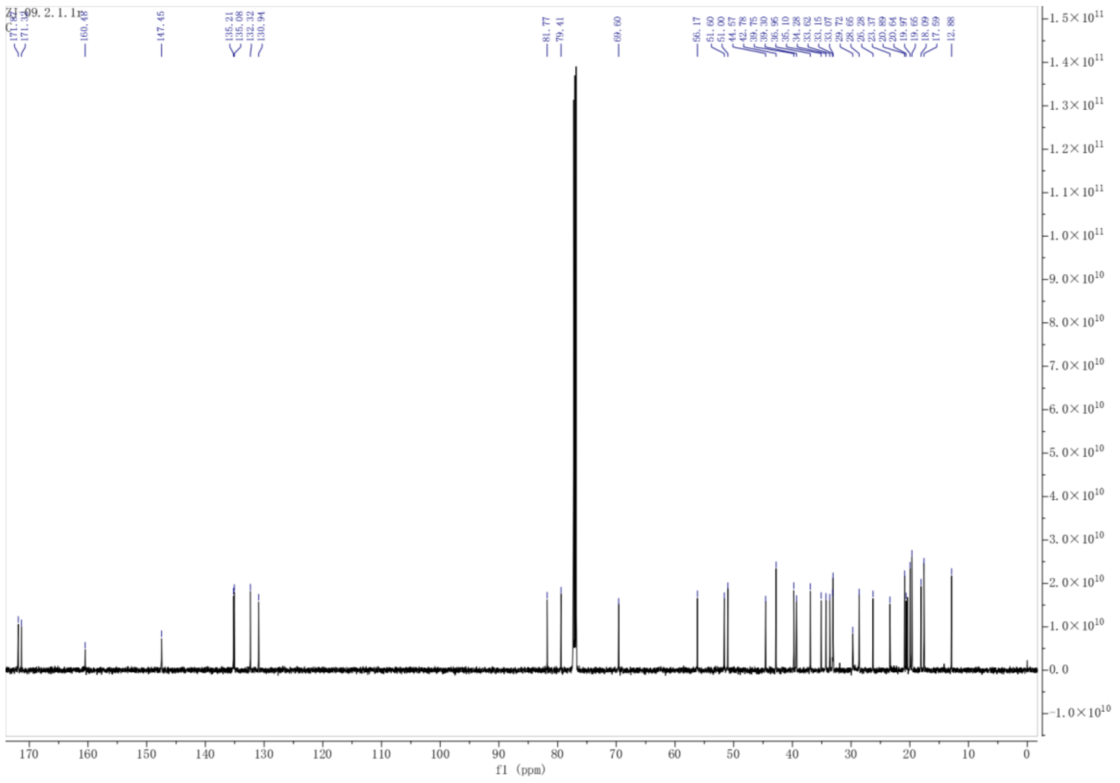
ZJ-09.2.1.1r
f1 (ppm)

^{1}H NMR, ^{13}C NMR and HRMS spectra of **3b**

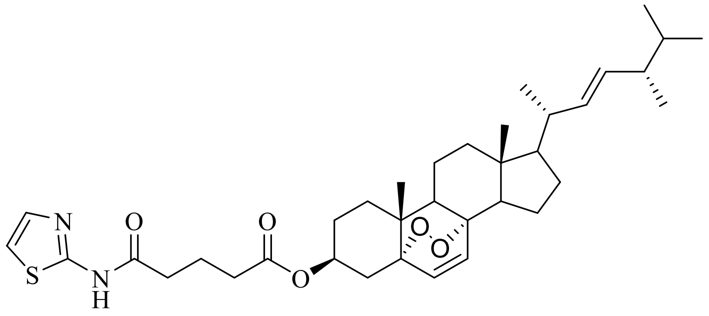

3b

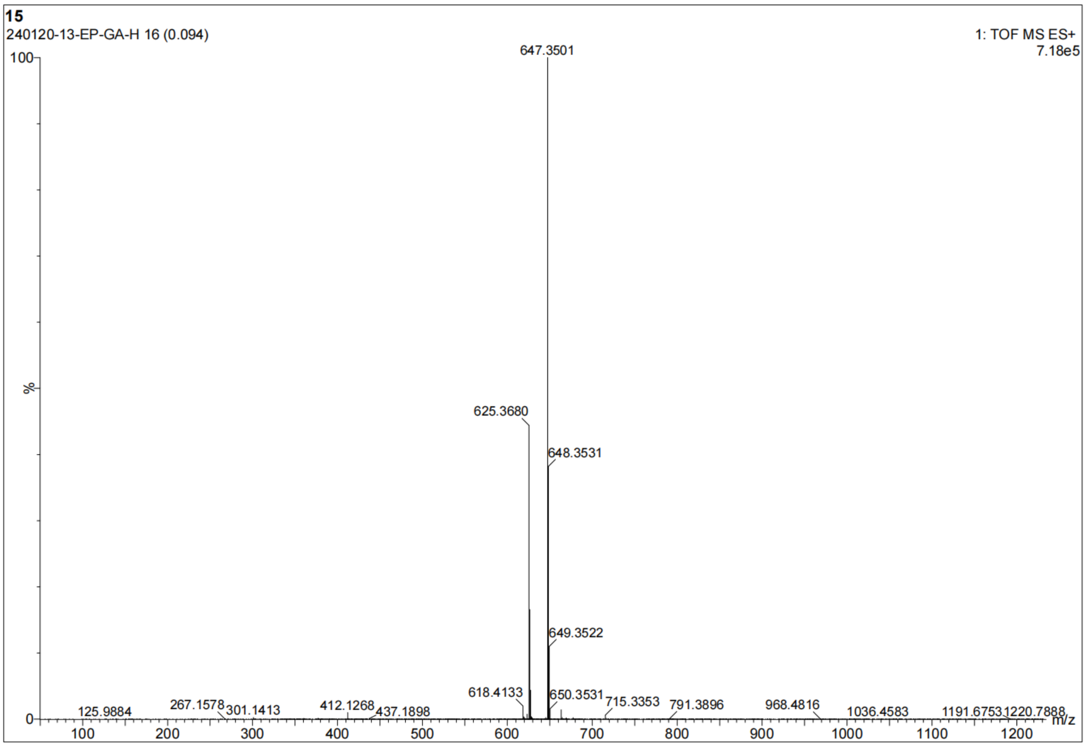

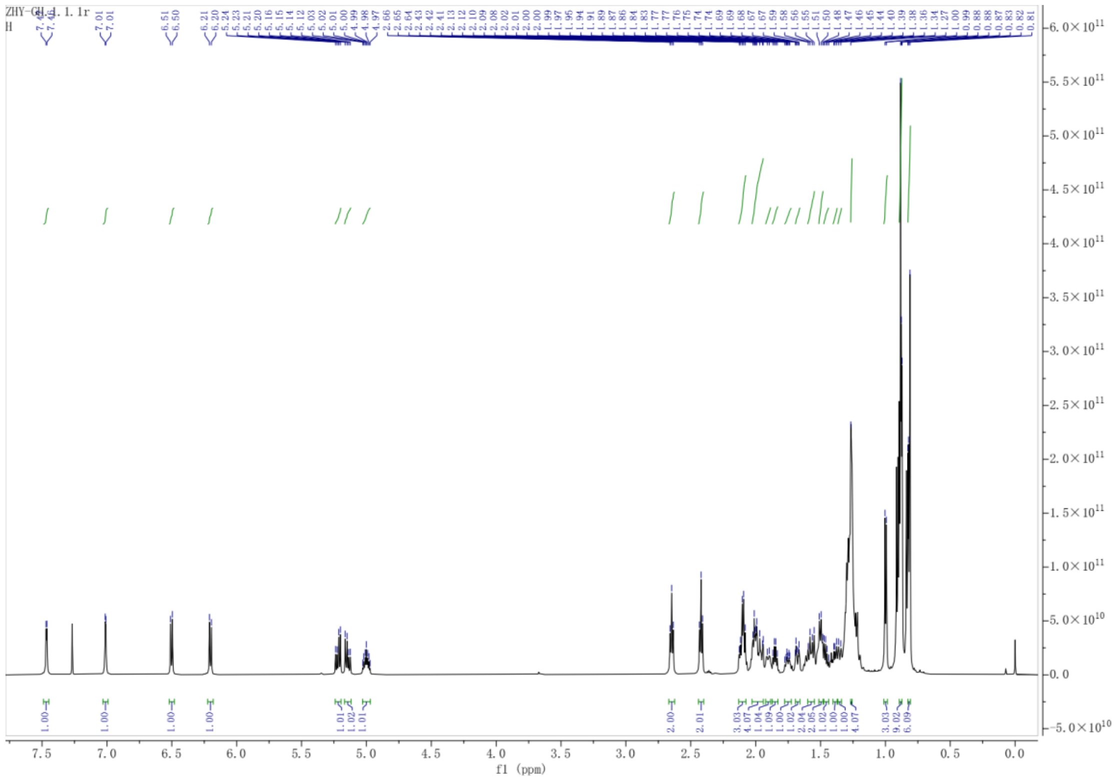

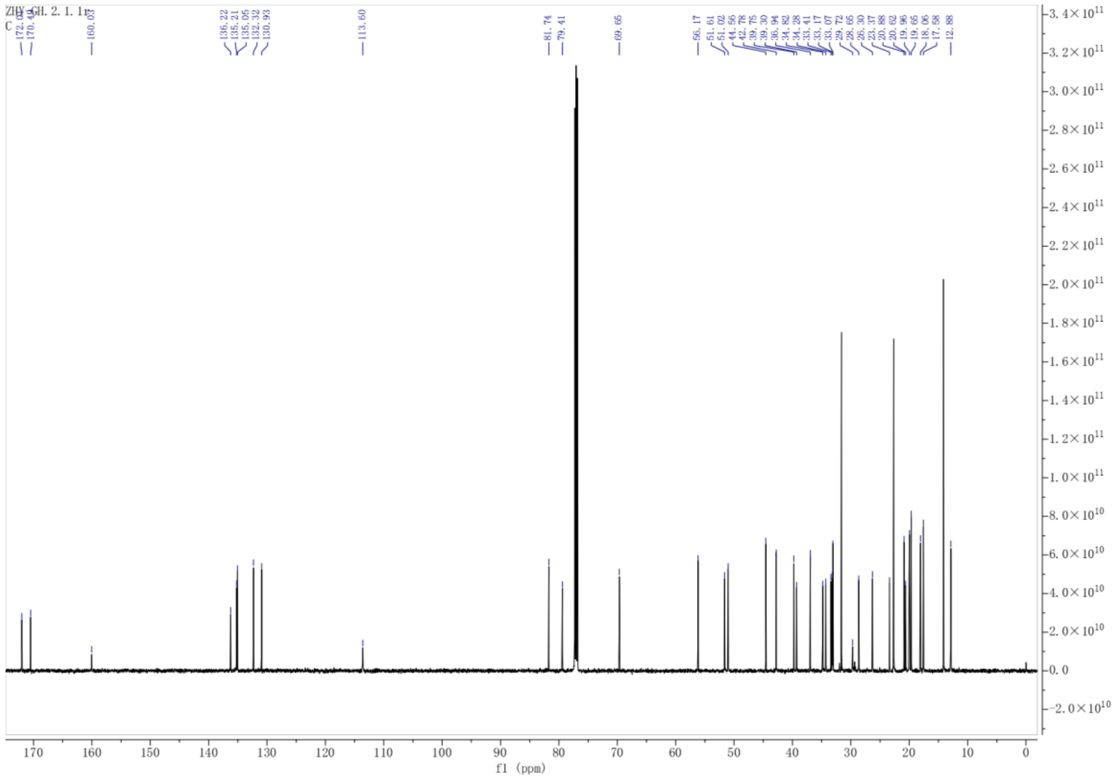

^{1}H NMR, ^{13}C NMR and HRMS spectra of **3c**

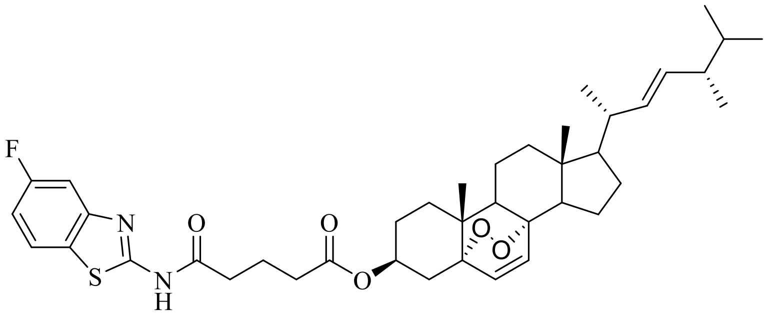

3c

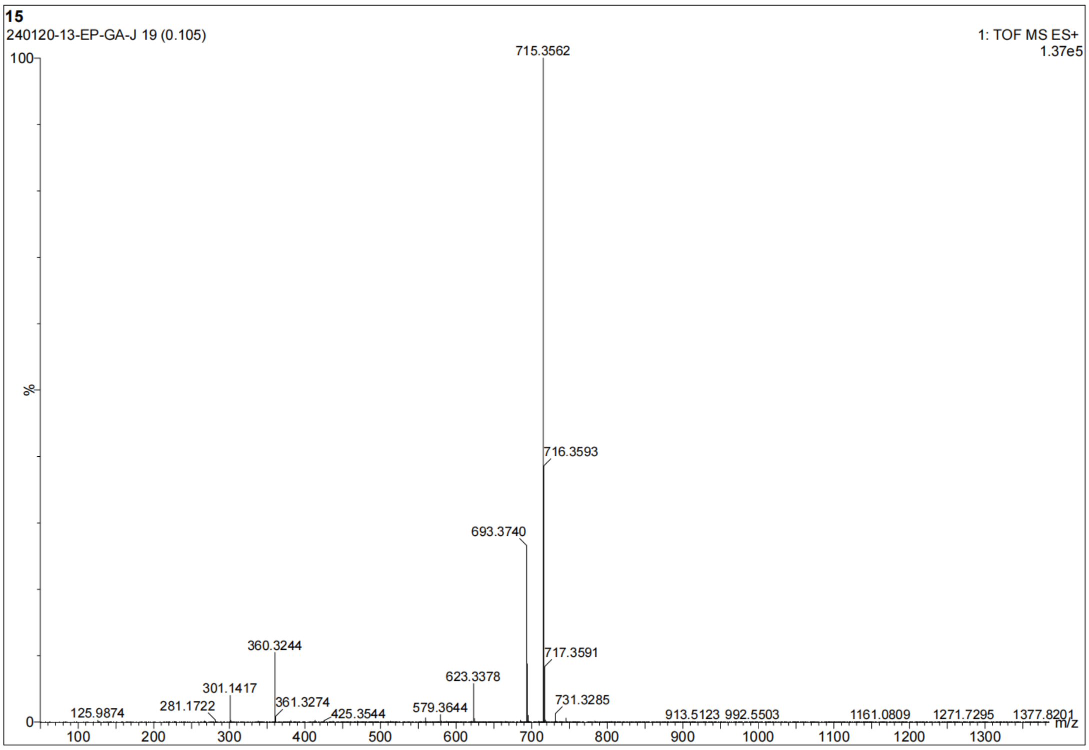

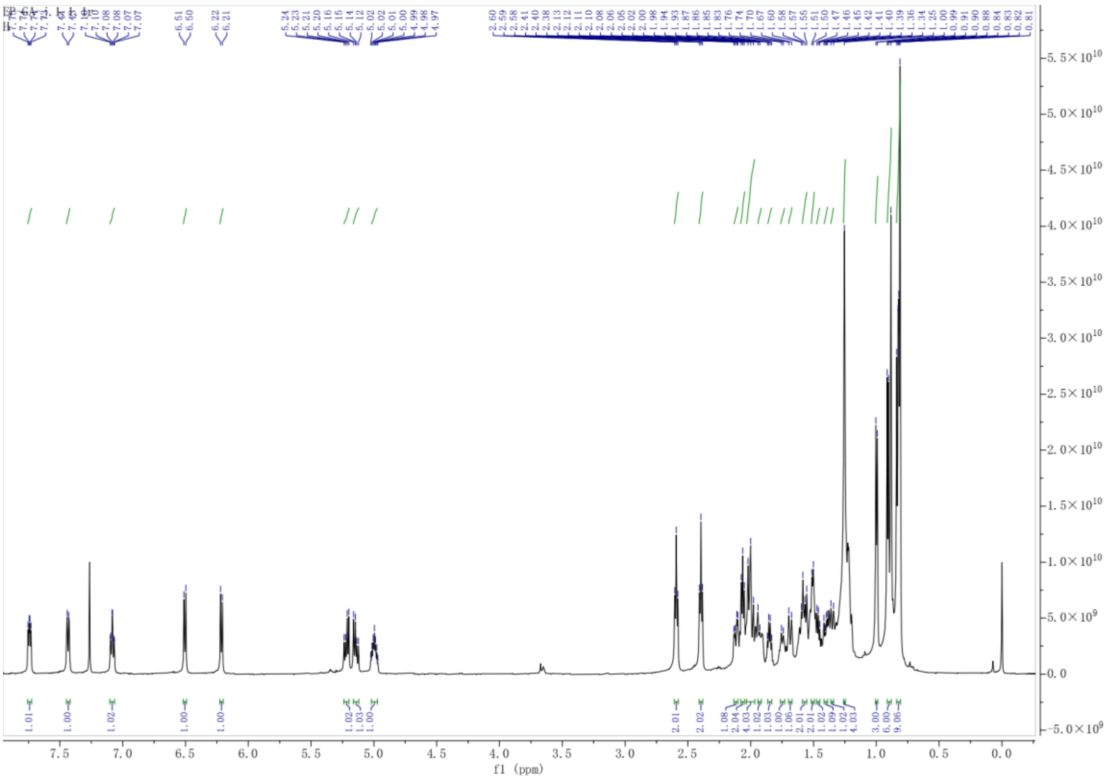
f1 (ppm)

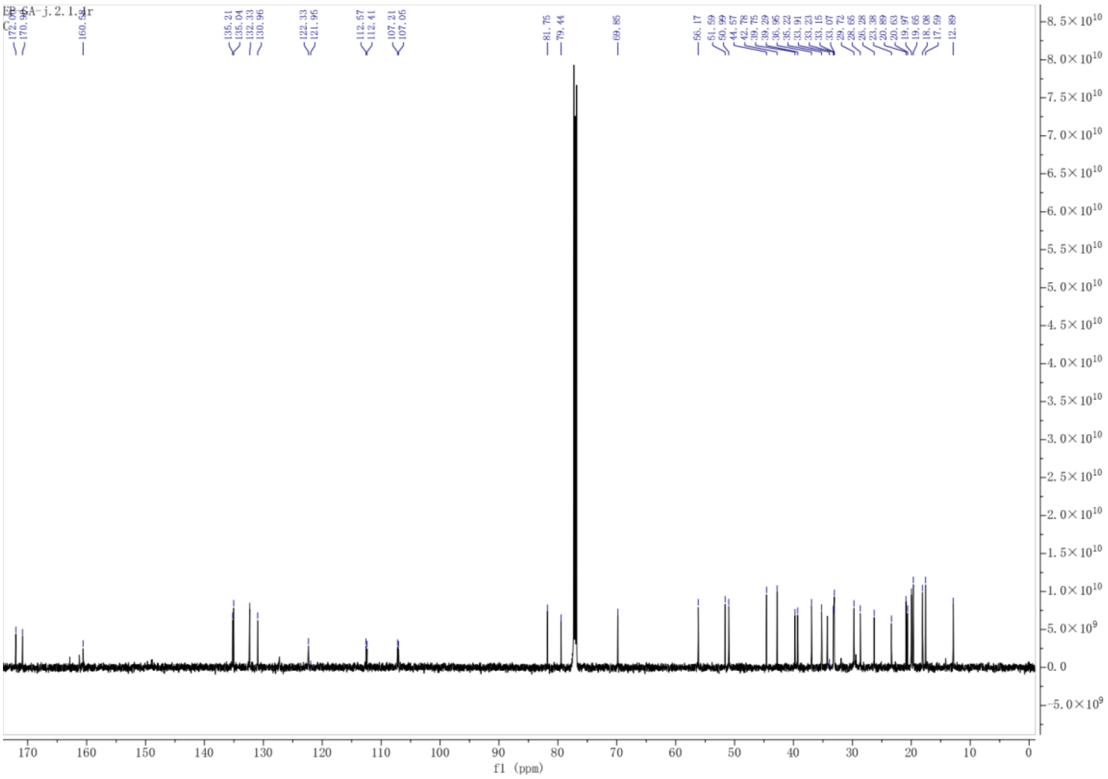
f1 (ppm)

^{1}H NMR, ^{13}C NMR and HRMS spectra of **3d**

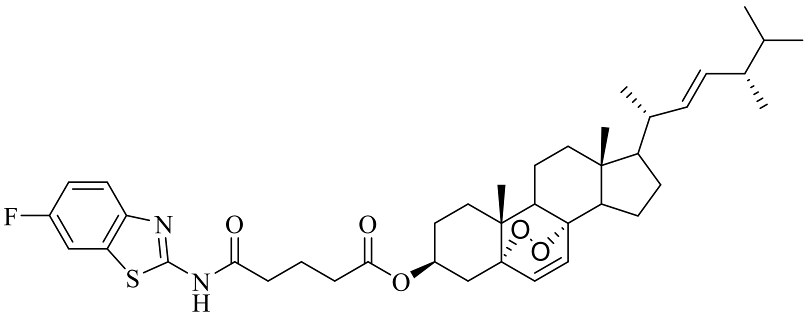

3d

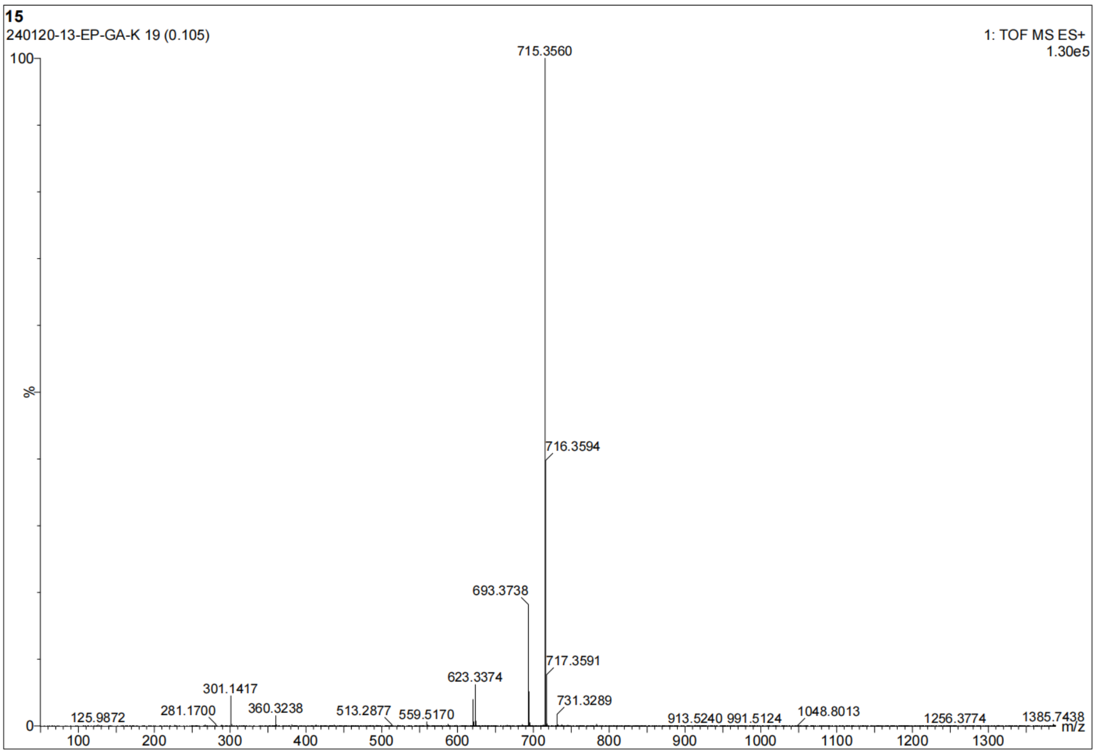

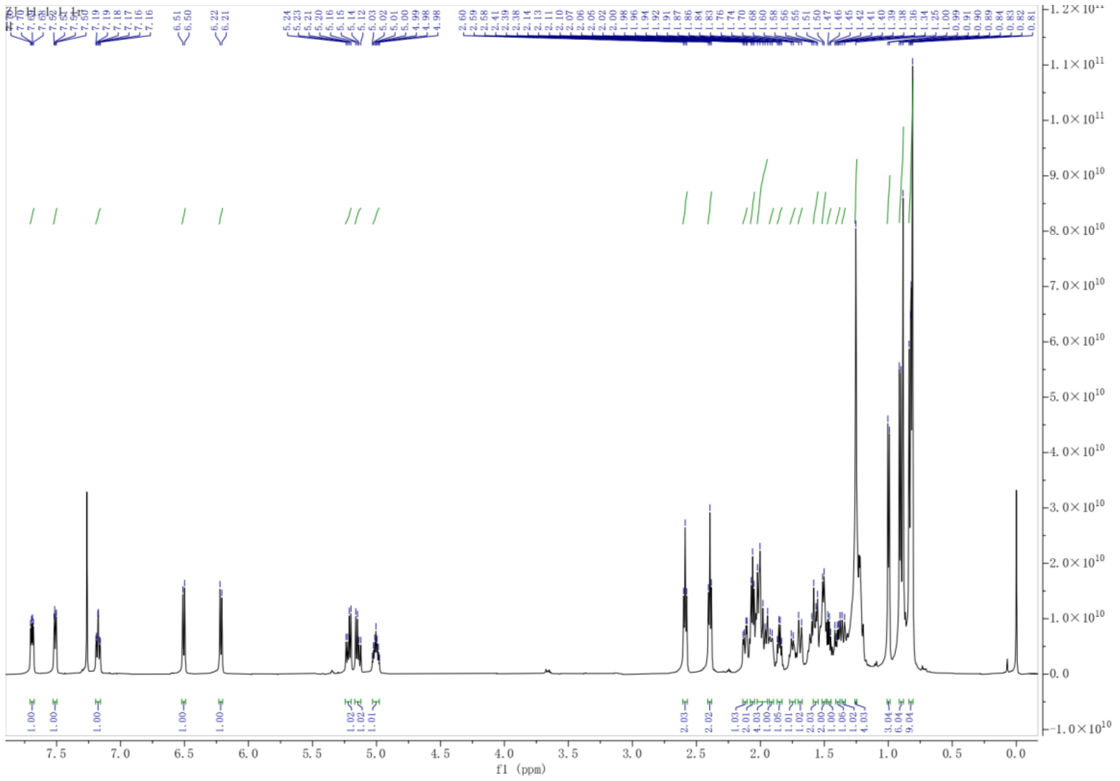
f1 (ppm)
7.5 7.0 6.5 6.0 5.5 5.0 4.5 4.0 3.5 3.0 2.5 2.0 1.5 1.0 0.5 0.0

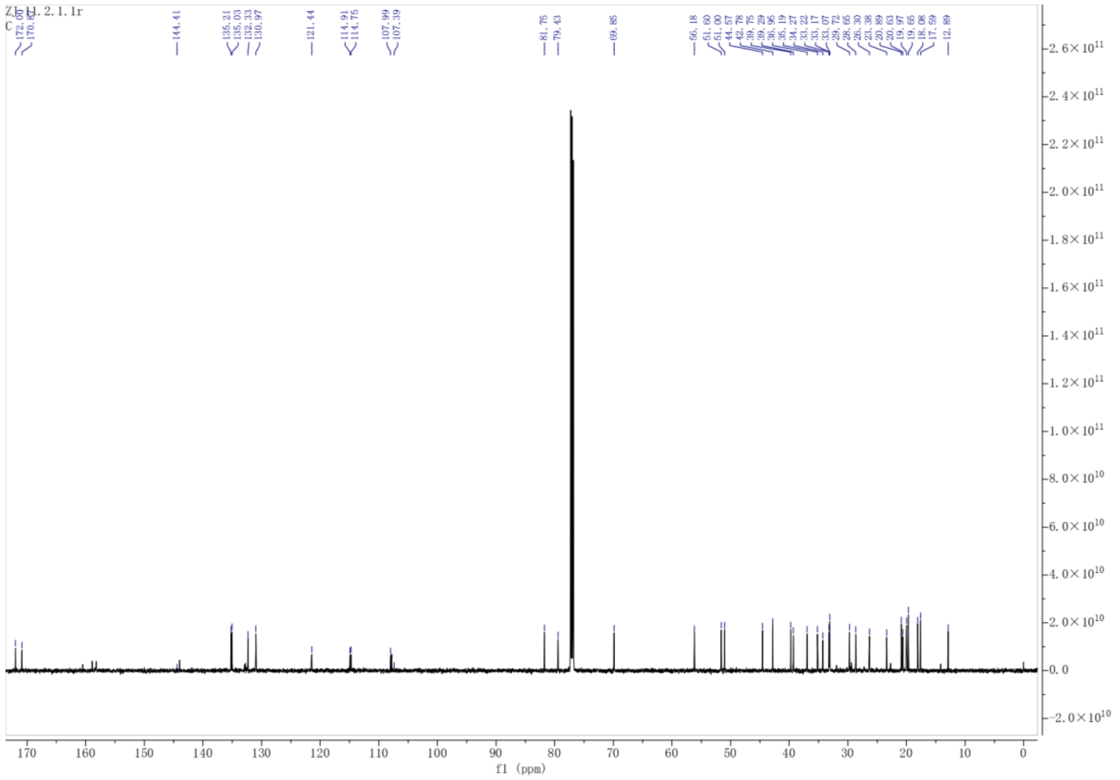
f1 (ppm)
170 160 150 140 130 120 110 100 90 80 70 60 50 40 30 20 10 0

^{1}H NMR, ^{13}C NMR and HRMS spectra of **3e**

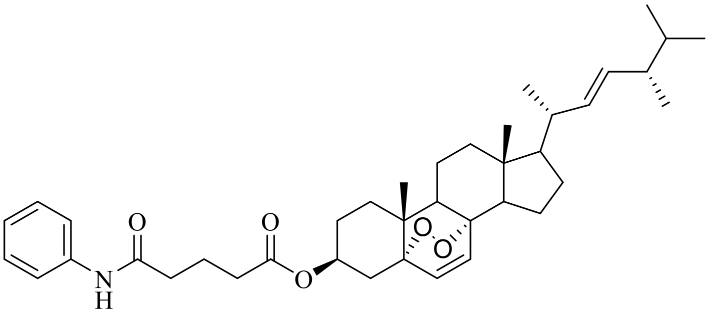

3e

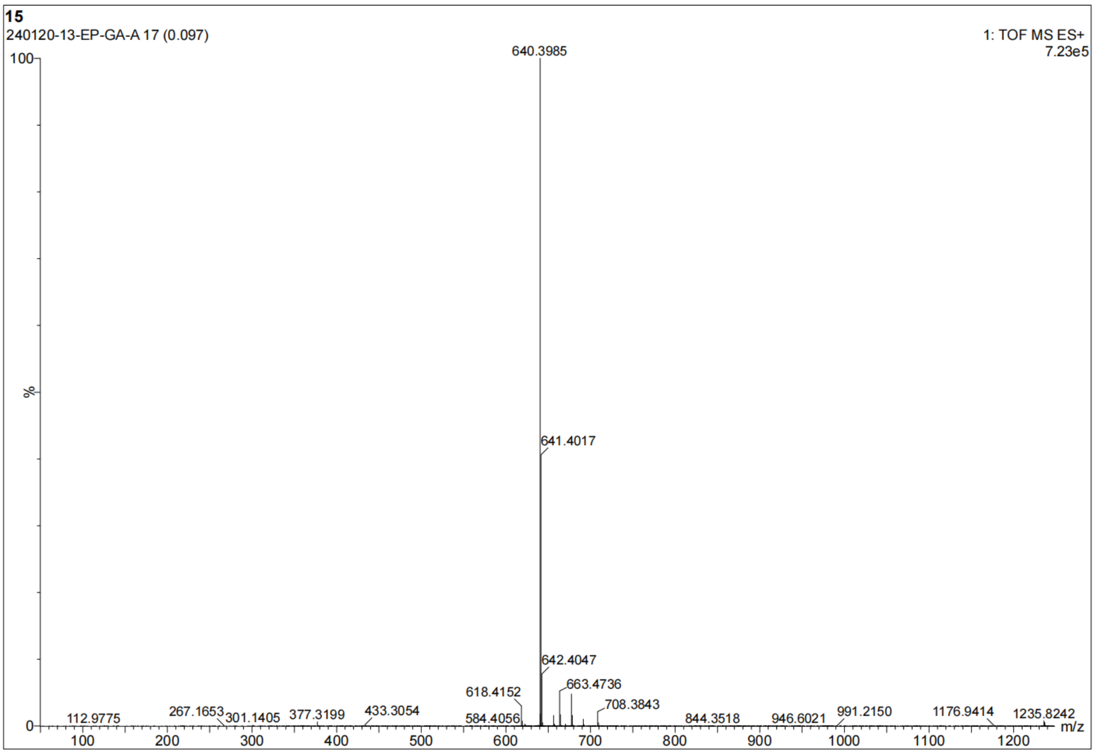

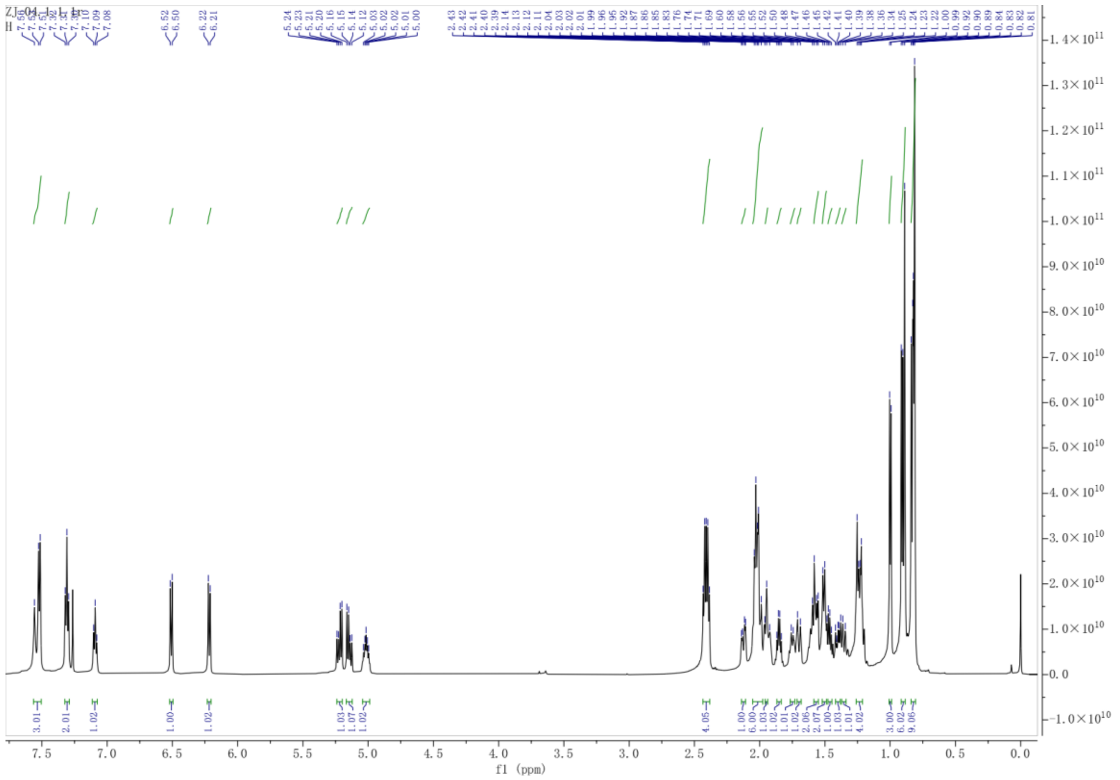

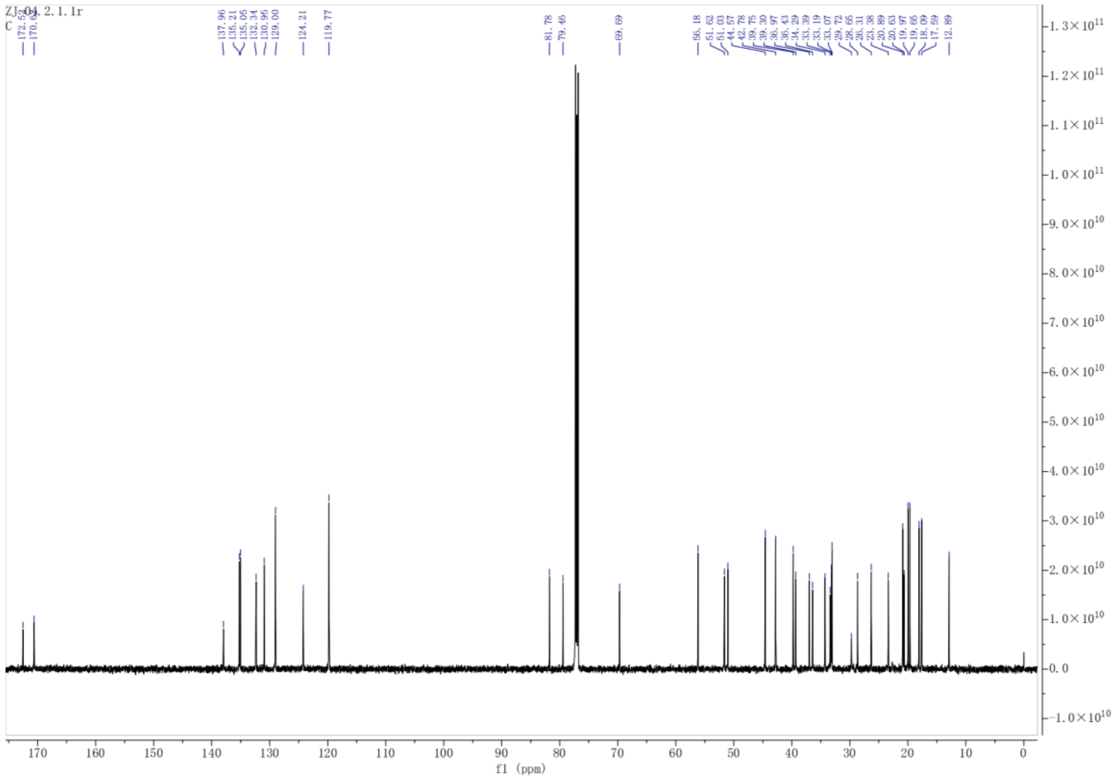

^{1}H NMR, ^{13}C NMR and HRMS spectra of **3f**

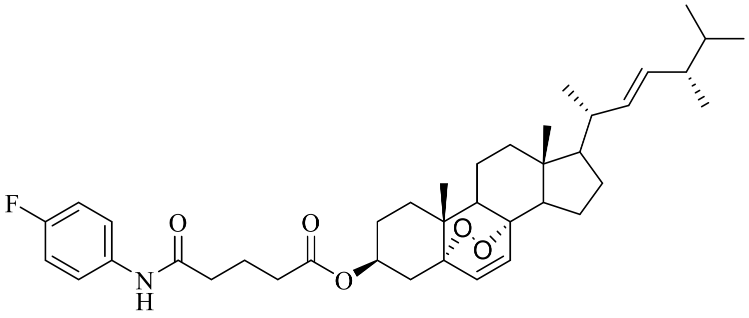

3f

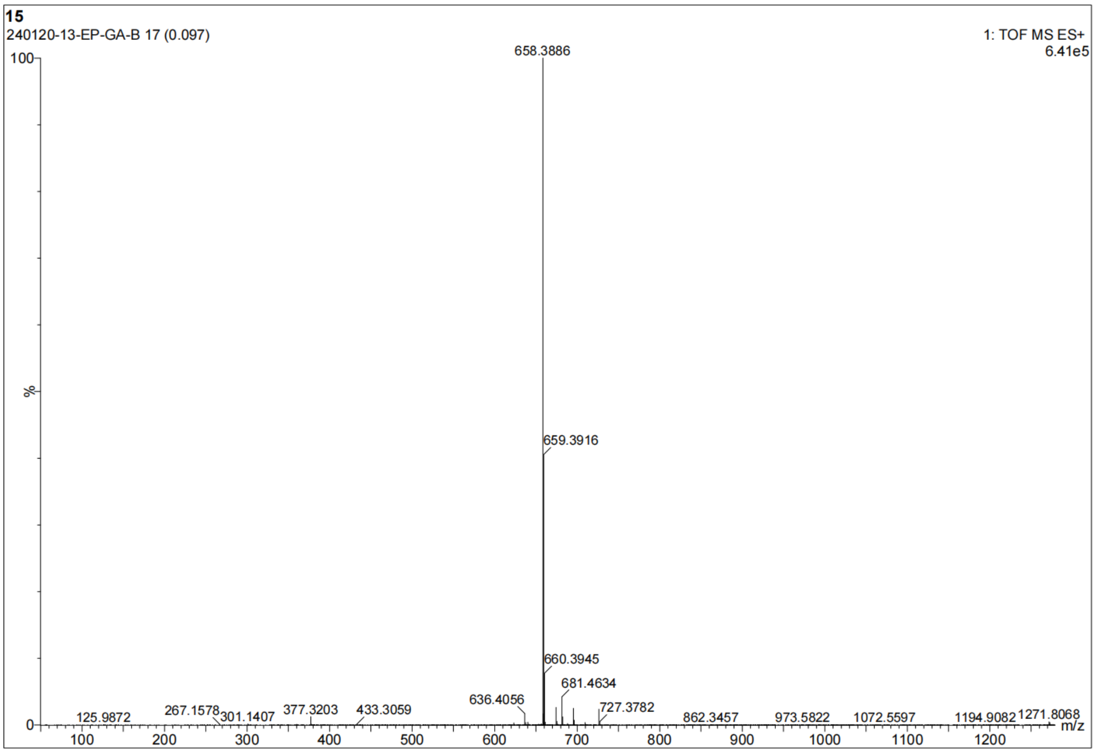

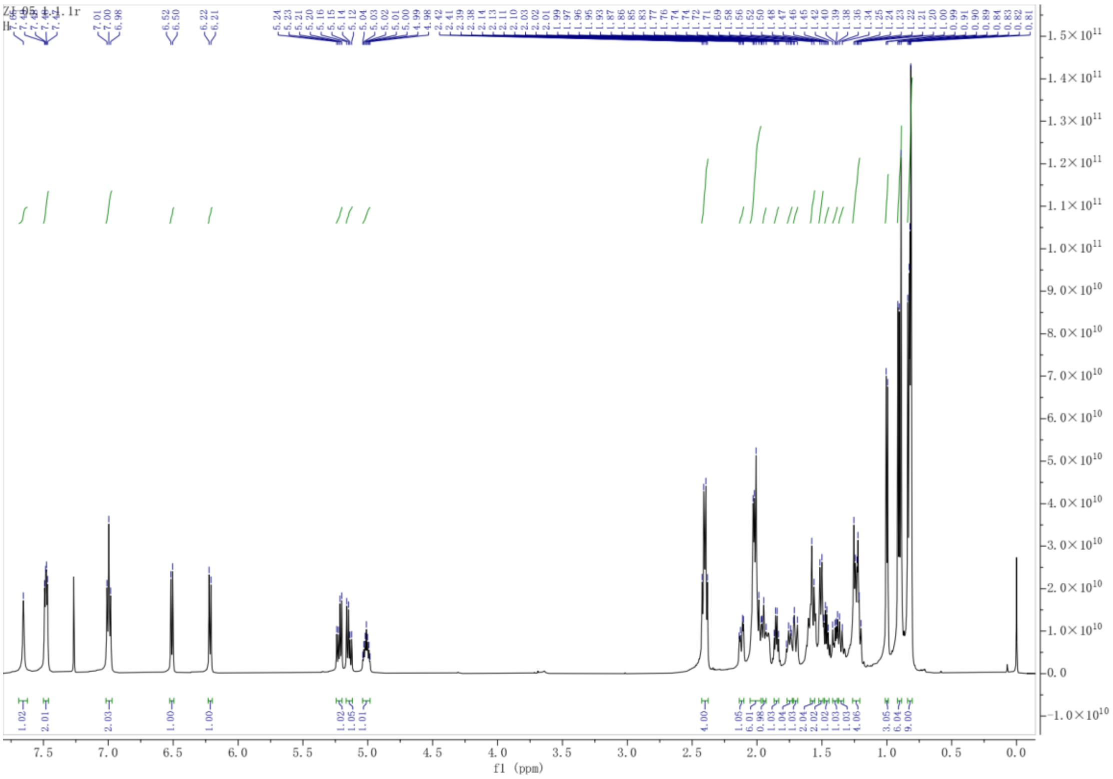

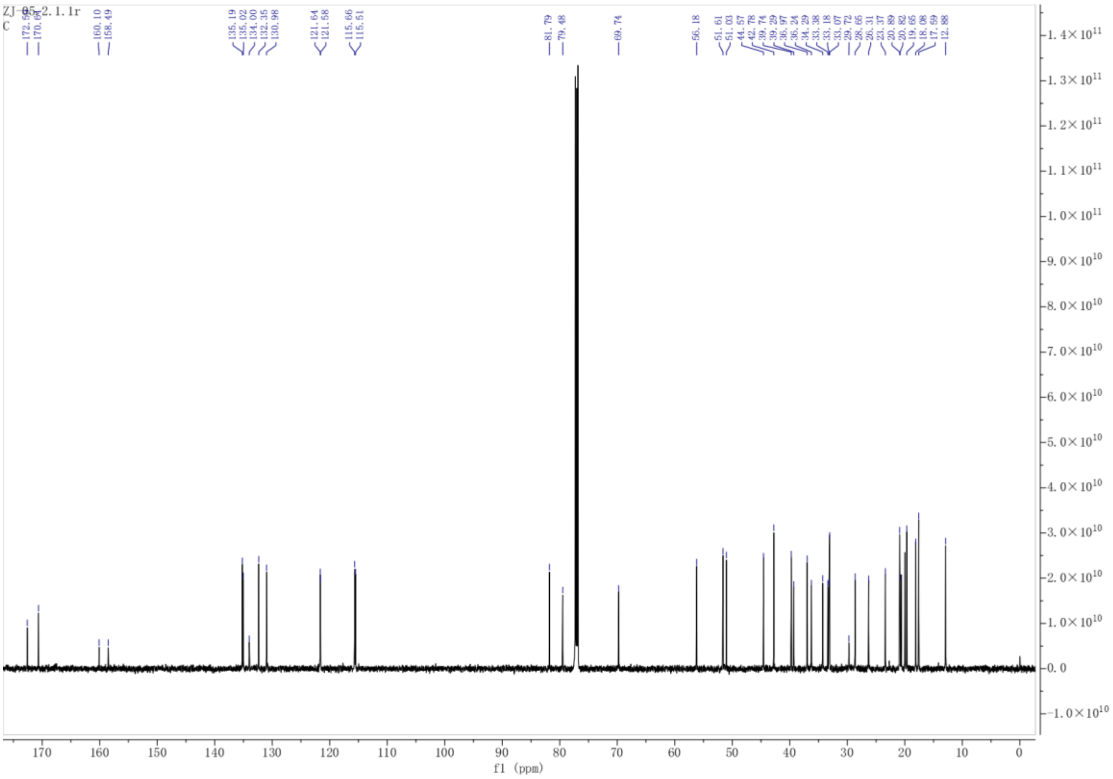

^{1}H NMR, ^{13}C NMR and HRMS spectra of **3g**

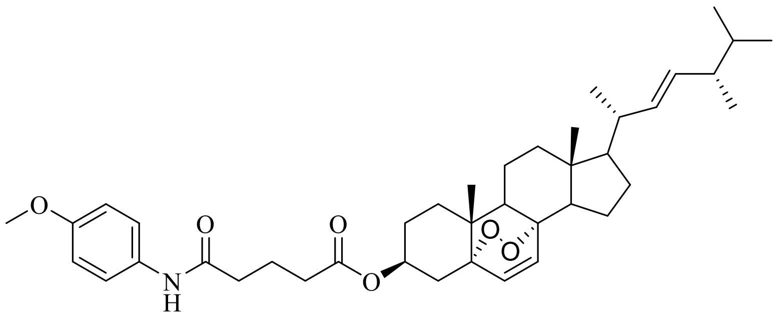

3g

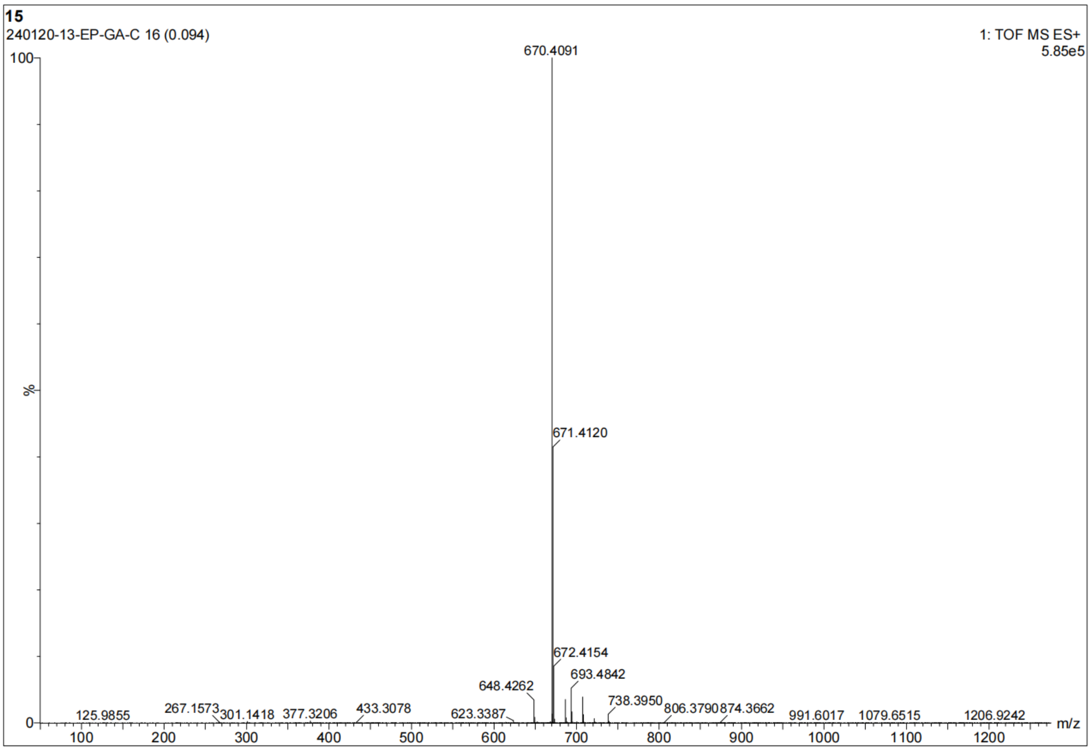

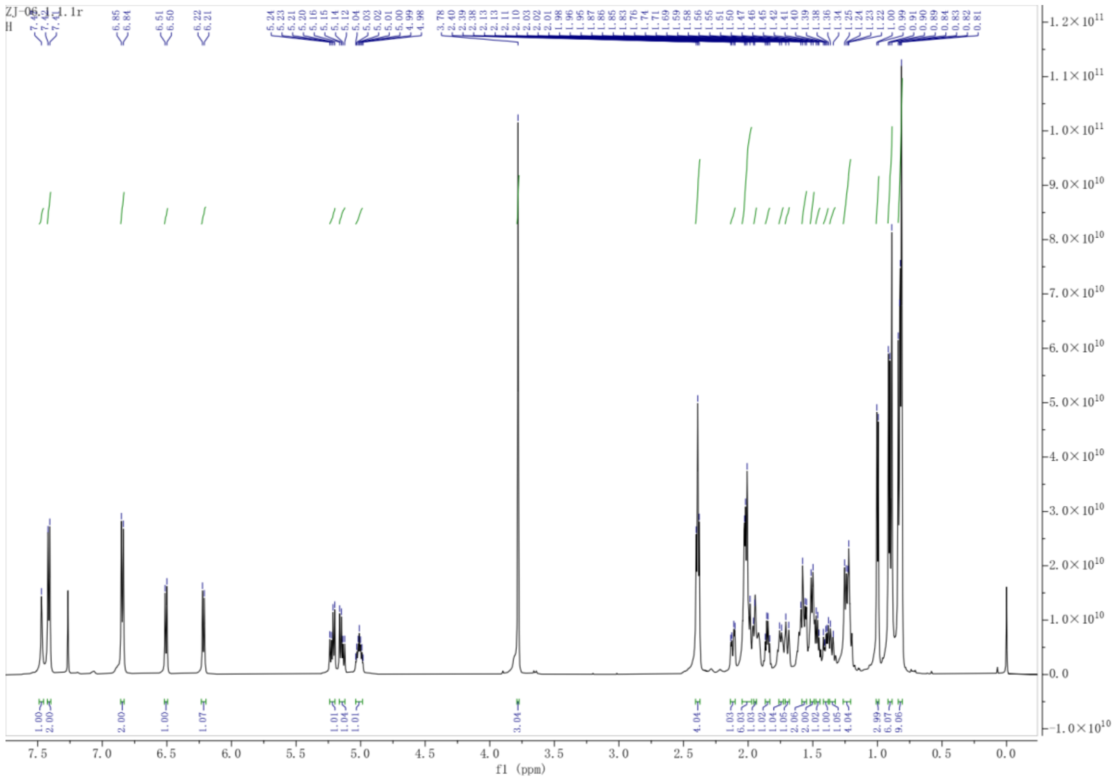
ZJ-06-1-1.1r
H
f1 (ppm)

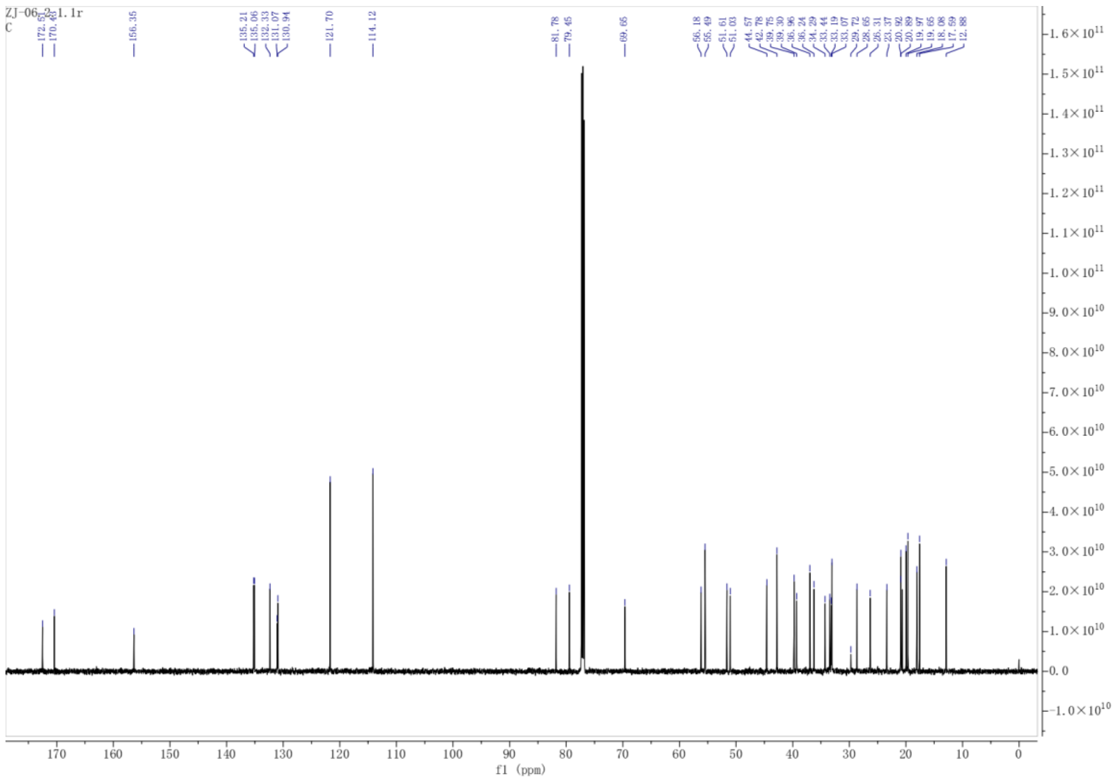
ZJ-06-2-1.1r
C
f1 (ppm)

^{1}H NMR, ^{13}C NMR and HRMS spectra of **3h**

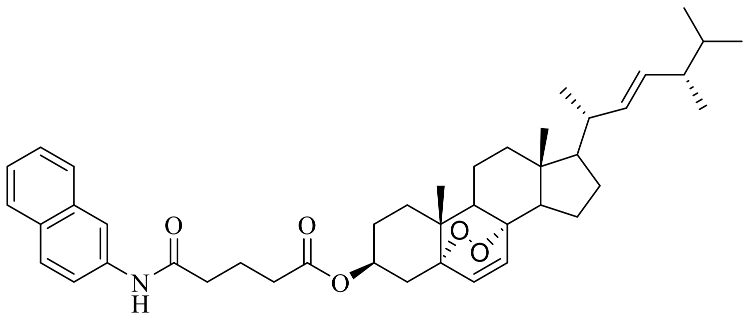

3h

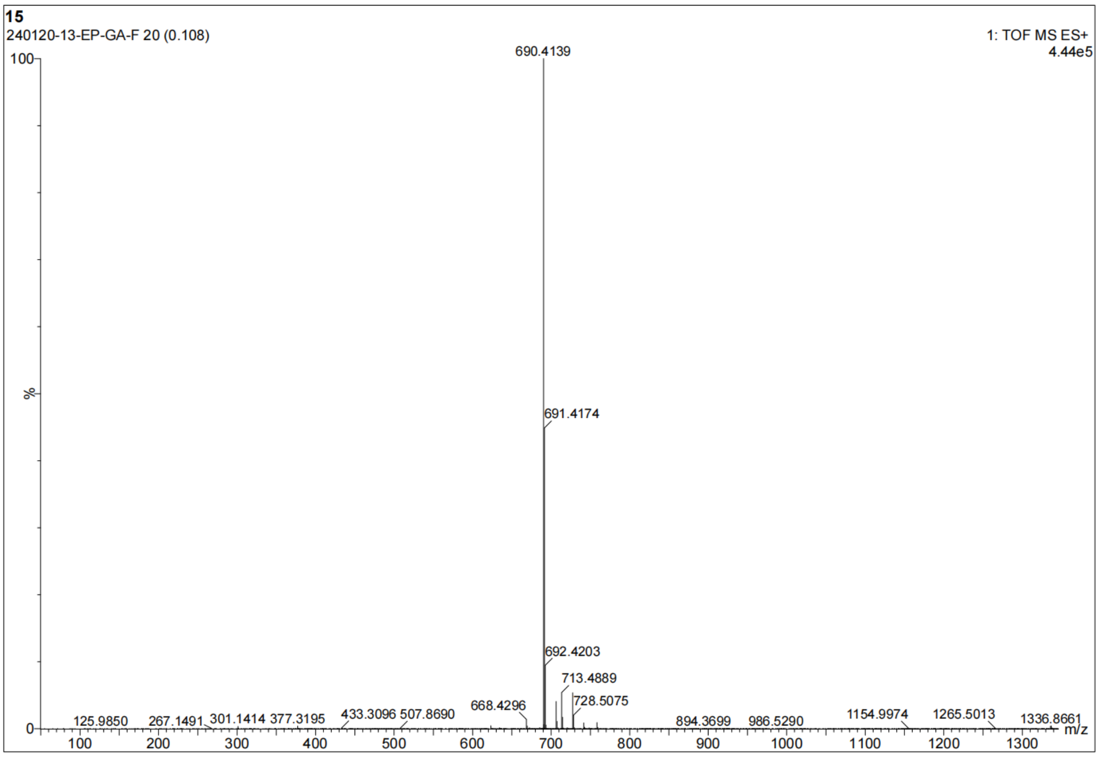

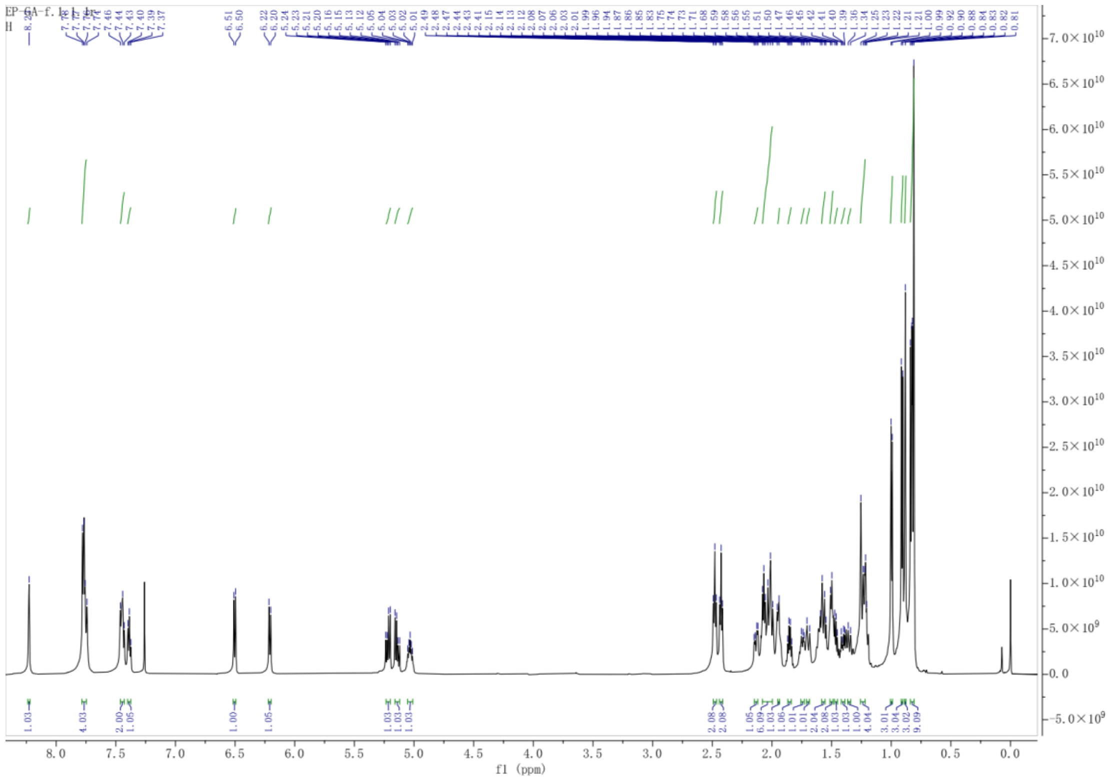

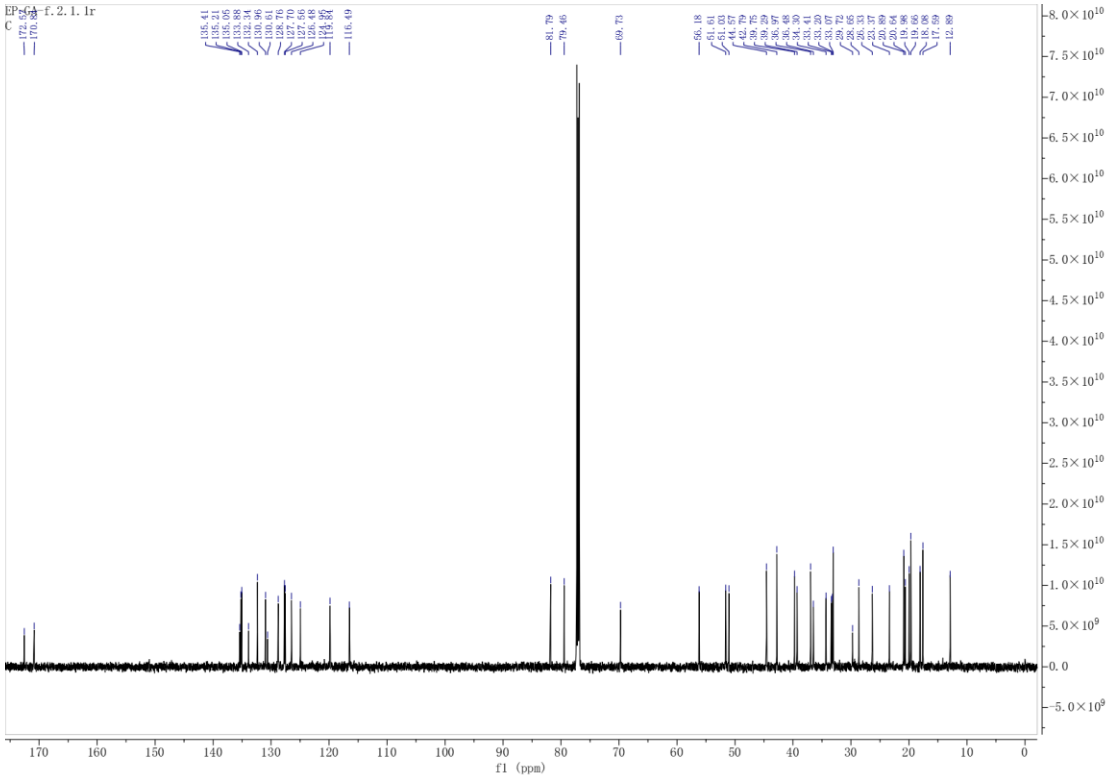

^{1}H NMR and ^{13}C NMR of **4**

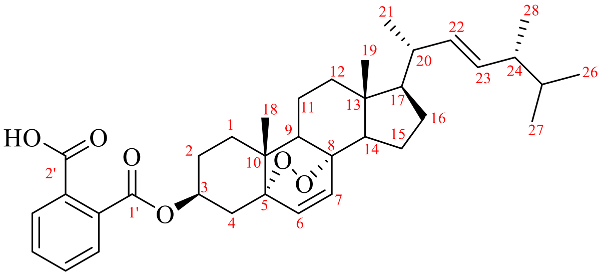

4

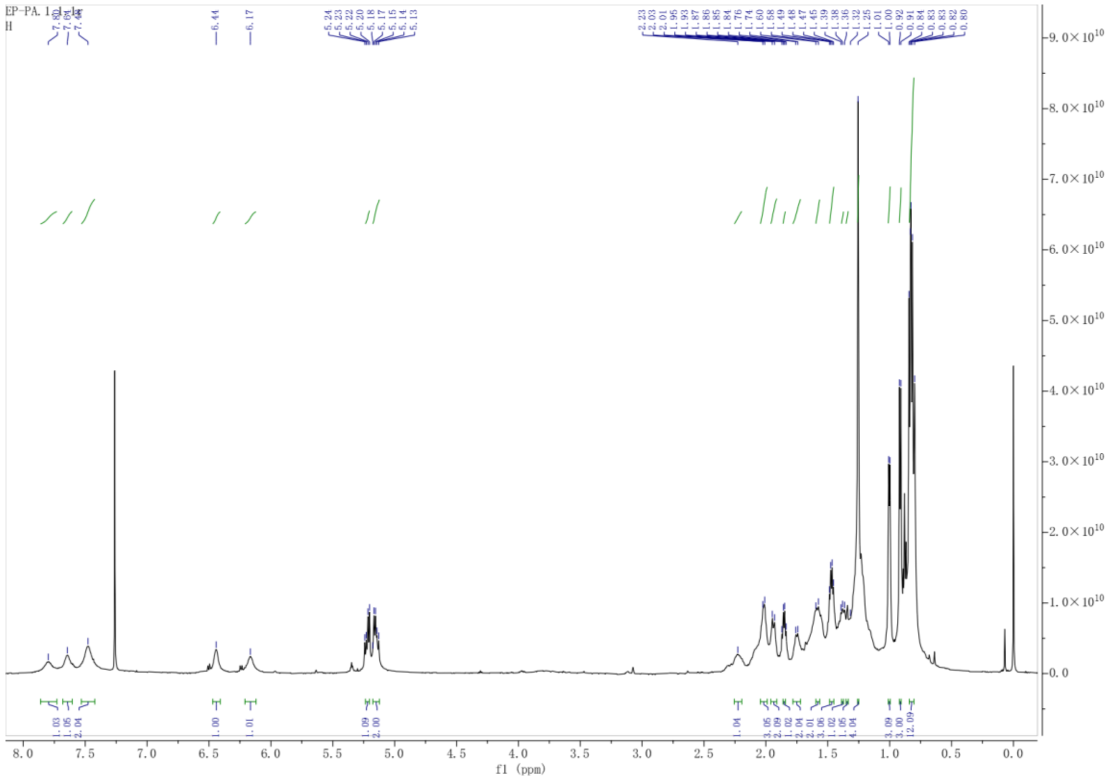

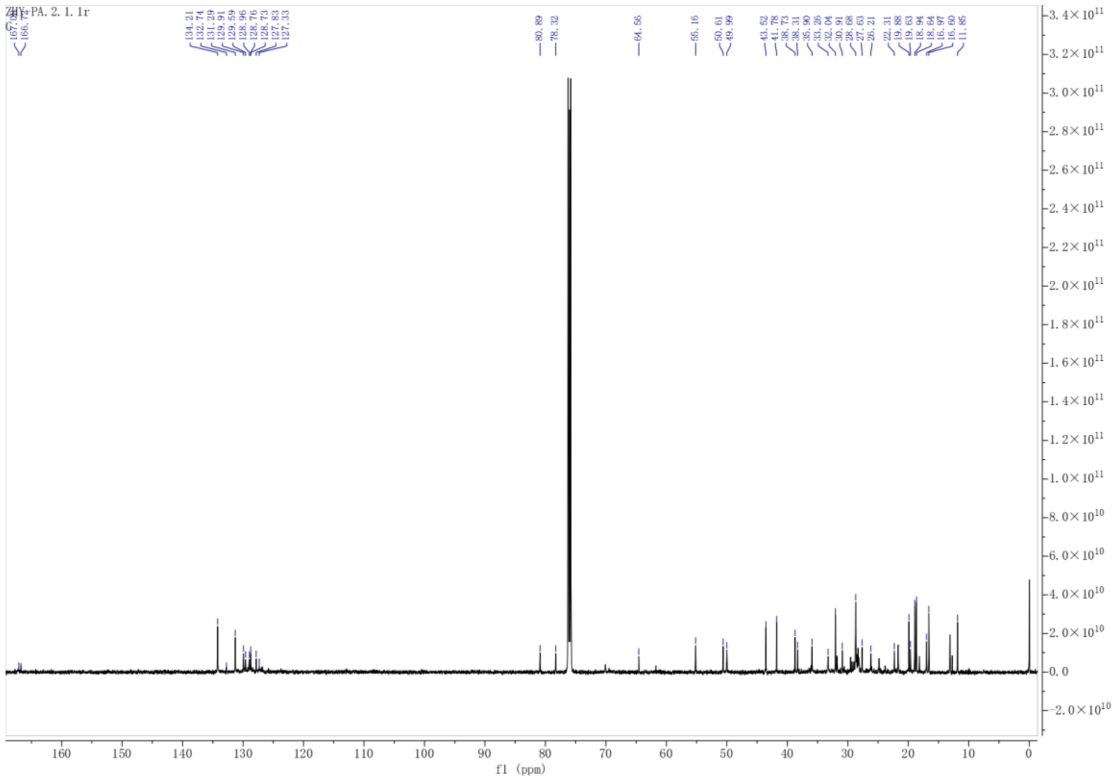

^{1}H NMR, ^{13}C NMR and HRMS spectra of **4a**

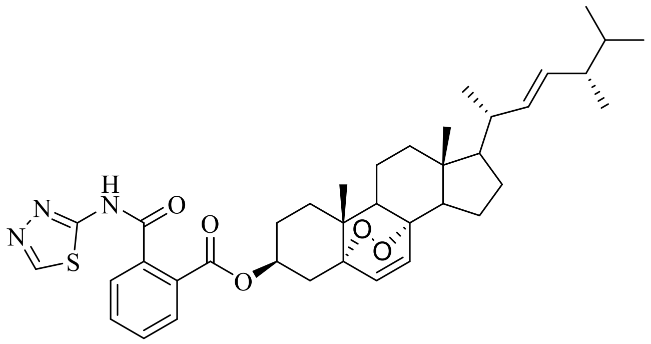

4a

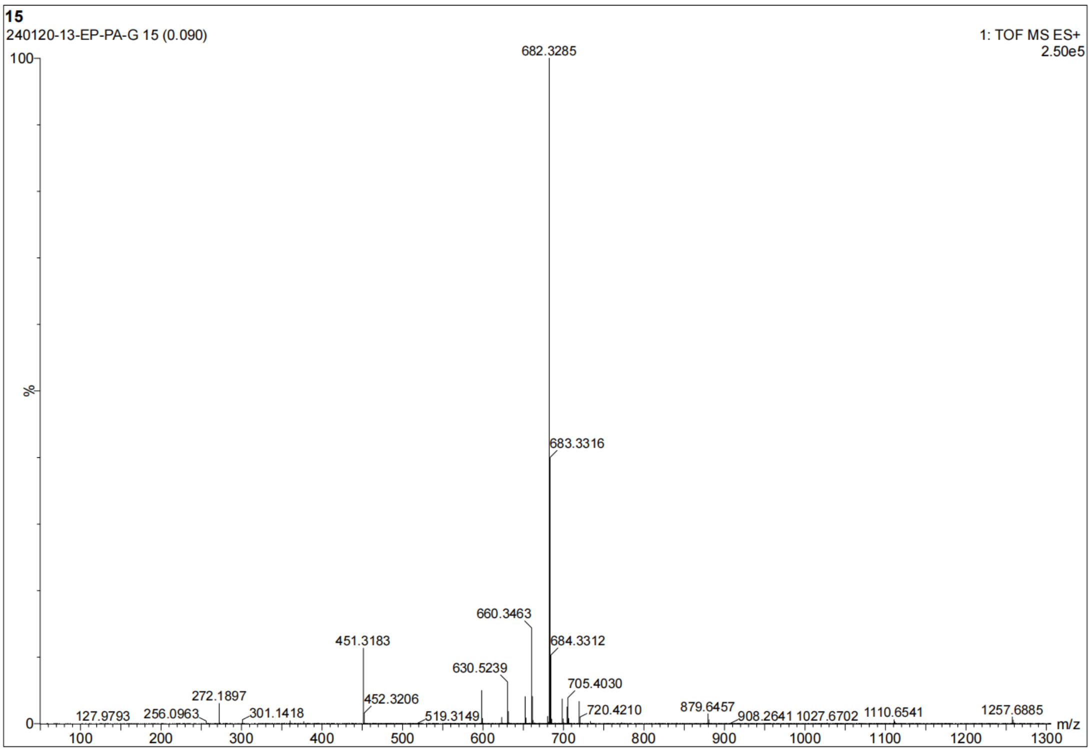

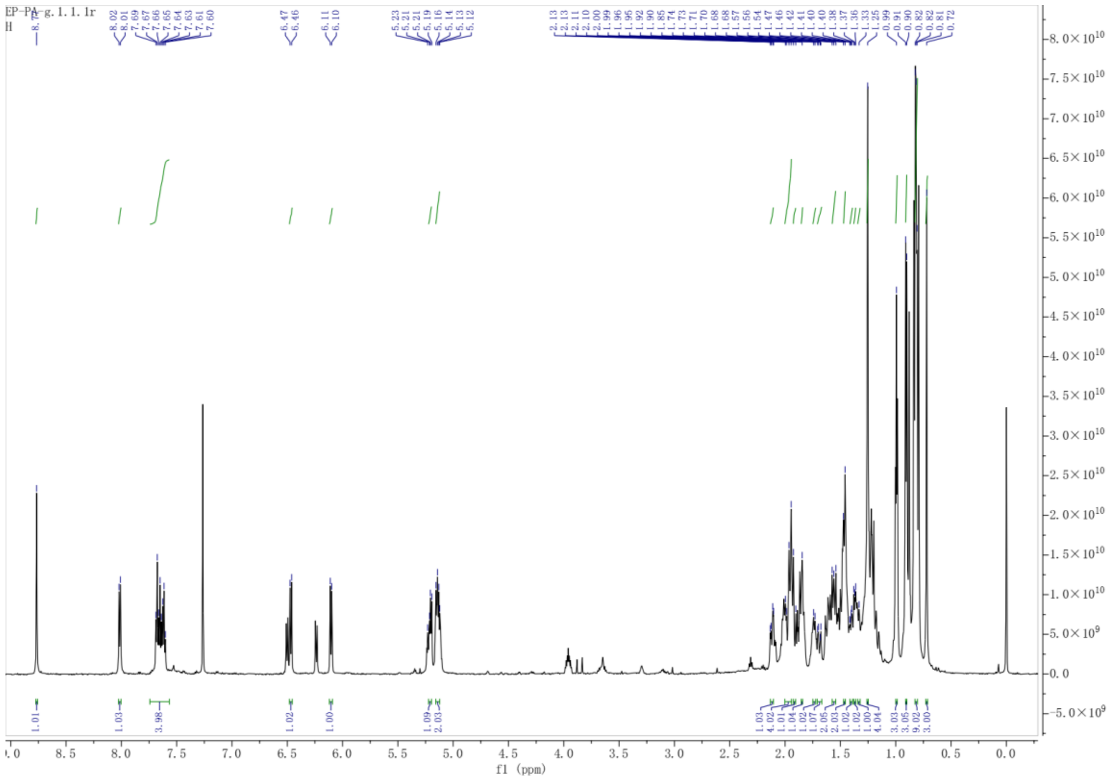

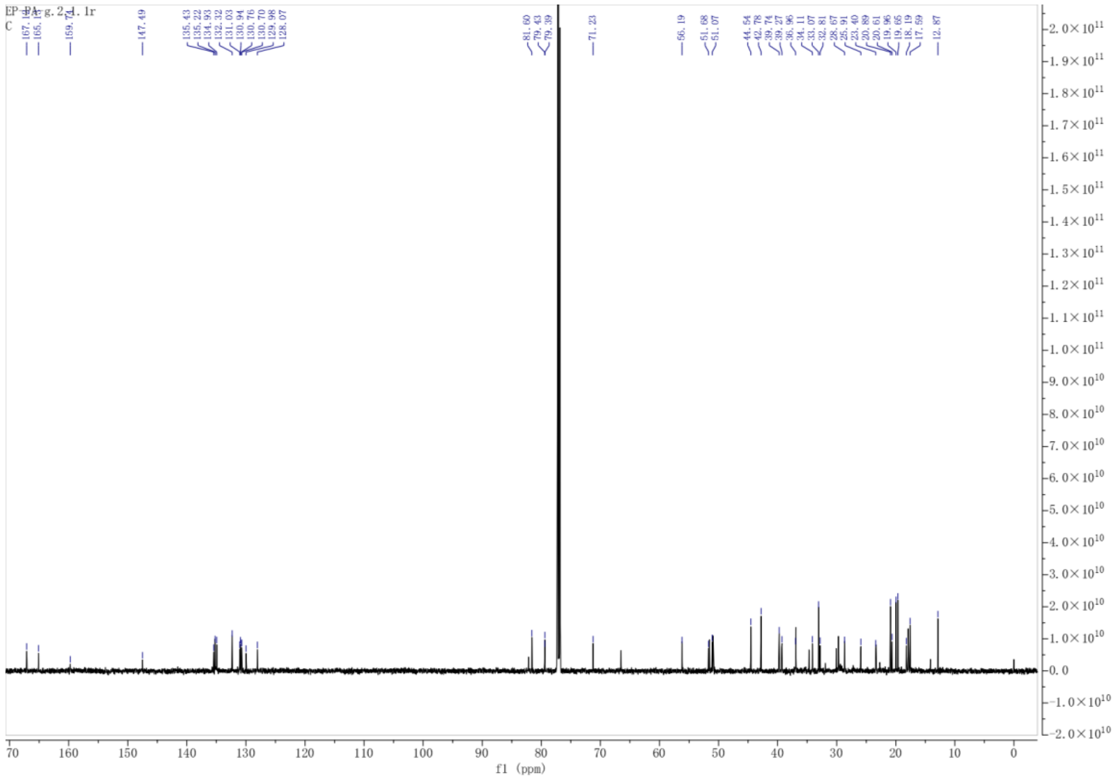

^{1}H NMR, ^{13}C NMR and HRMS spectra of **4b**

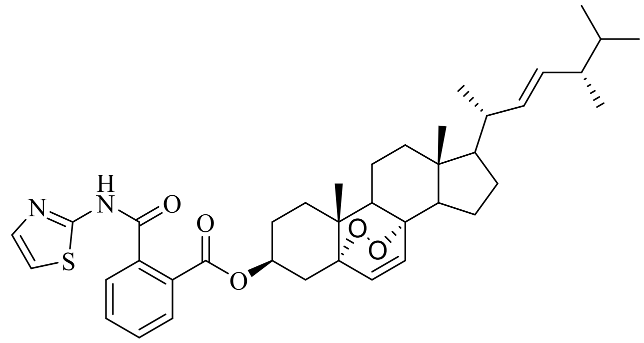

4b

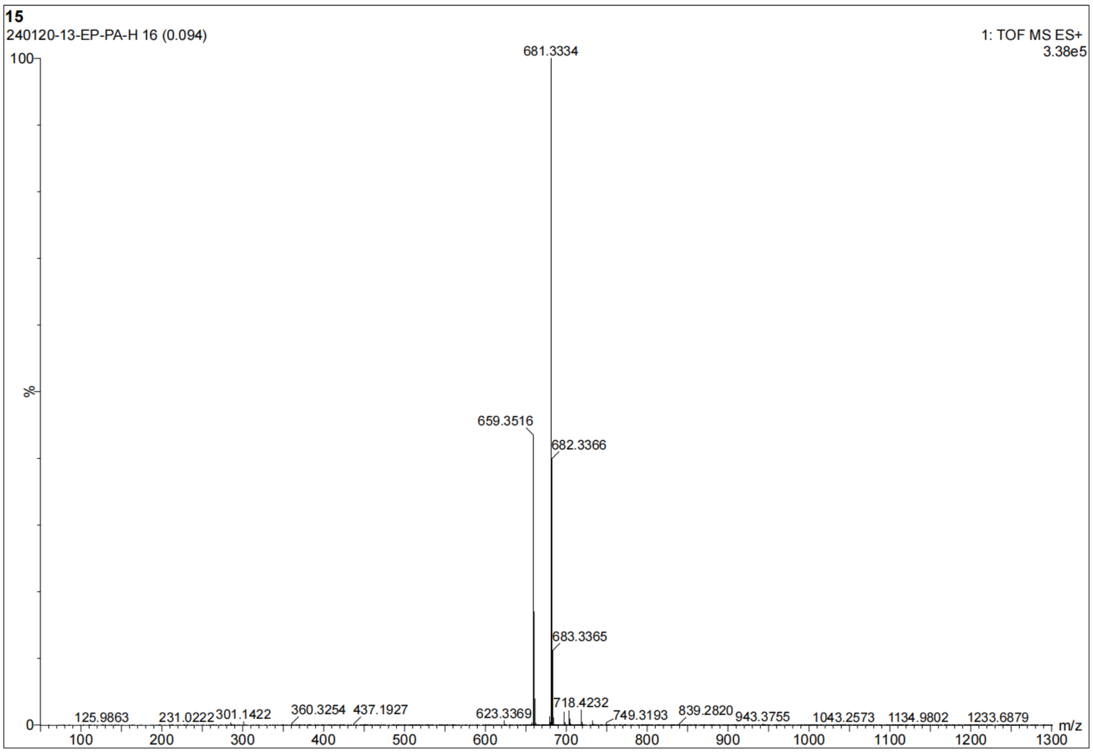

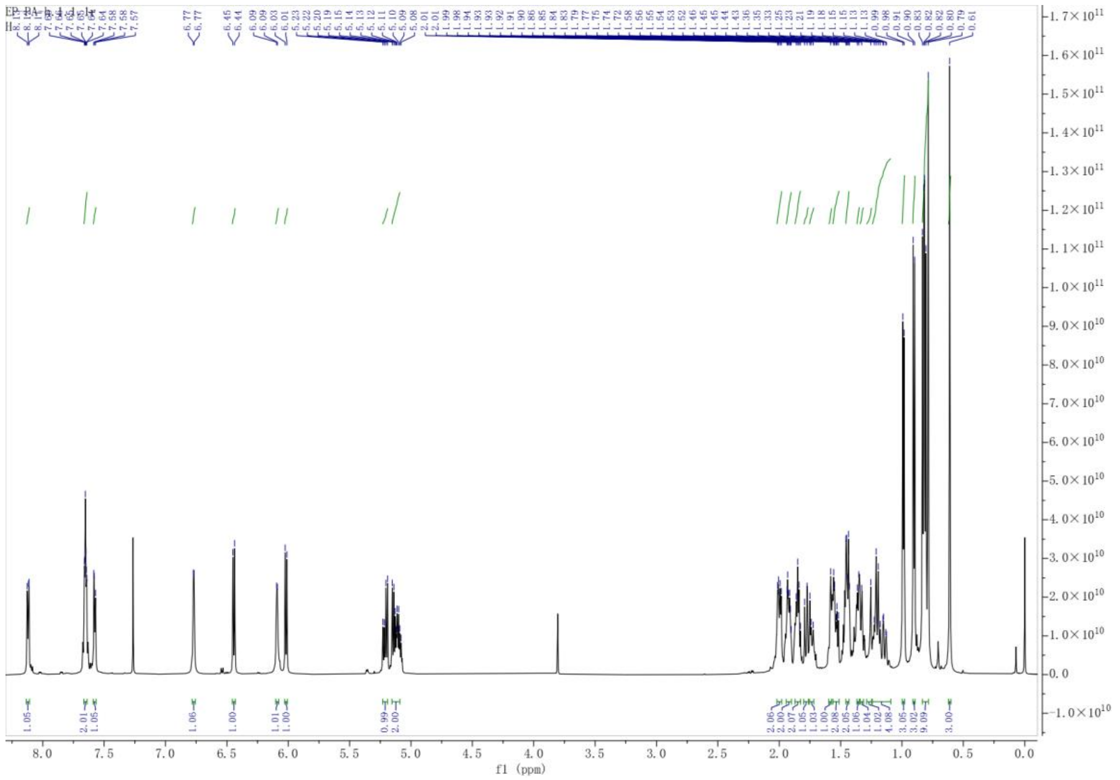

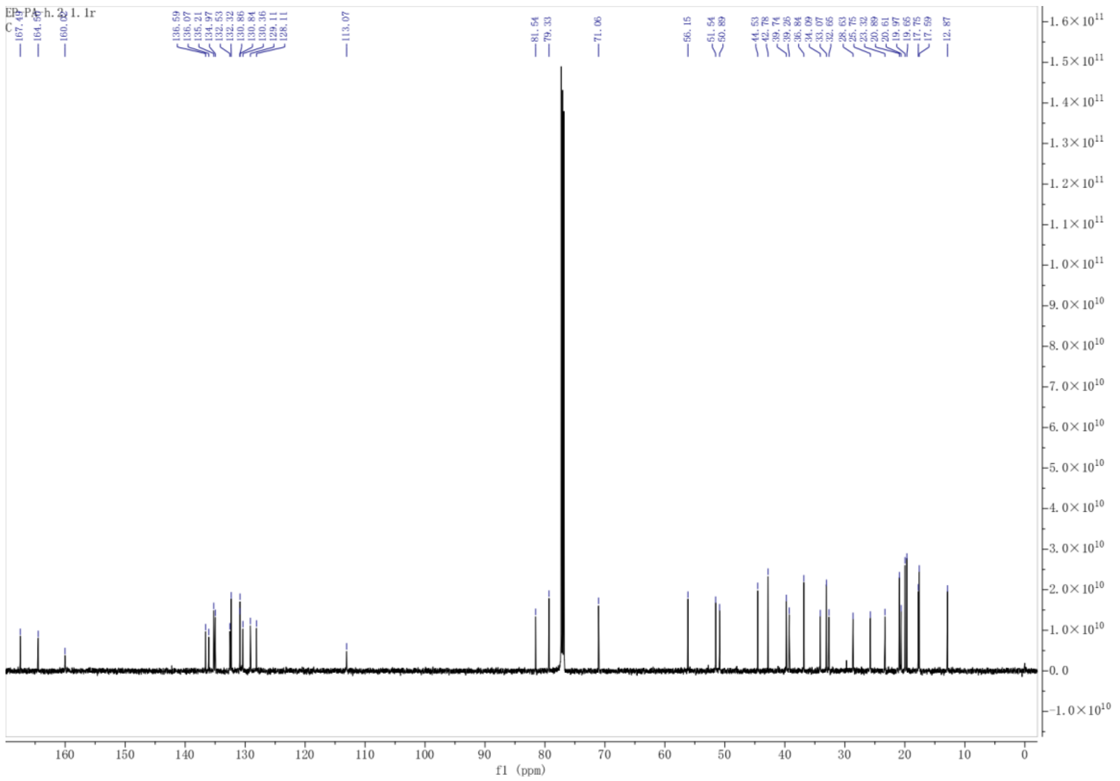

^{1}H NMR, ^{13}C NMR and HRMS spectra of **4c**

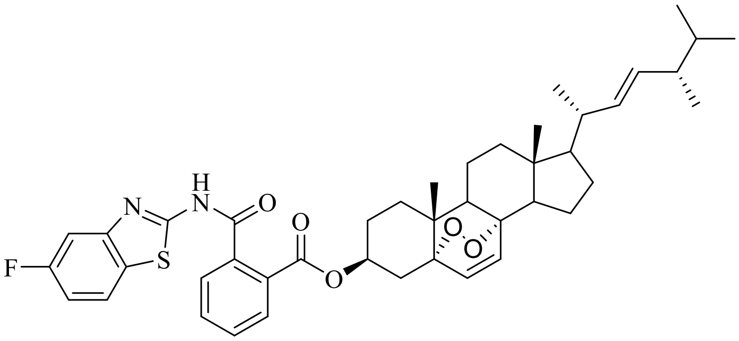

4c

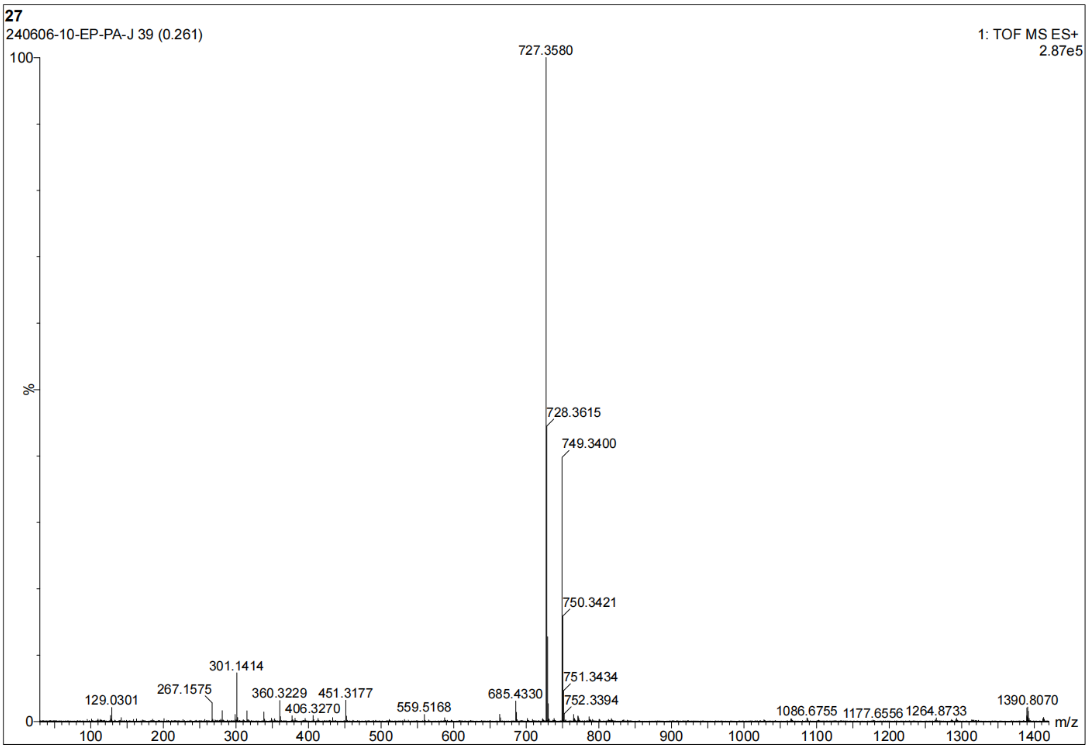

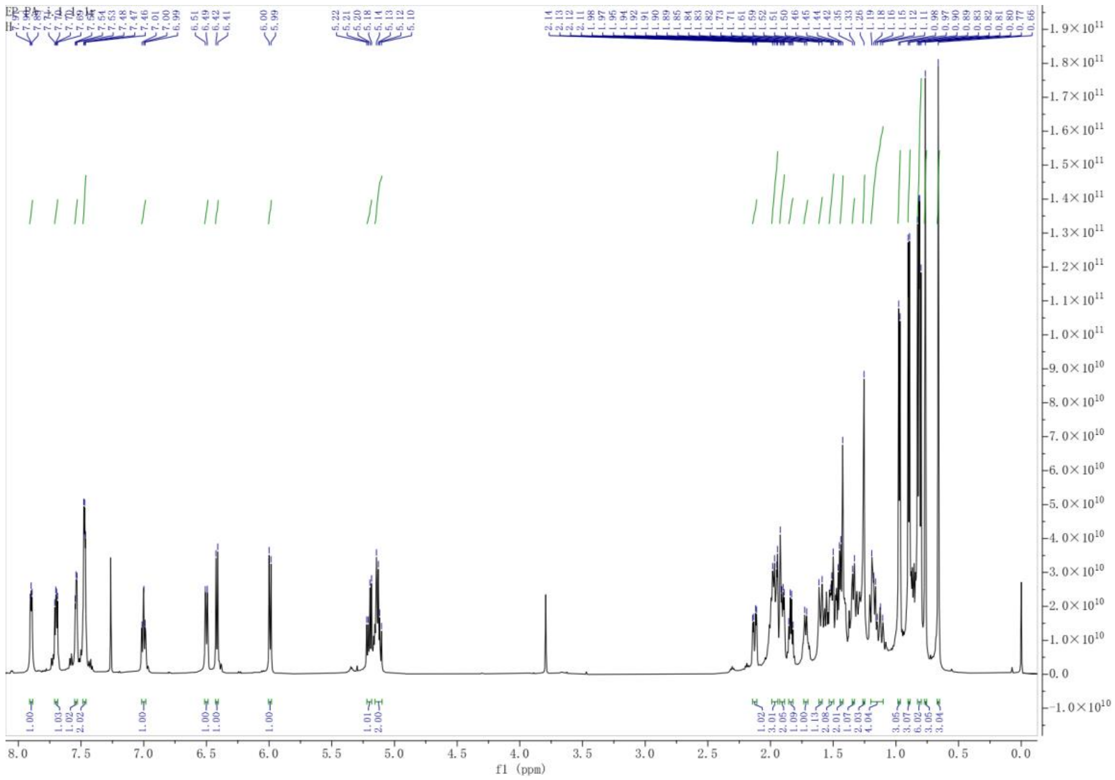

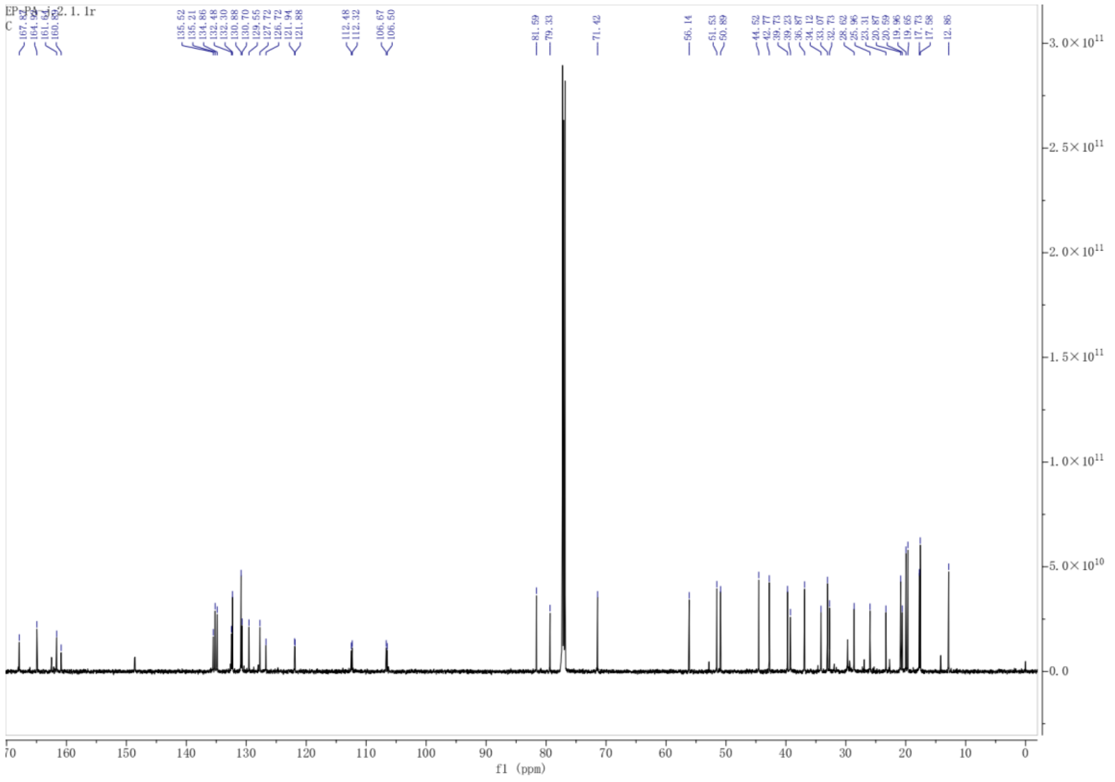

^{1}H NMR, ^{13}C NMR and HRMS spectra of **4d**

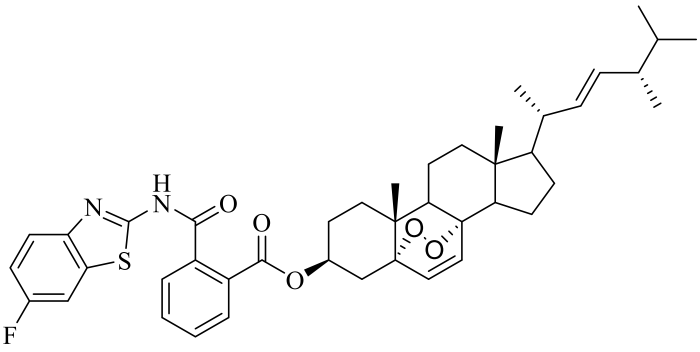

4d

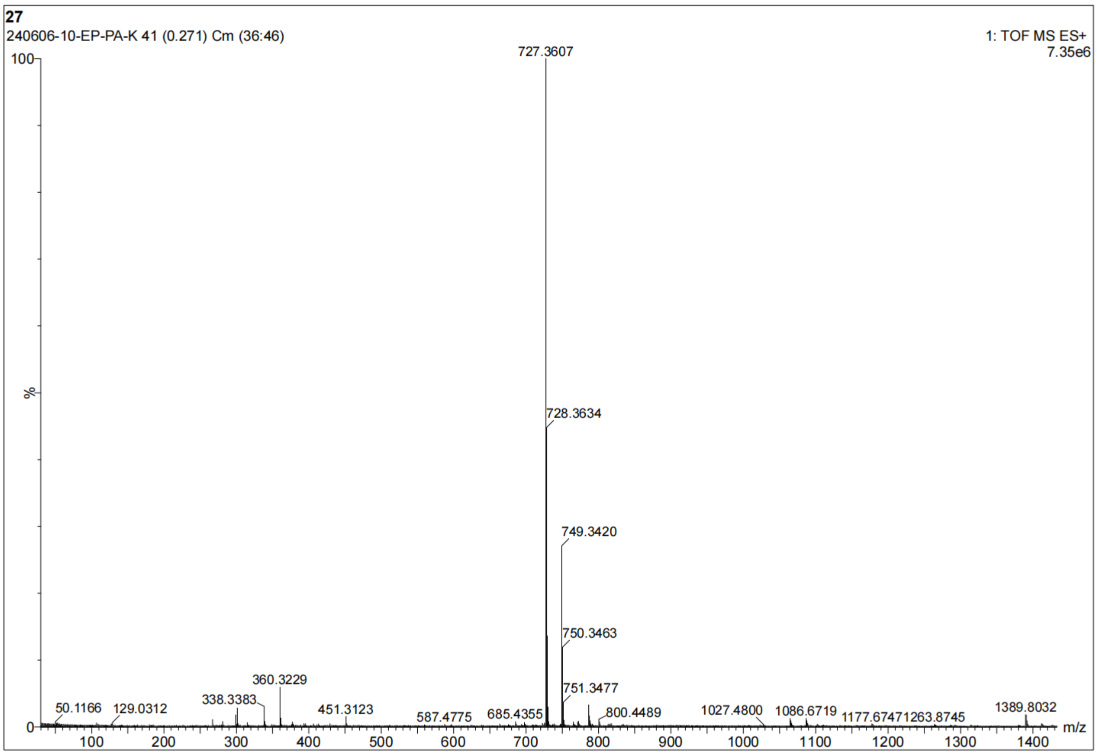

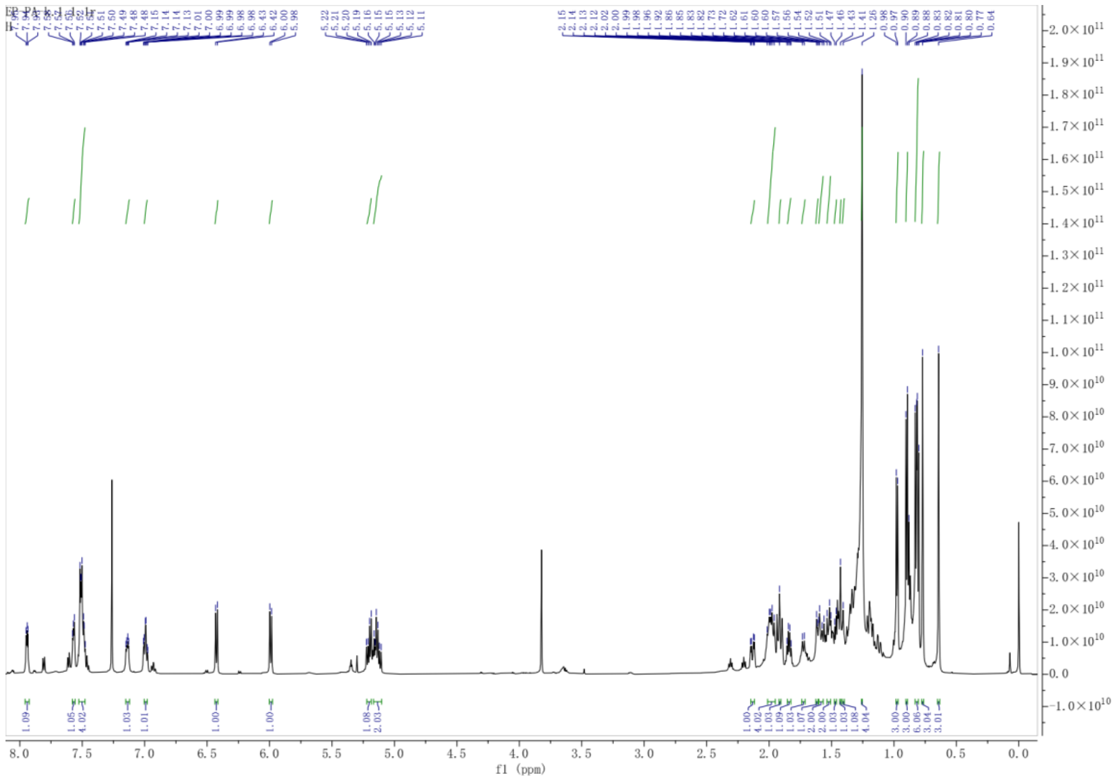

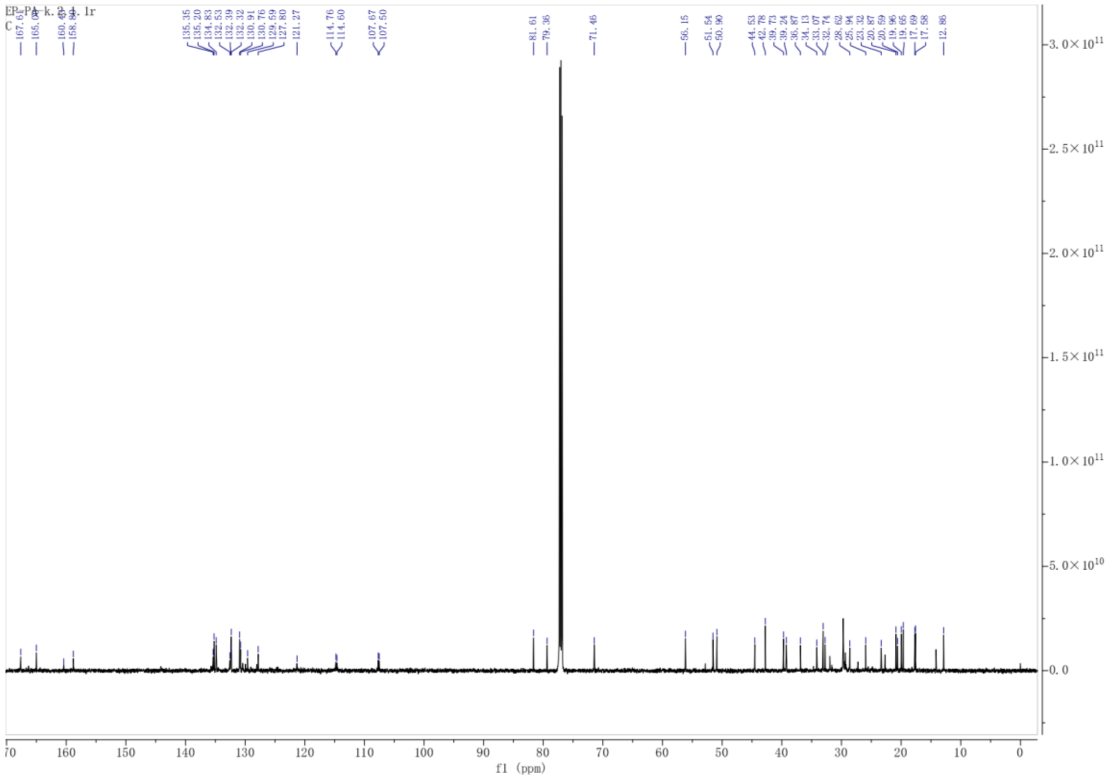

^{1}H NMR, ^{13}C NMR and HRMS spectra of **4e**

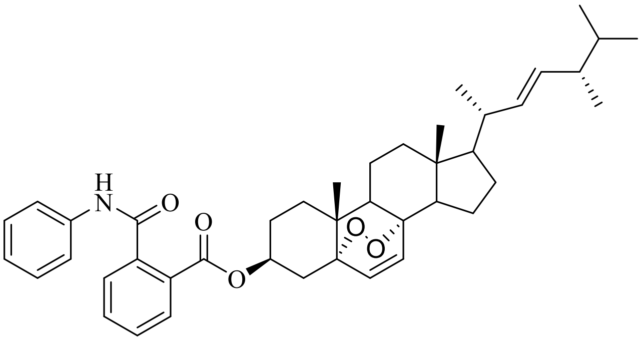

4e

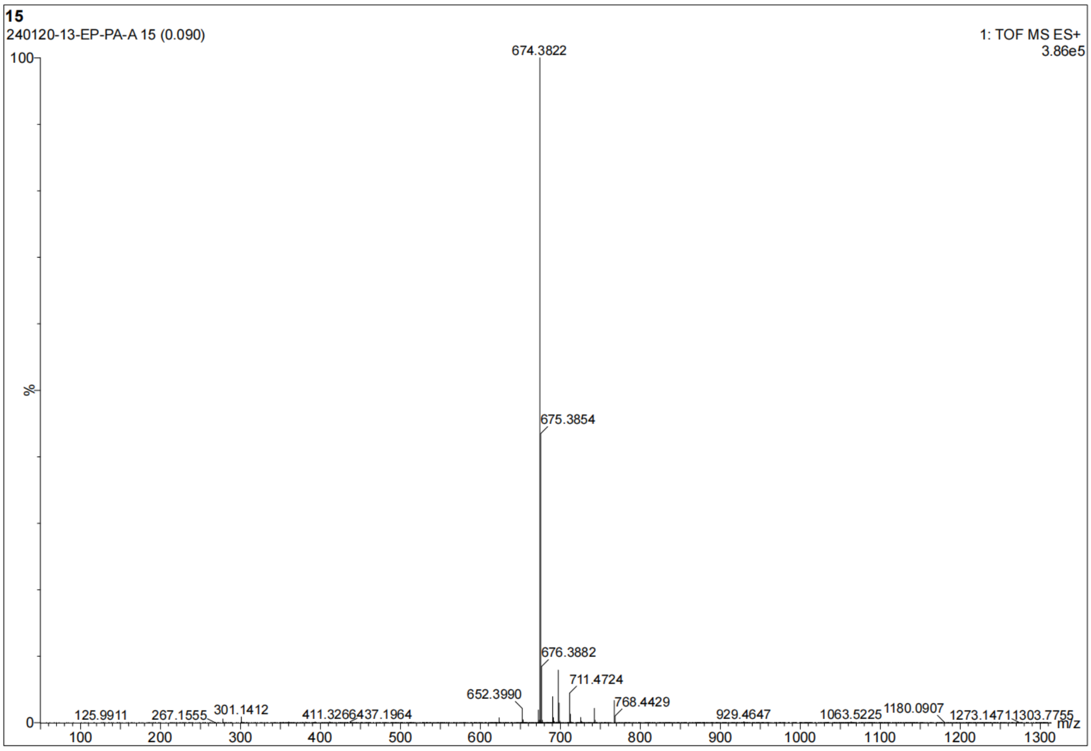

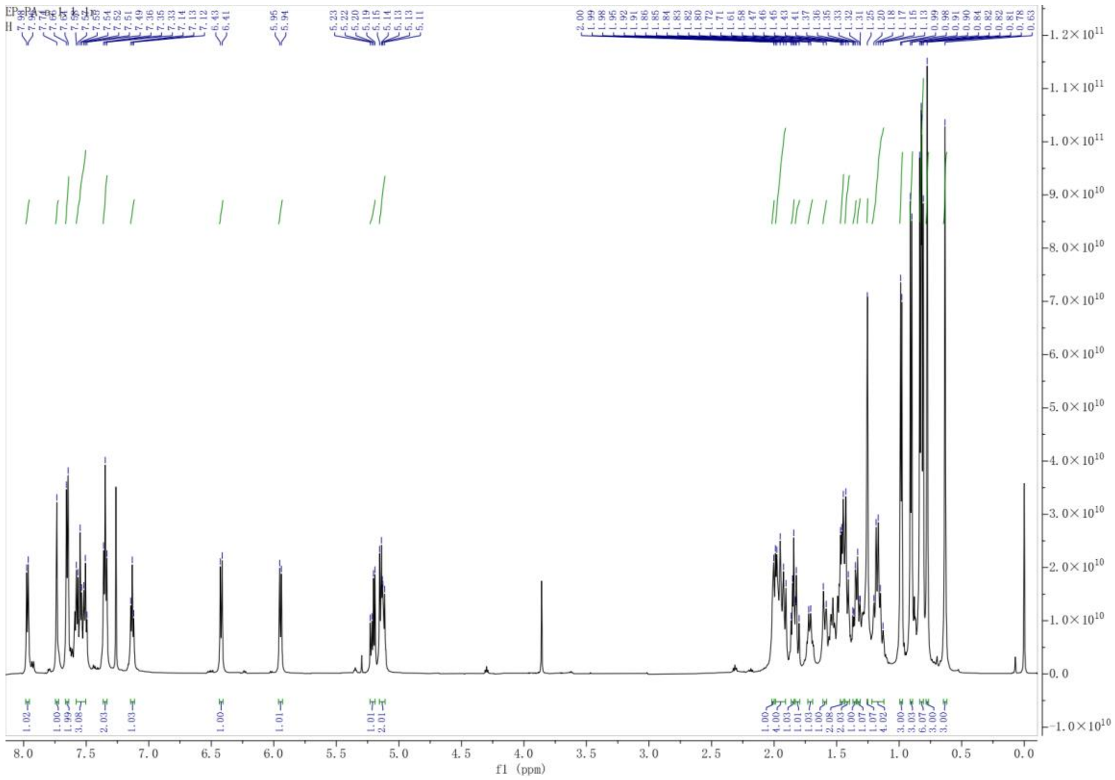

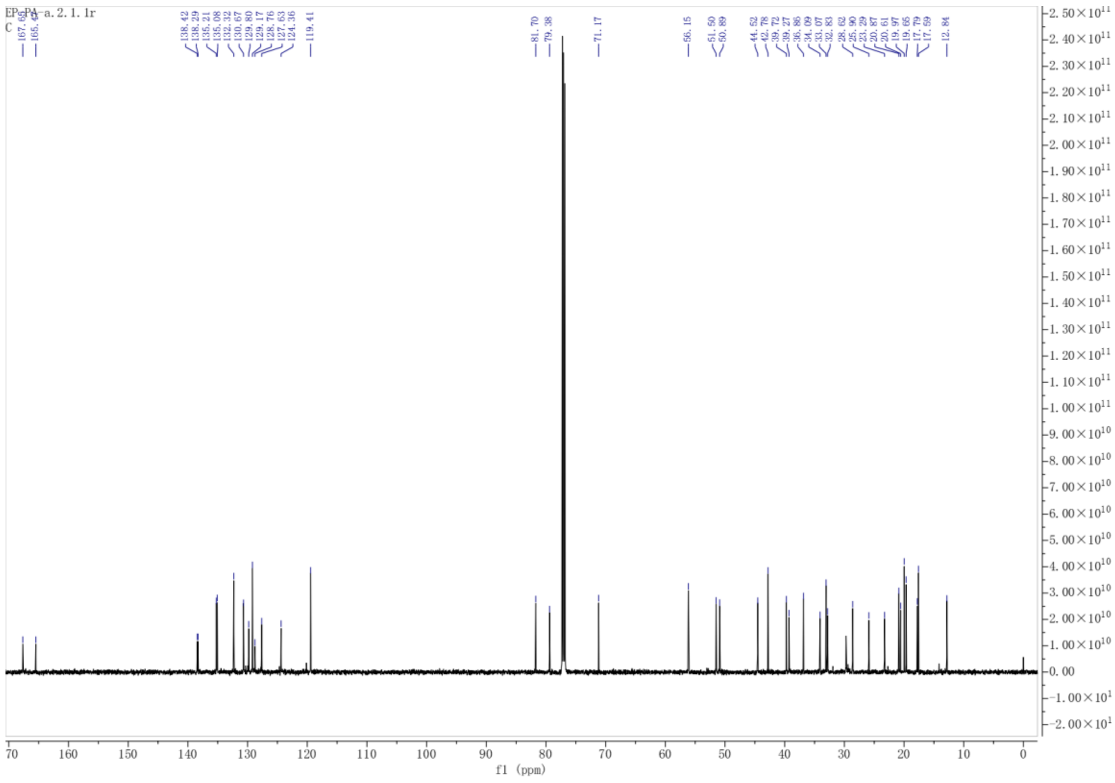

^{1}H NMR, ^{13}C NMR and HRMS spectra of **4f**

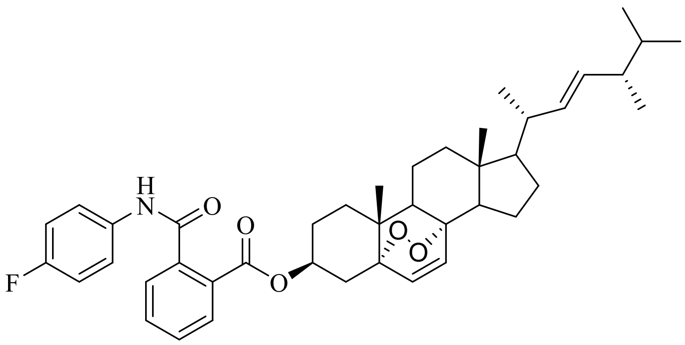

4f

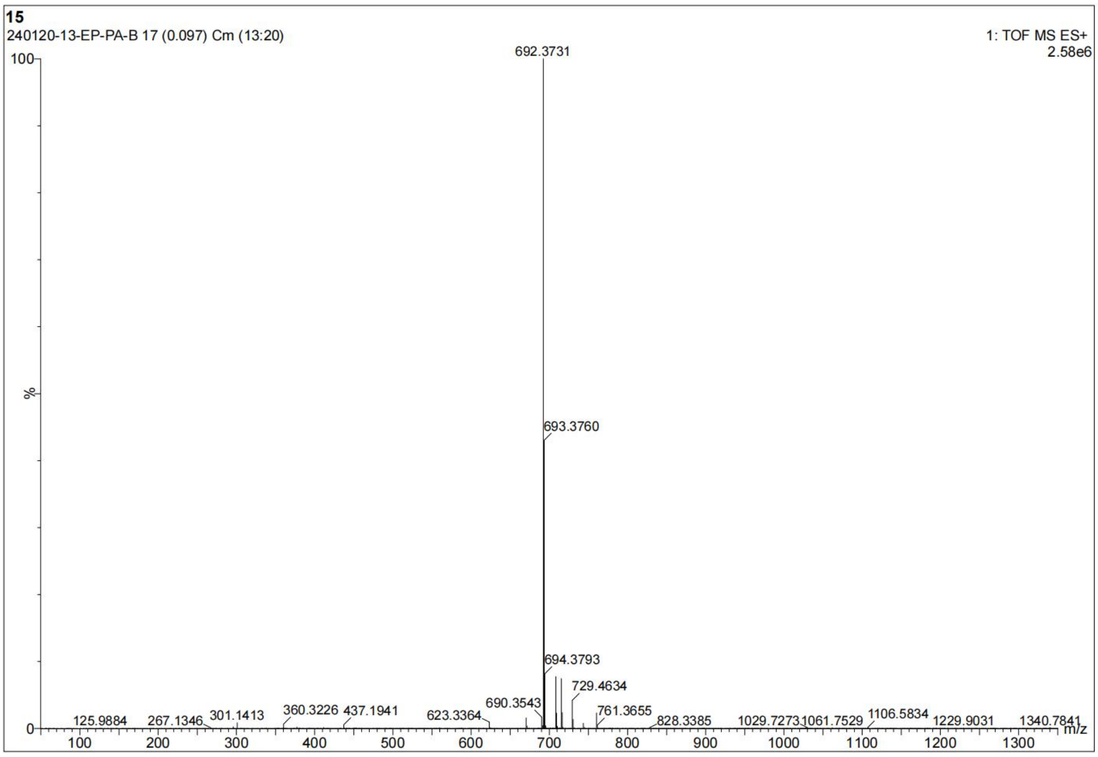

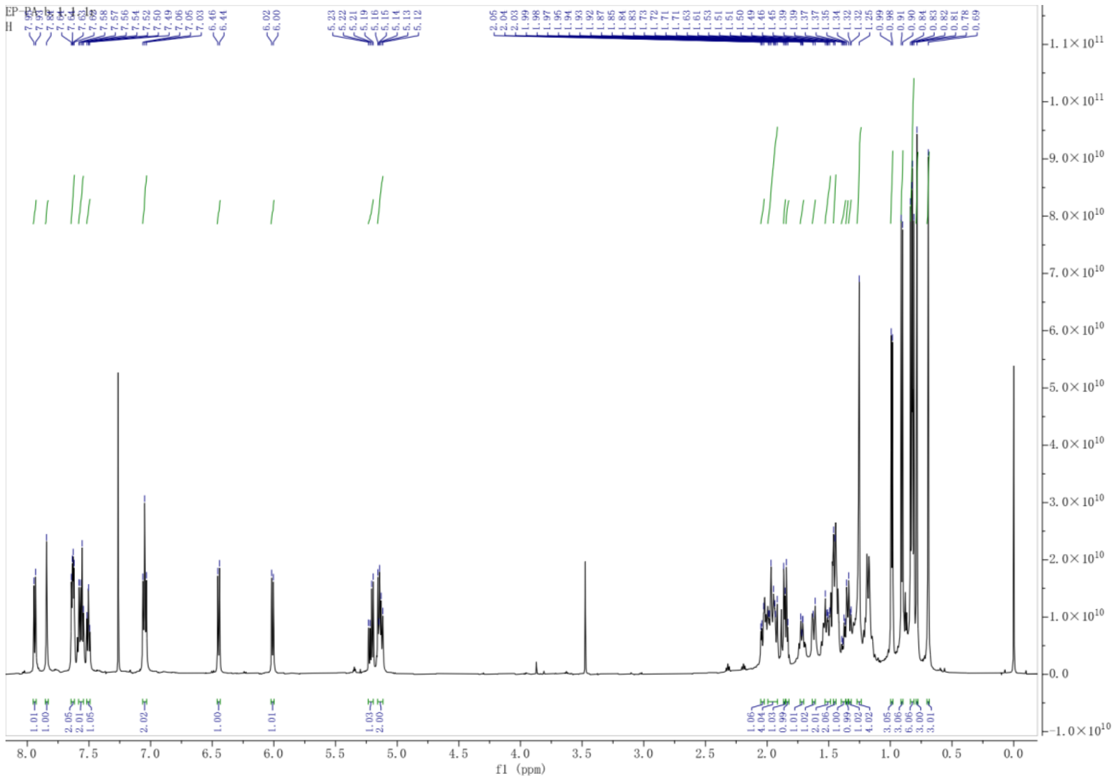

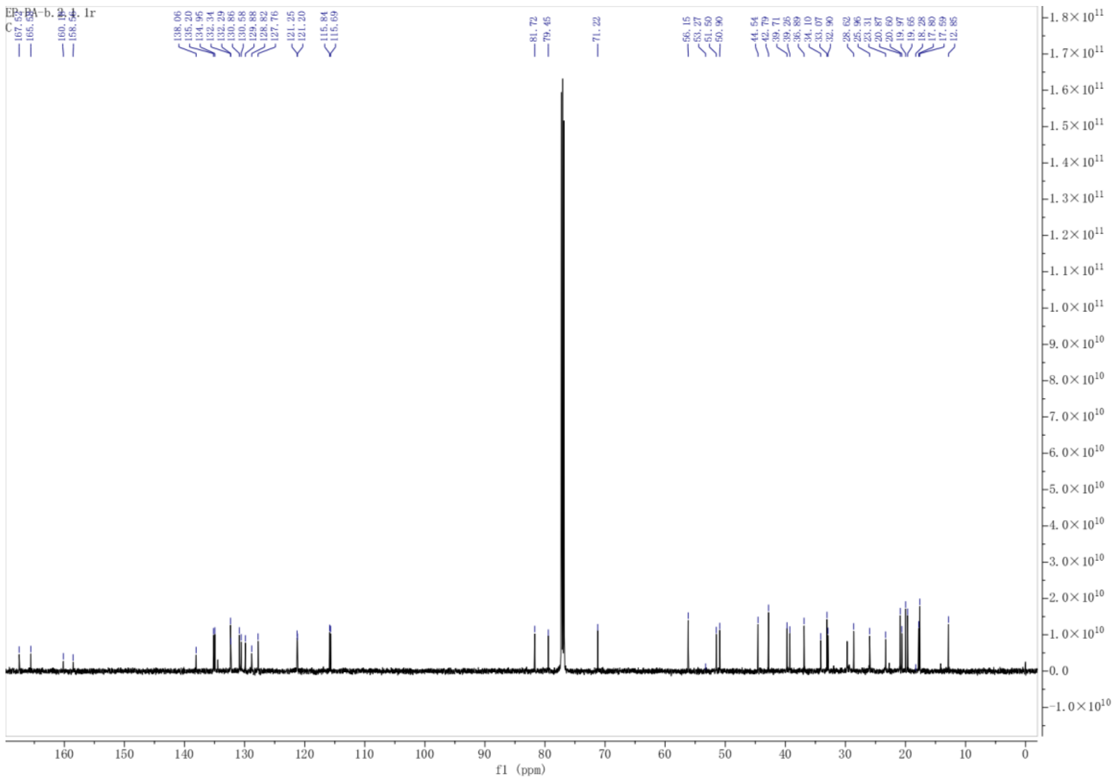

^{1}H NMR, ^{13}C NMR and HRMS spectra of **4g**

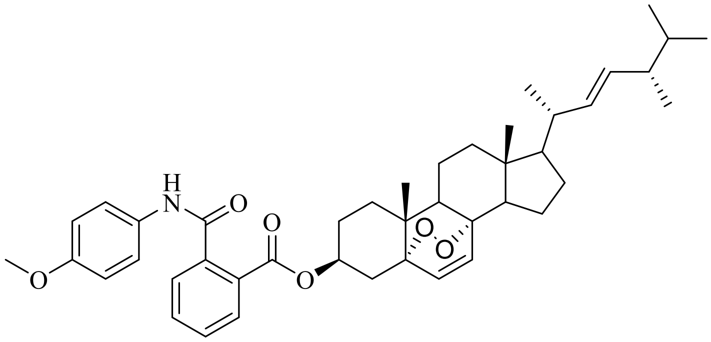

4g

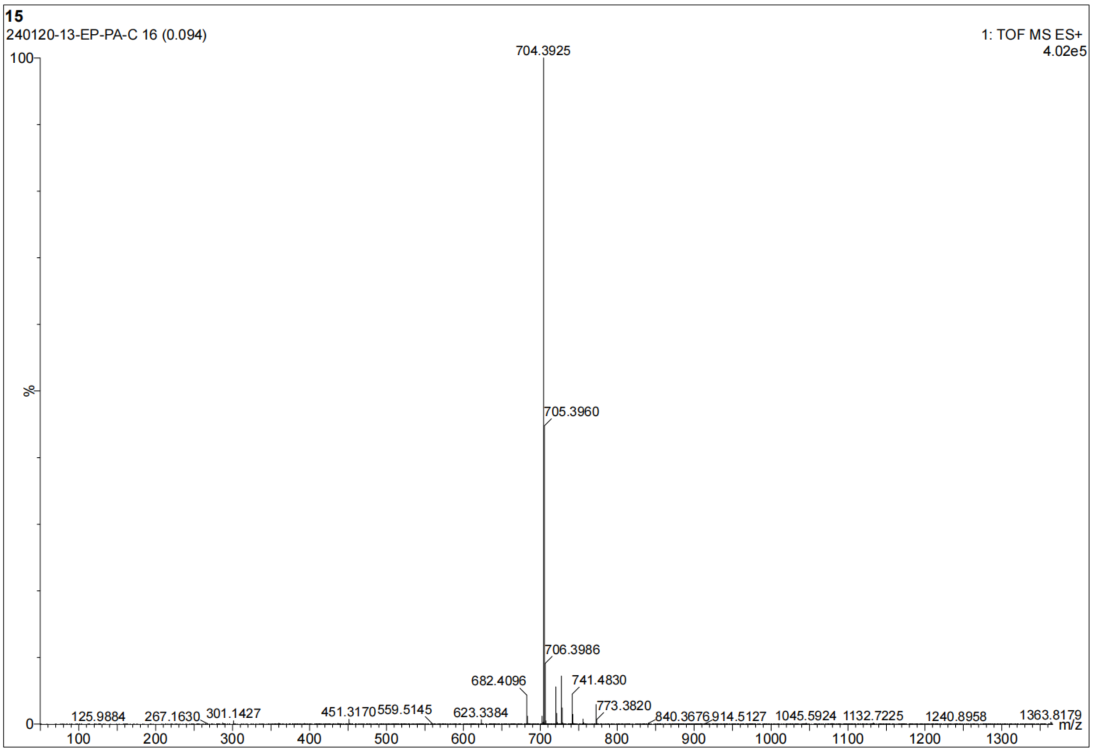

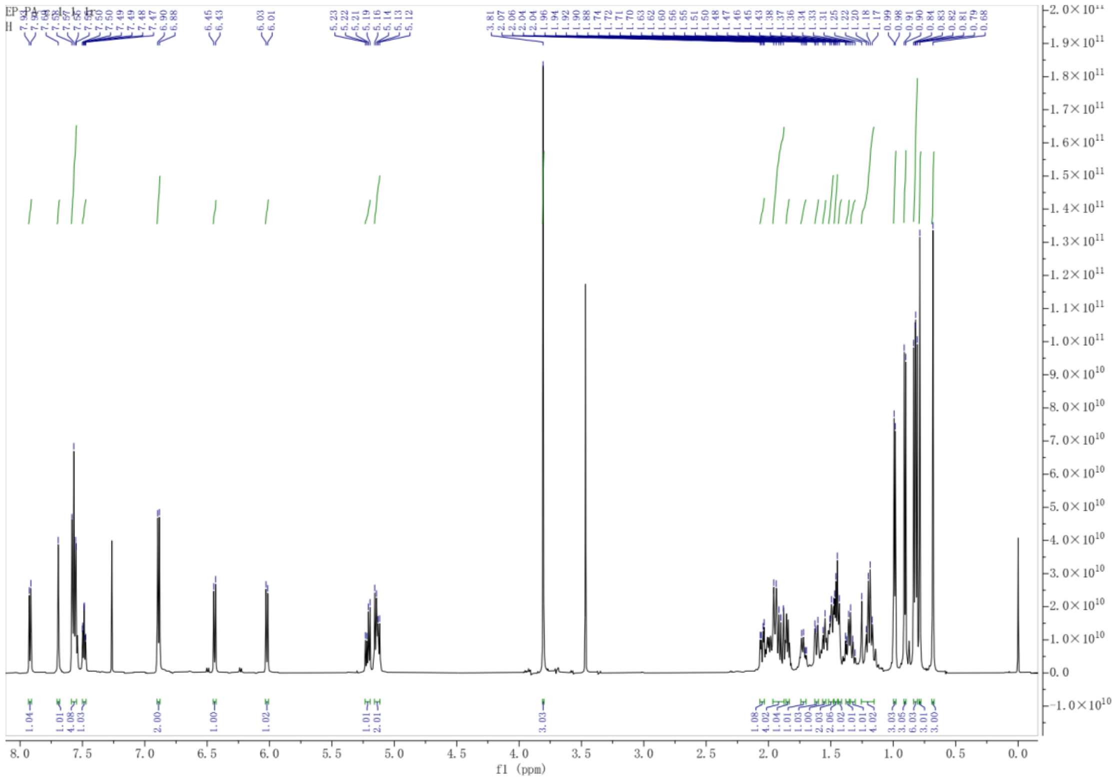

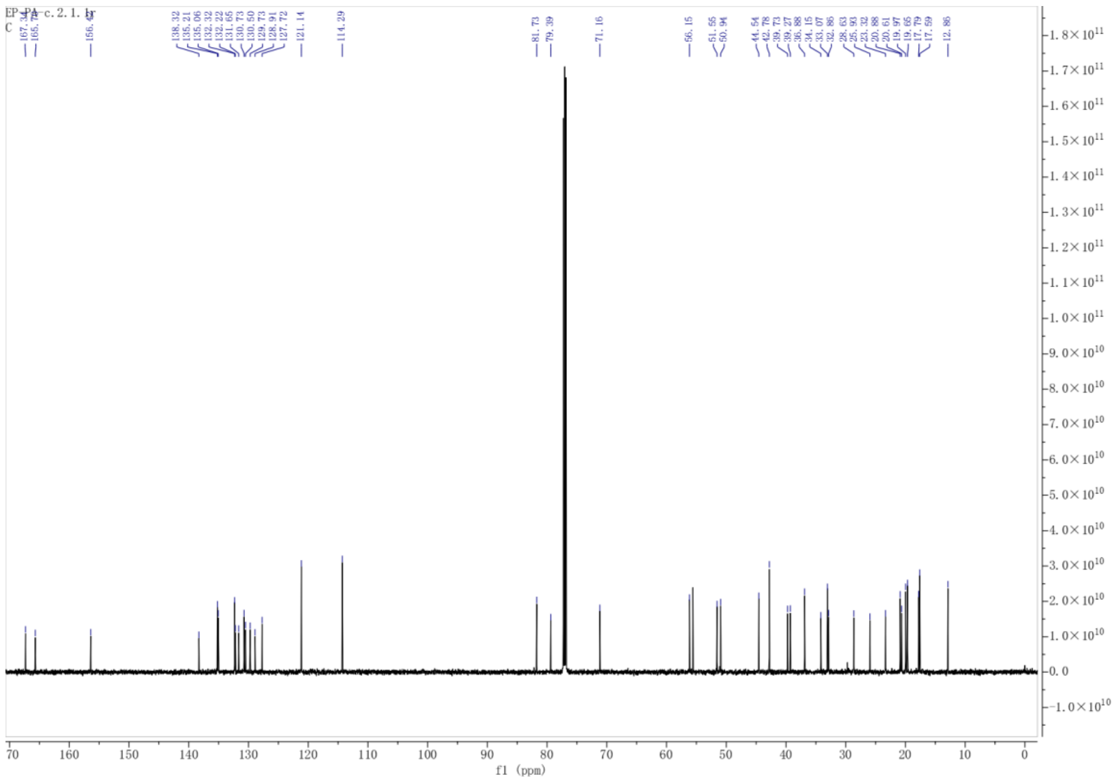

^{1}H NMR, ^{13}C NMR and HRMS spectra of **4h**

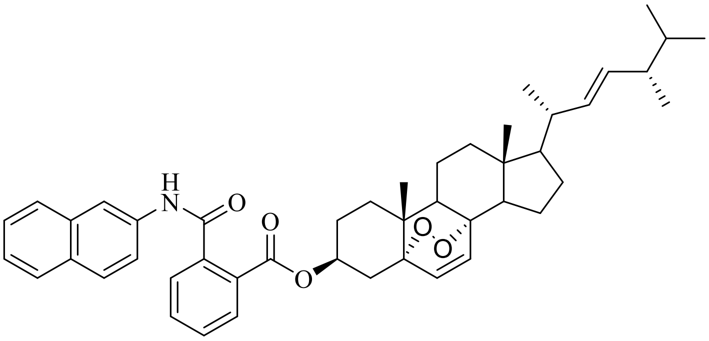

4h

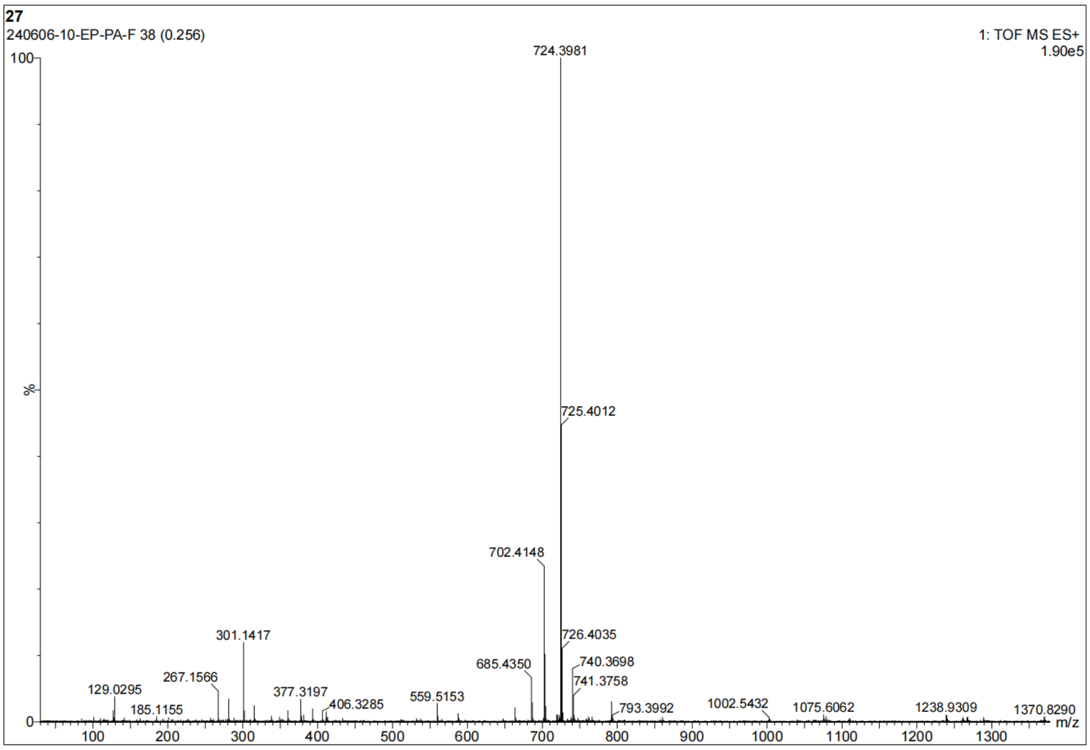

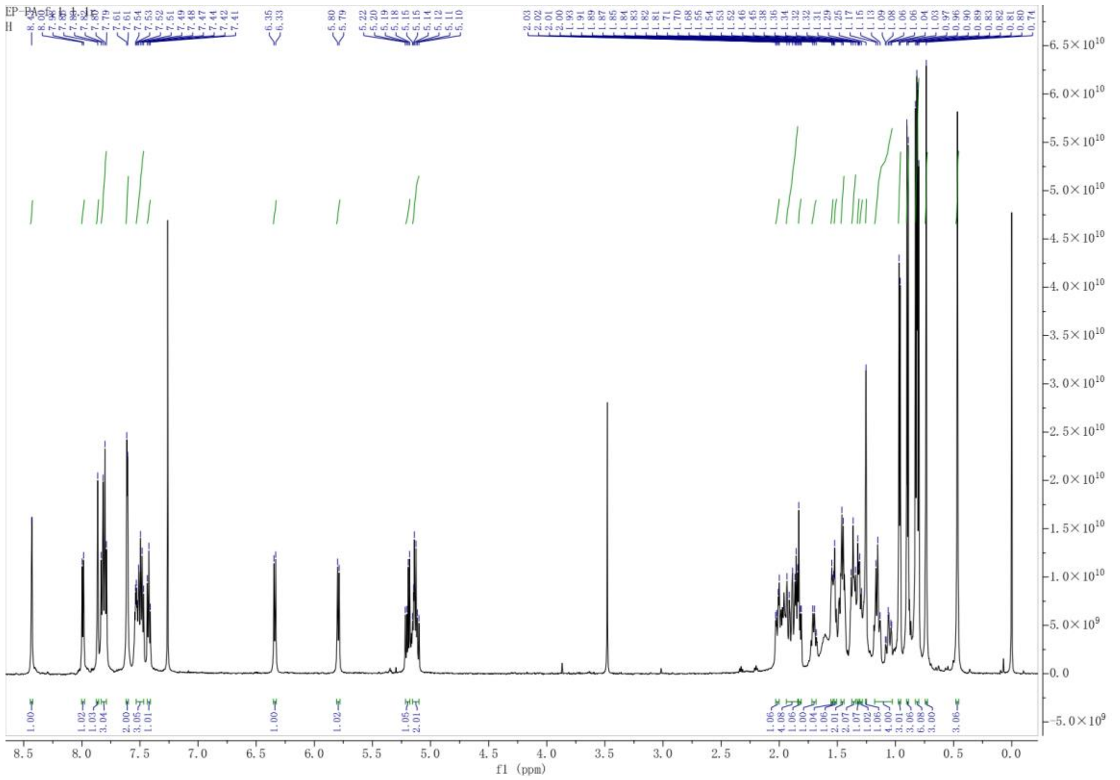

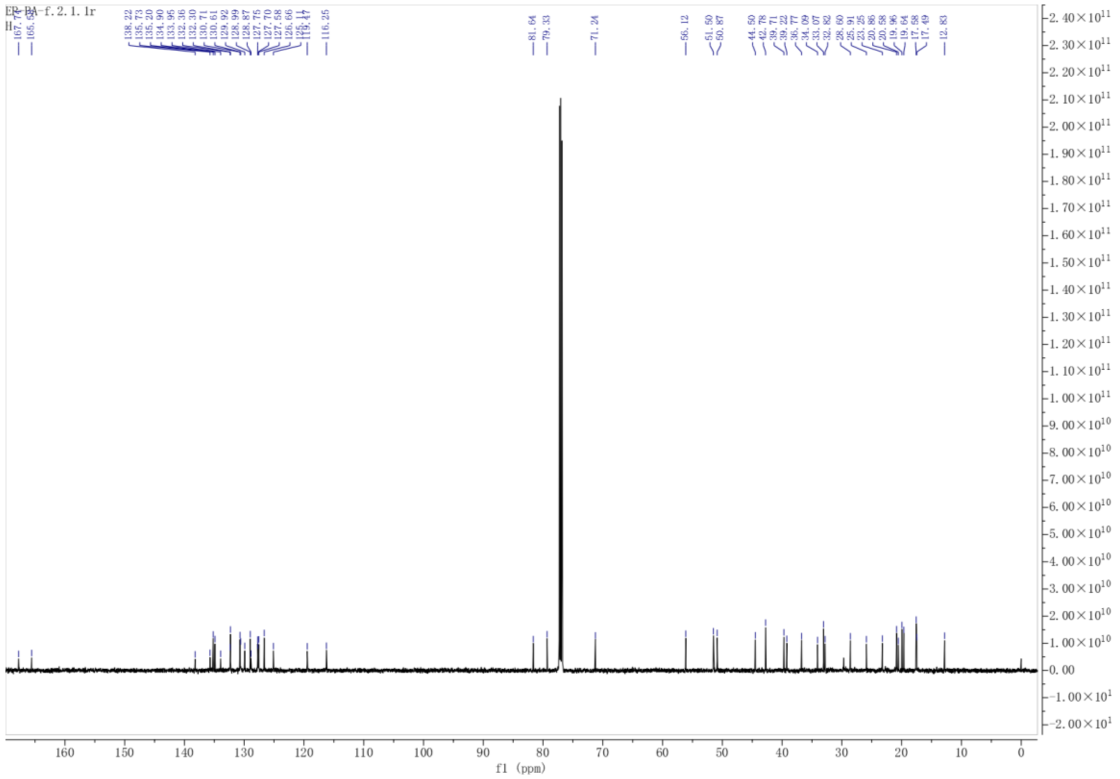

Supplement: Supplementary file 1 [file molecules-29-04375-s001.zip › molecules-3195697-supplementary.pdf]
